# Changes in knowledge of the major harms of tobacco smoking and secondhand smoke exposure among adults who smoke: findings from 26 countries of the International Tobacco Control (ITC) Project (2002–2022) and 32 countries of the Global Adult Tobacco Survey (GATS) (2008–2020)

**DOI:** 10.1136/bmjopen-2025-107667

**Published:** 2026-08-04

**Authors:** Janet Chung-Hall, Geoffrey T Fong, Gang Meng, Lorraine V Craig, Eunice O Indome, for the ITC Project Collaborators, Alissa C Kress, Jing Shi, Indu B Ahluwalia, for the ITC Project Collaborators, Raphaël Andler, Maansi Bansal-Travers, Tonatiuh Barrientos-Gutiérrez, Marcelo Boado, Premduth Burhoo, K Michael Cummings, Cristina de Abreu Perez, Tibor Demjén, Katherine A East, Richard Edwards, Esteve Fernández, Marcela Fu, Coral E Gartner, Fastone M Goma, Prakash C Gupta, A K M Ghulam Hussain, Andrew Hyland, Lawrence Ikamari, Aree Jampaklay, Yuan Jiang, Kota Katanoda, Gil-Yong Kim, Su Young Kim, Sungkyu Lee, Farizah Mohd Hairi, Ute Mons, Nigar Nargis, Amer Siddiq Amer Nordin, Jane Rahedi Ong’ang’o, Maizurah Omar, Mangesh S Pednekar, Krzysztof Przewoźniak, Hong Gwan Seo, James F Thrasher, Antigona Trofor, Constantine I Vardavas, Andrew M Waa, Marc C Willemsen, Nick Wilson, Witold A Zatoński

**Affiliations:** 1 School of Psychology, University of Waterloo, Waterloo, Ontario, Canada; 2 School of Public Health Sciences, University of Waterloo, Waterloo, Ontario, Canada; 3 Ontario Institute for Cancer Research, Toronto, Ontario, Canada; 4 National Center for Chronic Disease Prevention and Health Promotion, Office on Smoking and Health, Atlanta, Georgia, USA; 5 Noninfectious Disease Programs, CDC Foundation Inc, Atlanta, Georgia, USA

**Keywords:** PUBLIC HEALTH, Tobacco Use, Health

## Abstract

**Objectives:**

To assess levels of and trends in knowledge that smoking and secondhand smoke (SHS) cause lung cancer and cardiovascular disease (CVD) in 46 countries over two decades.

**Design:**

Longitudinal cohort and repeat cross-sectional surveys.

**Setting:**

Nationally representative surveys in 26 International Tobacco Control (ITC) Project countries (2002–2022) and 32 Global Adult Tobacco Survey (GATS) countries (2008–2020).

**Participants:**

Adults (aged 18+ years) who smoke tobacco (26 ITC countries: n=133 410; 32 GATS countries: n=110 537).

**Analysis outcomes:**

Current use of cigarettes at least monthly (all ITC countries) and/or bidis at least monthly (ITC India, Bangladesh and Zambia) and current use of any combustible tobacco products (all GATS countries). We conducted individual-level analyses of the ITC and GATS data separately, without combining the datasets or analysing previously published summary statistics. Generalised estimating equations and weighted multinomial regression models assessed country-level prevalence of knowledge for three smoking and two SHS outcomes. Multilevel models estimated mean prevalence across all countries and tested within-country linear trends over time for five disease indicators by survey. Across 12 countries with data from both surveys, correlations between ITC and GATS assessed consistency across indicators.

**Results:**

Knowledge that smoking causes lung cancer was highest (ITC=92.2%, GATS=91.9%), followed by heart disease (ITC=84.7%)/heart attack (GATS=80.0%) and stroke (ITC=75.0%, GATS=66.1%). Knowledge that SHS causes lung cancer (ITC=77.3%, GATS=82.1%) was highest, followed by heart attack (ITC=61.8%)/heart disease (GATS=72.5%) (all p<0.0001). In ITC and GATS countries, the average change in knowledge across the five diseases was +0.14% per year. There was agreement between the ITC and GATS data (range: Pearson correlations=0.60–0.81 across the five indicators).

**Conclusions:**

There is a need to improve and sustain people’s knowledge of diseases caused by smoking and SHS, especially CVD risks.

**STRENGTHS AND LIMITATIONS OF THIS STUDY**

- This study analysed nationally representative surveys from 46 countries—the International Tobacco Control (ITC) Project and the Global Adult Tobacco Survey (GATS)—to assess levels of and trends in knowledge of lung cancer and cardiovascular diseases over two decades.
- Use of self-reported data may be subject to recall bias.
- Generalisability of the findings is limited by low response rates in some ITC Surveys and by the restriction of ITC-GATS correlation analyses to 12 countries.

## Introduction

Over 8 million people die from tobacco-caused diseases per year; tobacco smoking is the single most preventable cause of death globally.^1^ The WHO Framework Convention on Tobacco Control (FCTC), entered into force in 2005, calls on Parties (183 to date) to implement evidence-based measures to reduce tobacco use.^2^ The WHO MPOWER package includes implementation of six evidence-based measures at country-level: **M**onitor tobacco use and policies; **P**rotect people from tobacco smoke; **O**ffer to help quit tobacco use; **W**arn about dangers of tobacco; **E**nforce bans on tobacco advertising, promotion, and sponsorship; and **R**aise tobacco taxes.^3^ The W strategies include the display of pictorial health warnings on 75% of the surface of cigarette packs^4 5^ and mass media campaigns to increase knowledge of tobacco-caused health risks.^6 7^

Knowledge of the harms of smoking is positively associated with quit intentions,^8^ attempts^9^ and cessation success.^10^ People who smoke and are unaware of the health risks of active smoking may be less likely to take steps to protect themselves from firsthand smoke exposure. Similarly, low awareness of the harms of secondhand smoke (SHS) exposure may reduce efforts to protect others around them from SHS.^7^ Despite efforts to inform people about the harms of smoking and SHS, gaps in knowledge remain. Studies have found that adults in the general population^11^ and those who use cigarettes^12–15^ and other smoked tobacco products^15^ report higher knowledge that smoking causes lung cancer compared with heart disease and/or stroke. They are also more likely to know that SHS exposure causes lung cancer as compared with heart disease.^14 15^ The 2023 WHO International Classification of Diseases 11th Revision defines heart disease and heart attack as circulatory system diseases and stroke as a cerebrovascular disease. In this paper, the term cardiovascular disease (CVD) will be used to refer to cardiovascular (coronary heart disease and myocardial infarction) and/or cerebrovascular disease (stroke) to allow for more readable text.

Limited studies have examined health risk perceptions of SHS. Single-country cross-sectional studies have found variation in knowledge of diseases caused by SHS across countries. Few studies have examined the risk perceptions of SHS. For example, studies from Canada^16^ and the UK^17^ have found high awareness that SHS is a risk factor for cancer among adults in the general population. In China, adults in the general population reported higher knowledge that SHS causes lung cancer compared with heart disease and respiratory disease.^18^ Similarly, adult males who smoke in Malaysia had limited knowledge of the risks of heart disease and respiratory diseases from SHS.^19^ In contrast, adults who do not smoke in Nigeria had high awareness that SHS causes cancer, CVD and respiratory disease.^20^ These country differences may reflect contextual determinants of SHS exposure—such as the adoption of smoke-free homes, implementation and enforcement of smoking bans in public places and SHS mass media campaigns^21–24^—which may in turn influence knowledge of SHS-caused diseases.

Few studies have examined changes in knowledge of smoking-caused and SHS-caused diseases. A repeated cross-sectional study found significant increases from 1999 to 2006 in risk perceptions of SHS for lung cancer and heart disease among adults of the Irish general adult population.^25^ One Global Adult Tobacco Survey (GATS) study in six countries between 2008 and 2016 found significant increases in knowledge that smoking causes lung cancer, heart attack and stroke among people aged 15+ years who smoke.^15^

This study assessed knowledge and changes in knowledge over time about key chronic diseases caused by smoking tobacco and SHS exposure among adults who smoke across 46 countries. Indicators included knowledge that smoking causes lung cancer, heart disease/heart attack and stroke in people who smoke and that SHS causes lung cancer and heart disease/heart attack in people who do not smoke.

## Methods

### Study design and participants

Data were from the International Tobacco Control (ITC) Project (ITC Country Surveys) and the Global Adult Tobacco Survey System (GATS).

ITC Project longitudinal cohort surveys with replenishment are administered in 31 countries using a standardised set of questions, data collection protocol and sampling design that allows for cross-country comparisons. For this study, we used ITC Project Survey data from nationally representative samples of adults (aged 18+ years) who smoke tobacco in 26 countries between 2002 and 2022: Australia, Bangladesh, Brazil, Canada, China, England/UK (caution is warranted in the comparison of ITC Survey data from the UK (which includes England, Wales, Scotland and Northern Ireland; from 2002 to 2010–2011) and England (2020). However, England accounts for ~85% of the population of the UK and smoking prevalence and tobacco control policies are similar across all four countries that comprise the UK. As such, we combined data from the UK and England for analysis, as approved by ITC Project principal investigators), France, Germany, Greece, Hungary, India, Japan, Kenya, Malaysia, Mauritius, Mexico, the Netherlands, New Zealand, Poland, Republic of Korea, Romania, Spain, Thailand, the US, Uruguay and Zambia (online supplemental table 1). Adults who smoke tobacco were defined as persons aged 18+ years who smoke tobacco, including those who currently smoke manufactured and/or roll-your-own cigarettes at least monthly and had smoked at least 100 cigarettes in their lifetime (all countries) and/or currently smoke bidis at least monthly (India, Bangladesh and Zambia).

10.1136/bmjopen-2025-107667.supp1 Supplementary data

GATS is a nationally representative repeated cross-sectional household survey of persons aged 15+ years that uses a standard core questionnaire, sample design and data collection methods. GATS data included in this study are from adults who smoke tobacco in 32 countries between 2008 and 2020: Argentina, Bangladesh, Botswana, Brazil, Cameroon, China, Costa Rica, Egypt, Ethiopia, Greece, India, Indonesia, Kazakhstan, Kenya, Malaysia, Mexico, Nigeria, Pakistan, Panama, Philippines, Poland, Qatar, Romania, Russian Federation, Senegal, United Republic of Tanzania, Thailand, Turkey, Uganda, Ukraine, Uruguay and Viet Nam (online supplemental table 2). For consistency in analysis across GATS and ITC, an age cut-off of 18+ years was used to define adults who smoke combustible tobacco products (bidis, kreteks, cheroots, manufactured cigarettes, hand-rolled cigarettes, cigars, cigarillos, pipes and waterpipes with tobacco).

Data on knowledge of smoking-caused and SHS-caused diseases were collected from adults who smoked tobacco and participated in at least one wave of the ITC Surveys (n=133 410) and/or the GATS (n=110 537). We used data from all survey years to test for significant change in knowledge and data from the first and last survey waves to compute average yearly change in knowledge. Countries included in the ITC Surveys and/or GATS are listed in online supplemental table 3. Sample demographics and methodological details for ITC Surveys are described elsewhere^26^ and in online individual country technical reports.^27^ Methodological details for the GATS and individual country fact sheets are available online.^28^

This study includes data from 26 countries of the ITC Project (2002–2022) that are available on reasonable request (dataset),^29^ and data from 32 countries of the GATS (2008–2020) that are available in a public open-access repository (dataset) (https://www.gtssacademy.org/).^28^ Data from ITC and GATS countries that were collected and/or became publicly available after these dates were excluded from the analyses. All data relevant to the study are included in the article or uploaded as supplementary information.

### Measures

Knowledge of smoking-caused diseases was assessed by asking participants if they know or believe that smoking causes lung cancer, heart disease/heart attack and stroke. Respondents were also asked if they know or believe that SHS causes lung cancer and heart disease/heart attack in people who do not smoke. Responses were coded as ‘yes’ versus ‘no/don’t know’. Response ‘refused’ was excluded from the analysis. ITC Survey and GATS questions and response options are presented in online supplemental table 4. Questions for all five disease outcomes were not included at every survey wave across all 26 ITC and 32 GATS countries. We used the most recent data available for each country to calculate point estimates and all-country means of the prevalence of knowledge for each disease outcome, and all longitudinal data to assess changes in knowledge over time.

### Statistical analysis

We conducted individual-level analyses of the ITC and GATS data separately, without combining datasets or analysing previously published summary statistics. Point estimates of the prevalence of knowledge for each disease outcome by country were calculated using weighted multinomial survey logistic regression models for pooled repeated cross-sectional GATS data and generalised estimating equations (GEE) for pooled longitudinal ITC data. All models incorporated survey design information (strata and primary sampling units for multi-stage sampling surveys), adjusted for sex, age, smoking status (daily, non-daily) and time-in-sample (ITC countries).

Point estimates of the all-country means of prevalence of knowledge for each disease outcome across all ITC countries and all GATS countries, respectively, were calculated using a multilevel model (two levels: individual and country), with random intercepts at the country level and roughly equal weights for each country. These point estimates cannot be used to make inferences to the global population. As such, we report the estimated all-country means of knowledge for each health effect without the associated CIs, which would refer to a hypothetical superpopulation of countries. The all-country mean estimates in this analysis, therefore, only serve as the central point of selected countries. GEE and survey logistic regression models were used to test statistical differences in comparing knowledge between the five health effects among respondents in ITC and GATS countries, respectively.

We estimated changes in knowledge for each outcome among adults who smoke in countries with at least two waves of data between 2002 and 2022 (ITC Project Surveys) and between 2008 and 2020 (GATS). Analysis of the longitudinal ITC data used generalised estimating equations to account for within-subject correlations given that a proportion of individuals participated in more than one survey. Analysis of the repeated cross-sectional GATS data used weighted multinomial survey logistic regression models. Two-level models (individual and country) with random intercepts and random slopes of survey years at the country level were used to test for significant yearly change in knowledge for each health effect within countries. This modelling approach controlled for the number of years between the first and last survey waves and assumed a linear survey year effect within each country.

Data from ITC and GATS countries were pooled and fitted separately to ensure comparability within ITC countries and within GATS countries. Pearson correlations (r) between ITC and GATS mean prevalence estimates of knowledge for each of the five diseases were used to examine the consistency of estimates from different surveys conducted in the 12 overlapping countries.

The predicted marginal standardisation method was used to generate all marginal estimates of the outcomes. Data analyses were conducted using SAS V.9.4 and SAS-callable SUDAAN V.11.0.3. Statistical significance (p<0.05 is considered statistically significant) and CIs were computed at the 95% confidence level.

### Patient and public involvement

Patients and/or the public were not involved in the design, conduct, reporting or dissemination plans of this research.

## Results

### Knowledge of diseases caused by smoking and SHS exposure

Knowledge of diseases caused by smoking among adults who smoke was highest for lung cancer (ITC=92.2%, GATS=91.9%), followed by heart disease (ITC=84.7%)/heart attack (GATS=80.0%) and stroke (ITC=75.0%, GATS=66.1%) (online supplemental table 5). Knowledge that SHS causes lung cancer among people who do not smoke (ITC=77.3%, GATS=82.1%) was higher than for SHS causes heart attack (ITC=61.8%)/heart disease (GATS=72.5%) in people who do not smoke. All differences between knowledge of the five diseases caused by smoking and SHS exposure were significant at p<0.001.

### Knowledge that active smoking causes lung cancer, heart disease/heart attack and stroke

**Figure 1:**
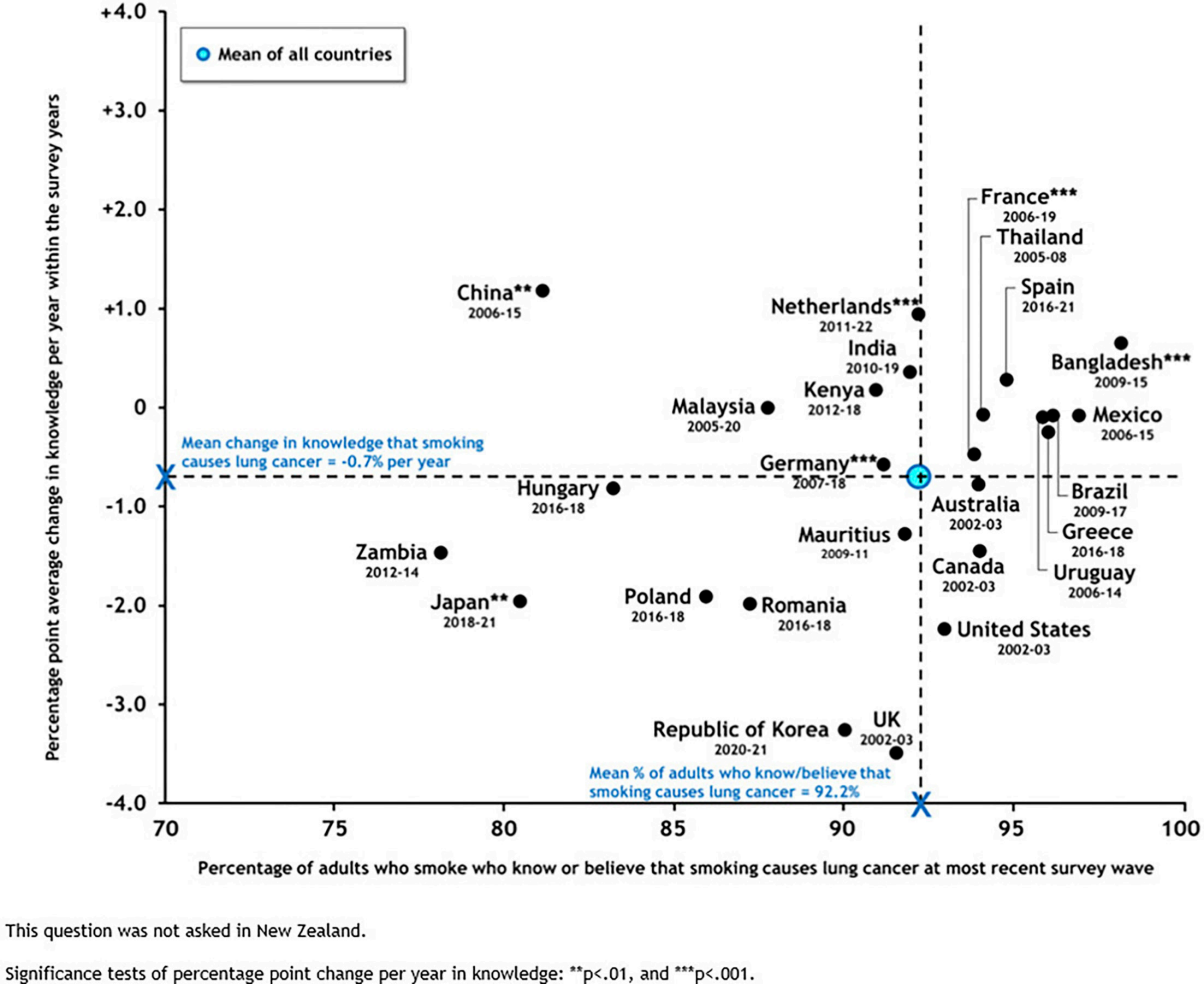
Percentage of adults who smoke who know or believe that smoking causes lung cancer in 25 International Tobacco Control (ITC) Project countries, 2002–2022.

At the most recent survey across 25 ITC countries, 92.2% of adults who smoke overall knew that smoking causes lung cancer, ranging from 98.2% in Bangladesh (2014–2015) to 78.1% in Zambia (2014) (figure 1). Across all ITC countries, knowledge that smoking causes lung cancer decreased by 0.7 percentage points (PP) per year. Knowledge increased significantly in three countries: China (+1.2 PP per year), the Netherlands (+0.9 PP per year) and Bangladesh (+0.6 PP per year). Knowledge decreased significantly in three countries: Japan (−2.0 PP per year), Germany (−0.6 PP per year) and France (−0.5 PP per year) (figure 1). There was no significant change in knowledge in any of the other countries. Detailed results of longitudinal trends in knowledge of smoking-caused diseases in ITC countries are provided in online supplemental table 7).

**Figure 2:**
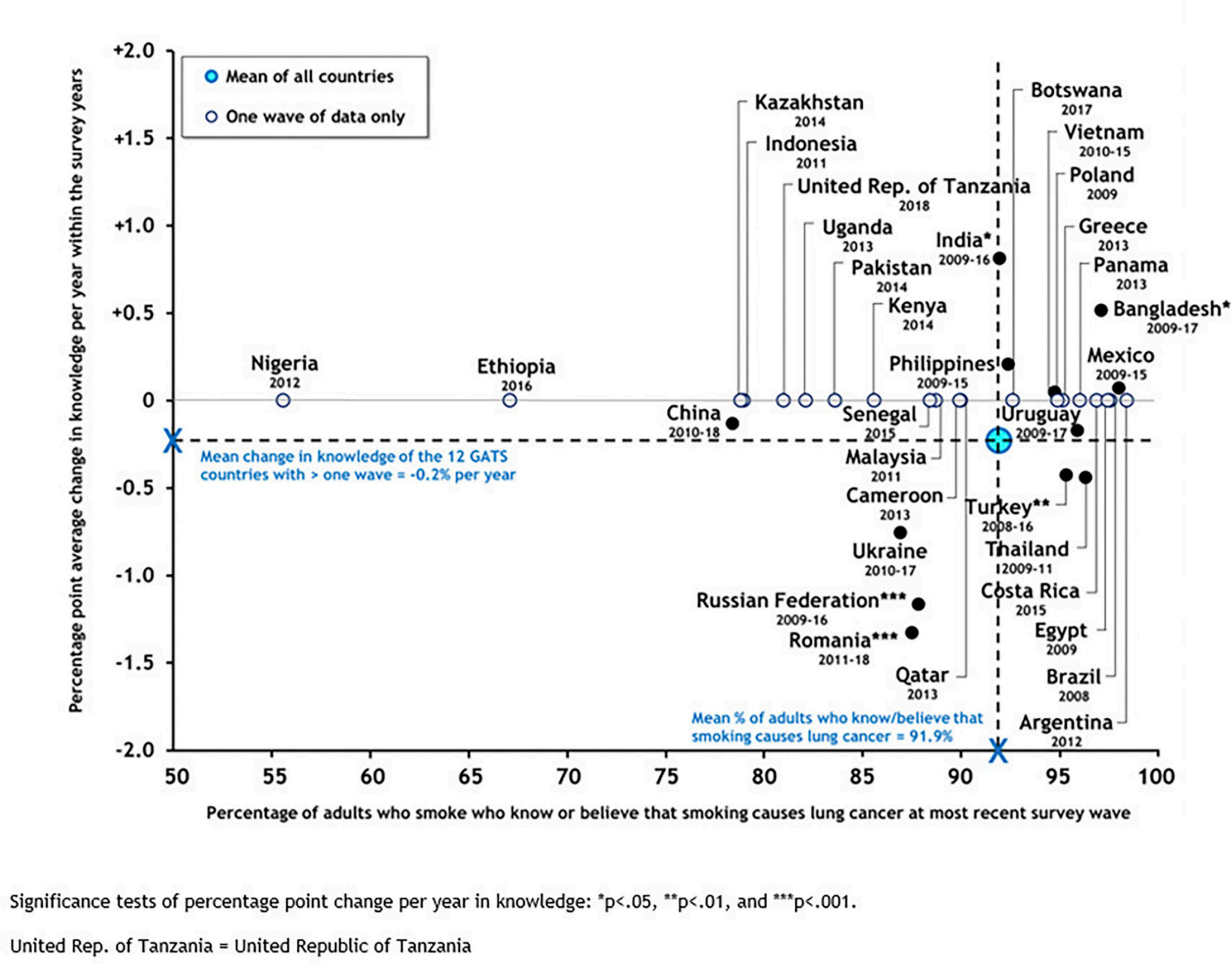
Percentage of adults who smoke who know or believe that smoking causes lung cancer in 32 Global Adult Tobacco Survey (GATS) countries, 2008–2018.

At the most recent survey across 32 GATS countries, 91.9% of adults who smoke overall knew that smoking causes lung cancer, ranging from 98.4% in Argentina (2012) to 56.6% in Nigeria (2012) (figure 2). Across the 12 GATS countries with at least 2 years of data, knowledge that smoking causes lung cancer decreased by 0.2 PP per year. Knowledge increased significantly in two countries: India (+0.8 PP per year) and Bangladesh (+0.5 PP per year). Knowledge decreased significantly in three countries: Romania (−1.3 PP per year), the Russian Federation (−1.2 PP per year) and Turkey (−0.4 PP per year) (figure 2). There was no significant change in knowledge in any of the other countries. For the 20 GATS countries with only one year of data, the change is presented as zero. Detailed results of longitudinal trends in knowledge of smoking-caused diseases in GATS countries are provided in online supplemental table 8.

**Figure 3:**
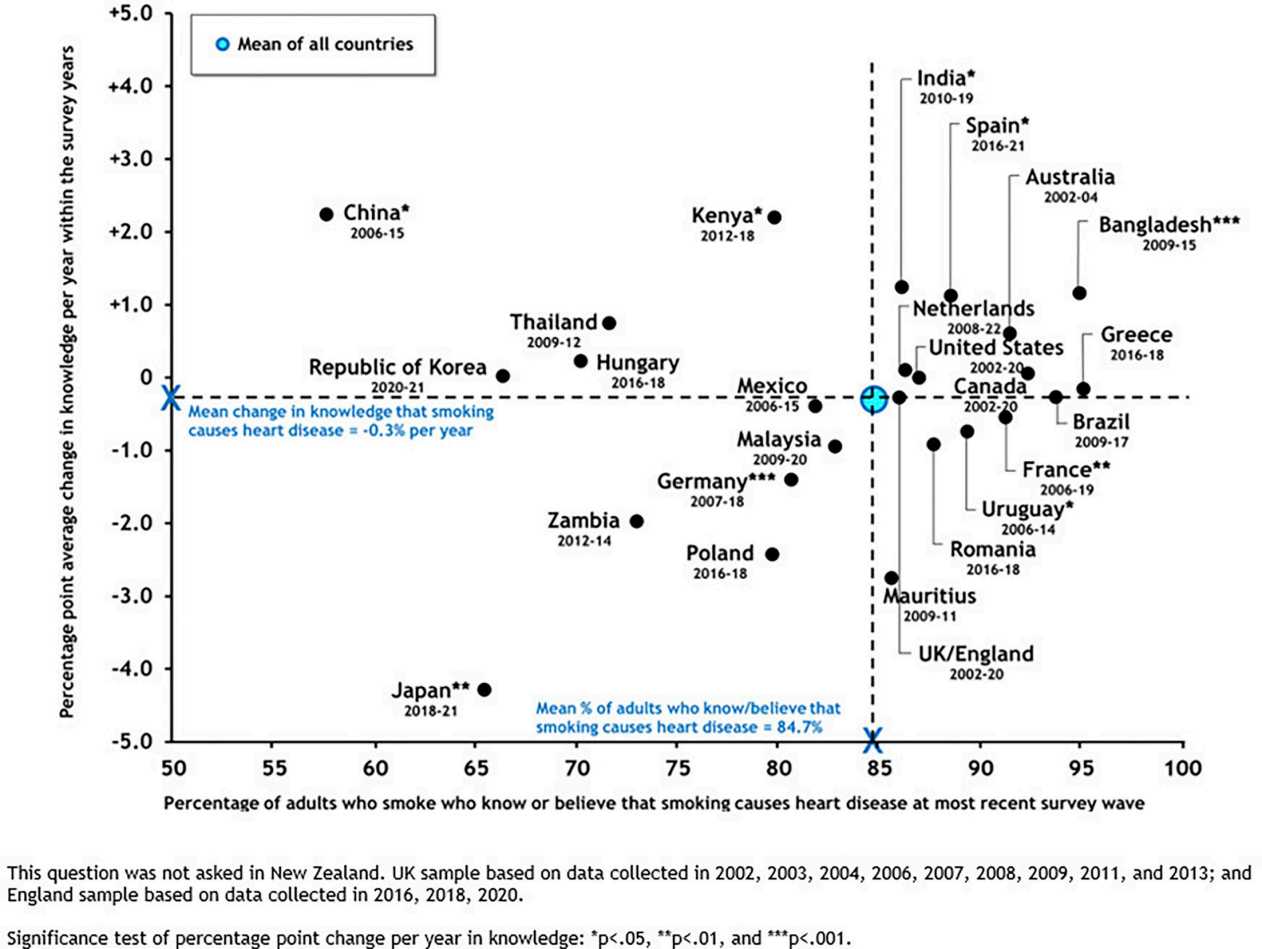
Percentage of adults who smoke who know or believe that smoking causes heart disease in 25 International Tobacco Control (ITC) Project countries, 2002–2022.

At the most recent survey across 25 ITC countries, 84.7% of adults who smoke knew that smoking causes heart disease, ranging from 94.9% in Bangladesh (2014–2015) to 57.7% in China (2013–2015) (figure 3). Across all ITC countries, knowledge that smoking causes heart disease decreased by 0.3 PP per year. Knowledge increased significantly in five countries: China (+2.2 PP per year), Kenya (+2.2 PP per year), India (+1.2 PP per year), Bangladesh (+1.2 PP per year) and Spain (+1.1 PP per year). Knowledge decreased significantly in four countries: Japan (−4.3 PP per year), Germany (−1.4 PP per year), Uruguay (−0.7 PP per year) and France (−0.5 PP per year) (figure 3). There was no significant change in knowledge in any of the other countries.

**Figure 4:**
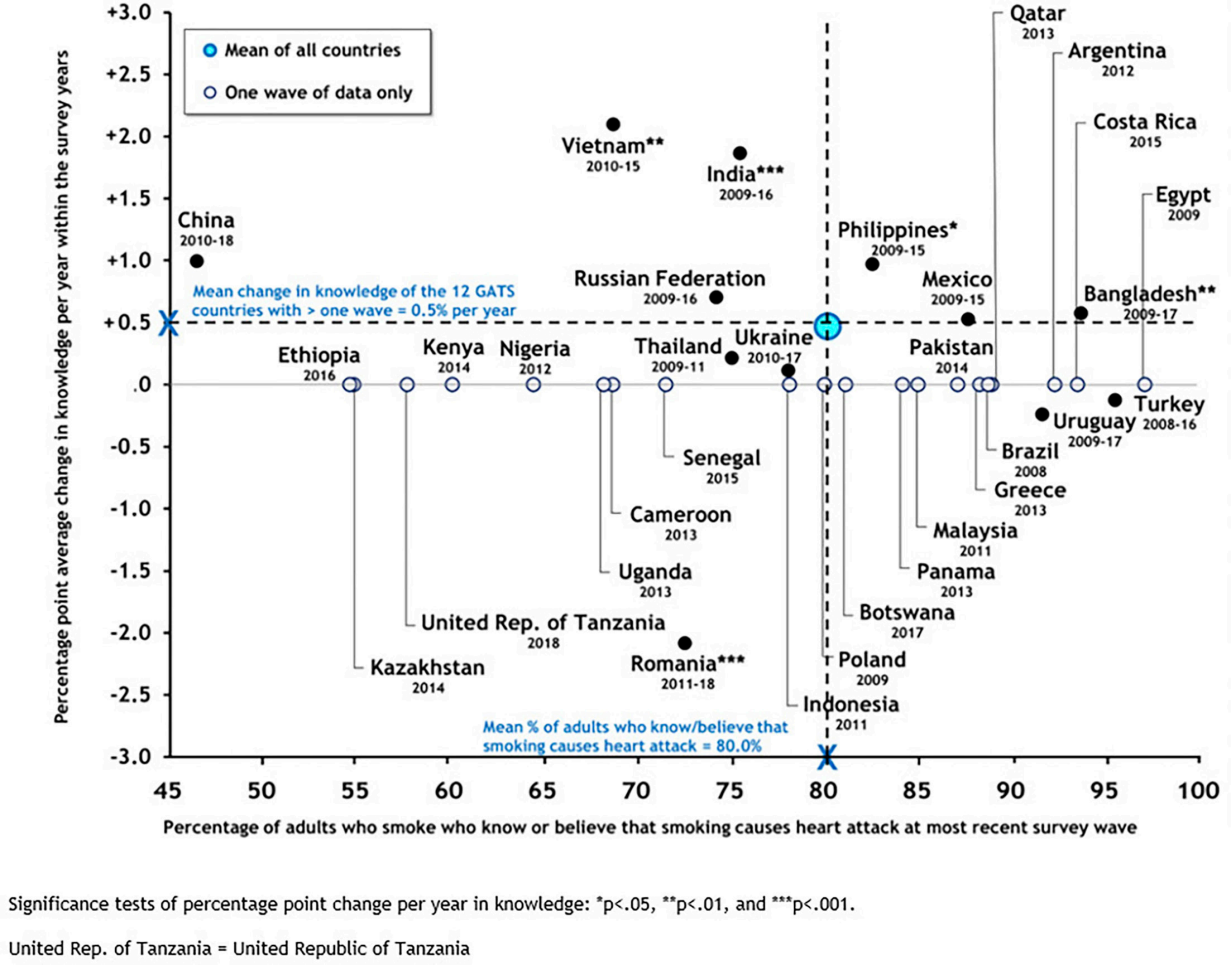
Percentage of adults who smoke who know or believe that smoking causes heart attack in 32 Global Adult Tobacco Survey (GATS) countries, 2008–2018.

At the most recent survey across 32 GATS countries, 80.0% of adults who smoke knew that smoking causes heart attack, ranging from 95.3% in Turkey (2016) to 46.4% in China (2018) (figure 4). Across all GATS countries with at least two years of data, knowledge that smoking causes heart attack increased by 0.5 PP per year. Knowledge increased significantly in four countries: Vietnam (+2.2 PP per year), India (+1.9 PP per year), the Philippines (+1.0 PP per year) and Bangladesh (+0.6 PP per year). Knowledge decreased significantly in Romania (−2.1 PP per year) (figure 4). There was no significant change in knowledge in any of the other countries.

**Figure 5:**
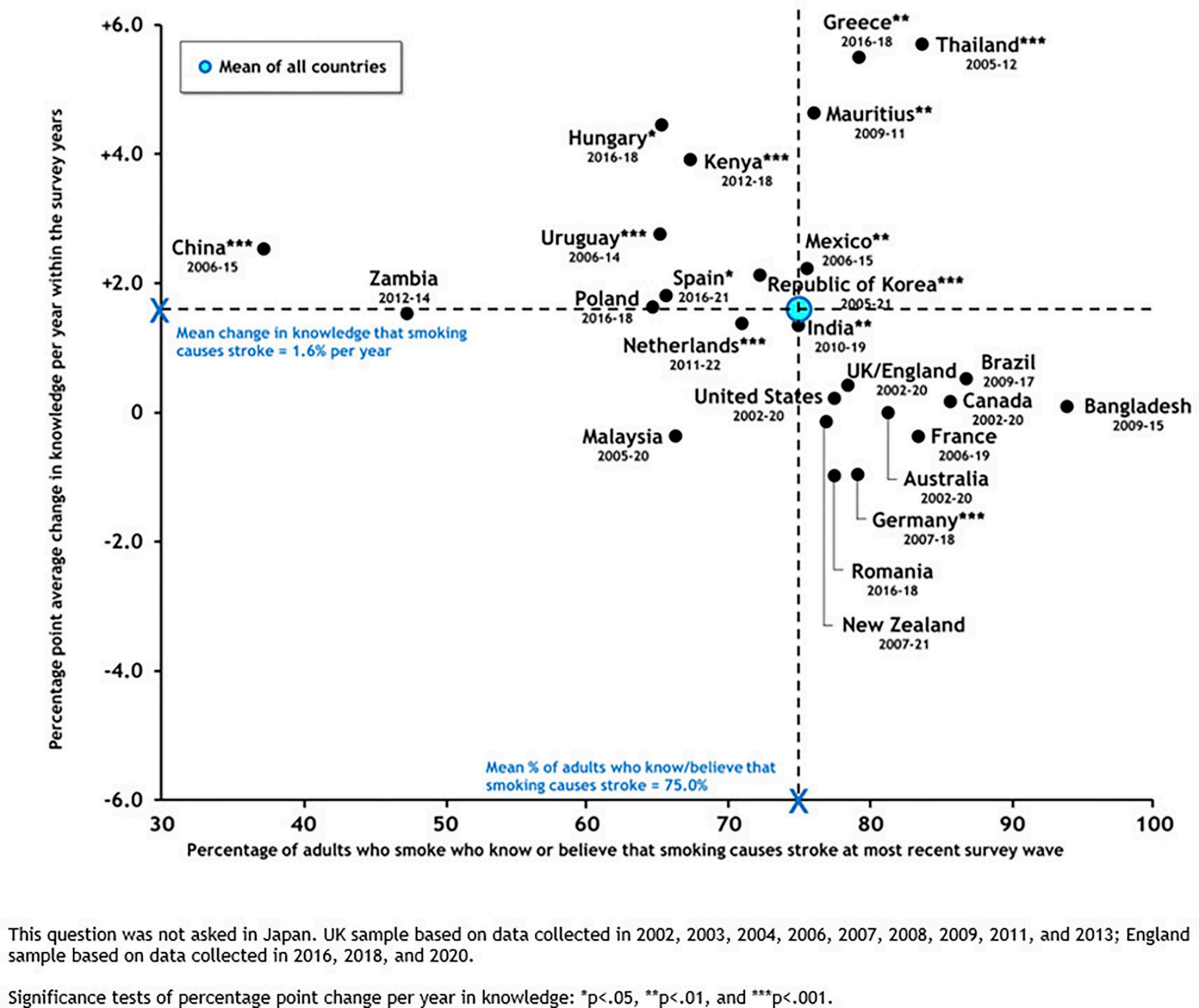
Percentage of adults who smoke who know or believe that smoking causes stroke in 25 International Tobacco Control (ITC) Project countries, 2002–2022.

At the most recent survey across 25 ITC countries, 75.0% of adults who smoke knew that smoking causes stroke, ranging from 93.9% in Bangladesh (2014–2015) to 37.3% in China (2013–2015) (figure 5). Across all ITC countries, knowledge that smoking causes stroke increased by 1.6 PP per year. Knowledge increased significantly in 11 countries: Thailand (+5.7 PP per year), Greece (+5.5 PP per year), Mauritius (+4.6 PP per year), Hungary (+4.5 PP per year), Kenya (+3.9 PP per year), Uruguay (+2.8 PP per year), China (+2.5 PP per year), Mexico (+2.2 PP per year), Republic of Korea (+2.1 PP per year), the Netherlands (+1.4 PP per year) and India (+1.3 PP per year). Knowledge decreased significantly in Germany (−1.0 PP per year) (figure 5). There was no significant change in knowledge in any of the other countries.

**Figure 6:**
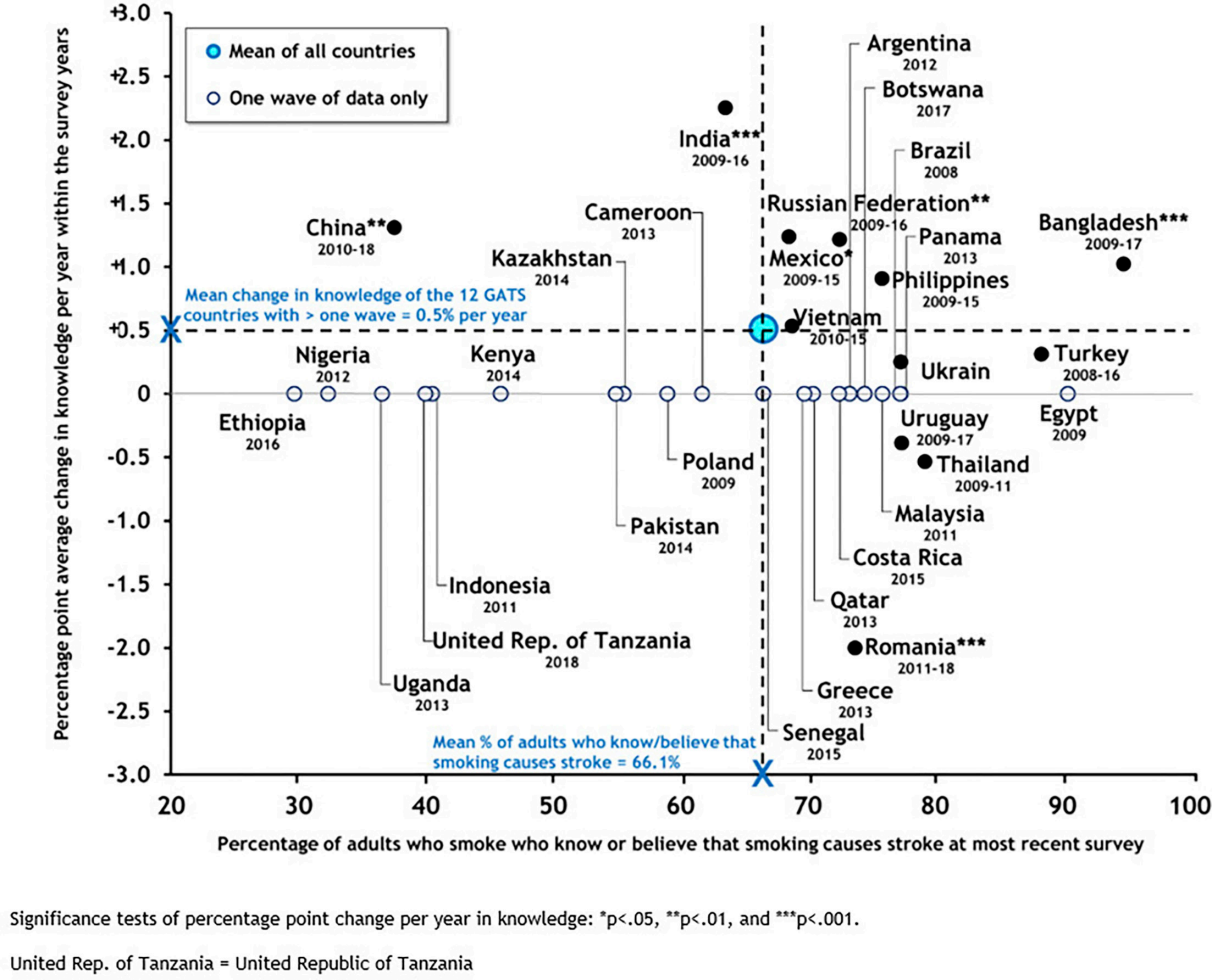
Percentage of adults who smoke who know or believe that smoking causes stroke in 32 Global Adult Tobacco Survey (GATS) countries, 2008–2018.

At the most recent survey across 32 GATS countries, 66.1% of adults who smoke overall knew that smoking causes stroke, ranging from 94.1% in Bangladesh (2017) to 29.6% in Ethiopia^9^ (figure 6). Across all GATS countries with at least 2 years of data, knowledge that smoking causes stroke increased by 0.5 PP per year. Knowledge increased significantly in five countries: India (+2.3 PP per year), China (+1.3 PP per year), the Russian Federation (+1.2 PP per year), Mexico (+1.2 PP per year) and Bangladesh (+1.0 PP per year). Knowledge decreased significantly in Romania (−2.0 PP per year) (figure 6). There was no significant change in knowledge in any of the other countries.

### Knowledge of the risks of SHS exposure causing lung cancer and heart disease/heart attack

**Figure 7:**
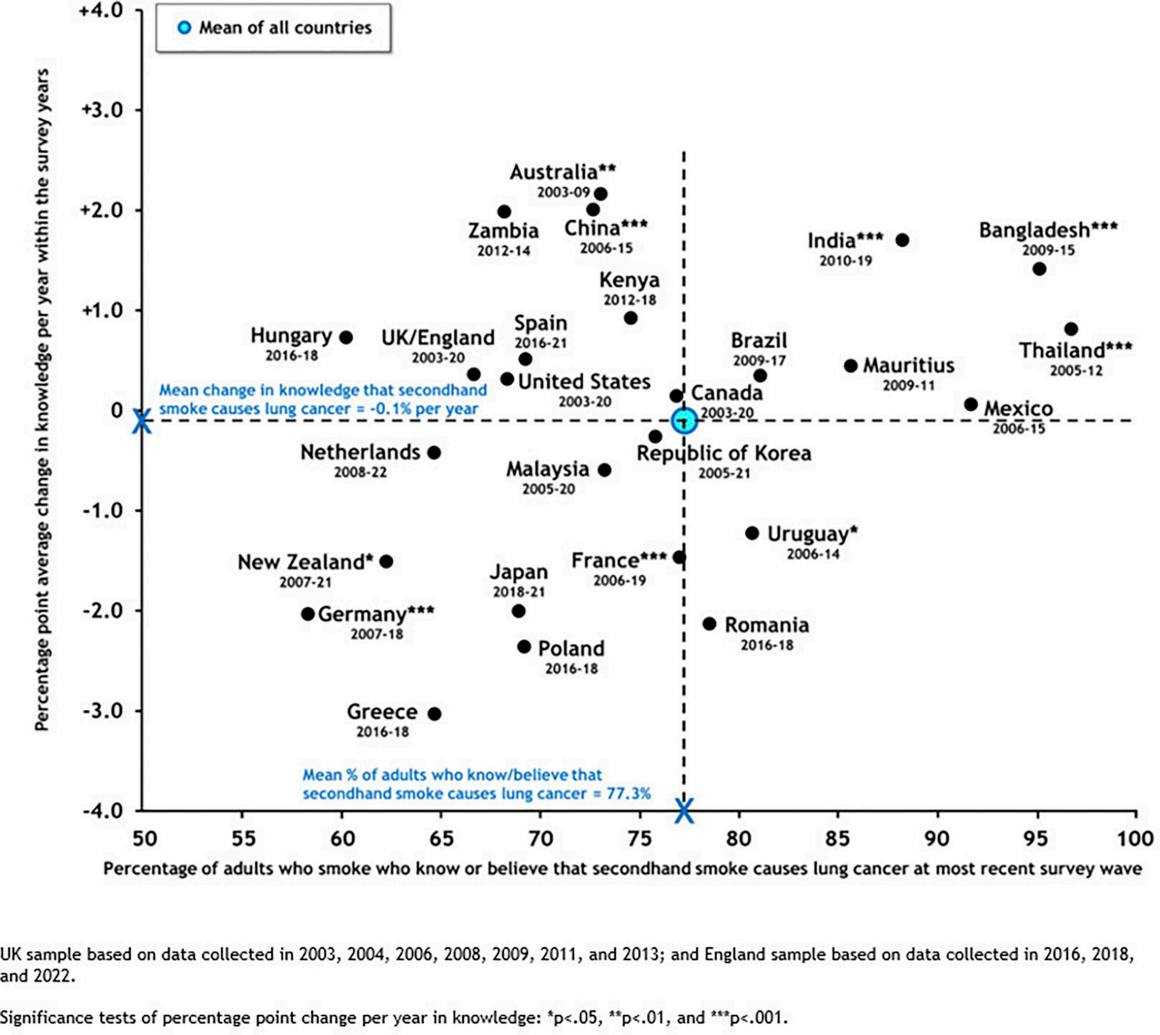
Percentage of adults who smoke who know or believe that secondhand smoke causes lung cancer in 26 International Tobacco Control (ITC) Project countries, 2003–2022.

At the most recent survey across 26 ITC countries, 77.3% of adults who smoke knew that SHS causes lung cancer in people who do not smoke, ranging from 96.8% in Thailand (2012) to 58.4% in Germany (2018) (figure 7). Across all ITC countries, knowledge that SHS causes lung cancer in people who do not smoke decreased by 0.1 PP per year. Knowledge increased significantly in five countries: Australia (+2.2 PP per year), China (+2.0 PP per year), India (+1.7 PP per year), Bangladesh (+1.4% PP per year) and Thailand (+0.8 PP per year). Knowledge decreased significantly in four countries: Germany (−2.0 PP per year), New Zealand (−1.5 PP per year), France (−1.5 PP per year) and Uruguay (−1.2 PP per year) (figure 7). There was no significant change in knowledge in any of the other countries. Detailed results of longitudinal trends in knowledge of SHS-caused diseases in ITC countries are provided in online supplemental table 9.

**Figure 8:**
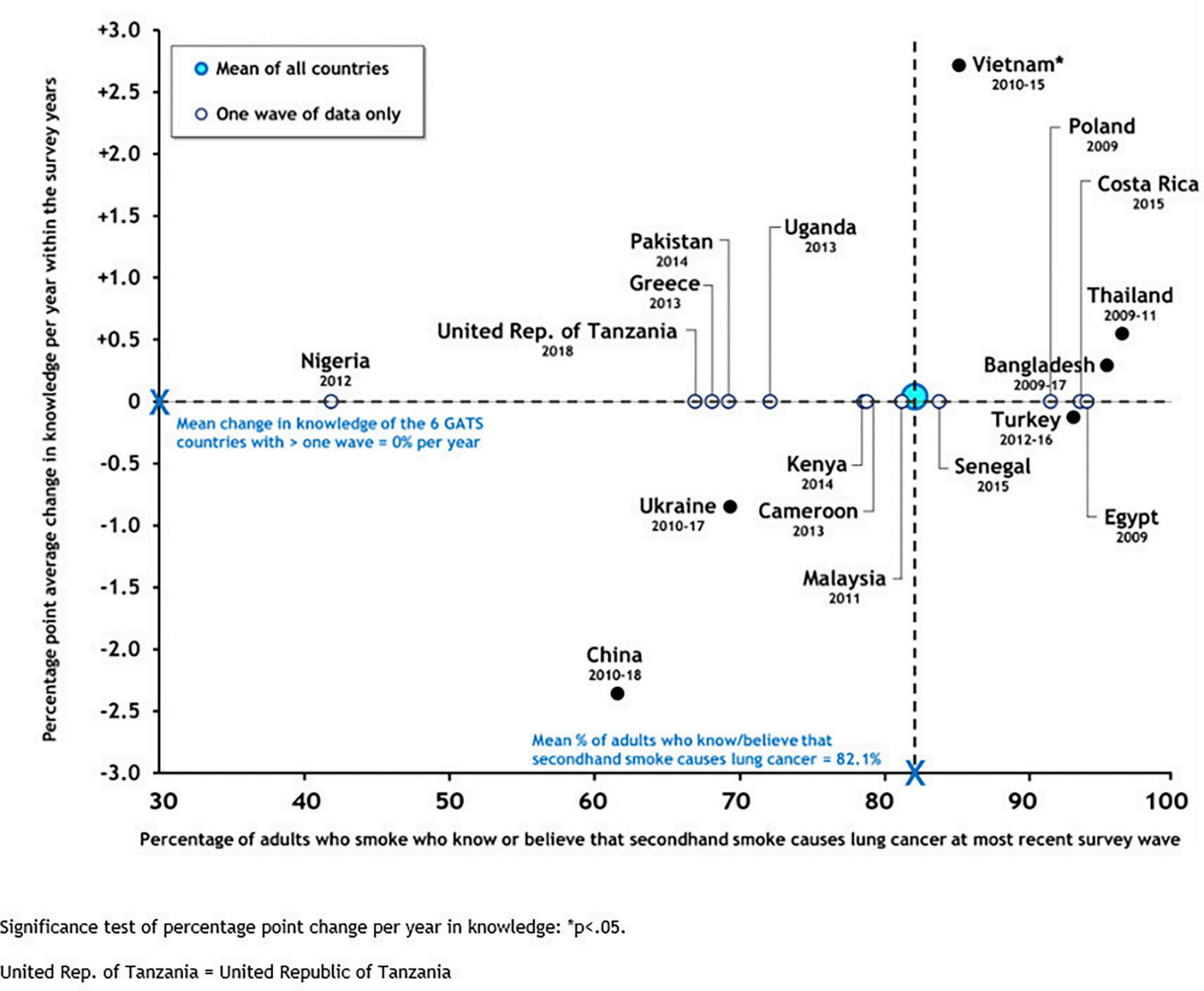
Percentage of adults who smoke who know or believe that secondhand smoke causes lung cancer in 18 Global Adult Tobacco Survey (GATS) countries, 2009–2018.

At the most recent survey across 18 GATS countries, 82.1% of adults who smoke knew that SHS causes lung cancer in people who do not smoke, ranging from 96.4% in Thailand (2011) to 41.9% in Nigeria (2012) (figure 8). Across all GATS countries with at least two years of data, there was no change in knowledge that SHS causes lung cancer in people who do not smoke. Knowledge increased significantly in Vietnam (+2.7 PP per year) (figure 8). There was no significant change in knowledge in any of the other countries. Detailed results of longitudinal trends in knowledge of SHS-caused diseases in GATS countries are provided in online supplemental table 10).

**Figure 9:**
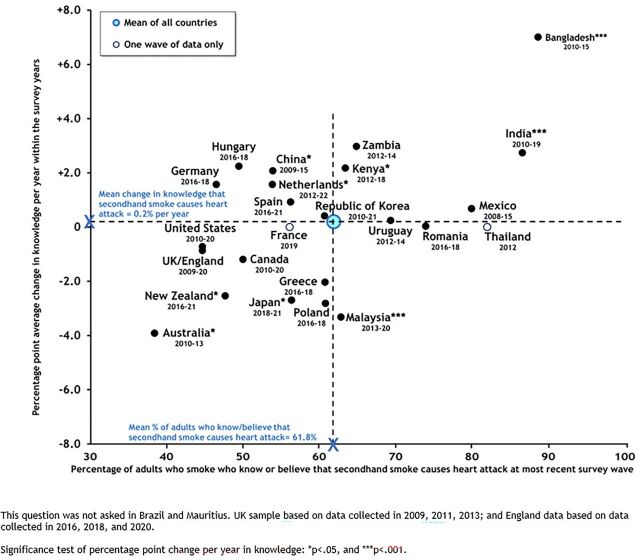
Percentage of adults who smoke who know or believe that secondhand smoke causes heart attack in 24 International Tobacco Control (ITC) Project countries, 2008–2022.

In the most recent survey administered across 24 ITC countries, 61.8% of adults who smoke knew that SHS causes heart attacks in people who do not smoke, ranging from 88.5% in Bangladesh (2014–2015) to 38.4% in Australia (2013) (figure 9). Across all ITC countries, knowledge that SHS causes heart attacks in people who do not smoke increased by 0.2 PP per year. Knowledge increased significantly in five countries: Bangladesh (+7.0 PP per year), India (+2.7 PP per year), the Netherlands (+1.6 PP per year), Kenya (+2.2 PP per year) and China (+2.1 PP per year). Knowledge decreased significantly in four countries: Australia (−3.9 PP per year), Malaysia (−3.3 PP per year), Japan (−2.7 PP per year) and New Zealand (−2.5 PP per year) (figure 9). There was no significant change in knowledge in any of the other countries.

**Figure 10:**
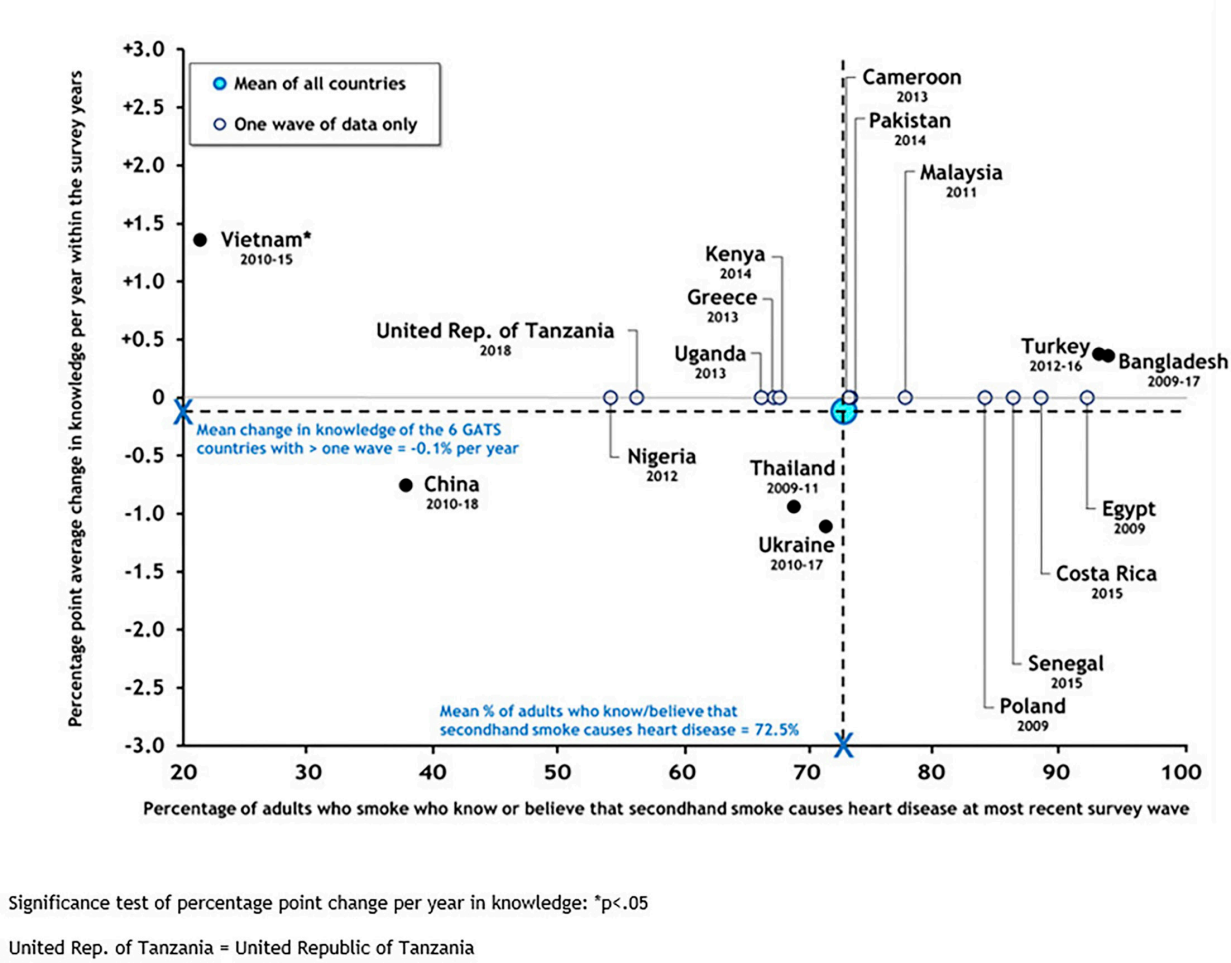
Percentage of adults who smoke who know or believe that secondhand smoke causes heart disease in 18 Global Adult Tobacco Survey (GATS) countries, 2009–2018.

At the most recent survey across 18 GATS countries, 72.5% of adults who smoke knew that SHS causes heart disease in people who do not smoke, ranging from 93.5% in Bangladesh (2017) to 21.3% in Vietnam (2015) (figure 10). Across all GATS countries with at least two years of data, knowledge that SHS causes heart disease in people who do not smoke decreased by 0.1 PP per year. Knowledge increased significantly in Vietnam (+1.4 PP per year) (figure 10). There was no significant change in knowledge in any of the other countries.

### Comparison of knowledge levels among respondents in ITC and GATS countries

Compared with overall knowledge that smoking causes lung cancer (ITC=92.2%, GATS=91.9%), knowledge that smoking causes heart attack (ITC=84.7%)/heart disease (GATS=80.0%) and stroke (ITC=75.0%, GATS=66.1%) was lower, as was knowledge of the harms of SHS for people who do not smoke (SHS causes lung cancer: ITC=77.3%, GATS=82.1%; SHS causes heart attack (ITC=61.8%)/heart disease (GATS=72.5%) (all p<0.0001) (online supplemental table 5).

Knowledge that smoking causes stroke was the only 1 of 5 harms where there was an increase in both ITC and GATS over time (+1.6 PP in ITC; +0.5 PP in GATS). Knowledge that smoking causes lung cancer was the only health harm where there was a decrease in both ITC and GATS (−0.7 PP in ITC; −0.2 PP in GATS). The average estimated net change in knowledge across the five health harms was +0.14 PP in both ITC and GATS (online supplemental table 6).

### Correlation of ITC and GATS data

In the 12 overlapping ITC and GATS countries, significant positive correlations were found between ITC and GATS estimates of knowledge among adults who smoke that smoking causes lung cancer (r=0.80, p=0.002), heart disease/heart attack (r=0.81, p=0.001) and stroke (r=0.80, p=0.002). For the seven overlapping ITC and GATS countries where knowledge of SHS harms was assessed, moderate but non-significant correlations were found between ITC and GATS estimates of knowledge that SHS causes lung cancer (r=0.69, p=0.09) and heart disease/heart attack (r=0.60, p=0.16) in people who do not smoke.

## Discussion

Overall, there was a strong correlation between ITC and GATS knowledge estimates in countries where both surveys were conducted. There were five key findings from our analyses about knowledge of smoking-caused and SHS-caused diseases in 46 countries across two decades. First, adults who smoke were more likely to know that smoking elevates their risk for lung cancer compared with heart disease/heart attack and stroke. Second, adults who smoke were less likely to know about the risks related to SHS. Third, adults who smoke had higher knowledge that SHS causes lung cancer compared with heart disease/heart attack in people who do not smoke. Fourth, knowledge of smoking-caused and SHS-caused diseases has improved in several countries over time; however, knowledge has generally remained unchanged in most countries and has regressed in others. Finally, there was a near-zero net change over time in knowledge of smoking-caused and SHS-caused diseases (+0.14% per year across five disease outcomes in ITC and GATS countries).

There has been a steady upward trend in the global burden of CVD from 1990 to 2019, with the total number of CVD deaths rising from 12.1 million to 18.6 million, and the total number of disability-adjusted life years due to CVD rising from 17.7 million to 34.4 million over the last three decades.^30^ The largest number of premature non-communicable disease (NCD) deaths globally is caused by CVDs,^31 32^ for which smoking is a major modifiable risk factor. In 2019, heart disease and stroke collectively accounted for 2.6 million smoking-attributable deaths worldwide, compared with 1.3 million deaths from lung, tracheal and bronchus cancer.^33^ A 2024 study found that exposure to SHS significantly increased risk for nine health outcomes, with the greatest risk observed for CVD.^34^ Although smoking and SHS are known risk factors for CVD, adults in many countries still lack knowledge of their harmful effects on cardiovascular health. Consistent with previous studies,^12 14 15^ we found that adults who smoke were more likely to know that smoking causes lung cancer than heart disease and stroke. CVD is still the leading preventable cause of mortality worldwide,^30 35^ and while the relative risk from smoking for CVD is lower than for lung cancer, more people die prematurely from CVD than from lung cancer caused by smoking.^35 36^

SHS is one of the leading causes of preventable death,^37^ killing an estimated 1.3 million people who do not smoke in 2021.^38^ We found that across 40 countries, about 65.0% of adults who smoke knew that SHS exposure causes heart disease/heart attack in people who do not smoke. Notably, even in several high-income ITC countries—including Australia, Germany, England, the US, New Zealand, Canada and Hungary—less than half of adults who smoke knew of this risk, in contrast with higher knowledge that active smoking causes heart disease and stroke.

Knowledge that smoking causes stroke significantly increased in 11 ITC countries and 3 GATS countries between 2002 and 2022. Nevertheless, there were no significant changes in knowledge of stroke in 13 ITC countries and 7 GATS countries. Likewise, there were no significant changes in knowledge among adults who smoke that smoking causes heart disease/heart attack in people who smoke and that SHS exposure causes heart disease/heart attack in people who do not smoke in 14 ITC countries and 5 GATS countries, respectively. While significant gains in knowledge of the CVD risks of smoking tobacco and/or SHS exposure were observed in several low-and-middle income countries (LMICs) such as Bangladesh, India, Thailand and Vietnam, knowledge levels have remained low in others such as Nigeria and Ethiopia. Significant declines in knowledge of some of these risks were also observed in several high-income countries, where baseline risk knowledge was relatively high, including Germany and Japan. This suggests that measures to educate people about the diseases caused by smoking may have a greater impact in countries where knowledge is low compared with those where knowledge is already high. It is key for countries to tailor interventions to address knowledge gaps.

Country-level differences observed may reflect differences in public health education strategies and implementation methods regarding the harms of smoking. For example, health warnings on cigarette packs, the size of warnings and languages used are one way of informing people about diseases caused by smoking, which may encourage those who smoke to reduce consumption or take steps to quit.^39–41^ It is possible that people who smoke may have higher levels of knowledge of specific health risks of smoking that were depicted on cigarette pack warnings at the time of the ITC and GATS country surveys compared with health risks that were not addressed by the warnings.

However, knowledge alone may be insufficient to change behaviour. Social Cognitive Theory provides a theoretical framework for understanding the underlying mechanisms of health behaviours and informing the development of interventions, whereby behaviour is determined by reciprocal interactions between personal, environmental and behavioural factors.^42^ For example, exposure to health warnings on cigarette packs in combination with knowledge of smoking-caused and SHS-caused diseases, self-efficacy to reduce or quit smoking and supportive environments such as smoke-free homes, workplaces and public places can facilitate smoking cessation and protection against SHS exposure. Future research should examine the associations between knowledge of smoking-caused diseases and health warnings (type (text-only or pictorial), content, size and location (front and/or back of pack)) as well as other tobacco control policies, which were beyond the scope of the current study.

Since the WHO FCTC came into force in 2005, countries around the world have made significant progress in educating their populations about the dangers of tobacco use and SHS exposure through the implementation of tobacco advertising, promotion and sponsorship bans,^43^ health warnings on tobacco packaging,^44^ smoke-free laws^45^ and anti-tobacco campaigns,^44^ which have likely contributed to reductions in the tobacco use prevalence from 29.3% in 2005 to 20.9% in 2022.^45 46^ However, we found no overall change (+0.14% per year) in knowledge across five smoking-caused and SHS-caused diseases in both ITC and GATS countries over a two-decade period, even though 43 of the 46 countries included in the present study are WHO FCTC Parties.^47^ This may reflect an uneven pace in the implementation of MPOWER measures—including taxation, health warnings on cigarette packs and mass media campaigns—that are especially effective for communicating smoking-caused risks^45^ and are most impactful when they are integrated as a part of a comprehensive tobacco control strategy.^48^ For example, the impact of smoke-free laws on knowledge of SHS-caused diseases could potentially be strengthened by complementary mass media campaigns, which have been shown to increase knowledge of SHS risks, reduce smoking in homes and promote smoking cessation.^23^

Tobacco control policies may be more effective at improving knowledge of smoking and SHS-caused diseases when supported by strong implementation, enforcement and compliance; however, substantial gaps remain. Studies have found weak implementation, limited accountability and poor enforcement of smoke-free laws in LMICs after WHO FCTC ratification,^49^ and a 2024 systematic review and meta-analysis reported high levels of non-compliance with smoke-free laws, particularly in public places and workplaces.^50^ These findings align with an assessment of the WHO FCTC at its 20th anniversary, which concluded that progress has slowed despite earlier gains due to tobacco industry interference, limited political will and resources and the rise of new nicotine products, highlighting the need for best-practice implementation, regulation and intergovernmental cooperation.^51^

It is also important to note that reductions in mortality and morbidity from smoking-caused and SHS-caused diseases may influence public perceptions of their harms, particularly in countries with declining smoking prevalence. Moreover, advances in healthcare and effective treatments in high-resource countries could reduce the disease burden and improve quality of life even in the absence of smoking prevention policies, potentially contributing to low population knowledge of the harms of smoking and SHS. This underscores the importance of prioritising resources for tobacco use prevention rather than focusing solely on treatment.

This study has limitations. First, self-reported data may be subject to recall bias that might result in overestimation or underestimation of knowledge or social desirability bias. Second, some ITC Survey response rates were low due to the nature of the mode and/or protocols for data collection (online supplemental table 1), which may limit the generalisability of the study findings. Third, we found statistically significant positive correlations for all three smoking-caused diseases in the 12 overlapping ITC and GATS countries, while non-significant correlations were found for the two SHS-caused diseases due to reduced power from the small sample size (n=7 observations). Caution is warranted in the comparison of ITC and GATS estimates due to differences in research designs, sampling across ITC Surveys and between ITC Surveys and GATS, survey year(s) in overlapping ITC and GATS countries and the ITC Survey and GATS questions used to measure knowledge (online supplemental tables 1, 2 and 4). However, correlation of the ITC and GATS data despite their different research and methodological designs allows for more confident inferences.^52 53^ Fourth, ITC and GATS data correlation analyses were limited to 12 countries, so the results are not generalisable. Finally, our results are limited to knowledge of the harms of smoking among adults who currently smoke tobacco. Our finding for an overall near-zero net change in knowledge over time may or may not reflect changes in overall knowledge in the population. It is possible that while knowledge of diseases caused by smoking and SHS among adults who smoke did not change over time, knowledge may have increased among adults who have quit smoking and those who have never smoked. As such, we examined whether changes in knowledge for each disease outcome differed by smoking status across 12 GATS countries where more than one survey wave was conducted among adults who smoke, adults who have quit smoking and adults who have never smoked (ITC longitudinal cohort surveys primarily recruit adults who smoke at baseline. Respondents who quit smoking at subsequent survey waves may be recontacted as part of the longitudinal cohort; however, these individuals do not constitute a population-representative sample of adults who have quit smoking. Although these respondents retain adults who smoke-based survey weights for longitudinal analyses (to represent the previous-wave population of adults who smoke), they are analytically classified as adults who quit and are not included as adults who currently smoke in our analyses. For this reason, analyses comparing changes in knowledge by smoking status (adults who currently, formerly and never smoked) were conducted using GATS data only). In general, there were no consistent patterns for changes in knowledge of each disease outcome across the 12 GATS countries (online supplemental table 5). Non-significant increases/decreases in knowledge over time among adults who previously smoked and those who have never smoked suggest that this pattern among people who smoke is consistent with that in the overall population.

This study found high knowledge that smoking causes lung cancer among adults who smoke, lower knowledge that smoking causes CVD and even lower knowledge of SHS harms. There was no overall change in knowledge over two decades. The United Nations 2030 Agenda for Sustainable Development calls for strengthening implementation of the WHO FCTC to achieve the target for a one-third reduction in premature mortality from NCDs by 2030.^54^ To achieve this, countries could consider implementation of WHO FCTC measures shown to have synergistic effects on promoting smoking cessation and reducing smoking prevalence.^55 56^ An important factor that can facilitate efforts to strengthen policies is to increase awareness of the harms of using cigarettes and other smoked tobacco products.

## Conclusions

Across 46 countries over two decades, adults who smoke had high knowledge that smoking causes lung cancer, substantially lower knowledge of cardiovascular harms and even lower awareness of SHS-caused diseases. The near-zero net change in knowledge over time underscores the need for comprehensive, well-implemented and enforced tobacco control strategies—particularly health warnings, mass media campaigns and smoke-free policies—to address knowledge gaps and accelerate progress towards global NCD prevention targets.

## Supplementary Material

Reviewer comments

Author's manuscript

## Data Availability

Data are available in a public open-access repository. Data are available upon reasonable request. All data relevant to the study are included in the article or uploaded as supplementary information. Data for 26 countries of the International Tobacco Control (ITC) Project (2002-2022) are available upon reasonable request. Data for 32 countries of the Global Adult Tobacco Survey (GATS) (2008-2020) are available in a public open-access repository. All data relevant to the study are included in the article or uploaded as supplementary information.

## References

[1] Murray CJL, Aravkin AY, Zheng P, et al. Global burden of 87 risk factors in 204 countries and territories, 1990–2019: a systematic analysis for the Global Burden of Disease Study 2019. The Lancet 2020;396:1223–49. 10.1016/S0140-6736(20)30752-2 PMC7566194 33069327

[2] World Health Organization. WHO Framework Convention on Tobacco Control. Geneva: World Health Organization. 2003.

[3] World Health Organization.. MPOWER: A policy package to reverse the tobacco epidemic. World Health Organization: Geneva. 2008.

[4] Hammond D. Health warning messages on tobacco products: a review. Tob Control 2011;20:327–37. 10.1136/tc.2010.037630 21606180

[5] World Health Organization. Tackling NCDS: ‘best buys’ and other recommended interventions for prevention and control of noncommunicable diseases. Geneva: World Health Organization. 2017.

[6] Carson-Chahhoud KV, Ameer F, Sayehmiri K, et al. Mass media interventions for preventing smoking in young people. Cochrane Database Syst Rev 2010;2017:CD001006. 10.1002/14651858.CD001006.pub3 PMC6481357 28574573

[7] Evans KA, Sims M, Judge K, et al. Assessing the knowledge of the potential harm to others caused by second-hand smoke and its impact on protective behaviours at home. J Public Health (Bangkok) 2012;34:183–94. 10.1093/pubmed/fdr104 PMC3355753 22201034

[8] Yang J, Hammond D, Driezen P, et al. Health knowledge and perception of risks among Chinese smokers and non-smokers: findings from the Wave 1 ITC China Survey. Tob Control 2010;19 Suppl 2:i18–23. 10.1136/tc.2009.029710 20935195 PMC5654747

[9] Demissie HS, Smith T, de Quevedo IG, et al. Factors associated with quit attempt and successful quitting among adults who smoke tobacco in Ethiopia: Global Adult Tobacco Survey (GATS) 2016. Tob Prev Cessat 2022;8:12. 10.18332/tpc/146170 35350770 PMC8915294

[10] Hyland A, Li Q, Bauer JE, et al. Predictors of cessation in a cohort of current and former smokers followed over 13 years. Nicotine Tob Res 2004;6 Suppl 3:S363–9. 10.1080/14622200412331320761 15799599

[11] Chiosi JJ, Andes L, Asma S, et al. Warning about the harms of tobacco use in 22 countries: findings from a cross-sectional household survey. Tob Control 2016;25:393–401. 10.1136/tobaccocontrol-2014-052047 25953532

[12] Siahpush M, McNeill A, Hammond D, et al. Socioeconomic and country variations in knowledge of health risks of tobacco smoking and toxic constituents of smoke: results from the 2002 International Tobacco Control (ITC) Four Country Survey. Tob Control 2006;15 Suppl 3:iii65–70. 10.1136/tc.2005.013276 16754949 PMC2593062

[13] Mills SD, Wiesen CA. Beliefs about the health effects of smoking among adults in the United States. Health Educ Behav 2022;49:497–505. 10.1177/10901981211004136 33870757 PMC9150142

[14] Trofor AC, Papadakis S, Lotrean LM, et al. Knowledge of the health risks of smoking and impact of cigarette warning labels among tobacco users in six European countries: findings from the EUREST-PLUS ITC Europe Surveys. Tob Induc Dis 2018;16:A10. 10.18332/tid/99542 31516464 PMC6661855

[15] Ahluwalia IB, Smith T, Arrazola RA, et al. Current tobacco smoking, quit attempts, and knowledge about smoking risks among persons aged ≥15 years - Global Adult Tobacco Survey, 28 countries, 2008-2016. MMWR 2018;67:1072–6. 10.15585/mmwr.mm6738a7 30260941 PMC6188126

[16] Rydz E, Telfer J, Quinn EK, et al. Canadians’ knowledge of cancer risk factors and belief in cancer myths. BMC Public Health 2024;24:329. 10.1186/s12889-024-17832-3 38291409 PMC10829248

[17] Shahab L, McGowan JA, Waller J, et al. Prevalence of beliefs about actual and mythical causes of cancer and their association with socio-demographic and health-related characteristics: findings from a cross-sectional survey in England. Eur J Cancer 2018;103:308–16. 10.1016/j.ejca.2018.03.029 29705530 PMC6202672

[18] Sun Y, Wang D, Yi Y, et al. Outdoor secondhand smoke exposure in public places frequented by minors in the urban area of Hangzhou City, China: a cross-sectional study. Tob Induc Dis 2024;22. 10.18332/tid/192129 PMC11375629 39239104

[19] Abdul Mutalib RNS, Abd Rani NL, Zulkifli A, et al. Knowledge, beliefs, and behaviors related to secondhand smoke and smoking in the home: a qualitative study with men in Malaysia. Nicotine Tob Res 2023;25:821–7. 10.1093/ntr/ntac239 36239239 PMC10032199

[20] Ezekiel E, Tomini S, Odukoya O. Secondhand smoke exposure, perceived risks and knowledge of the national tobacco law among non-smoking adults in outdoor motor parks in an urban area, Lagos, Nigeria. Tob Induc Dis 2018;16:A491. 10.18332/tid/84641

[21] Tattan-Birch H, Jarvis MJ. Children’s exposure to second-hand smoke 10 years on from smoke-free legislation in England: Cotinine data from the Health Survey for England 1998-2018. Lancet Reg Health Eur 2022;15:100315. 10.1016/j.lanepe.2022.100315 35146477 PMC8819129

[22] Nanninga S, Lhachimi SK, Bolte G. Impact of public smoking bans on children’s exposure to tobacco smoke at home: a systematic review and meta-analysis. BMC Public Health 2018;18:749. 10.1186/s12889-018-5679-z 29925343 PMC6011268

[23] Lim CCW, Rutherford B, Gartner C, et al. A systematic review of second-hand smoking mass media campaigns (2002-2022). BMC Public Health 2024;24:693. 10.1186/s12889-024-18222-5 38438990 PMC10913644

[24] Chido-Amajuoyi OG, Osaghae I, Agaku IT, et al. Exposure to school-based tobacco prevention interventions in low-income and middle-income countries and its association with psychosocial predictors of smoking among adolescents: a pooled cross-sectional analysis of Global Youth Tobacco Survey data from 38 countries. BMJ Open 2024;14:e070749. 10.1136/bmjopen-2022-070749 PMC10900417 38413149

[25] Lonergan BJ, Meaney S, Perry IJ, et al. Smokers still underestimate the risks posed by secondhand smoke: a repeated cross-sectional study. Nicotine Tob Res 2014;16:1121–8. 10.1093/ntr/ntu046 24867880

[26] Thompson ME, Driezen P, Boudreau C, et al. Methods of the International Tobacco Control (ITC) EUREST-PLUS ITC Europe Surveys. Eur J Public Health 2020;30:iii4–9. 10.1093/eurpub/ckz212 32053183 PMC7526778

[27] ITC Project. ITC Project. Methods and Technical Reports. 2024. Available: https://itcproject.org/methods/technical-reports/

[28] Global Tobacco Surveillance System. GTSS Academy. Global Tobacco Surveillance System Academy, 2026. Available: https://www.gtssacademy.org/

[29] International Tobacco Control Policy Evaluation Project. Data request forms. 2026. Available: https://itcproject.org/request-data-form/

[30] Roth GA, Mensah GA, Johnson CO, et al. Global burden of cardiovascular diseases and risk factors, 1990-2019: update from the GBD 2019 Study. J Am College Cardiology 2020;76:2982–3021. 10.1016/j.jacc.2020.11.010 PMC7755038 33309175

[31] National Cancer Institute. Health effects of exposure to environmental tobacco smoke. Smoking and tobacco control monograph 10. Bethesda, MD: National Cancer Institute, 1999.

[32] Barnoya J, Glantz SA. Cardiovascular effects of secondhand smoke: nearly as large as smoking. Circulation 2005;111:2684–98. 10.1161/CIRCULATIONAHA.104.492215 15911719

[33] Institute for Health Metrics and Evaluation. GBD summaries. secondhand smoke - level 3 risk. 2019. Available: https://www.healthdata.org/results/gbd_summaries/2019/secondhand-smoke-level-3-risk

[34] Flor LS, Anderson JA, Ahmad N, et al. Health effects associated with exposure to secondhand smoke: a Burden of Proof study. Nat Med 2024;30:149–67. 10.1038/s41591-023-02743-4 38195750 PMC10803272

[35] Global Burden of Disease Collaborative Network. Global burden of disease study 2019 (GBD 2019) results. 2020. Available: https://vizhub.healthdata.org/gbd-results/

[36] Reitsma MB, Kendrick PJ, Ababneh E, et al. Spatial, temporal, and demographic patterns in prevalence of smoking tobacco use and attributable disease burden in 204 countries and territories, 1990–2019: a systematic analysis from the Global Burden of Disease Study 2019. The Lancet 2021;397:2337–60. 10.1016/S0140-6736(21)01169-7 PMC8223261 34051883

[37] Öberg M, Jaakkola MS, Woodward A, et al. Worldwide burden of disease from exposure to second-hand smoke: a retrospective analysis of data from 192 countries. The Lancet 2011;377:139–46. 10.1016/S0140-6736(10)61388-8 21112082

[38] Brauer M, Roth GA, Aravkin AY, et al. Global burden and strength of evidence for 88 risk factors in 204 countries and 811 subnational locations, 1990–2021: a systematic analysis for the Global Burden of Disease Study 2021. The Lancet 2024;403:2162–203. 10.1016/S0140-6736(24)00933-4 PMC11120204 38762324

[39] World Health Organization. Tobacco control (TFI). 2021.

[40] Noar SM, Francis DB, Bridges C, et al. The impact of strengthening cigarette pack warnings: Systematic review of longitudinal observational studies. Soc Sci Med 2016;164:118–29. 10.1016/j.socscimed.2016.06.011 27423739 PMC5026824

[41] Ngo A, Cheng K-W, Shang C, et al. Global evidence on the association between cigarette graphic warning labels and cigarette smoking prevalence and consumption. Int J Environ Res Public Health 2018;15:421. 10.3390/ijerph15030421 29495581 PMC5876966

[42] Mantey D, Hunt E, Hoelscher D, et al. Social cognitive theory and health behaviors. 2024.

[43] World Health Organization. WHO report on the global tobacco epidemic, 2013: enforcing bans on tobacco advertising, promotion and sponsorship. Geneva World Health Organization; 2013.

[44] World Health Organization. WHO report on the global tobacco epidemic, 2011: warning about the dangers of tobacco. Geneva: World Health Organization; 2011.

[45] World Health Organization. WHO report on the global tobacco epidemic, 2023: protect people from tobacco smoke. Geneva: World Health Organization; 2023.

[46] World Health Organization. WHO global report on trends in prevalence of tobacco use 2000–2030. Geneva: World Health Organization; 2024.

[47] World Health Organization. WHO Framework Convention on Tobacco Control. Parties. 2024. Available: https://fctc.who.int/who-fctc/overview/parties

[48] World Health Organization. WHO report on the global tobacco epidemic, 2025. Geneva: World Health Organization; 2025.

[49] Byron MJ, Cohen JE, Frattaroli S, et al. Implementing smoke-free policies in low- and middle-income countries: a brief review and research agenda. Tob Induc Dis 2019;17:60. 10.18332/tid/110007 31582949 PMC6770618

[50] Daba C, Atamo A, Gasheya KA, et al. Non-compliance with smoke-free law in public places: a systematic review and meta-analysis of global studies. Front Public Health 2024;12:1354980. 10.3389/fpubh.2024.1354980 38694973 PMC11061889

[51] Gilmore AB, Callard C, Sy D, et al. 20th anniversary of the WHO Framework Convention on Tobacco Control coming into force: time for a step change in ambition. The Lancet 2025;405:677–81. 10.1016/S0140-6736(25)00336-8 PMC7617489 40020702

[52] Hill AB. The environment and disease: association or causation? Proc R Soc Med 1965;58:295–300. 10.1177/003591576505800503 14283879 PMC1898525

[53] Campbell DT, Fiske DW. Convergent and discriminant validation by the multitrait-multimethod matrix. Psychol Bull 1959;56:81–105. 10.1037/h0046016 13634291

[54] United Nations. Transforming our world: the 2030 agenda for sustainable development. 2015.

[55] Chung-Hall J, Craig L, Gravely S, et al. Impact of the WHO FCTC over the first decade: a global evidence review prepared for the Impact Assessment Expert Group. Tob Control 2019;28:s119–28. 10.1136/tobaccocontrol-2018-054389 29880598 PMC6589489

[56] Hoffman SJ, Tan C. Overview of systematic reviews on the health-related effects of government tobacco control policies. BMC Public Health 2015;15:744. 10.1186/s12889-015-2041-6 26242915 PMC4526291

