# Supplementary material for: Changes in knowledge of the major harms of tobacco smoking and secondhand smoke exposure among adults who smoke: findings from 26 countries of the International Tobacco Control (ITC) Project (2002–2022) and 32 countries of the Global Adult Tobacco Survey (GATS) (2008–2020)
Source: BMJ Open. 2026 Aug 4;16(8):e107667. doi: 10.1136/bmjopen-2025-107667 (PMC13479502; doi:10.1136/bmjopen-2025-107667)
Supplement: Supplementary file 1 [file bmjopen-16-8-s001.pdf]

Supplementary Appendix

Supplementary table 1. International Tobacco Control Policy Evaluation Project (ITC) Survey methods in 26 countries included in analysis, 2002-2022.

Country	Wave	Year	Data Collection Mode	Sample Representativeness	Sampling Design	Adults who Smoke† (N)	Replenishment Response Rate	Replenishment Cooperation Rate
Australia	1	2002	Telephone	National (telephone: random digit dialing)	Stratified random single stage	2194	45.8%	78.8%
	2	2003				1893	36.7%	79.3%
	3	2004				1757	44.2%	79.2%
	4	2005-2006				1602	39.5%	79.9%
	5	2006-2007				1656	45.3%	84.9%
	6	2007-2008				1604	29.1%	79.6%
	7	2008-2009	Telephone, Web	National (telephone; random digit dialing), Probability web panel	Stratified random single stage	1182	30.2%	80.7%
	8	2010-2011				893	28.0%	81.2%
	8.5	2011-2012				848	17.5%	96.6%
	9	2013				792	13.2%	98.3%
	V1	2016	Web	National probability web panel	Stratified single stage	1304	32.7%	93.1%
	V2	2018				1244	22.2%	95.1%
	V3	2020				1219	35.5%	93.4%
Bangladesh	1	2009	Face-to-Face	National	Stratified multi-stage	3041	> 90% household enumeration rate	81.1% individual response rate
	2	2010				2931	No new enumeration	Not computed (a)
	3	2011-2012				2493	> 90% household enumeration rate	Not reported
	4	2014-2015				1880	No new enumeration	Not computed (a)
Brazil	1	2009	Telephone	3 cities: Rio de Janeiro, São Paulo, Porto Alegre	Stratified single stage	1215	10.5%	90.7%
	2	2012-2013				1097	21.6%	98.9%
	3	2016-2017				1216	35.5%	60.4%
Canada	1	2002	Telephone	National (telephone: random digit dialing)	Stratified single stage	2070	49.5%	82.3%
	2	2003				1872	35.3%	86.6%
	3	2004				1735	50.0%	78.8%
	4	2005-2006				1590	29.4%	79.4%
	5	2006-2007				1514	27.3%	75.1%
	6	2007-2008				1413	26.7%	79.9%

Chung-Hall J, *et al. BMJ Open* 2026; 16:e107667. doi: 10.1136/bmjopen-2025-107667

Country	Wave	Year	Data Collection Mode	Sample Representativeness	Sampling Design	Adults who Smoke[†] (N)	Replenishment Response Rate	Replenishment Cooperation Rate
	7	2008-2009	Telephone, Web	National (telephone: random digit dialing), National probability web panel	Stratified single stage	1185	29.0%	81.1%
	8	2010-2011				904	29.9%	71.4%
	9	2013-2014				720	13.8%	66.5%
	V1	2016	Web	National probability web panel	Stratified single stage	2820	19.1%	91.1%
	V2	2018				2822	25.6%	94.8%
	V3	2020				2843	14.6%	93.2%
China	1	2006	Face-to-Face	6 cities: Beijing, Changsha, Guangzhou, Shanghai, Shenyang, Yinchuan	Stratified multi-stage	4732	Beijing:50.0%<br>Changsha:50.0%<br>Guangzhou:66.0%<br>Shanghai:61.3%<br>Shenyang:50.0%<br>Yinchuan:39.4%	Not computed (c)
	2	2007-2008				4623	Not computed (a)	Not computed (a)
	3	2009		7 cities: Beijing, Changsha, Guangzhou, Shanghai, Shenyang, Yinchuan, Kunming		5210	Beijing:Not computed (c)<br>Changsha:Not computed (c)<br>Guangzhou:85.0%<br>Shanghai:Not computed (c)<br>Shenyang:Not computed (c)<br>Yinchuan:Not computed (c)<br>Kunming:23.0%	Not computed (c)
	4	2010-2011				5077	Beijing:92.2%<br>Changsha:Not computed (c)<br>Guangzhou:85.0%<br>Shanghai:Not computed (c)<br>Shenyang:Not computed (c)<br>Yinchuan:Not computed (c)<br>Kunming:Not computed (c)	Not computed (c)
	5	2013-2015		5 cities: Beijing, Shanghai, Guangzhou, Shenyang, Kunming<br>5 rural areas: Xining, Tongren, Huzhou, Changzhi, Yichun		7387	Not computed (c)	Not computed (c)

Country	Wave	Year	Data Collection Mode	Sample Representativeness	Sampling Design	Adults who Smoke† (N)	Replenishment Response Rate	Replenishment Cooperation Rate
England	V1	2016	Web	Customized web panel	Stratified single stage	3536	15.2%	92.6%
	V2	2018				4149	44.1%	82.3%
	V3	2020				3233	13.7%	81.2%
France	1	2006-2007	Telephone	National (telephone: random digit dialing)	Stratified single stage	1735	36.3%	75.3%
	2	2008				1540	36.1%	80.5%
	3	2012				1420	21.3%	78.8%
	A1	2019	Web	National: customized web panel	Stratified single stage	1679	9.7%	92.5%
Germany	1	2007	Telephone	National	Stratified single stage	1515	26.1%	94.9%
	2	2009				912	Not computed (a)	Not computed (a)
	3	2011				515	Not computed (a)	Not computed (a)
	E1	2016	Face-to-face		Stratified multi-stage	1003	70.4%	83.4%
	E2	2018				946	68.7%	80.6%
Greece	E1	2016	Face-to-face	National	Stratified multi-stage	1000	89.6%	91.7%
	E2	2018				949	92.6%	93.3%
Hungary	E1	2016	Face-to-face	National	Stratified multi-stage	1000	92.8%	94.0%
	E2	2018				949	90.4%	91.6%
India**	1	2010-2011	Face-to-face	4 states: Bihar, Maharashtra, Madhya Pradesh, West Bengal	Stratified multi-stage	2042	Bihar:97.1%<br>Maharashtra:93.2%<br>Madhya Pradesh:93.0%<br>West Bengal:87.1%	Bihar:98.8%<br>Maharashtra:96.0%<br>Madhya Pradesh: 94.1%<br>West Bengal:93.0%
	2	2012-2013				1784	Bihar:97.7%<br>Maharashtra:82.3%<br>Madhya Pradesh:97.5%<br>West Bengal:82.3%	Bihar:100.0%<br>Maharashtra:90.9%<br>Madhya Pradesh:99.5%<br>West Bengal:90.7%
	3	2018-2019				1472	Bihar:65.1%<br>Maharashtra:81.5%<br>Madhya Pradesh:82.2%<br>West Bengal:75.0%	Bihar:100.0%<br>Maharashtra:93.8%<br>Madhya Pradesh: 99.5%<br>West Bengal: 100.0%
Japan	1	2018	Web	National probability web panel	Stratified single stage	3837	46.7%	96.8%
	2	2019				2689	27.2%	96.9%
	3	2020				2793	23.1%	94.8%
	4	2021				2649	27.4%	94.5%

Chung-Hall J, *et al. BMJ Open* 2026; 16:e107667. doi: 10.1136/bmjopen-2025-107667

Country	Wave	Year	Data Collection Mode	Sample Representativeness	Sampling Design	Adults who Smoke† (N)	Replenishment Response Rate	Replenishment Cooperation Rate
Kenya	1	2012	Face-to-face	National	Stratified multi-stage	1049	82.3%	Not computed (c)
	2	2018				833	Not computed (c)	Not computed (c)
Malaysia	1	2005	Face-to-face	6 states: Kedah, Selangor, Johore, Terengganu, Sabah, Sarawak	Stratified multi-stage	1974	Not computed (c)	Not computed (c)
	2	2006-2007				1478	Not computed (c)	Not computed (c)
	3	2008	Face-to-face, Telephone	7 states: Kedah, Penang, Selangor, Johore, Terengganu, Sabah, Sarawak	Stratified multi-stage	1809	Not computed (c)	Not computed (c)
	4	2009				1872	Not computed (c)	Not computed (c)
	5	2011-2012	Telephone	7 states: Kedah, Penang, Selangor, Johore, Terengganu, Sabah, Sarawak	Stratified multi-stage and single stage	1764	35.6%	55.9%
	6	2013-2014				1694	46.1%	85.5%
	S1	2020	Web	National population having internet access	Stratified single stage	1047	11.3%	Not computed (c)
Mauritius	1	2009	Face-to-face	National	Stratified multi-stage	598	80.7%	89.8%
	2	2010				553	96.9%	100.0%
	3	2011				535	96.0%	100.0%
Mexico	1	2006	Face-to-face	4 cities: Ciudad Juárez, Guadalajara, Ciudad de México, Tijuana	Stratified multi-stage	1079	Not computed (c)	88.7%
	2	2007		4 cities: Ciudad Juárez, Guadalajara, Ciudad de México, Tijuana		941	47.2%	Not computed (c)
	3	2008		7 cities: Ciudad Juárez, Guadalajara, Ciudad de México, Tijuana, Monterrey, Puebla, Mérida		1861	41.9%	76.1%
	4	2010		7 cities: Guadalajara, Ciudad de México, Tijuana, Leon, Monterrey, Puebla, Mérida		1853	41.5%	87.4%
	5	2011		7 cities: Guadalajara, Ciudad de México, Tijuana, Leon, Monterrey, Puebla, Mérida		1764	47.2%	Not computed (c)
	6	2012		7 cities: Guadalajara, Ciudad de México, Tijuana, Leon, Monterrey, Puebla, Mérida		1747	Not computed (c)	93.8%
	7	2014-2015		3 cities: Ciudad de México, Guadalajara, Monterrey		778	Not computed (c)	Not computed (c)

Country	Wave	Year	Data Collection Mode	Sample Representativeness	Sampling Design	Adults who Smoke[†] (N)	Replenishment Response Rate	Replenishment Cooperation Rate
Netherlands	1	2008	Telephone, Web	National (telephone: random digit dialing), Probability web panel	Stratified single stage	2072	4.2%	78.1%
	2		Web				83.0%*	Not computed (c)
	3		Telephone, Web				80.0%*	Not computed (c)
	4		Web				70.0%*	Not computed (c)
	5	2011	Web	National probability web panel (TNS NIPObase)	Stratified single stage	1672	65.0% *	Not computed (c)
	6	2012				1551	79.0% *	Not computed (c)
	7	2013				1480	79.0% *	Not computed (c)
	8	2014				1516	70.0% *	Not computed (c)
	9	2015				1217	66.0% *	Not computed (c)
	10	2016				1213	67.0% *	Not computed (c)
	11	2017				1199	69.0% *	Not computed (c)
	D1	2020	Web	National probability web panel (TNS NIPObase)	Stratified single stage	2067	57.6%	96.1%
	D2	2020				1752	58.3%	96.2%
	D3	2021				1721	54.0%	93.9%
	D4	2022				1734	55.4%	95.2%
New Zealand	1	2007-2008	Telephone	National	Sampled from New Zealand Health Survey national sample	1236	32.6%	56.4%
	2	2008-2009				761	Not computed (a)	Not computed (a)
	L1	2016-2017				910	27.6%	53.3%
	L2	2018				726	19.7%	Not computed (c)
	L3	2020-2021	Web	National, customized web panel, targeted social media	Sampled from New Zealand Health Survey national sample, web panel, social media	992	Not computed (b)	62.5%
Poland	1	2016	Face-to-face	National	Stratified multi-stage	1006	79.5%	81.6%
	2	2018				948	86.5%	87.6%
Republic of Korea	1	2005	Telephone	National	Stratified single stage	995	14.5%	71.2%
	2	2008				1737	2.4%	79.4%
	3	2010				1560	8.8%	95.3%
	R1	2016				2000	11.6%	99.8%
	A1	2020	Web	Customized web panel	Stratified single stage	3840	15.2%	97.4%
	A2	2021				3641	16.4%	97.0%

Chung-Hall J, *et al. BMJ Open* 2026; 16:e107667. doi: 10.1136/bmjopen-2025-107667

Country	Wave	Year	Data Collection Mode	Sample Representativeness	Sampling Design	Adults who Smoke† (N)	Replenishment Response Rate	Replenishment Cooperation Rate
Romania	1	2016	Face-to-face	National	Stratified multi-stage	1001	80.0%	81.2%
	2	2018				918	83.4%	84.8%
Spain	1	2016	Face-to-face	National	Stratified multi-stage	1001	81.1%	82.0%
	2	2018				886	83.1%	84.0%
	2.5	2021				864	80.8	96.2
Thailand	1	2005	Face-to-face	4 regions: Northern Region, North-Eastern Region, Central Plain Region, Southern Region	Stratified multi-stage	2000	Not computed (c)	Household cooperation rate 93.6%
	2	2006				1866	Not computed (c)	Not computed (c)
	3	2008				2163	Not computed (c)	Not computed (c)
	4	2009				1907	Not computed (c)	Not computed (c)
	5	2011				1687	Not computed (c)	Not computed (c)
	6	2012				1670	Not computed (c)	Not computed (c)
United States	1	2002	Telephone	National (telephone: random digit dialing)	Stratified single stage	2065	25.6%	83.2%
	2	2003				1837	Not computed (c)	88.6%
	3	2004				1846	34.9%	74.1%
	4	2005-2006				1698	23.4%	74.9%
	5	2006-2007				1668	20.7%	71.9%
	6	2007-2008				1570	22.3%	73.9%
	7	2008-2009	Telephone, Web	National (telephone: random digit dialing), probability web panel	Stratified single stage	1331	29.7%	90.8%
	8	2010-2011				1456	31.6%	90.1%
	9	2013-2015				1456	58.6% (web panel part)	
	V1	2016	Web	National probability web panel	Stratified single stage	2197	60.1%	80.4%
	V2	2018				2066	37.7%	75.2%
	V3	2020				1817	49.3%	86.7%
United Kingdom	1	2002	Telephone	National (telephone: random digit dialing)	Stratified single stage	2306	37.8%	78.7%
	2	2003				1866	25.8%	73.9%

Country	Wave	Year	Data Collection Mode	Sample Representativeness	Sampling Design	Adults who Smoke† (N)	Replenishment Response Rate	Replenishment Cooperation Rate
	3	2004				1748	41.6%	71.6%
	4	2006				1631	21.4%	78.7%
	5	2007				1567	12.9%	87.3%
	6	2008				1492	15.4%	74.6%
	7	2009	Telephone, Web	National (telephone: random digit dialing), National probability web panel	Stratified single stage	1309	32.6%	82.1%
	8	2011				812	Not computed (c)	Not computed (c)
	9	2013				926	Not computed (c)	Not computed (c)
Uruguay	1	2006	Face-to-face	Montevideo City	Stratified multi-stage	887	61.0%	72.6%
	2	2008-2009		5 cities: Montevideo, Salto, Maldonado, Durazno, Rivera		1294	68.7%	78.2%
	3	2010-2011				1224	63.2%	72.4%
	4	2012				1200	Not computed (c)	Not computed (c)
	5	2014				1181	Not computed (c)	Not computed (c)
Zambia	1	2012	Face-to-face	National	Stratified multi-stage	1188	61.1%	93.4%
	2	2014				1045	Not computed (c)	Not computed (c)

† Adults who smoke are defined as individuals (aged 18 years and older) who currently smoke cigarettes at least monthly.

Not computed (a): Little or no replenishment conducted

Not computed (b): Invitation data not available from survey firm

Not computed (c): Incomplete information available from field staff

Supplementary table 2. Global Adult Tobacco Survey (GATS) methods in 32 countries included in analysis, 2008–2020.

Country	Wave	Year	Data Collection Mode	Sample Representativeness	Sampling Design	Adults who Smoke[†] (N)	Response Rate
Argentina	1	2012	Face-to-face interview	National	Stratified multi-stage	1610	74.3%
Bangladesh	1	2009	Face-to-face interview	National	Stratified multi-stage	2207	93.6%
	2	2017	Face-to-face interview	National	Stratified multi-stage	2470	90.8%
Botswana	1	2017	Face-to-face interview	National	Stratified multi-stage	628	80.0%
Brazil	1	2008	Face-to-face interview	National	Stratified multi-stage	6911	94.0%
Cameroon	1	2013	Face-to-face interview	National	Stratified multi-stage	399	94.1%
China	1	2010	Face-to-face interview	National	Stratified multi-stage	4002	96.0%
	2	2018	Face-to-face interview	National	Stratified multi-stage	4953	91.5%
Costa Rica	1	2015	Face-to-face interview	National	Stratified multi-stage	650	89.2%
Egypt	1	2009	Face-to-face interview	National	Stratified multi-stage	4124	97.2%
Ethiopia	1	2016	Face-to-face interview	National	Stratified multi-stage	695	93.4%
Greece	1	2013	Face-to-face interview	National	Stratified multi-stage	1662	69.6%
India	1	2009	Face-to-face interview	National	Stratified multi-stage	11538	91.8%
	2	2017	Face-to-face interview	National	Stratified multi-stage	9434	92.9%
Indonesia	1	2011	Face-to-face interview	National	Stratified multi-stage	2783	94.3%
Kazakhstan	1	2014	Face-to-face interview	National	Stratified multi-stage	1020	96.7%
Kenya	1	2014	Face-to-face interview	National	Stratified multi-stage	447	87.1%
Malaysia	1	2011	Face-to-face interview	National	Stratified multi-stage	965	85.3%
Mexico	1	2009	Face-to-face interview	National	Stratified multi-stage	1752	82.5%
	2	2015	Face-to-face interview	National	Stratified multi-stage	1809	82.7%
Nigeria	1	2012	Face-to-face interview	National	Stratified multi-stage	422	89.1%

Country	Wave	Year	Data Collection Mode	Sample Representativeness	Sampling Design	Adults who Smoke† (N)	Response Rate
Pakistan	1	2014	Face-to-face interview	National	Stratified multi-stage	953	81.0%
Panama	1	2013	Face-to-face interview	National	Stratified multi-stage	973	88.4%
Philippines	1	2009	Face-to-face interview	National	Stratified multi-stage	2698	94.7%
	2	2015	Face-to-face interview	National	Stratified multi-stage	2751	92.1%
Poland	1	2009	Face-to-face interview	National	Stratified multi-stage	2400	65.1%
Qatar	1	2013	Face-to-face interview	National	Stratified multi-stage	1070	98.5%
Romania	1	2011	Face-to-face interview	National	Stratified multi-stage	1044	88.5%
	2	2018	Face-to-face interview	National	Stratified multi-stage	1236	88.0%
Russian Federation	1	2009	Face-to-face interview	National	Stratified multi-stage	4747	97.7%
	2	2016	Face-to-face interview	National	Stratified multi-stage	3312	98.2%
Senegal	1	2015	Face-to-face interview	National	Stratified multi-stage	267	97.0%
Thailand	1	2009	Face-to-face interview	National	Stratified multi-stage	4860	94.2%
	2	2011	Face-to-face interview	National	Stratified multi-stage	4229	96.3%
Turkey	1	2008	Face-to-face interview	National	Stratified multi-stage	2666	90.9%
	2	2012	Face-to-face interview	National	Stratified multi-stage	2376	90.1%
	3	2016	Face-to-face interview	National	Stratified multi-stage	2792	82.2%
Uganda	1	2013	Face-to-face interview	National	Stratified multi-stage	568	86.6%
Ukraine	1	2010	Face-to-face interview	National	Stratified multi-stage	2361	76.1%
	2	2017	Face-to-face interview	National	Stratified multi-stage	1776	64.4%
United Republic of Tanzania	1	2018	Face-to-face interview	National	Stratified multi-stage	375	91.7%
Uruguay	1	2009	Face-to-face interview	National	Stratified multi-stage	1362	95.2%
	2	2017	Face-to-face interview	National	Stratified multi-stage	1043	89.4%

Country	Wave	Year	Data Collection Mode	Sample Representativeness	Sampling Design	Adults who Smoke[†] (N)	Response Rate
Viet Nam	1	2010	Face-to-face interview	National	Stratified multi-stage	2240	92.7%
	2	2015	Face-to-face interview	National	Stratified multi-stage	1957	95.8%

[†] Defined adults who smoke as persons (aged 18+ years) who currently smoked any combustible tobacco products (bidis, kreteks, cheroots, manufactured cigarettes, hand-rolled cigarettes, cigars, cigarillos, pipes, waterpipe with tobacco).

Supplementary table 3. International Tobacco Control Policy Evaluation Project (ITC) countries (2002–2022) and Global Adult Tobacco Survey (GATS) countries (2008–2020).

	**ITC**	**GATS**
Argentina	NA	X
Australia	X	NA
Bangladesh	X	X
Botswana	NA	X
Brazil	X	X
Cameroon	NA	X
Canada	X	NA
China	X	X
Costa Rica	NA	X
Egypt	NA	X
England	X	NA
Ethiopia	NA	X
France	X	NA
Germany	X	NA
Greece	X	X
Hungary	X	NA
India	X	X
Indonesia	NA	X
Japan	X	NA
Kazakhstan	NA	X
Kenya	X	X
Malaysia	X	X
Mauritius	X	NA
Mexico	X	X
Netherlands	X	NA
New Zealand	X	NA
Nigeria	NA	X
Pakistan	NA	X
Panama	NA	X
Philippines	NA	X
Poland	X	X
Qatar	NA	X
Republic of Korea	X	NA
Romania	X	X
Russian Federation	NA	X
Senegal	NA	X
Spain	X	NA
Thailand	X	X
Turkey	NA	X
Uganda	NA	X
Ukraine	NA	X
United Republic of Tanzania	NA	X
United States	X	NA
Uruguay	X	X
Viet Nam	NA	X
Zambia	X	NA

X=country included in ITC Project Surveys and/or GATS
NA=country not included in ITC Project Surveys and/or GATS

Chung-Hall J, *et al. BMJ Open* 2026; 16:e107667. doi: 10.1136/bmjopen-2025-107667

Supplementary table 4. International Tobacco Control Policy Evaluation Project (ITC) Survey and Global Adult Tobacco Survey (GATS) measures of knowledge about the risks of smoking and secondhand smoke exposure for lung cancer, heart disease/heart attack, and stroke.

Health knowledge variable	ITC Project Survey Question and Response Options	GATS Question and Response Options
Smoking causes lung cancer[a]	Based on what you know or believe, does smoking cause lung cancer in smokers? (question asked in 25/26 ITC countries)<br>☐ Yes<br>☐ No<br>☐ Refused<br>☐ Don't know	Based on what you know or believe, does smoking tobacco cause lung cancer? (question asked in 32 GATS countries)<br>☐ Yes<br>☐ No<br>☐ Refused<br>☐ Don't know
Smoking causes heart disease[b,c]	Based on what you know or believe, does smoking cause heart disease in smokers? (question asked in 25/26 ITC countries)<br>☐ Yes<br>☐ No<br>☐ Refused<br>☐ Don't know	Based on what you know or believe, does smoking tobacco cause heart attack? (question asked in 32 GATS countries)<br>☐ Yes<br>☐ No<br>☐ Refused<br>☐ Don't know
Smoking causes stroke	Based on what you know or believe, does smoking cause stroke in smokers? (question asked in 25/26 ITC countries)<br>☐ Yes<br>☐ No<br>☐ Refused<br>☐ Don't know	Based on what you know or believe, does smoking tobacco cause stroke (blood clots in the brain)? (question asked in 32 GATS countries<br>☐ Yes<br>☐ No<br>☐ Refused<br>☐ Don't know
Secondhand smoke causes lung cancer[d]	Based on what you know or believe, does smoking cause lung cancer in non-smokers from secondhand smoke? (question asked in 26 ITC countries)<br>☐ Yes<br>☐ No<br>☐ Refused<br>☐ Don't know	Based on what you know or believe does breathing other people's smoke cause lung cancer? (question asked in 18/32 GATS countries)<br>☐ Yes<br>☐ No<br>☐ Refused<br>☐ Don't know
Secondhand smoke causes heart attack[e]	Based on what you know or believe, does secondhand smoke cause heart attack in non-smokers? (question asked in 22/26 ITC countries)<br>☐ Yes<br>☐ No<br>☐ Refused<br>☐ Don't know	Based on what you know or believe, does breathing in other people's smoke cause heart disease? (question asked in 18/32 GATS countries)<br>☐ Yes<br>☐ No<br>☐ Refused<br>☐ Don't know

[a] ITC Bangladesh Wave 1 (2009), Wave 2 (2010), Wave 3 (2011-12), Wave 4 (2014-15) Survey: Based on what you know or believe, does smoking cigarettes cause lung cancer in smokers?
ITC France Wave 1 (2006-07), Wave 2 (2008), and Wave 3 (2012) Survey: Based on what you know or believe, smoking can cause (Wave 3)/can smoking cause (Wave 1, Wave 2) lung cancer?
ITC Germany Wave 1 (2016) and Wave 2 (2018) Survey: Based on what you know or believe, does smoking cause lung cancer?
ITC India Wave 3 Survey: Based on what you know or believe, does smoking cigarettes cause lung cancer in smokers?
ITC Japan Wave 3 (2020) and Wave 4 (2021) Survey: Based on what you know or believe, does smoking cigarettes cause lung cancer in cigarette smokers?

ITC Republic of Korea Wave 1 (2005) and Wave 2 (2008) Survey: Based on what you know or believe, does smoking cigarettes cause lung cancer in cigarette smokers?
ITC Republic of Korea Wave 1 (2020) and Wave 2 (2021) Survey: Based on what you know or believe, does smoking cigarettes cause lung cancer in cigarette smokers?
ITC Netherlands Wave 5 (2011), Wave 6 (2012), Wave 7 (2013), Wave 8 (2014), Wave 9 (2015), Wave 10 (2016), Wave 11 (2017) Survey: Based on what you know or believe, does smoking cause lung cancer?
ITC Mauritius Wave 1 (2009) Survey: Based on what you know or believe, does smoking cigarettes cause lung cancer?
[b] ITC Japan Wave 1 (2018), Wave 2 (2019), and Wave 3 (2020) Survey: Based on what you know or believe, does smoking cigarettes cause myocardial infarction in cigarette smokers?
[c] ITC Bangladesh Wave 1 (2009) and Wave 2 (2010) Survey: Based on what you know or believe, does smoking cigarettes cause coronary heart disease/COPD?
[d] ITC Australia Wave 3 (2020) Survey: Based on what you know or believe, does secondhand smoke cause lung disease, including lung cancer, in non-smokers?
ITC Bangladesh Wave 1 (2009) Survey: Based on what you know or believe, does smoking cigarettes cause lung cancer in non-smokers from secondhand smoke?
ITC Canada Wave 3 (2020) Survey: Based on what you know or believe, does secondhand smoke cause lung disease, including lung cancer, in non-smokers?
ITC China Wave 1 (2006), Wave 2 (2007-08), Wave 3 (2009), Wave 4 (2010-11), Wave 5 (2013-15) Survey: Do you think smoking causes lung cancer in non-smokers from secondhand smoke?
ITC England Wave 3 (2020) Survey: Based on what you know or believe, does secondhand smoke cause lung disease, including lung cancer, in non-smokers?
ITC France Wave 1 (2006-07), Wave 2 (2008), Wave 3 (2012) Survey: Based on what you know or believe, can passive smoking cause lung cancer in non-smokers?
ITC India Wave 3 (2018-19) Survey: Based on what you know or believe, does passive cigarette smoking cause lung cancer in non-smokers?
ITC Japan Wave 1 (2018), Wave 2 (2019), Wave 3 (2020) Survey: Based on what you know or believe, does smoking cigarettes cause lung cancer in non-smokers from secondhand smoke?
ITC Japan Wave 4 (2021) Survey: Based on what you know or believe, does secondhand cigarette smoke cause lung cancer in non-smokers?
ITC Republic of Korea Wave 1 (2020) and Wave 2 (2021) Survey: Based on what you know or believe, does secondhand cigarette smoke cause lung cancer in non-smokers?
ITC Spain 2.5 (2021) Survey: Based on what you know or believe, does breathing secondhand smoke cause lung cancer in non-smokers?
ITC United States Wave 3 (2020) Survey: Based on what you know or believe, does secondhand smoke cause lung disease, including lung cancer, in non-smokers?
[e] ITC Bangladesh Wave 2 (2010): Based on what you know or believe, does passive smoking cause coronary heart disease/COPD in non-smokers?

Chung-Hall J, *et al. BMJ Open* 2026; 16:e107667. doi: 10.1136/bmjopen-2025-107667

Supplementary table 5. Proportions of adults who smoke and had knowledge about health risks of smoking at most recent survey for which data was available in ITC countries and GATS countries.[a]

	**Smoking causes lung cancer**		**Smoking causes heart disease (ITC)/heart attack (GATS)**		**Smoking causes stroke**		**Secondhand smoke causes lung cancer**		**Secondhand smoke causes heart attack (ITC)/heart disease (GATS)**	
**Country**	**ITC % [95% CI] (year)**	**GATS % [95% CI] (year)**	**ITC % [95% CI] (year)**	**GATS % [95% CI] (year)**	**ITC % [95% CI] (year)**	**GATS % [95% CI] (year)**	**ITC % [95% CI] (year)**	**GATS % [95% CI] (year)**	**ITC % [95% CI] (year)**	**GATS % [95% CI] (year)**
Argentina	NA	98.4 [97.1-99.1] (2012)	NA	92.1 [88.7-94.5] (2012)	NA	72.8 [64.7-79.6] (2012)	NA	NA	NA	NA
Australia	94.0 [92.6-95.1] (2003)	NA	91.4 [89.8-92.8] (2004)	NA	81.3 [77.8-84.4] (2020)	NA	73.1 [70.1-75.9] (2008-09)	NA	38.4 [34.3-42.6] (2013)	NA
Bangladesh	98.2 [96.9-98.9] (2014-15)	97.1 [95.8-98.0] (2017)	94.9 [91.3-97.0] (2014-15)	93.5 [91.9-94.8] (2017)	93.9 [90.3-96.3] (2014-15)	94.1 [92.4-95.5] (2017)	95.2 [93.0-96.7] (2014-15)	95.4 [94.1-96.4] (2017)	88.5 [82.4-92.7] (2014-15)	93.5 [91.9-94.8] (2017)
Botswana	NA	92.6 [89.5-94.8] (2017)	NA	81.0 [76.6-84.7] (2017)	NA	74.0 [68.9-78.4] (2017)	NA	NA	NA	NA
Brazil	96.2 [94.8-97.2] (2016-17)	97.5 [97.1-98.0] (2008)	93.7 [91.8-95.2] (2016-17)	88.6 [87.5-89.5] (2008)	86.8 [84.2-89.1] (2016-2017)	76.7 [75.3-78.1] (2008)	81.1 [78.3-83.7] (2016-17)	NA	NA	NA
Canada	94.0 [92.7-95.1] (2003)	NA	92.3 [91.1-93.4] (2020)	NA	85.7 [84.0-87.2] (2020)	NA	76.9 [74.9-78.8] (2020)	NA	49.9 [47.6-52.2] (2020)	NA
Cameroon	NA	89.9 [85.5-93.1] (2013)	NA	68.6 [61.2-75.1] (2013)	NA	61.3 [54.5-67.7] (2013)	NA	78.8 [73.3-83.4] (2013)	NA	72.9 [65.5-79.2] (2013)
China	81.1 [78.3-83.7] (2013-15)	78.4 [76.1-80.5] (2018)	57.7 [54.6-60.6] (2013-15)	46.4 [43.5-49.3] (2018)	37.3 [34.8-39.7] (2013-15)	37.4 [34.7-40.2] (2018)	72.7 [69.2-76.0] (2013-15)	61.6 [59.0-64.2] (2018)	53.8 [50.7-56.9] (2013-15)	37.7 [35.1-40.4] (2018)
Costa Rica	NA	96.9 [95.0-98.0] (2015)	NA	93.3 [91.0-95.1] (2015)	NA	72.0 [66.9-76.6] (2015)	NA	93.5 [90.8-95.5] (2015)	NA	88.2 [84.8-90.9] (2015)
Egypt	NA	97.4 [96.2-98.2] (2009)	NA	96.9 [96.0-97.6] (2009)	NA	89.8 [88.2-91.2] (2009)	NA	94.0 [92.7-95.1] (2009)	NA	91.8 [90.3-93.1] (2009)
England/ United Kingdom	91.5 [89.9-92.9] (2003)[b]	NA	86.0 [84.0-87.7] (2020)[c]	NA	78.5 [76.1-80.6] (2020)[c]	NA	66.7 [64.1-69.2] (2020)[c]	NA	44.6 [42.0-47.3] (2020)[c]	NA
Ethiopia	NA	67.1 [48.2-81.7] (2016)	NA	54.6 [39.2-69.1] (2016)	NA	29.6 [19.2-42.8] (2016)	NA	NA	NA	NA
France (2019)	93.8 [92.1-95.2] (2019)	NA	91.2 [89.4-92.8] (2019)	NA	83.4 [81.0-85.6] (2019)	NA	77.0 [74.2-79.6] (2019)	NA	56.1 [52.9-59.2] (2019)	NA
Germany	91.2 [85.6-94.7] (2018)	NA	80.6 [73.9-85.9] (2018)	NA	79.2 [72.9-84.3] (2018)	NA	58.4 [51.5-64.9] (2018)	NA	46.4 [39.6-53.4] (2018)	NA
Greece	96.0 [93.1-97.8] (2018)	95.1 [92.7-96.8] (2013)	95.1 [90.9-97.4] (2018)	88.1 [83.5-91.5] (2013)	79.2 [74.2-83.5] (2018)	69.3 [61.8-75.9] (2013)	64.7 [58.7-70.3] (2018)	68.1 [61.1-74.4] (2013)	60.7 [53.8-67.1] (2018)	66.9 [59.8-73.4] (2013)
Hungary	83.2 [78.3-87.2] (2018)	NA	70.2 [64.4-75.5] (2018)	NA	65.3 [59.7-70.6] (2018)	NA	60.3 [54.1-66.2] (2018)	NA	49.4 [43.5-55.3] (2018)	NA
India	91.9 [87.8-94.8] (2018-19)	91.9 [90.9-92.8] (2016)	86.1 [82.0-89.4] (2018-19)	75.3 [73.6-77.0] (2016)	75.0 [68.7-80.3] (2018-19)	63.1 [61.1-65.1] (2016)	88.3 [84.5-91.2] (2018-19)	NA	86.5 [82.1-89.9] (2018-19)	NA
Indonesia	NA	78.8 [75.4-81.9] (2011)	NA	78.0 [74.2-81.3] (2011)	NA	40.4 [35.6-45.3] (2011)	NA	NA	NA	NA
Japan	80.5 [77.9-82.8] (2021)	NA	65.5 [62.7-68.1] (2021)	NA	NA	NA	69.0 [66.7-71.2] (2021)	NA	56.3 [53.5-59.0] (2021)	NA
Kazakhstan	NA	78.9 [75.1-82.3] (2014)	NA	54.8 [50.4-59.0] (2014)	NA	55.2 [50.8-59.5] (2014)	NA	NA	NA	NA
Kenya	90.9 [87.9-93.3] (2018)	85.6 [80.6-89.4] (2014)	79.8 [75.7-83.4] (2018)	60.0 [53.7-66.0] (2014)	67.3 [62.1-72.2] (2018)	45.7 [38.4-53.1] (2014)	74.6 [69.4-79.2] (2018)	78.6 [72.4-83.7] (2014)	63.3 [57.7-68.6] (2018)	67.4 [60.7-73.5] (2014)
Malaysia	87.8 [84.7-90.3] (2020)	88.7 [85.1-91.5] (2011)	82.8 [79.8-85.4] (2020)	84.8 [80.9-88.1] (2011)	66.3 [62.5-70.0] (2020)	75.4 [70.9-79.4] (2011)	73.3 [69.6-76.7] (2020)	81.2 [77.3-84.6] (2011)	62.7 [58.7-66.6] (2020)	77.4 [73.0-81.2] (2011)

Chung-Hall J, et al. BMJ Open 2026; 16:e107667. doi: 10.1136/bmjopen-2025-107667

	**Smoking causes lung cancer**		**Smoking causes heart disease (ITC)/heart attack (GATS)**		**Smoking causes stroke**		**Secondhand smoke causes lung cancer**		**Secondhand smoke causes heart attack (ITC)/heart disease (GATS)**	
**Country**	**ITC % [95% CI] (year)**	**GATS % [95% CI] (year)**	**ITC % [95% CI] (year)**	**GATS % [95% CI] (year)**	**ITC % [95% CI] (year)**	**GATS % [95% CI] (year)**	**ITC % [95% CI] (year)**	**GATS % [95% CI] (year)**	**ITC % [95% CI] (year)**	**GATS % [95% CI] (year)**
Mauritius	91.8 [87.1-94.9] (2011)	NA	85.6 [80.9-89.3] (2011)	NA	76.1 [70.4-81.0] (2011)	NA	85.7 [80.4-89.8] (2011)	NA	NA	NA
Mexico	96.9 [94.4-98.3] (2014-15)	98.0 [97.1-98.6] (2015)	81.8 [76.7-86.0] (2014-15)	87.5 [85.3-89.4] (2015)	75.6 [70.8-79.8] (2014-15)	68.1 [65.2-70.8] (2015)	91.7 [88.1-94.3] (2014-15)	NA	79.9 [75.0-84.0] (2014-15)	NA
Netherlands	92.2 [90.7-93.5] (2022)	NA	86.3 [84.3-88.0] (2022)	NA	71.0 [68.5-73.3] (2022)	NA	64.7 [62.2-67.2] (2022)	NA	53.8 [51.2-56.4] (2022)	NA
New Zealand	NA	NA	NA	NA	76.9 [73.0-80.4] (2020-21)	NA	62.3 [58.1-66.3] (2020-21)	NA	47.6 [43.5-51.8] (2020-21)	NA
Nigeria	NA	55.6 [48.4-62.6] (2012)	NA	64.3 [57.3-70.8] (2012)	NA	32.2 [25.5-39.8] (2012)	NA	41.9 [35.1-49.0] (2012)	NA	54.0 [46.6-61.2] (2012)
Pakistan	NA	83.6 [79.8-86.8] (2014)	NA	86.9 [83.7-89.6] (2014)	NA	54.6 [49.1-60.1] (2014)	NA	69.3 [64.6-73.5] (2014)	NA	73.1 [68.6-77.1] (2014)
Panama	NA	96.0 [94.1-97.3] (2013)	NA	84.0 [78.2-88.5] (2013)	NA	76.8 [70.0-82.4] (2013)	NA	NA	NA	NA
Philippines	NA	93.6 [92.2-94.8] (2015)	NA	82.4 [79.9-84.6] (2015)	NA	75.3 [72.3-78.2] (2015)	NA	NA	NA	NA
Poland	85.9 [81.1-89.7] (2018)	94.9 [93.6-95.9] (2009)	79.7 [74.2-84.2] (2018)	79.8 [77.5-82.0] (2009)	64.7 [59.4-69.7] (2018)	58.6 [55.7-61.5] (2009)	69.2 [61.4-76.1] (2018)	91.4 [89.7-92.9] (2009)	60.7 [53.1-67.9] (2018)	83.7 [81.4-85.8] (2009)
Qatar	NA	90.0 [86.4-92.7] (2013)	NA	88.7 [85.3-91.4] (2013)	NA	70.0 [64.9-74.6] (2013)	NA	NA	NA	NA
Republic of Korea	90.0 [87.9-91.8] (2021)	NA	66.4 [61.6-70.8] (2021)	NA	72.2 [67.6-76.4] (2021)	NA	75.8 [70.3-80.6] (2021)	NA	60.6 [55.9-65.1] (2021)	NA
Romania	87.2 [83.1-90.5] (2018)	87.5 [84.3-90.1] (2018)	87.7 [83.5-90.9] (2018)	72.4 [68.1-76.3] (2018)	77.5 [72.1-82.1] (2018)	73.2 [68.7-77.3] (2018)	78.6 [73.3-83.0] (2018)	NA	73.9 [68.5-78.6] (2018)	NA
Russian Federation	NA	87.8 [85.8-89.6] (2016)	NA	74.1 [71.2-76.8] (2016)	NA	72.0 [68.9-74.9] (2016)	NA	NA	NA	NA
Senegal	NA	88.4 [83.0-92.3] (2015)	NA	71.4 [64.4-77.5] (2015)	NA	66.1 [58.6-72.8] (2015)	NA	83.8 [77.9-88.4] (2015)	NA	85.9 [80.4-90.1] (2015)
Spain	94.8 [92.2-96.6] (2021)	NA	88.5 [85.1-91.2] (2021)	NA	65.6 [58.6-72.1] (2021)	NA	69.3 [63.7-74.4] (2021)	NA	56.2 [49.6-62.5] (2021)	NA
Thailand	94.1 [92.9-95.1] (2008)	96.3 [95.0-97.2] (2011)	71.6 [67.4-75.5] (2012)	74.9 [72.3-77.3] (2011)	83.7 [81.4-85.7] (2012)	78.7 [76.1-81.0] (2011)	96.8 [95.2-97.9] (2012)	96.4 [95.5-97.1] (2011)	81.9 [77.8-85.4] (2012)	68.5 [65.8-71.2] (2011)
Turkey	NA	95.3 [94.0-96.3] (2016)	NA	95.3 [94.1-96.3] (2016)	NA	87.7 [85.7-89.4] (2016)	NA	93.0 [91.5-94.3] (2016)	NA	92.8 [91.3-94.0] (2016)
Uganda	NA	82.1 [77.9-85.7] (2013)	NA	68.1 [62.7-73.0] (2013)	NA	36.4 [30.5-42.8] (2013)	NA	72.1 [66.6-77.1] (2013)	NA	66.0 [60.1-71.4] (2013)
Ukraine	NA	86.9 [83.7-89.5] (2017)	NA	77.9 [74.6-80.9] (2017)	NA	76.8 [73.5-79.7] (2017)	NA	69.4 [65.9-72.6] (2017)	NA	71.1 [67.8-74.1] (2017)
Uruguay	95.9 [93.9-97.2] (2014)	95.9 [94.0-97.2] (2017)	89.3 [86.2-91.8] (2014)	91.4 [89.2-93.2] (2017)	65.2 [61.6-68.7] (2014)	76.8 [73.9-79.6] (2017)	80.7 [77.4-83.6] (2014)	NA	69.3 [65.4-72.8] (2014)	NA
United Republic of Tanzania	NA	81.0 [76.0-85.1] (2018)	NA	57.6 [51.4-63.6] (2018)	NA	39.8 [33.5-46.4] (2018)	NA	67.0 [61.0-72.4] (2018)	NA	56.0 [50.0-61.9] (2018)
United States	93.0 [91.6-94.1] (2003)	NA	86.9 [84.7-88.9] (2020)	NA	77.5 [74.8-80.0] (2020)	NA	68.4 [65.5-71.2] (2020)	NA	44.6 [41.5-47.8] (2020)	NA
Viet Nam	NA	94.7 [93.1-96.0] (2015)	NA	68.6 [65.6-71.4] (2015)	NA	68.3 [65.5-71.0] (2015)	NA	85.1 [82.7-87.3] (2015)	NA	21.3 [19.0-23.9] (2015)

Chung-Hall J, *et al. BMJ Open* 2026; 16:e107667. doi: 10.1136/bmjopen-2025-107667

	**Smoking causes lung cancer**		**Smoking causes heart disease (ITC)/heart attack (GATS)**		**Smoking causes stroke**		**Secondhand smoke causes lung cancer**		**Secondhand smoke causes heart attack (ITC)/heart disease (GATS)**	
**Country**	**ITC % [95% CI] (year)**	**GATS % [95% CI] (year)**	**ITC % [95% CI] (year)**	**GATS % [95% CI] (year)**	**ITC % [95% CI] (year)**	**GATS % [95% CI] (year)**	**ITC % [95% CI] (year)**	**GATS % [95% CI] (year)**	**ITC % [95% CI] (year)**	**GATS % [95% CI] (year)**
Zambia	78.1 [72.6-82.8] (2014)	NA	73.0 [69.8-76.0] (2014)	NA	47.4 [43.4-51.4] (2014)	NA	68.2 [63.3-72.8] (2014)	NA	64.8 [60.2-69.1] (2014)	NA
**Overall [a]**	**92.2**	**91.9**	**84.7**	**80.0**	**75.0**	**66.1**	**77.3**	**82.1**	**61.8**	**72.5**

Abbreviations: CI, confidence interval; NA=no data collected as country was not included in ITC Project Surveys and/or GATS.
[a] Point estimates of the mean knowledge for each health effect across all ITC countries and all GATS countries were calculated using a multi-level model (two levels: individual and country) with random intercepts at the country level, with roughly equal weights for each country. These point estimates cannot be used to make inferences to the global population since the countries included in our analysis were not randomly selected from all countries in the world. As such, we report the estimated all-country means of knowledge for each health effect without the associated confidence intervals, which would refer to a hypothetical superpopulation of countries. The all-country mean estimates in this analysis therefore only serve as the central point of selected countries.
[b] Data collected for United Kingdom sample in 2003.
[c] Data collected for England sample in 2020.

Supplementary table 6. Overall knowledge at most recent survey and average changes in knowledge per year (percentage points (PP)/year) of five diseases caused by smoking and secondhand smoke, among adults who smoke in ITC and GATS countries.

	**Data Source**			
	**ITC**		**GATS**	
	**Overall knowledge at most recent survey (%)**	**Average change in knowledge per year**	**Overall knowledge at most recent survey (%)**	**Average change in knowledge per year**
Smoking causes lung cancer	92.2	– 0.7 PP	91.9	– 0.2 PP
Smoking causes heart disease (ITC) / heart attack (GATS)	84.7	– 0.3 PP	80.0	+ 0.5 PP
Smoking causes stroke	75.0	+ 1.6 PP	66.1	+ 0.5 PP
Secondhand smoke causes lung cancer	77.3	– 0.1 PP	82.1	0.0 PP
Secondhand smoke causes heart attack (ITC) / heart disease (GATS)	61.8	+ 0.2 PP	72.5	– 0.1 PP
Simple average of estimated net change in knowledge across the five health harms		+0.14 PP		+0.14 PP

Note: This table presents a descriptive summary of changes in knowledge across the five health effects in ITC and GATS countries that are not derived from statistical models. Testing for statistically significant changes in knowledge cannot be assessed.

Abbreviation: PP=percentage points

Supplementary table 7. Changes in knowledge that smoking causes lung cancer, heart disease, and stroke among adults who smoke in ITC countries.

ITC Country	Smoking causes lung cancer				Smoking causes heart disease				Smoking causes stroke			
	**First survey % [95% CI] (year)**	**Last survey % [95% CI] (year)**	**Average PP change per year**	**p-value**	**First survey % [95% CI] (year)**	**Last survey % [95% CI] (year)**	**Average PP change per year**	**p-value**	**First survey % [95% CI] (year)**	**Last survey % [95% CI] (year)**	**Average PP change per year**	**p-value**
Australia	94.1<br>[92.9,95.1]<br>(2002)	93.3<br>[91.8,94.6]<br>(2003)	-0.8	.890	89.2<br>[87.8,90.6]<br>(2002)	90.5<br>[88.7,91.9]<br>(2004)	+0.6	.080	80.9<br>[79.0,82.6]<br>(2002)	80.8<br>[77.3,83.9]<br>(2020)	0.0	.694
Bangladesh	94.5<br>[92.3,96.1]<br>(2009)	98.1<br>[96.6,98.9]<br>(2014-15)	+0.6	.000	88.1<br>[84.3,91.1]<br>(2009)	94.5<br>[90.9,96.7]<br>(2014-15)	+1.2	.000	93.1<br>[90.4,95.1]<br>(2009)	93.6<br>[89.8,96.1]<br>(2014-15)	+0.1	.100
Brazil	96.6<br>[95.3,97.6]<br>(2009)	96.0<br>[94.5,97.1]<br>(2016-17)	-0.1	.700	95.4<br>[94.0,96.5]<br>(2009)	93.4<br>[91.5,94.9]<br>(2016-17)	-0.3	.420	82.6<br>[80.1,84.9]<br>(2009)	86.6<br>[83.9,88.8]<br>(2016-17)	+0.5	.210
Canada	94.9<br>[93.8,95.8]<br>(2002)	93.4<br>[92.1,94.6]<br>(2003)	-1.5	.590	91.2<br>[89.8,92.4]<br>(2002)	92.1<br>[90.9,93.2]<br>(2020)	+0.1	.810	82.6<br>[80.7,84.3]<br>(2002)	85.6<br>[83.9,87.1]<br>(2020)	+0.2	.870
China	71.5<br>[69.2,73.7]<br>(2006)	80.9<br>[77.8,83.7]<br>(2013-15)	+1.2	.007	38.0<br>[35.5,40.5]<br>(2006)	57.0<br>[53.8,60.1]<br>(2013-15)	+2.2	.010	16.2<br>[14.5,18.1]<br>(2006)	36.5<br>[34.1,39.0]<br>(2013-15)	+2.5	.000
England/UK[a]	94.0<br>[92.9,95.0]<br>(2002)	90.5<br>[88.8,92.1]<br>(2003)	-3.5	.090	90.1<br>[88.8,91.4]<br>(2002)	85.1<br>[83.1,87.0]<br>(2020)	-0.3	.590	70.5<br>[68.3,72.5]<br>(2002)	78.1<br>[75.7,80.3]<br>(2020)	+0.4	.830
France	99.1<br>[98.4,99.5]<br>(2006-07)	93.1<br>[91.3,94.6]<br>(2019)	-0.5	.000	97.1<br>[96.0,97.9]<br>(2006-07)	90.3<br>[88.4,91.9]<br>(2019)	-0.5	.003	87.6<br>[85.7,89.3]<br>(2006-07)	83.0<br>[80.5,85.1]<br>(2019)	-0.4	.310
Germany	97.8<br>[96.8,98.5]<br>(2007)	91.5<br>[88.1,94.0]<br>(2018)	-0.6	.000	95.6<br>[94.3,96.6]<br>(2007)	80.1<br>[76.1,83.6]<br>(2018)	-1.4	.000	88.9<br>[86.8,90.7]<br>(2007)	78.3<br>[74.2,81.9]<br>(2018)	-1.0	.000
Greece	96.4<br>[94.6,97.7]<br>(2016)	95.9<br>[92.7,97.8]<br>(2018)	-0.3	.890	95.2<br>[92.7,96.9]<br>(2016)	94.9<br>[90.8,97.3]<br>(2018)	-0.2	.430	68.8<br>[63.4,73.7]<br>(2016)	79.8<br>[74.7,84.1]<br>(2018)	+5.5	.003
Hungary	84.4<br>[80.5,87.7]<br>(2016)	82.8<br>[77.8,86.9]<br>(2018)	-0.8	.320	68.9<br>[62.7,74.4]<br>(2016)	69.3<br>[63.5,74.6]<br>(2018)	+0.2	.830	56.0<br>[50.0,61.7]<br>(2016)	64.9<br>[59.2,70.1]<br>(2018)	+4.5	.037
India	89.2<br>[86.5,91.4]<br>(2010-11)	92.1<br>[87.7,94.9]<br>(2018-19)	+0.4	.130	75.5<br>[71.2,79.3]<br>(2010-11)	85.4<br>[80.9,89.0]<br>(2018-19)	+1.2	.010	62.9<br>[57.6,67.9]<br>(2010-11)	73.6<br>[67.1,79.3]<br>(2018-19)	+1.3	.006
Japan	86.2<br>[84.9,87.4]<br>(2018)	80.3<br>[77.5,82.9]<br>(2021)	-2.0	.007	77.1<br>[75.5,78.6]<br>(2018)	64.2<br>[61.3,67.1]<br>(2021)	-4.3	.002	NA	NA	NA	NA
Kenya	90.0<br>[86.8,92.5]<br>(2012)	91.1<br>[87.9,93.4]<br>(2018)	+0.2	.700	65.7<br>[58.8,72.1]<br>(2012)	78.9<br>[74.6,82.7]<br>(2018)	+2.2	.010	44.7<br>[37.9,51.8]<br>(2012)	68.2<br>[63.2,72.9]<br>(2018)	+3.9	.000
Malaysia	87.3<br>[83.3,90.5]<br>(2005)	87.3<br>[84.2,89.8]<br>(2020)	0.0	.880	92.0<br>[90.3,93.4]<br>(2009)	81.5<br>[78.4,84.2]<br>(2020)	-1.0	.080	70.4<br>[65.2,75.2]<br>(2005)	65.0<br>[61.2,68.6]<br>(2020)	-0.4	.610
Mauritius	94.0<br>[90.7,96.2]<br>(2009)	91.4<br>[87.1,94.4]<br>(2011)	-1.3	.740	90.3<br>[85.9,93.4]<br>(2009)	84.8<br>[80.0,88.6]<br>(2011)	-2.8	.400	66.0<br>[59.6,71.9]<br>(2009)	75.3<br>[69.5,80.3]<br>(2011)	+4.6	.008

ITC Country	Smoking causes lung cancer				Smoking causes heart disease				Smoking causes stroke			
	**First survey % [95% CI] (year)**	**Last survey % [95% CI] (year)**	**Average PP change per year**	**p-value**	**First survey % [95% CI] (year)**	**Last survey % [95% CI] (year)**	**Average PP change per year**	**p-value**	**First survey % [95% CI] (year)**	**Last survey % [95% CI] (year)**	**Average PP change per year**	**p-value**
Mexico	97.2 [95.5,98.2] (2006)	96.5 [93.6,98.1] (2014-15)	-0.1	.980	83.0 [79.8,85.7] (2006)	79.6 [74.1,84.2] (2014-15)	-0.4	.700	55.9 [50.6,61.1] (2006)	74.8 [69.8,79.3] (2014-15)	+2.2	.004
Netherlands	81.4 [77.5,84.7] (2011)	91.8 [90.1,93.1] (2022)	0.9	.000	83.8 [81.9,85.5] (2008)	85.2 [83.2,87.1] (2022)	+0.1	.130	55.6 [52.1,59.0] (2011)	70.7 [68.2,73.1] (2022)	+1.4	.001
New Zealand	NA	NA	NA	NA	NA	NA	NA	NA	78.2 [74.9,81.1] (2007-08)	76.3 [72.5,79.8] (2020-21)	-0.1	.760
Poland	88.5 [84.6,91.6] (2016)	84.7 [79.8,88.6] (2018)	-1.9	.180	83.8 [78.3,88.1] (2016)	78.9 [73.0,83.8] (2018)	-2.4	.180	61.2 [55.4,66.7] (2016)	64.5 [58.7,69.9] (2018)	+1.6	.740
Republic of Korea	91.2 [89.3,92.8] (2020)	87.9 [85.4,90.1] (2021)	-3.3	.770	66.2 [62.6,69.7] (2020)	66.3 [63.1,69.3] (2021)	0.0	.940	37.0 [33.5,40.6] (2005)	71.0 [66.1,75.4] (2021)	+2.1	.000
Romania	90.3 [87.6,92.4] (2016)	86.3 [81.9,89.7] (2018)	-2.0	.180	88.5 [85.1,91.2] (2016)	86.6 [82.4,90.0] (2018)	-0.9	.950	78.5 [74.8,81.8] (2016)	76.5 [71.2,81.2] (2018)	-1.0	.520
Spain	93.2 [88.9,95.9] (2016)	94.6 [91.8,96.4] (2021)	+0.3	.420	82.8 [77.5,87.1] (2016)	88.4 [85.5,90.8] (2021)	+1.1	.040	56.6 [49.9,63.0] (2016)	65.6 [59.8,70.9] (2021)	+1.8	.050
Thailand	94.3 [91.5,96.1] (2005)	94.0 [92.9,95.0] (2008)	-0.1	.840	69.0 [65.8,72.0] (2009)	71.2 [66.8,75.3] (2012)	+0.7	.540	44.0 [40.6,47.4] (2005)	83.9 [81.6,85.9] (2012)	-5.7	.000
United States	94.4 [93.2,95.3] (2002)	92.1 [90.6,93.4] (2003)	-2.2	.250	86.4 [84.7,88.0] (2002)	86.4 [84.0,88.4] (2020)	0.0	.930	73.3 [71.1,75.4] (2002)	77.2 [74.4,79.8] (2020)	+0.2	.800
Uruguay	96.8 [94.3,98.2] (2006)	96.0 [94.1,97.3] (2014)	-0.1	.530	95.3 [93.0,96.9] (2006)	89.4 [86.6,91.7] (2014)	-0.7	.030	43.0 [38.4,47.8] (2006)	65.1 [61.5,68.5] (2014)	+2.8	.000
Zambia	82.0 [79.0,84.6] (2012)	79.0 [73.8,83.5] (2014)	-1.5	.080	76.1 [71.9,79.9] (2012)	72.2 [68.9,75.2] (2014)	-2.0	.100	45.1 [38.7,51.6] (2012)	48.1 [43.1,53.2] (2014)	+1.5	.280

Abbreviations: NA=no data available; PP=percentage points
[a] Data collected in the UK in 2002 and 2003, and England in 2020.

Supplementary table 8. Changes in knowledge that smoking causes lung cancer, heart attack, and stroke among adults who smoke in GATS countries.

GATS Country	Smoking causes lung cancer				Smoking causes heart attack				Smoking causes stroke			
	First survey % [95% CI] (year)	Last survey % [95% CI] (year)	Average PP change per year	p-value	First survey % [95% CI] (year)	Last survey % [95% CI] (year)	Average PP change per year	p-value	First survey % [95% CI] (year)	Last survey % [95% CI] (year)	Average PP change per year	p-value
Argentina	98.4 [97.1, 99.1] (2012)	NA	NA	NA	92.1 [88.7, 94.5] (2012)	NA	NA	NA	72.8 [64.7, 79.6] (2012)	NA	NA	NA
Bangladesh	93.0 [91.1, 94.4] (2009)	97.1 [95.8, 98.0] (2017)	+0.5	.015	88.9 [86.7, 90.8] (2009)	93.5 [91.9, 94.8] (2017)	+0.6	.009	85.9 [83.3, 88.2] (2009)	94.1 [92.4, 95.5] (2017)	+1.0	.000
Botswana	92.6 [89.5, 94.8] (2017)	NA	NA	NA	81.0 [76.6, 84.7] (2017)	NA	NA	NA	74.0 [68.9, 78.4] (2017)	NA	NA	NA
Brazil	97.5 [97.1, 98.0] (2008)	NA	NA	NA	88.6 [87.5, 89.5] (2008)	NA	NA	NA	76.7 [75.3, 78.1] (2008)	NA	NA	NA
Cameroon	89.9 [85.5, 93.1] (2013)	NA	NA	NA	68.6 [61.2, 75.1] (2013)	NA	NA	NA	61.3 [54.5, 67.7] (2013)	NA	NA	NA
China	79.4 [75.6, 82.8] (2010)	78.4 [76.1, 80.5] (2018)	-0.1	.820	38.5 [34.3, 42.8] (2010)	46.4 [43.5, 49.3] (2018)	+1.0	.110	26.9 [23.0, 31.2] (2010)	37.4 [34.7, 40.2] (2018)	+1.3	0.018
Costa Rica	96.9 [95.0, 98.0] (2015)	NA	NA	NA	93.3 [91.0, 95.1] (2015)	NA	NA	NA	72.0 [66.9, 76.6] (2015)	NA	NA	NA
Egypt	97.4 [96.2, 98.2] (2009)	NA	NA	NA	96.9 [96.0, 97.6] (2009)	NA	NA	NA	89.8 [88.2, 91.2] (2009)	NA	NA	NA
Ethiopia	67.1 [48.2, 81.7] (2016)	NA	NA	NA	54.6 [39.2, 69.1] (2016)	NA	NA	NA	29.6 [19.2, 42.8] (2016)	NA	NA	NA
Greece	95.1 [92.7, 96.8] (2013)	NA	NA	NA	88.1 [83.5, 91.5] (2013)	NA	NA	NA	69.3 [61.8, 75.9] (2013)	NA	NA	NA
India	86.2 [84.7, 87.6] (2009)	91.9 [90.9, 92.8] (2016)	0.8	.042	62.3 [60.1, 64.4] (2009)	75.3 [73.6, 77.0] (2016)	+1.9	.000	47.4 [45.0, 49.7] (2009)	63.1 [61.1, 65.1] (2016)	+2.3	.000
Indonesia	78.8 [75.4, 81.9] (2011)	NA	NA	NA	78.0 [74.2, 81.3] (2011)	NA	NA	NA	40.4 [35.6, 45.3] (2011)	NA	NA	NA
Kazakhstan	78.9 [75.1, 82.3] (2014)	NA	NA	NA	54.8 [50.4, 59.0] (2014)	NA	NA	NA	55.2 [50.8, 59.5] (2014)	NA	NA	NA
Kenya	85.6 [80.6, 89.4] (2014)	NA	NA	NA	60.0 [53.7, 66.0] (2014)	NA	NA	NA	45.7 [38.4, 53.1] (2014)	NA	NA	NA

GATS Country	Smoking causes lung cancer				Smoking causes heart attack				Smoking causes stroke			
	First survey % [95% CI] (year)	Last survey % [95% CI] (year)	Average PP change per year	p-value	First survey % [95% CI] (year)	Last survey % [95% CI] (year)	Average PP change per year	p-value	First survey % [95% CI] (year)	Last survey % [95% CI] (year)	Average PP change per year	p-value
Malaysia	88.7 [85.1, 91.5] (2011)	NA	NA	NA	84.8 [80.9, 88.1] (2011)	NA	NA	NA	75.4 [70.9, 79.4] (2011)	NA	NA	NA
Mexico	97.5 [96.5, 98.3] (2009)	98.0 [97.1, 98.6] (2015)	+0.1	.590	84.3 [81.9, 86.5] (2009)	87.5 [85.3, 89.4] (2015)	+0.5	.120	60.6 [57.5, 63.6] (2009)	68.1 [65.2, 70.8] (2015)	+1.2	.046
Nigeria	55.6 [48.4, 62.6] (2012)	NA	NA	NA	64.3 [57.3, 70.8] (2012)	NA	NA	NA	32.2 [25.5, 39.8] (2012)	NA	NA	NA
Pakistan	83.6 [79.8, 86.8] (2014)	NA	NA	NA	86.9 [83.7, 89.6] (2014)	NA	NA	NA	54.6 [49.1, 60.1] (2014)	NA	NA	NA
Panama	96.0 [94.1, 97.3] (2013)	NA	NA	NA	84.0 [78.2, 88.5] (2013)	NA	NA	NA	76.8 [70.0, 82.4] (2013)	NA	NA	NA
Philippines	92.4 [90.6, 93.8] (2009)	93.6 [92.2, 94.8] (2015)	+0.2	.520	76.6 [74.1, 78.9] (2009)	82.4 [79.9, 84.6] (2015)	+1.0	.028	69.9 [67.1, 72.5] (2009)	75.3 [72.3, 78.2] (2015)	+0.9	.090
Poland	94.9 [93.6, 95.9] (2009)	NA	NA	NA	79.8 [77.5, 82.0] (2009)	NA	NA	NA	58.6 [55.7, 61.5] (2009)	NA	NA	NA
Qatar	90.0 [86.4, 92.7] (2013)	NA	NA	NA	88.7 [85.3, 91.4] (2013)	NA	NA	NA	70.0 [64.9, 74.6] (2013)	NA	NA	NA
Romania	96.8 [95.4, 97.7] (2011)	87.5 [84.3, 90.1] (2018)	-1.3	.000	87.0 [84.3, 89.2] (2011)	72.4 [68.1, 76.3] (2018)	-2.1	.000	87.2 [84.5, 89.6] (2011)	73.2 [68.7, 77.3] (2018)	-2.0	.000
Russian Federation	96.0 [95.0, 96.8] (2009)	87.8 [85.8, 89.6] (2016)	-1.2	.000	69.2 [66.4, 71.8] (2009)	74.1 [71.2, 76.8] (2016)	+0.7	.170	63.5 [60.5, 66.3] (2009)	72.0 [68.9, 74.9] (2016)	+1.2	.027
Senegal	88.4 [83.0, 92.3] (2015)	NA	NA	NA	71.4 [64.4, 77.5] (2015)	NA	NA	NA	66.1 [58.6, 72.8] (2015)	NA	NA	NA
Thailand	97.2 [96.4, 97.8] (2009)	96.3 [95.0, 97.2] (2011)	-0.4	.080	74.5 [72.2, 76.6] (2009)	74.9 [72.3, 77.3] (2011)	+0.2	.670	79.7 [77.2, 82.1] (2009)	78.7 [76.1, 81.0] (2011)	-0.5	.510
Turkey	98.7 [98.0, 99.2] (2008)	95.3 [94.0, 96.3] (2016)	-0.4	.002	96.3 [95.2, 97.1] (2008)	95.3 [94.1, 96.3] (2016)	-0.1	.460	85.2 [83.2, 87.0] (2008)	87.7 [85.7, 89.4] (2016)	+0.3	.290
Uganda	82.1 [77.9, 85.7] (2013)	NA	NA	NA	68.1 [62.7, 73.0] (2013)	NA	NA	NA	36.4 [30.5, 42.8] (2013)	NA	NA	NA
Ukraine	92.2 [90.5, 93.6] (2010)	86.9 [83.7, 89.5] (2017)	-0.8	.060	77.1 [74.5, 79.5] (2010)	77.9 [74.6, 80.9] (2017)	+0.1	.780	75.0 [72.2, 77.6] (2010)	76.8 [73.5, 79.7] (2017)	+0.3	.550
United Republic of Tanzania	81.0 [76.0, 85.1] (2018)	NA	NA	NA	57.6 [51.4, 63.6] (2018)	NA	NA	NA	39.8 [33.5, 46.4] (2018)	NA	NA	NA

GATS Country	Smoking causes lung cancer				Smoking causes heart attack				Smoking causes stroke			
	**First survey % [95% CI] (year)**	**Last survey % [95% CI] (year)**	**Average PP change per year**	**p-value**	**First survey % [95% CI] (year)**	**Last survey % [95% CI] (year)**	**Average PP change per year**	**p-value**	**First survey % [95% CI] (year)**	**Last survey % [95% CI] (year)**	**Average PP change per year**	**p-value**
Uruguay	97.2 [95.8, 98.2] (2009)	95.9 [94.0, 97.2] (2017)	-0.2	.300	93.3 [91.0, 95.1] (2009)	91.4 [89.2, 93.2] (2017)	-0.2	.370	79.9 [76.6, 82.9] (2009)	76.8 [73.9, 79.6] (2017)	-0.4	.480
Viet Nam	94.5 [93.1, 95.6] (2010)	94.7 [93.1, 96.0] (2015)	0.0	.920	58.1 [55.2, 61.0] (2010)	68.6 [65.6, 71.4] (2015)	+2.1	.001	65.6 [62.7, 68.4] (2010)	68.3 [65.5, 71.0] (2015)	+0.5	.360

Abbreviations: NA=no data available; PP=percentage points

Supplementary table 9. Changes in knowledge that SHS causes lung cancer and heart disease among adults who smoke in ITC countries.

	Secondhand smoke causes lung cancer				Secondhand smoke causes heart disease			
**ITC Country**	**First survey % [95% CI] (year)**	**Last survey % [95% CI] (year)**	**Average PP change per year**	**p-value**	**First survey % [95% CI] (year)**	**Last survey % [95% CI] (year)**	**Average PP change per year**	**p-value**
Australia	60.6 [57.8,63.3] (2003)	72.5 [69.4,75.3] (2008-09)	+2.2	.003	49.1 [45.4,52.8] (2010-11)	39.3 [35.3,43.5] (2013)	-3.9	.024
Bangladesh	87.1 [81.0,91.4] (2009)	94.9 [92.1,96.7] (2014-15)	+1.4	.000	56.9 [51.0,62.7] (2010)	88.4 [82.6,92.4] (2014-15)	+7.0	.000
Brazil	78.0 [75.2,80.6] (2009)	80.7 [77.9,83.2] (2016-17)	+0.3	.380	NA	NA	NA	NA
Canada	74.3 [71.9,76.5] (2003)	76.8 [74.8,78.7 (2020)	+0.1	.690	61.6 [58.1,64.9] (2010-11)	50.2 [47.9,52.5] (2020)	-1.2	.250
China	56.9 [54.6,59.2] (2006)	73.0 [69.4,76.3] (2013-15)	+2.0	.000	43.3 [40.6,46.1] (2009)	53.7 [50.7,56.7] (2013-15)	+2.1	.036
England/UK[a]	59.7 [56.9,62.4] (2003)	65.9 [63.2,68.4] (2020)	+0.4	.890	53.0 [48.8,57.2] (2010-11)	44.8 [42.2,47.5] (2020)	-0.9	.600
France	94.0 [92.6,95.2] (2006-07)	75.7 [72.9,78.3] (2019)	-1.5	.000	55.8 [52.8,58.9] (2019)	NA	NA	NA
Germany	80.6 [78.2,82.8] (2007)	58.2 [53.5,62.8] (2018)	-2.0	.000	41.8 [35.2,48.7] (2016)	44.9 [38.3,51.8] (2018)	+1.6	.310
Greece	70.8 [64.5,76.4] (2016)	64.8 [58.8,70.3] (2018)	-3.0	.110	63.8 [57.6,69.7] (2016)	59.8 [53.1,66.1] (2018)	-2.0	.330
Hungary	58.7 [52.4,64.7] (2016)	60.1 [53.8,66.1] (2018)	+0.7	.770	44.5 [37.8,51.4] (2016)	49.0 [42.9,55.1] (2018)	+2.2	.220
India	74.8 [70.6,78.5] (2010-11)	88.4 [84.6,91.3] (2019-19)	+1.7	.000	65.0 [60.6,69.1] (2010-11)	86.9 [82.7,90.2] (2018-19)	+2.7	.000
Japan	74.9 [73.3,76.4] (2018)	68.9 [66.5,71.1] (2021)	-2.0	.080	64.3 [62.4,66.1] (2018)	56.2 [53.4,59.0] (2021)	-2.7	.046
Kenya	68.8 [63.4,73.7] (2012)	74.3 [69.1,79.0] (2018)	+0.9	.120	50.9 [43.6,58.1] (2012)	63.9 [58.1,69.3] (2018)	+2.2	.041
Malaysia	81.2 [76.3,85.3] (2005)	72.3 [68.6,75.8] (2020)	-0.6	.270	82.5 [80.1,84.7] (2013-14)	62.6 [58.6,66.4] (2020)	-3.3	.000
Mauritius	84.3 [79.5,88.1] (2009)	85.2 [79.8,89.2] (2011)	+0.4	.210	NA	NA	NA	NA

	**Secondhand smoke causes lung cancer**				**Secondhand smoke causes heart disease**			
**ITC Country**	**First survey % [95% CI] (year)**	**Last survey % [95% CI] (year)**	**Average PP change per year**	**p-value**	**First survey % [95% CI] (year)**	**Last survey % [95% CI] (year)**	**Average PP change per year**	**p-value**
Mexico	90.7 [87.9,92.9] (2006)	91.2 [87.2,94.1] (2014-15)	+0.1	.330	74.7 [71.2,78.0] (2008)	79.1 [73.7,83.7] (2014-15)	+0.7	.110
Netherlands	69.8 [67.5,72.1] (2008)	63.9 [61.3,66.5] (2022)	-0.4	.490	38.1 [35.1,41.3] (2012)	53.8 [51.2,56.3] (2022)	+1.6	.023
New Zealand	80.8 [77.9,83.4] (2007-08)	61.2 [57.1,65.2] (2020-21)	-1.5	.019	58.0 [53.8,62.0] (2016-17)	47.8 [43.8,51.8] (2020-21)	-2.5	.027
Poland	73.5 [68.0,78.4] (2016)	68.8 [61.4,75.3] (2018)	-2.4	.290	65.7 [59.7,71.2] (2016)	60.1 [52.6,67.1] (2018)	-2.8	.130
Republic of Korea	79.5 [76.7,82.1] (2005)	75.4 [69.6,80.4] (2021)	-0.3	.100	55.8 [52.8,58.7] (2010)	60.3 [55.6,64.8] (2021)	+0.4	.780
Romania	81.9 [78.3,85.1] (2016)	77.7 [72.3,82.3] (2018)	-2.1	.400	73.8 [68.9,78.1] (2016)	73.8 [68.7,78.4] (2018)	0.0	.780
Spain	67.2 [60.3,73.5] (2016)	69.8 [64.9,74.3] (2021)	+0.5	.550	51.8 [44.7,58.9] (2016)	56.5 [50.8,62.0] (2021)	+0.9	.440
Thailand	91.1 [88.2,93.4] (2005)	96.8 [95.4,97.8] (2012)	+0.8	.000	82.2 [78.2,85.6] (2012)	NA	NA	NA
United States	62.7 [60.0,65.2] (2003)	68.1 [65.1,70.9] (2020)	+0.3	.990	51.6 [47.5,55.6] (2010-11)	44.7 [41.4,47.9] (2020)	-0.7	.440
Uruguay	89.3 [86.5,91.6] (2006)	79.5 [76.0,82.7] (2014)	-1.2	.015	69.1 [64.3,73.5] (2012)	69.6 [65.9,73.1] (2014)	+0.2	.900
Zambia	64.3 [57.0,70.9] (2012)	68.2 [62.9,73.1] (2014)	+2.0	.160	59.4 [52.2,66.2] (2012)	65.3 [60.5,69.9] (2014)	+3.0	.080

Abbreviations: NA=no data available; PP=percentage points
[a] Data collected in the UK in 2003 and 2010-11, and England in 2020.

Supplementary table 10. Changes in knowledge that SHS causes lung cancer and heart disease among adults who smoke in GATS countries.

GATS Country	Secondhand smoke causes lung cancer				Secondhand smoke causes heart disease			
	First survey % [95% CI] (year)	Last survey % [95% CI] (year)	Average PP change per year	p-value	First survey % [95% CI] (year)	Last survey % [95% CI] (year)	Average PP change per year	p-value
Argentina	NA	NA	NA	NA	NA	NA	NA	NA
Bangladesh	93.0 [91.3, 94.4] (2009)	95.4 [94.1, 96.4] (2017)	+0.3	.360	90.6 [88.6, 92.3] (2009)	93.5 [91.9, 94.8] (2017)	+0.4	.160
Botswana	NA	NA	NA	NA	NA	NA	NA	NA
Brazil	NA	NA	NA	NA	NA	NA	NA	NA
Cameroon	78.8 [73.3, 83.4] (2013)	NA	NA	NA	72.9 [65.5, 79.2] (2013)	NA	NA	NA
China	80.5 [75.8, 84.4] (2010)	61.6 [59.0, 64.2] (2018)	-2.4	.050	43.8 [37.7, 50.0] (2010)	37.7 [35.1, 40.4] (2018)	-0.8	.360
Costa Rica	93.5 [90.8, 95.5] (2015)	NA	NA	NA	88.2 [84.8, 90.9] (2015)	NA	NA	NA
Egypt	94.0 [92.7, 95.1] (2009)	NA	NA	NA	91.8 [90.3, 93.1] (2009)	NA	NA	NA
Ethiopia	NA	NA	NA	NA	NA	NA	NA	NA
Greece	68.1 [61.1, 74.4] (2013)	NA	NA	NA	66.9 [59.8, 73.4] (2013)	NA	NA	NA
India	NA	NA	NA	NA	NA	NA	NA	NA
Indonesia	NA	NA	NA	NA	NA	NA	NA	NA
Kazakhstan	NA	NA	NA	NA	NA	NA	NA	NA
Kenya	78.6 [72.4, 83.7] (2014)	NA	NA	NA	67.4 [60.7, 73.5] (2014)	NA	NA	NA
Malaysia	81.2 [77.3, 84.6] (2011)	NA	NA	NA	77.4 [73.0, 81.2] (2011)	NA	NA	NA
Mexico	NA	NA	NA	NA	NA	NA	NA	NA
Nigeria	41.9 [35.1, 49.0] (2012)	NA	NA	NA	54.0 [46.6, 61.2] (2012)	NA	NA	NA
Pakistan	69.3 [64.6, 73.5] (2014)	NA	NA	NA	73.1 [68.6, 77.1] (2014)	NA	NA	NA
Panama	NA	NA	NA	NA	NA	NA	NA	NA
Philippines	NA	NA	NA	NA	NA	NA	NA	NA
Poland	91.4 [89.7, 92.9] (2009)	NA	NA	NA	83.7 [81.4, 85.8] (2009)	NA	NA	NA
Qatar	NA	NA	NA	NA	NA	NA	NA	NA
Romania	NA	NA	NA	NA	NA	NA	NA	NA
Russian Federation	NA	NA	NA	NA	NA	NA	NA	NA

Chung-Hall J, *et al. BMJ Open* 2026; 16:e107667. doi: 10.1136/bmjopen-2025-107667

GATS Country	Secondhand smoke causes lung cancer				Secondhand smoke causes heart disease			
	First survey % [95% CI] (year)	Last survey % [95% CI] (year)	Average PP change per year	p-value	First survey % [95% CI] (year)	Last survey % [95% CI] (year)	Average PP change per year	p-value
Senegal	83.8 [77.9, 88.4] (2015)	NA	NA	NA	85.9 [80.4, 90.1] (2015)	NA	NA	NA
Thailand	95.3 [94.3, 96.1] (2009)	96.4 [95.5, 97.1] (2011)	+0.6	.090	70.4 [67.8, 72.9] (2009)	68.5 [65.8, 71.2] (2011)	-0.9	.340
Turkey	93.5 [92.2, 94.7] (2012)	93.0 [91.5, 94.3] (2016)	-0.1	.830	91.3 [89.3, 92.9] (2012)	92.8 [91.3, 94.0] (2016)	+0.4	.240
Uganda	72.1 [66.6, 77.1] (2013)	NA	NA	NA	66.0 [60.1, 71.4] (2013)	NA	NA	NA
Ukraine	75.3 [72.4, 78.1] (2010)	69.4 [65.9, 72.6] (2017)	-0.9	.490	78.8 [76.3, 81.2] (2010)	71.1 [67.8, 74.1] (2017)	-1.1	.100
United Republic of Tanzania	67.0 [61.0, 72.4] (2018)	NA	NA	NA	56.0 [50.0, 61.9] (2018)	NA	NA	NA
Uruguay	NA	NA	NA	NA	NA	NA	NA	NA
Viet Nam	71.6 [68.8, 74.2] (2010)	85.1 [82.7, 87.3] (2015)	+2.7	.010	14.6 [12.5, 16.9] (2010)	21.3 [19.0, 23.9] (2015)	+1.4	.018

Abbreviations: NA=no data available; PP=percentage points

Supplementary file 1A. ITC and GATS Project authors, ITC principal investigators, ITC funding statements, ITC ethics statements, and ITC acknowledgements.

Janet Chung-Hall,[1] Geoffrey T. Fong, [1,2,3] Gang Meng,[1] Lorraine V. Craig,[1] Eunice O. Indome[1], for the ITC Project Collaborators, Alissa C. Kress,[4] Jing Shi,[4,5] and Indu B. Ahluwalia[4] for the Global Adult Tobacco Survey Team

Affiliation(s):

1. University of Waterloo, Psychology, Waterloo, ON, Canada
2. University of Waterloo, School of Public Health Sciences, Waterloo, ON, Canada
3. Ontario Institute for Cancer Research, Toronto, ON, Canada
4. National Center for Chronic Disease Prevention and Health Promotion Office on Smoking and Health, Atlanta, GA, US
5. CDC Foundation Inc., Noninfectious Disease Programs, Atlanta, GA, US

ITC Project Collaborators Individual Author Names and Email Addresses:

Raphaël Andler[6] (Raphael.ANDLER@santepubliquefrance.fr), Maansi Bansal-Travers[7] (Maansi.Travers@roswellpark.org), Tonatiuh Barrientos-Gutiérrez[8] (tbarrientos@insp.mx), Marcelo Boado[9] (mrbmsoc2001@yahoo.com.ar), Premduth Burhoo[10] (burhoo@hotmail.com), K. Michael Cummings[11] (cummingk@musc.edu), Cristina de Abreu Perez[12] (cristinadeabreuperez@gmail.com), Tibor Demjén[13] (tibor.demjen@gmail.com), Katherine A. East[14] (K.East@bsms.ac.uk), Richard Edwards[15] (richard.edwards@flinders.edu.au), Esteve Fernández[16-20] (efernandez@iconcologia.net), Marcela Fu[16-19,21] (mfu@iconcologia.net), Coral Gartner[22] (c.gartner@uq.edu.au), Fastone M. Goma[23] (gomafm@yahoo.co.uk), Prakash C. Gupta[24] (pcgupta@healis.org), A.K.M. Ghulam Hussain[25] (think2100@gmail.com), Andrew Hyland[7] (Andrew.Hyland@RoswellPark.org), Lawrence Ikamari[26] (likamari@uonbi.ac.ke), Aree Jampaklay[27] (aree.ude@mahidol.ac.th), Yuan Jiang[28,29] (jiangyuan88@vip.sina.com), Kota Katanoda[30] (kkatanod@ncc.go.jp), Gil-yong Kim[31] (kimgy@khepi.or.kr) , Su Young Kim[32] (suyoung@ncrc.or.kr), Sungkyu Lee[33,34] (wwwvince77@gmail.com), Farizah Mohd Hairi[35,36] (farizah@um.edu.my), Ute Mons[37–39] (ute.mons@uk-koeln.de), Nigar Nargis[40] (nigar.nargis@cancer.org), Amer Siddiq Amer Nordin[35,36] (amersiddiq@um.edu.my), Jane Rahedi Ong'ang'o[41] (jrnabongo@gmail.com), Maizurah Omar[42] (maizurahomar@gmail.com), Mangesh S. Pednekar[24] (pednekarm@healis.org), Krzysztof Przewoźniak[43,44] (krzysztof.przewozniak@wp.pl), Hong Gwan Seo[45] (hgseo@designhospital.co.krmailto:), James F. Thrasher[8,46] (THRASHER@mailbox.sc.edu), Antigona Trofor[47] (atrofor@yahoo.com), Constantine I. Vardavas[48] (vardavas@tobcontrol.eu), Andrew M. Waa[49] (andrew.waa@otago.ac.nz), Marc C. Willemsen[50] (marc.willemsen@maastrichtuniversity.nl), Nick Wilson[49] (nick.wilson@otago.ac.nz), Witold A Zatoński[51-53] (wazatonski@gmail.com)

ITC Project Collaborators Affilation(s):

6. Prevention and Health Promotion Department, Santé publique France, Saint-Maurice, France
7. Department of Health Behavior, Roswell Park Comprehensive Cancer Center, Buffalo, NY, USA
8. Center for Population Health Research, National Institute of Public Health, Cuernavaca, Mexico
9. Department of Sociology, Faculty of Social Sciences, University of the Republic, Montevideo, Uruguay
10. Mauritius Institute of Health, Pamplemousses, Mauritius
11. Department of Psychiatry & Behavioral Sciences, Medical University of South Carolina, Charleston, South Carolina, USA
12. National School of Public Health Sérgio Arouca, Oswaldo Cruz Foundation, Rio de Janeiro, Brazil

13. Hungarian Focal Point for Tobacco Control, Ministry of Interior, Hungary
14. Department of Primary Care and Public Health, Brighton and Sussex Medical School, Brighton, Sussex, UK
15. Department of Public Health, University of Otago, Wellington, New Zealand
16. Tobacco Control Unit, WHO Collaborating Center, Institut Català d'Oncologia (ICO), l'Hospitalet de Llobregat, Spain
17. Tobacco Control Research Group, Epidemiology, Public Health, Cancer Prevention and Palliative Care Programme-EPIBELL, Institut d'Investigació Biomèdica de Bellvitge (IDIBELL), l'Hospitalet de Llobregat, Spain
18. Centre for Biomedical Research in Respiratory Disease, Institute of Health Carlos III, Madrid, Spain
19. Department of Clinical Sciences, School of Medicine and Health Sciences, University of Barcelona, Barcelona, Catalonia, Spain
20. Secretariat of Public Health, Department of Health, Generalitat de Catalunya, Barcelona, Spain
21. Department of Public Health, Mental health, and Maternal and Child Health Nursing, School of Nursing, University of Barcelona, Barcelona, Catalonia, Spain
22. NHMRC Centre of Research Excellence on Achieving the Tobacco Endgame, Faculty of Health, Medicine, and Behavioural Sciences, The University of Queensland, Brisbane, Queensland, Australia
23. Centre for Primary Care Research, University of Zambia, Lusaka, Zambia
24. Healis Sekhsaria Institute for Public Health, Navi Mumbai, Maharashtra, India
25. Department of Economics, University of Dhaka, Dhaka, Bangladesh
26. Population Studies and Research Institute, University of Nairobi, Nairobi, Kenya
27. Institute for Population and Social Research, Mahidol University, Salaya, Thailand
28. National Tobacco Control Office, Chinese Center for Disease Control and Prevention, Beijing, China
29. ThinkTank Research Center for Health Development, Beijing, China
30. National Cancer Center Institute for Cancer Control, Tokyo, Japan
31. National Tobacco Control Center, Korean Health Promotion Institute, Seoul, Republic of Korea
32. National Center for the Rights of the Child, Seoul, Republic of Korea
33. Korea Center for Tobacco Control Research and Education, Seoul, Republic of Korea
34. Graduate School of Public Health, Institute for Health Promotion, Yonsei University, Seoul, Republic of Korea
35. Nicotine Addiction Research Group, University of Malaya Centre of Addiction Sciences, University of Malaya, Kuala Lumpur, Malaysia
36. Department of Social and Preventive Medicine, Faculty of Medicine, University of Malaya, Kuala Lumpur, Malaysia
37. Division of Primary Cancer Prevention, German Cancer Research Center (DKFZ), Heidelberg, Germany
38. Cancer Prevention Unit & WHO Collaborating Centre, German Cancer Research Center (DKFZ), Heidelberg, Germany
39. Medical Faculty Mannheim, Heidelberg University, Mannheim, Germany
40. Department of Surveillance, Prevention, and Health Services Research, American Cancer Society, Atlanta, GA, USA
41. Centre for Respiratory Disease Research, Kenya Medical Research Institute, Nairobi, Kenya
42. National Poison Centre, Universiti Sains Malaysia, Penang, Malaysia
43. Global Institute of Family Health, University of Kalisz, Kalisz, Poland
44. Global Research, Implementation and Training Lab, Department of Population and Public Health Sciences, Keck School of Medicine, University of Southern California, Los Angeles, CA, USA
45. Department of Disease Screening, Design Hospital, Jeonju, Republic of Korea
46. Department of Health Promotion, Education & Behavior, Arnold School of Public Health, University of South Carolina, Columbia, USA
47. Grigore T. Popa University of Medicine and Pharmacy, Iasi, Romania
48. Department of Hygiene, Epidemiology and Medical Statistics, School of Medicine, National and Kapodistrian University of Athens, Greece
49. College of Medicine and Public Health, Flinders University, South Australia, Australia
50. Department of Health Promotion, Maastricht University (CAPHRI), Maastricht, The Netherlands

51. Maria Skłodowska-Curie National Research Institute of Oncology, Warsaw, Poland
52. European Observatory of Health Inequalities, President Stanisław Wojciechowski State University of Applied Sciences, Kalisz, Poland
53. Global Institute of Family Health, University of Kalisz, Kalisz, Poland

Ethics statement:
The **ITC Four Country** (Australia, Canada, United Kingdom, and United States) survey protocols and all materials, including the survey questionnaires, were cleared for ethics by Research Ethics Board, University of Waterloo, Canada (REB#10556, REB#13978, REB#17469), Human Research Ethics at Cancer Council Victoria, Australia (IRB HREC 0211), Office of Research Subject Protection, Roswell Comprehensive Cancer Center, US (IRB NT 02-20), and Research Ethics Office, King's College London, UK (IRB PNM/13/14-151). The **ITC Four Country Smoking and Vaping** (Australia, Canada, England, and United States) survey protocols and all materials, including the survey questionnaires, were cleared for ethics by Research Ethics Board, University of Waterloo, Canada (REB#20803/30570, REB#20803/30878), Human Research Ethics at Cancer Council Victoria, Australia (IRB HREC 1603), Human Research Ethics Committee, The University of Queensland, Australia (IRB #2016000330), Research Ethics Office, King's College London, UK (IRB RESCM-17/18-2240), and ethics clearance at the Medical University of South Carolina, US was waived due to minimal risk. The **ITC Bangladesh** survey protocols and all materials, including the survey questionnaires, were cleared for ethics by Research Ethics Board, University of Waterloo, Canada (REB#15019, REB#19862/30312), and Bangladesh Medical Research Council (IRB BMRC/ERC/2013-2016/1729, IRB BMRC/ERC/2007-2010/1372). The **ITC Brazil** survey protocols and all materials, including the survey questionnaires, were cleared for ethics by Research Ethics Board, University of Waterloo, Canada (REB#15076, REB#21456/30826), and National Cancer Institute of Brazil (INCA)—IRB 99/08). The **ITC China** survey protocols and all materials, including the survey questionnaires, were cleared for ethics by Research Ethics Board, University of Waterloo, Canada (REB#12539, REB#15305, REB#17014/30105), Cancer Council Victoria, Australia (IRB IER0803), and Chinese Center for Disease Control and Prevention (IRB 201114, IRB 201325). The **ITC France** (first cohort) survey protocols and all materials, including the survey questionnaires, were cleared for ethics by Research Ethics Board, University of Waterloo, Canada (REB#13005, 183/30190). The **ITC France** (second cohort) survey protocols and all materials, including the survey questionnaires, were cleared for ethics by Research Ethics Board, University of Waterloo, Canada (REB#40720). The **ITC Germany** (first cohort) survey protocols and all materials, including the survey questionnaires, were cleared for ethics by Research Ethics Board, University of Waterloo, Canada (REB#13858), and Heidelberg Medical School, Germany (IRB S-138/2007). The **EUREST-PLUS** (**ITC 6 European Country** in Germany (second cohort), Greece, Hungary, Poland, Romania, and Spain) survey protocols and all materials, including the survey questionnaires, were cleared for ethics by Research Ethics Board, University of Waterloo, Canada (REB#21262/30709), Comisia de Etica Iuliu Hatieganu University of Medicine and Pharmacy, Romania (IRB 155), Research Ethics Boards of the Bellvitge University Hospital, (IRB PR100/16), Committee and Dean of the Department of Health Care and Life Sciences, State College of Higher Vocational Education, Poland (IRB 1/2016), Research and Ethics Committee, Medical School, University of Athens, Greece (IRB 1516023880), Scientific and Research Committee, Medical Research Council, Hungary (IRB 11012-2-2016/EKU 0198/16, IRB 46344-2/2017/EKU), and Heidelberg Medical School, Germany (IRB 196/2016). The **ITC EUREST-PLUS Spain** (third wave) survey protocols and all materials, including the survey questionnaires, were cleared for ethics by Research Ethics Board, University of Waterloo, Canada (REB#41105), and Research Ethics Boards of the Bellvitge University Hospital, (IRB PR100/16, IRB PR248/17). The **ITC India** survey protocols and all materials, including the survey questionnaires, were cleared for ethics by Research Ethics Board, University of Waterloo, Canada (REB#132,97, REB#15722, REB#22140/31086), and Healis Sekhsaria Institute for Public Health (IRB 00007340). The **ITC Japan** survey protocols and all materials, including the survey questionnaires, were cleared for ethics by Research Ethics Board, University of Waterloo, Canada (REB#22508/31428), National Cancer Center, Japan (IRB 2021-069), and Osaka International Cancer Institute (IRB 21054). The **ITC Kenya** survey protocols and all materials, including the survey questionnaires, were cleared for ethics by Research Ethics Board, University of Waterloo, Canada (REB#18311/30136), and University of Nairobi (IRB KNH-ERC/A/281). The **ITC Thailand** survey protocols and all materials, including the survey questionnaires, were cleared for ethics by Research Ethics Board, University of Waterloo, Canada (REB#11762, REB#15468), Roswell Park Comprehensive Cancer Center, US (NT-02-20), Cancer Council Victoria, Australia (HREC 0420), and Mahidol University, Thailand (IRB 0517·191/1003). The **ITC Malaysia** (first cohort) survey protocols and all materials, including the survey

questionnaires, were cleared for ethics by Research Ethics Board, University of Waterloo, Canada (REB#11762, REB#15468), Roswell Park Comprehensive Cancer Center, US (NT-02-20), Cancer Council Victoria, Australia (HREC 0420), and Universiti Sains Malaysia, Malaysia (USM/PPSP/Ethics Com./2004(137·4[3])). The **ITC Malaysia** (second cohort) survey protocols and all materials, including the survey questionnaires, were cleared for ethics by Research Ethics Board, University of Waterloo, Canada (REB#40825) Medical Research Ethics Committee, University Malaya, Malaysia (MREC ID#2019118-8018). The **ITC Mauritius** survey protocols and all materials, including the survey questionnaires, were cleared for ethics by Research Ethics Board, University of Waterloo, Canada (REB#15487), and Mauritius Institute of Health, Mauritius (IRB MHC/CT/NETH/MIH). The **ITC Mexico** survey protocols and all materials, including the survey questionnaires, were cleared for ethics by Research Ethics Board, University of Waterloo, Canada (REB#13195, REB#13472, REB#16353), and Instituto Nacional de Salud Publica, Mexico (IRB 759). The **ITC Netherlands** (first cohort) survey protocols and all materials, including the survey questionnaires, were cleared for ethics by Research Ethics Board, University of Waterloo, Canada (REB#14666, REB#18920/30242). The **ITC Netherlands** (second cohort) survey protocols and all materials, including the survey questionnaires, were cleared for ethics by Research Ethics Board, University of Waterloo, Canada (REB#41704). The **ITC New Zealand** (first cohort) survey protocols and all materials, including the survey questionnaires, were cleared for ethics by Research Ethics Board, University of Waterloo, Canada (REB#13547), and Multi-Region Ethics Committee, New Zealand (MEC/06/07/071). The **ITC New Zealand (EASE: Evidence for Achieving Smokefree 2025 Equitably)** (second cohort) survey protocols and all materials, including the survey questionnaires, were cleared for ethics by Research Ethics Board, University of Waterloo, Canada (REB#21211/30726, REB#42549) and Human Ethics Committee, University of Otago (IRB 15/126, IRB 20/020). The **ITC Korea** (first cohort) survey protocols and all materials, including the survey questionnaires, were cleared for ethics by Research Ethics Board, University of Waterloo, Canada (REB#12547, REB#15017), and Internal Review Board, National Cancer Center, Republic of Korea (IRB NCCNCS-05-050, IRB NCCNCS-08-172, IRB NCCNCS-10-373), The **ITC Korea** (second cohort) survey protocols and all materials, including the survey questionnaires, were cleared for ethics by Research Ethics Board, University of Waterloo, Canada (REB#21287/30768) and Internal Review Board, National Cancer Center, Republic of Korea (IRB NCCNCS—2016-0129). The **ITC Korea** (third cohort) survey protocols and all materials, including the survey questionnaires, were cleared for ethics by Research Ethics Board, University of Waterloo, Canada (REB#41512) and Korea Health Promotion Institute (IRB KHPI 120160811107AN01-2004-HR-042-02). The **ITC Uruguay** survey protocols and all materials, including the survey questionnaires, were cleared for ethics by Research Ethics Board, University of Waterloo, Canada (REB#13195, REB#13472, REB#16665/30090) and Universidad de la República, Uruguay (IRB 071140-000649-08). The **ITC Zambia** survey protocols and all materials, including the survey questionnaires, were cleared for ethics by Research Ethics Board, University of Waterloo, Canada (REB#18086/303137), University of Zambia, Zambia (IRB 021-05-12). All secondary data analysis using ITC country data were cleared for ethics by Research Ethics Board, University of Waterloo, Canada (REB#31040). All participants provided consent for participating in the ITC surveys.

Funding statement:
The **ITC Four Country Surveys** (Australia, Canada, the United Kingdom, the United States) were supported by grants from the US National Cancer Institute (R01 CA100362, R01 CA090955, P50 CA111236 (Roswell Park Transdisciplinary Tobacco Use Research Center), and P01 CA138389), Robert Wood Johnson Foundation (045734), the Canadian Institutes of Health Research (FDN-148477, MOP-57897, MOP-79551, MOP-115016), the Canadian Tobacco Control Research Initiative (014578), Australian Commonwealth Department of Health and Ageing, the National Health and Medical Research Council of Australia (265903, 450110, 1005922), and Cancer Research UK (C312/A3726, C312/A6465, C321/A11039, C25586/A19540). The **ITC Four Country Smoking and Vaping Surveys** (Australia, Canada, England, and the United States) were supported by grants from the US National Cancer Institute (P01 CA200512), the Canadian Institutes of Health Research (FDN-148477), and the National Health and Medical Research Council of Australia (APP1106451). The **ITC Bangladesh Surveys** were supported by grants from the US National Cancer Institute (P01 CA138389), the International Development Research Centre (104831-003), and Canadian Institutes for Health Research (MOP-79551, MOP-115016). The **ITC Brazil Surveys** were supported by the Instituto Nacional de Câncer (INCA, National Cancer Institute), Secretaria Nacional de Pokiticas sobre Drogas (SENAD, National Secretariat for Drug Policy), Pan-American Health Organization (PAHO), Brazil Fundação do Câncer (BR/LOA/1500048·00), and the

Canadian Institutes of Health Research (MOP-115016, FDN-148477). The **ITC China Surveys** were supported by grants from the US National Cancer Institute at the National Institutes of Health (R01 CA125116), P50 CA111236 (Roswell Park Transdisciplinary Tobacco Use Research Center), Canadian Institutes for Health Research (MOP-79551, MOP-115016), and the Chinese Center for Disease Control and Prevention. The **ITC France Surveys** (first cohort) were supported by grants from the Observatoire français des drogues et toxicomanies (OFDT), Institut national de prevention et d'education pour la sante (INPES), and the Institut nationale du cancer (INCa). The ITC France Surveys (second cohort) were supported by grants from Santé publique France, the Institut nationale du cancer (INCa), and the Canadian Institutes of Health Research (FDN-148477). The **ITC Germany Surveys** (first cohort) were supported by grants from the German Federal Ministry of Health, Dieter Mennekes-Umweltstiftung, and Germany Cancer Research Center (DKFZ). The EUREST-PLUS Project (ITC 6 European Country Surveys) in Germany (second cohort), Greece, Hungary, Poland, Romania, and Spain has received funding from the European Union's Horizon2020 research and innovation programme 681109, and additional support was provided to the University of Waterloo by the Canadian Institutes of Health Research (FDN-148477). EF is partly supported by Ministry of Universities and Research, Government of Catalonia (2017SGR319) and by the Instituto de Salud Carlos III and co-funded by the European Regional Development Fund (INT16/00211 and INT17/00103), Government of Spain, and CERCA Programme Generalitat de Catalunya for the institutional support to IDIBELL. The EUREST-PLUS Spain Survey was supported by grants from the Instituto de Salud Carlos III (PI17/01338, Co-funded by European Regional Development Fund and the Canadian Institutes for Health Research (FDN-148477). EF and MF are partly supported by the Ministry of Universities and Research, Government of Catalonia (2021SGR00906) ,and CERCA Programme Generalitat de Catalunya for the institutional support to IDIBELL. The **ITC India Surveys** were supported by grants from the US National Cancer Institute (P50 CA111236, P01 CA138389), the Canadian Institutes of Health Research (MOP-79551, MOP-115016, FDN-148477), and Healis Sekhsaria Institute for Public Health. The **ITC Japan Surveys** were supported by the Japan National Cancer Center and Research Development Fund (28-A-24) and the Canadian Institutes of Health Research (FDN-148477). The **ITC Kenya Surveys** were supported by grants from the Canadian Institutes of Health Research (MOP-115016, FDN-148477). The **ITC Thailand Surveys** were supported by grants from the US National Cancer Institute (P50 CA111236, P01 CA138389 (Roswell Park Transdisciplinary Tobacco Use Research Center), the Canadian Institutes of Health Research (MOP-79551, MOP-115016), and the ThaiHealth Promotion Foundation. The ITC Malaysia Surveys (first cohort) were supported by grants from the US National Cancer Institute (P50 CA111236 (Roswell Park Transdisciplinary Tobacco Use Research Center) and P01 CA138389), the Canadian Institutes of Health Research (MOP-79551, MOP-115016), and the Malaysian Ministry of Health. The **ITC Malaysia Surveys** (second cohort) were supported by the Malaysia Ministry of Higher Education-LRGS NanoMITe (RU029-2014), the University of Malaya Research University Grant (RU029C-2014 and RU001A-2021), and the Canadian Institutes of Health Research (FDN-148477). The **ITC Mauritius Surveys** were supported by grants from the International Development Research Centre, the Canadian Institutes of Health Research (MOP-115016), and was partially supported by the World Lung Foundation. The **ITC Mexico Surveys** were supported by grants from the Mexican Consejo Nacional de Ciencia y Tecnologia (Salud-2007-C01-70032, Salud-2013-01-202671), Bloomberg Global Initiative—International Union Against Tuberculosis and Lung Disease, the US National Cancer Institute (P50 CA111236 (Roswell Park Transdisciplinary Tobacco Use Research Center) and the Canadian Institutes for Health Research (MOP-115016). The **ITC Netherlands Surveys** (first cohort) were supported by grants from The Netherlands Organisation for Health Research and Development (ZonMw 121010008, ZonMw 200130002), The SILNE Project (funded by the European Commission through FP7 HEALTH-F3-2011-278273) and the Dutch Cancer Foundation (KWF) (UM 2014-7210). The ITC Netherlands Surveys (second cohort) were supported by grants from Longfonds (Lung Foundation Netherlands), Hartstichting (Netherlands Heart Foundation), KWF Kankerbestrijding (Dutch Cancer Society), Trombosestichting Nederland (Thrombosis Foundation), Diabetesfonds (Diabetes Fund) and the Canadian Institutes of Health Research (FDN-148477). The **ITC New Zealand Surveys** (first cohort) were supported by grants from the New Zealand Health Research Council (06/453), the New Zealand Ministry of Health, University of Otago, the US National Cancer Institute (P50 CA111236 (Roswell Park Transdisciplinary Tobacco Use Research Centre)), and the Canadian Institutes of Health Research (MOP-79551). The ITC New Zealand (EASE: Evidence for Achieving Smokefree 2025 Equitably) Surveys (second cohort) were supported by grants from the New Zealand Health Research Council (GA215F 15/072, HRC 19/641), the New Zealand Ministry of Health, and the Canadian Institutes for Health Research (FDN-148477). The **ITC Korea Surveys** (first cohort) were supported by grants from the National Cancer Center of Korea (0731040-1), the Korean Ministry of Health and Welfare, and the US National Cancer Institute (P50 CA111236, R01 CA125116) and the Canadian Institutes of Health Research (MOP-79551). The ITC Korea Survey (second cohort) was supported by a grant from the Korean Ministry of Health and Welfare and the Canadian Institutes of Health Research

(MOP-115016). The ITC Korea Surveys (third cohort) were supported by a grant from the Korean Ministry of Health and Welfare and the Canadian Institutes of Health Research (FDN-148477). The **ITC Uruguay Surveys** were supported by grants from the US National Cancer Institute (P50 CA111236 (Roswell Park Transdisciplinary Tobacco Use Research Center), P01 CA138389), and the Canadian Institutes of Health Research (MOP-115016). The ITC Zambia Surveys were supported by a grant from the Canadian Institutes of Health Research (MOP-115016). Additional support provided by the Ontario Institute for Cancer Research (Senior Investigator Grant to GTF (2002-present)), and the Canadian Cancer Society Research Institute (Senior Prevention Scientist Award to GTF (2002-09), and O. Harold Warwick Prize to GTF (2021). Financial support for **GATS** was provided by the CDC Foundation with grants from Bloomberg Philanthropies and the Bill and Melinda Gates Foundation, and/or in-country donor funding for GATS countries listed in Supplemental file 1B. The funders had no role in the design of the study; in the collection, analyses, or interpretation of data; in the writing of the manuscript, or in the decision to publish the results.

Competing interest statement:
KMC has in the past and continues to serve as a paid witness in litigation filed against cigarette manufacturers. GTF has been an expert witness or consultant for governments defending their country's policies or regulations in litigation. CG has been a paid expert witness for the Australian Government in litigation concerning nicotine product marketing. KK received a JMWH Bayer Grant from Japan Society for Menopause and Women's Health. ASAN has received an unconditional educational grant from Johnson & Johnson Malaysia Sdn. Bhd. JFT has served as paid expert witness in legal challenges against tobacco and vaping companies. All other authors have no conflicts of interest to declare.

Acknowledgements: The ITC authors would like to acknowledge and thank all ITC team members at the University of Waterloo, Canada and the ITC country teams for their contributions: **ITC Four Country** and **ITC Four Country Smoking and Vaping** teams at Roswell Park Comprehensive Cancer Center, US, King's College London, UK, and Cancer Council Victoria, University of Melbourne, and The Queensland University, Australia; **ITC Brazil** teams at the Brazilian National Cancer Institute (INCA), Alliance for the Control of Tobacco Use (ACTbr), the Federal University of Rio de Janeiro, the Oswaldo Cruz Foundation, and the International Union against Tuberculosis and Lung Disease (IUATLD); **ITC Bangladesh** teams at the Bureau of Economic Research and the Department of Economics, University of Dhaka; **ITC China** teams at the China CDC and the local CDCs in Beijing, Changsha, Changzhi, Guangzhou, Huzhou, Shanghai, Shenyang, Tongren, Yichun, Yinchuan, Xining, and Yunnan Health Education Institute at Kunming; **ITC France** (first cohort) team at l'Institut national de prevention et d'éducation pour la santé (INPES), l'Observatoire françaisdes drogues et des toxicomanies (OFDT) and l'Institut national du cancer (INCa); **ITC France** (second cohort) team at Santé publique France and l'Institut national du cancer (INCa); **ITC Germany** (first cohort) team at Deutsches Krebsforschungszentrum (DKFZ; German Cancer Research Center), Bundesministerium für Gesundheit (German Federal Ministry of Health), and Dieter Mennekes-Umweltstiftung; **ITC India** teams at Bihar--School of Preventive Oncology, West Bengal--Cancer Foundation of India, Madhya Pradesh--Madhya Pradesh Voluntary Health Association, and Maharashtra—Healis-Sekhsaria Institute for Public Health; **ITC Kenya** team at Population Studies and Research Institute (PSRI), University of Nairobi; **ITC Korea** (first cohort) teams at National Cancer Center, Asan Medical Center, Sungjyunjwan University College of Medicine, Ewha Womans University School of Medicine, Dankook University, Korean Cell Line Bank and Cancer Research Center, Hallym University Sacred Heart Hospital; **ITC Korea** (second cohort) teams at National Cancer Center and Seoul National University; **ITC Korea** (third cohort) teams at Korea Health Promotion Institute, National Cancer Center, Korea Center for National Tobacco Control Research & Education, and Seoul National University; **ITC Malaysia** (first cohort) teams at the National Poison Centre, Universiti Sains Malaysia and the Malaysian Ministry of Health; **ITC Malaysia** (second cohort) team at University Malaya; **ITC Mauritius** teams at Mauritius Institute of Health and ViSa; **ITC Mexico** team at the Mexican National Institute of Public Health (INSP); **ITC Netherlands (first cohort)** teams at Maastricht University, University of Amsterdam, and Leiden University Medical Centre); **ITC Netherlands (second cohort)** teams at Maastricht University and University of Amsterdam (ASCoR); **ITC New Zealand** (first and second cohorts) team at University of Otago; **ITC Thailand** team at the Institute for Population and Social Research (IPSR), Mahidol University Salaya; **ITC Uruguay** team at the Departamento de Sociología, the Facultad de Ciencias Sociales of the Universidad de la República de Uruguay and the Sindicato Médico del Uruguay**; ITC Zambia** team at University of Zambia School of Medicine; **ITC EUREST-PLUS Spain** team at Tobacco Control Unit, Catalan Institute of Oncology (ICO) and Bellvitge Biomedical Research Institute (IDIBELL); and **the EUREST-PLUS (ITC 6 European Country)** consortium

members: European Network on Smoking and Tobacco Prevention (ENSP), Belgium: Constantine I. Vardavas, Andrea Glahn, Christina N. Kyriakos, Dominick Nguyen, Katerina Nikitara, Cornel Radu-Loghin and Polina Starchenko; University of Crete (UOC), **Greece**: Aristidis Tsatsakis, Charis Girvalaki, Chryssi Igoumenaki, Sophia Papadakis, Aikaterini Papathanasaki, Manolis Tzatzarakis and Alexander I. Vardavas; Kantar Public, Belgium: Nicolas Bécuwe, Lavinia Deaconu, Sophie Goudet, Christopher Hanley and Oscar Rivière; Smoking or Health Hungarian Foundation (SHHF), **Hungary**: Tibor Demjén, Judit Kiss and Anna Piroska Kovacs; Tobacco Control Unit, Catalan Institute of Oncology (ICO) and Bellvitge Biomedical Research Institute (IDIBELL), Catalonia, **Spain**: Esteve Fernández, Yolanda Castellano, Marcela Fu, Sarah O. Nogueira and Olena Tigova; Kings College London (KCL), **United Kingdom**: Ann McNeill, Katherine East and Sara C. Hitchman; Cancer Prevention Unit and WHO Collaborating Centre for Tobacco Control, German Cancer Research Center (DKFZ), **Germany** (second cohort): Ute Mons and Sarah Kahnert; National and Kapodistrian University of Athens (UoA), **Greece**: Yannis Tountas, Panagiotis Behrakis, Filippos T. Filippidis, Christina Gratziou, Paraskevi Katsaounou, Theodosia Peleki, Ioanna Petroulia and Chara Tzavara; Aer Pur Romania, **Romania**: Antigona Carmen Trofor, Marius Eremia, Lucia Lotrean and Florin Mihaltan; European Respiratory Society (ERS), Switzerland: Gernot Rohde, Tamaki Asano, Claudia Cichon, Amy Far, Céline Genton, Melanie Jessner, Linnea Hedman, Christer Janson, Ann Lindberg, Beth Maguire, Sofia Ravara, Valérie Vaccaro and Brian Ward; Maastricht University, the Netherlands: Marc Willemsen, Hein de Vries, Karin Hummel and Gera E. Nagelhout; Health Promotion Foundation (HPF), **Poland**: Witold A. Zatoński, Aleksandra Herbeć, Kinga Janik-Koncewicz, Krzysztof Przewoźniak and Mateusz Zatoński. University of Waterloo (UW), Canada: Geoffrey T. Fong, Thomas K. Agar, Pete Driezen, Shannon Gravely, Anne C. K. Quah and Mary E. Thompson.

ITC Project's Data Availability Statement: In each country participating in the international Tobacco Control Policy Evaluation (ITC) Project, the data are jointly owned by the lead researcher(s) in that country and the ITC Project at the University of Waterloo. Data from the ITC Project are available to approved researchers 2 years after the date of issuance of cleaned data sets by the ITC Data Management Centre. Researchers interested in using ITC data are required to apply for approval by submitting an International Tobacco Control Data Repository (ITCDR) request application and subsequently to sign an ITCDR Data Usage Agreement. The criteria for data usage approval and the contents of the Data Usage Agreement are described online (http://www.itcproject.org).

Supplementary file 1B. GATS funding statements

Financial support for GATS was provided by the CDC Foundation with grants from Bloomberg Philanthropies and the Bill and Melinda Gates Foundation. Details are provided in Supplemental Table 1.

Supplemental Table 1. Global Adult Tobacco Survey (GATS) donor funding in 32 countries included in analysis, 2008-2020.

Country	Wave	Year	GATS Funding
Argentina	1	2012	Bloomberg Philanthropies
Bangladesh	1	2009	Bloomberg Philanthropies
	2	2017	Bloomberg Philanthropies
Botswana	1	2017	Bill and Melinda Gates Foundation
Brazil	1	2008	56 % Bloomberg Philanthropies<br>44% In-country funding
Cameroon	1	2013	Bill and Melinda Gates Foundation
China	1	2010	Bill and Melinda Gates Foundation
	2	2018	69% Bloomberg Philanthropies<br>31% In-country funding
Costa Rica	1	2015	In-country funding
Egypt	1	2009	Bloomberg Philanthropies
Ethiopia	1	2016	Bill and Melinda Gates Foundation
Greece	1	2013	In-country funding
India	1	2009	36% Bloomberg<br>67% In-country funding
	2	2017	33% Bloomberg Philanthropies<br>67% In-country funding
Indonesia	1	2011	Bloomberg Philanthropies
Kazakhstan	1	2014	Bloomberg Philanthropies
Kenya	1	2014	Bill and Melinda Gates Foundation
Malaysia	1	2011	60% Bloomberg Philanthropies<br>40% In-country funding
Mexico	1	2009	Bloomberg Philanthropies
	2	2015	76% Bloomberg Philanthropies<br>24% In-country funding
Nigeria	1	2012	Bloomberg Philanthropies
Pakistan	1	2014	Bloomberg Philanthropies
Panama	1	2013	In-country funding
Philippines	1	2009	Bloomberg Philanthropies
	2	2015	In- country funding
Poland	1	2009	Bloomberg Philanthropies
Qatar	1	2013	In-country funding
Romania	1	2011	Bloomberg Philanthropies
	2	2018	Bloomberg Philanthropies
Russian Federation	1	2009	Bloomberg Philanthropies
	2	2016	70% Bloomberg Philanthropies<br>30% In-country funding
Senegal	1	2015	Bill and Melinda Gates Foundation

Country	Wave	Year	GATS Funding
Thailand	1	2009	Bloomberg Philanthropies
	2	2011	73% Bloomberg Philanthropies<br>27% In-country funding
Turkey	1	2008	Bloomberg Philanthropies
	2	2012	Bloomberg Philanthropies
	3	2016	6% Bloomberg Philanthropies<br>94% In-country funding
Uganda	1	2013	Bill and Melinda Gates Foundation
Ukraine	1	2010	Bloomberg Philanthropies
	2	2017	Bloomberg Philanthropies
United Republic of Tanzania	1	2018	Bill and Melinda Gates Foundation
Uruguay	1	2009	Bloomberg Philanthropies
	2	2017	52% Bloomberg Philanthropies<br>48% In-country funding
Viet Nam	1	2010	Bloomberg Philanthropies
	2	2015	92% Bill and Melinda Gates<br>8% In-country funding

Chung-Hall J, *et al. BMJ Open* 2026; 16:e107667. doi: 10.1136/bmjopen-2025-107667
