# Supplementary material for: Changes in knowledge of the major harms of tobacco smoking and secondhand smoke exposure among adults who smoke: findings from 26 countries of the International Tobacco Control (ITC) Project (2002–2022) and 32 countries of the Global Adult Tobacco Survey (GATS) (2008–2020)
Source: BMJ Open. 2026 Aug 4;16(8):e107667. doi: 10.1136/bmjopen-2025-107667 (PMC13479502; doi:10.1136/bmjopen-2025-107667)
Supplement: Supplementary file 2 [file bmjopen-2025-107667.draft_revisions.pdf]

BMJ Open

BMJ Open is committed to open peer review. As part of this commitment we make the peer review history of every article we publish publicly available.

When an article is published we post the peer reviewers' comments and the authors' responses online. We also post the versions of the paper that were used during peer review. These are the versions that the peer review comments apply to.

The versions of the paper that follow are the versions that were submitted during the peer review process. They are not the versions of record or the final published versions. They should not be cited or distributed as the published version of this manuscript.

BMJ Open is an open access journal and the full, final, typeset and author-corrected version of record of the manuscript is available on our site with no access controls, subscription charges or pay-per-view fees (http://bmjopen.bmj.com).

If you have any questions on BMJ Open's open peer review process please email info.bmjopen@bmj.com

BMJ Open

Changes in knowledge about major harms of tobacco smoking and secondhand smoke exposure among adults who smoke: findings from 26 countries of the International Tobacco Control (ITC) Project (2002–2022) and 32 countries of the Global Adult Tobacco Survey (GATS) (2008–2020)

Journal:	*BMJ Open*
Manuscript ID	bmjopen-2025-107667
Article Type:	Original research
Date Submitted by the Author:	08-Jul-2025

Complete List of Authors:	Chung-Hall, Janet; University of Waterloo, Psychology<br>Fong, Geoffrey; University of Waterloo, Psychology, School of Public Health Sciences; Ontario Institute for Cancer Research<br>Meng, Gang; University of Waterloo<br>Craig, Lorraine; University of Waterloo, Psychology<br>Indome, Eunice; University of Waterloo<br>Kress, Alissa; National Center for Chronic Disease Prevention and Health Promotion Office on Smoking and Health<br>Shi, Jing; National Center for Chronic Disease Prevention and Health Promotion Office on Smoking and Health; CDC Foundation Inc, Noninfectious Disease Programs<br>Ahluwalia, Indu; National Center for Chronic Disease Prevention and Health Promotion Office on Smoking and Health

Keywords:	PUBLIC HEALTH, Tobacco Use, Health

SCHOLARONE™ Manuscripts

I, the Submitting Author has the right to grant and does grant on behalf of all authors of the Work (as defined in the below author licence), an exclusive licence and/or a non-exclusive licence for contributions from authors who are: i) UK Crown employees; ii) where BMJ has agreed a CC-BY licence shall apply, and/or iii) in accordance with the terms applicable for US Federal Government officers or employees acting as part of their official duties; on a worldwide, perpetual, irrevocable, royalty-free basis to BMJ Publishing Group Ltd ("BMJ") its licensees and where the relevant Journal is co-owned by BMJ to the co-owners of the Journal, to publish the Work in this journal and any other BMJ products and to exploit all rights, as set out in our licence.

The Submitting Author accepts and understands that any supply made under these terms is made by BMJ to the Submitting Author unless you are acting as an employee on behalf of your employer or a postgraduate student of an affiliated institution which is paying any applicable article publishing charge ("APC") for Open Access articles. Where the Submitting Author wishes to make the Work available on an Open Access basis (and intends to pay the relevant APC), the terms of reuse of such Open Access shall be governed by a Creative Commons licence – details of these licences and which Creative Commons licence will apply to this Work are set out in our licence referred to above.

Other than as permitted in any relevant BMJ Author's Self Archiving Policies, I confirm this Work has not been accepted for publication elsewhere, is not being considered for publication elsewhere and does not duplicate material already published. I confirm all authors consent to publication of this Work and authorise the granting of this licence.

For peer review only

Changes in knowledge about major harms of tobacco smoking and secondhand smoke exposure among adults who smoke: findings from 26 countries of the International Tobacco Control (ITC) Project (2002–2022) and 32 countries of the Global Adult Tobacco Survey (GATS) (2008–2020)

Janet Chung–Hall,[1] Geoffrey T. Fong, [1,2,3] Gang Meng,[1] Lorraine V. Craig,[1] and Eunice O. Indome[1] for the ITC Project Collaborators, and Alissa C. Kress,[4] Jing Shi,[4,5] and Indu B. Ahluwalia[4] for the Global Adult Tobacco Survey Team

Affiliation(s):
[1] Department of Psychology, University of Waterloo, Waterloo, Ontario, Canada
[2] School of Public Health Sciences, University of Waterloo, Waterloo, Ontario, Canada
[3] Ontario Institute for Cancer Research, Toronto, Ontario, Canada
[4] Office on Smoking and Health, National Center for Chronic Disease Prevention and Health Promotion, Centers for Disease Control and Prevention, Atlanta, Georgia, USA
[5] Noninfectious Disease Programs, CDC Foundation, Atlanta, Georgia, USA assigned to the Office on Smoking and Health, National Center for Chronic Disease Prevention and Health Promotion, Centers for Disease Control and Prevention

Corresponding author:
Dr. Janet Chung-Hall
University of Waterloo
Department of Psychology, ITC Project
200 University Avenue West
Waterloo, Ontario, Canada, N2L 3G1
Email: jchunghall@uwaterloo.ca

Word count: 4766

Disclaimer: The findings and conclusions in this article are those of the authors and do not necessarily represent the official positions of the U.S. Centers for Disease Control and Prevention or the CDC Foundation.

Funding Statement: The ITC Four Country Surveys (Australia, Canada, the United Kingdom, the United States) were supported by grants from the US National Cancer Institute (R01 CA100362, R01 CA090955, P50 CA111236 (Roswell Park Transdisciplinary Tobacco Use Research Center), and P01 CA138389), Robert Wood

For peer review only - http://bmjopen.bmj.com/site/about/guidelines.xhtml

Johnson Foundation (045734), the Canadian Institutes of Health Research (FDN-148477, MOP-57897, MOP-79551, MOP-115016), the Canadian Tobacco Control Research Initiative (014578), Australia Commonwealth Department of Health and Ageing, the National Health and Medical Research Council of Australia (265903, 450110, 1005922), and Cancer Research UK (C312/A3726, C312/A6465, C321/A11039, C25586/A19540). The ITC Four Country Smoking and Vaping Surveys (Australia, Canada, England, and the United States) were supported by grants from the US National Cancer Institute (P01 CA200512), the Canadian Institutes of Health Research (FDN-148477), and the National Health and Medical Research Council of Australia (APP1106451). The ITC Bangladesh Surveys were supported by grants from the US National Cancer Institute (P01 CA138389), the International Development Research Centre (IDRC Grant 104831-003), and Canadian Institutes for Health Research (MOP-79551, MOP-115016). The ITC Brazil Surveys were supported by the Instituto Nacional de Câncer (INCA, National Cancer Institute), Secretaria Nacional de Pokiticas sobre Drogas (SENAD, National Secretariat for Drug Policy), Pan-American Health Organization (PAHO), Brazil Fundação do Câncer (BR/LOA/1500048·00), and the Canadian Institutes of Health Research (MOP-115016, FDN-148477). The ITC China Surveys were supported by grants from the US National Cancer Institute at the National Institutes of Health (R01 CA125116), the Roswell Park Transdisciplinary Tobacco Use Research Center (P50 CA111236), Canadian Institutes for Health Research (MOP-79551, MOP-115016), and the Chinese Center for Disease Control and Prevention. The ITC France Surveys (first cohort) were supported by grants from the Observatoire français des drogues et toxicomanies (OFDT), Institut national de prevention et d'education pour la sante (INPES), and the Institut nationale du cancer (INCa). The ITC France Surveys (second cohort) were supported by grants from Santé publique France, the Institut nationale du cancer (INCa), and the Canadian Institutes of Health Research (FDN-148477). The ITC Germany Surveys (first cohort) were supported by grants from the German Federal Ministry of Health, Dieter Mennekes-Umweltstiftung, and Germany Cancer Research Center (DKFZ). The EUREST-PLUS Project (ITC 6 European Country Surveys) in Germany (second cohort), Greece, Hungary, Poland, Romania, and Spain has received funding from the European Union's Horizon2020 research and innovation programme under grant agreement No·681109 (C.I.V.) and the University of Waterloo (G.T.F.), and additional support was provided to the University of Waterloo by the Canadian Institutes of Health Research (FDN-148477). EF is partly supported by Ministry of Universities and Research, Government of Catalonia (2017SGR319) and by the Instituto Carlos III and co-funded by the European Regional Development Fund (FEDER) (INT16/00211 and INT17/00103), Government of Spain and thanks CERCA Programme Generalitat de Catalunya for the institutional support to IDIBELL. The EUREST-PLUS Spain Survey was supported by grants from the Instituto de Salud Carlos III (PI17/01338, Co-funded by European Regional Development Fund ERDF, a way to build Europe) and the Canadian Institute for Health Research (FDN-148477). EF and MF are partly supported by the Ministry of Universities and Research, Government of Catalonia (2021SGR00906). EF and MF are partly supported by the Ministry of Universities and Research, Government of Catalonia (2021SGR00906) and thank CERCA Programme Generalitat de Catalunya for the institutional support to IDIBELL. The ITC India Surveys were supported by grants from the US National Cancer Institute (P50 CA111236, P01 CA138389), the Canadian Institute of Health Research

For peer review only - http://bmjopen.bmj.com/site/about/guidelines.xhtml

(MOP-79551, MOP-115016, FDN-148477), and Healis Sekhsaria Institute for Public Health. The ITC Japan Surveys were supported by the Japan National Cancer Center and Research Development Fund (28-A-24) and the Canadian Institutes of Health Research (FDN-148477). The ITC Kenya Surveys were supported by grants from the Canadian Institutes of Health Research (MOP-115016, FDN-148477). The ITC Thailand Surveys were supported by grants from the US National Cancer Institute (P50 CA111236 (Roswell Park Transdisciplinary Tobacco Use Research Center), P01 CA138389), the Canadian Institutes of Health Research (MOP-79551, MOP-115016), and the ThaiHealth Promotion Foundation. The ITC Malaysia Surveys (first cohort) were supported by grants from the US National Cancer Institute (P50 CA111236 (Roswell Park Transdisciplinary Tobacco Use Research Center) and P01 CA138389), the Canadian Institutes of Health Research (MOP-79551, MOP-115016), and the Malaysian Ministry of Health. The ITC Malaysia Surveys (second cohort) were supported by the Malaysia Ministry of Higher Education-LRGS NanoMITe (RU029-2014), the University of Malaya Research University Grant (RU029C-2014 and RU001A-2021), and the Canadian Institutes of Health Research (FDN-148477). The ITC Mauritius Surveys were supported by grants from the International Development Research Centre, the Canadian Institutes of Health Research (MOP-115016), and was partially supported by the World Lung Foundation. The ITC Mexico Surveys were supported by grants from the Mexican Consejo Nacional de Ciencia y Tecnología (Salud-2007-C01-70032, Salud-2013-01-202671), Bloomberg Global Initiative—International Union Against Tuberculosis and Lung Disease (IUATLD), the US National Cancer Institute (Roswell Park Transdisciplinary Tobacco Use Research Center (P50 CA111236)) and the Canadian Institutes for Health Research (MOP-115016). The ITC Netherlands Surveys (first cohort) were supported by grants from The Netherlands Organisation for Health Research and Development (ZonMw 121010008, ZonMw 200130002), The SILNE Project (funded by the European Commission through FP7 HEALTH-F3-2011-278273) and the Dutch Cancer Foundation (KWF) (UM 2014-7210). The ITC Netherlands Surveys (second cohort) were supported by grants from Longfonds (Lung Foundation Netherlands), Hartstichting (Netherlands Heart Foundation), KWF Kankerbestrijding (Dutch Cancer Society), Trombosestichting Nederland (Thrombosis Foundation), Diabetesfonds (Diabetes Fund) and the Canadian Institutes of Health Research (FDN-148477). The ITC New Zealand Surveys (first cohort) were supported by grants from the New Zealand Health Research Council (06/453), the New Zealand Ministry of Health, University of Otago, the US National Cancer Institute ((P50 CA111236) Roswell Park Transdisciplinary Tobacco Use Research Centre), and the Canadian Institutes of Health Research (MOP-79551). The ITC New Zealand (EASE: Evidence for Achieving Smokefree 2025 Equitably) Surveys (second cohort) were supported by grants from the New Zealand Health Research Council (GA215F 15/072, HRC 19/641), the New Zealand Ministry of Health, and the Canadian Institute for Health Research (FDN-148477). The ITC Korea Surveys (first cohort) were supported by grants from the National Cancer Center of Korea (0731040-1), the Korean Ministry of Health and Welfare, and the US National Cancer Institute (P50 CA111236, R01 CA125116) and the Canadian Institutes of Health Research (MOP-79551). The ITC Korea Survey (second cohort) was supported by a grant from the Korean Ministry of Health and Welfare and the Canadian Institutes of Health Research (MOP-115016). The ITC Korea Surveys (third cohort) were supported by a grant from the Korean Ministry of Health and Welfare and the Canadian

For peer review only - http://bmjopen.bmj.com/site/about/guidelines.xhtml

Institutes of Health Research (FDN-148477). The ITC Uruguay Surveys were supported by grants from the US National Cancer Institute (P50 CA111236 (Roswell Park Transdisciplinary Tobacco Use Research Center), P01 CA138389), and the Canadian Institutes of Health Research (MOP-115016). The ITC Zambia Surveys were supported by a grant from the Canadian Institutes of Health Research (MOP-115016). Additional support to GTF is provided by a Senior Investigator Grant from the Ontario Institute for Cancer Research (2002-present), and a Senior Prevention Scientist Award from the Canadian Cancer Society Research Institute (2002-2009) and O. Harold Warwick Prize (2021). Financial support for GATS was provided by the CDC Foundation with grants from Bloomberg Philanthropies and the Bill & Melinda Gates Foundation. The funders had no role in the design of the study; in the collection, analyses, or interpretation of data; in the writing of the manuscript, or in the decision to publish the results.

Competing Interests: All authors have no competing interests to declare.

Patient and Public Involvement: Patients or the public were not involved in the design, or conduct, or reporting, or dissemination plans of our research.

For peer review only

ABSTRACT

Objectives. To assess levels of and trends in knowledge that smoking and secondhand smoke (SHS) causes lung cancer and cardiovascular disease (CVD) in 46 countries over two decades.

Design. Longitudinal cohort and repeat cross-sectional surveys.

Setting. Nationally representative surveys in 26 International Tobacco Control (ITC) countries (2002-2022), and 32 Global Adult Tobacco Survey (GATS) countries (2008-2020).

Participants. Adults (18+ years) who smoke tobacco (26 ITC countries: N=133,410; 32 GATS countries: N=110,537).

Primary and secondary outcomes. Current use of cigarettes at least monthly (all ITC countries) and/or current use of bidis at least monthly (ITC India, Bangladesh, and Zambia). Current use of any combustible tobacco products (all GATS countries). Generalized estimating equations and weighted multinomial regression models assessed country-level prevalence of knowledge for three smoking and two SHS outcomes. Multilevel models estimated all country mean prevalence and tested within country linear trends over time for five disease indicators by survey. Across 12 countries, with data for both surveys, correlations between ITC and GATS assessed consistency across indicators.

Results. Knowledge that smoking causes lung cancer was highest (ITC=92·2%, GATS=91·9%), followed by heart disease (ITC=84·7%)/heart attack (GATS=80·0%), and stroke (ITC=75·0%, GATS=66·1%). Knowledge that SHS causes lung cancer (ITC=77·3%, GATS=82·1%) was highest, followed by heart attack (ITC=61·8%)/heart

For peer review only - http://bmjopen.bmj.com/site/about/guidelines.xhtml

disease (GATS=72·5%) (all p<·0001). In ITC and GATS countries, average change in knowledge across five diseases was +0·14% per year. There was agreement between ITC and GATS data (range rs= ·60–·81 across five indicators).

Conclusions. There is a need to improve and sustain people's knowledge about diseases caused by smoking and SHS, especially CVD risks.

For peer review only

For peer review only - http://bmjopen.bmj.com/site/about/guidelines.xhtml

STRENGTHS AND LIMITATIONS OF THIS STUDY

- This study analyzed nationally representative data from two international surveys ─ the International Tobacco Control (ITC) Project and the Global Adult Tobacco Survey (GATS) ─ to assess levels of and trends in knowledge about lung cancer and cardiovascular disease in 46 countries over two decades.
- Significant positive correlations between ITC and GATS data, despite differences in research and methodological designs, strengthen the validity of findings and support more confident inferences.
- Use of self-reported data may be subject to measurement bias.
- Generalizability of findings is limited by low response rates for some ITC surveys and the restriction of ITC-GATS correlation analyses to 12 countries.

For peer review only

For peer review only - http://bmjopen.bmj.com/site/about/guidelines.xhtml

INTRODUCTION

Over 8 million people die from tobacco-caused diseases per year; tobacco smoking is the single most preventable cause of death globally.[1] Knowledge about the harms of smoking is positively associated with quit intentions,[2] attempts,[3] and cessation success.[4] People who smoke and are unaware of the health risks of secondhand smoke exposure (SHS) may be less likely to protect themselves or others around them for SHS.[5]

The WHO Framework Convention on Tobacco Control (FCTC), entered into force in 2005, calls upon Parties (183 to date) to implement evidence-based measures to reduce tobacco use.[6] The WHO MPOWER[a] package includes imp lementation of six evidence-based measures at country-level.[7] The W (warning about the dangers of tobacco) strategies include the display of pictorial health warnings on 75% of the surface of cigarette packs [8,9] and mass media campaigns to increase knowledge about tobacco-caused health risks.[5,10]

Despite efforts to inform people about the harms of smoking and SHS, gaps in knowledge remain. Studies have found that adults in general,[11] and those who use cigarettes[12–15] and other smoked tobacco products,[15] report higher knowledge that smoking causes lung cancer compared with heart disease and/or stroke.[b] They are also are more likely to know that SHS exposure causes lung cancer as compared with heart disease. [14,15]

[a] The six MPOWER measures are Monitor tobacco use and policies; Protect people from tobacco smoke; Offer to help quit tobacco use; Warn about dangers of tobacco; Enforce bans on tobacco advertising, promotion, and sponsorship; and Raise tobacco taxes.

[b] The 2023 World Health Organization International Classification of Diseases 11th Revision (ICD–11) defines heart disease and heart attack as circulatory system diseases, and stroke as a cerebrovascular disease. In this paper, the term CVD will be used to refer to cardiovascular (coronary heart disease, myocardial infarction) and/or cerebrovascular disease (stroke) to allow for more readable text.

For peer review only - http://bmjopen.bmj.com/site/about/guidelines.xhtml

Few studies have examined changes in knowledge about smoking- and SHS-caused diseases. A repeated cross-sectional study found significant increases from 1999 to 2006 in risk perceptions of SHS for lung cancer and heart disease among the general adult population.[16] One Global Adult Tobacco Survey (GATS) study in six countries between 2008 and 2016, found significant increases in knowledge that smoking causes lung cancer, heart attack, and stroke among persons aged 15+ years who smoke.[15]

This study assessed knowledge over two decades about key diseases caused by smoking tobacco and SHS exposure among adults who smoke across 46 countries. Indicators examined included knowledge that smoking causes lung cancer, heart disease/heart attack, and stroke in people who smoke, and that SHS causes lung cancer and heart disease/heart attack in people who do not smoke. We examined change over time in knowledge for each indicator from 2002 to 2022 (International Tobacco Control Policy Evaluation Project countries; ITC) and from 2008 to 2020 (GATS countries). We examined correlation of knowledge items across 12 countries with data from both surveys.

METHODS

Study design and participants

Data were from the ITC Project and the Global Tobacco Surveillance System (GTSS) GATS.

ITC Project longitudinal cohort surveys with replenishment are administered in 31 countries using a standardised set of questions, data collection protocol, and sampling design that allows for cross-country comparisons. For this study, we used ITC Project

For peer review only - http://bmjopen.bmj.com/site/about/guidelines.xhtml

Survey data from nationally representative samples of adults (aged 18+ years) who smoke tobacco in 26 countries between 2002 and 2022: Australia, Bangladesh, Brazil, Canada, China, England/United Kingdom (UK)[c], France, Germany, Greece, Hungary, India, Japan, Kenya, Malaysia, Mauritius, Mexico, the Netherlands, New Zealand, Poland, Republic of Korea, Romania, Spain, Thailand, the US, Uruguay, and Zambia (Supplementary table 1). Adults who smoke tobacco are defined as persons aged 18+ years who smoke tobacco, including those who currently smoke manufactured and/or roll-your own cigarettes at least monthly and had smoked at least 100 cigarettes in their lifetime (all countries), and/or currently smoke bidis at least monthly (India, Bangladesh, and Zambia).

The GTSS is the global standardized surveillance system for systematic cross-sectional monitoring of tobacco use and tracking of key tobacco control indicators in multiple countries.[17] For this study, we used GATS data. GATS is a nationally representative repeated cross-sectional household survey of persons aged 15+ years that uses a standard core questionnaire, sample design, and data collection methods. GATS data included in this study are from adults who smoke tobacco in 32 countries between 2008 and 2020: Argentina, Bangladesh, Botswana, Brazil, Cameroon, China, Costa Rica, Egypt, Ethiopia, Greece, India, Indonesia, Kazakhstan, Kenya, Malaysia, Mexico, Nigeria, Pakistan, Panama, Philippines, Poland, Qatar, Romania, Russian Federation, Senegal, Tanzania, Thailand, Turkey, Uganda, Ukraine, Uruguay, and Viet

[c] Caution is warranted in the comparison of ITC Survey data from the United Kingdom (which includes England, Wales, Scotland, and Northern Ireland;2002 to 2010–11) and England (2020). However, England accounts for ~85% of the population of the United Kingdom, and smoking prevalence and tobacco control policies are similar across all four countries that comprise the United Kingdom. As such, we combined data from the United Kingdom and England for analysis, as approved by ITC Project Principal Investigators.

For peer review only - http://bmjopen.bmj.com/site/about/guidelines.xhtml

Nam (Supplementary table 2). For consistency in analysis, an age cut-off of 18+ years was used to define adults who smoke combustible tobacco products (bidis, kreteks, cheroots, manufactured cigarettes, hand-rolled cigarettes, cigars, cigarillos, pipes, waterpipe with tobacco).

Data on knowledge of smoking- and SHS-caused diseases were collected from adults who smoked tobacco and participated in at least one wave of the ITC Surveys (N=133,410) and/or the GATS (N=110,537). We reported the level of knowledge at the most recent survey wave. We used data from all survey waves to test for significant yearly change in knowledge, and data from the first and last survey waves to compute average yearly change in knowledge. Countries included in the ITC Surveys and/or GATS are listed in Supplementary table 3. Sample demographics and methodological details for ITC Surveys are described elsewhere[18] and in online individual country technical reports.[19] Methodological details for the GATS and individual country fact sheets are available online.[20]

This study includes publicly available data: 31 ITC countries (ITC Project Principal Investigators' approval on July 28, 2023 for use of 2002 to 2022 ITC Survey data), and 32 GATS countries (US Centers for Disease Control and Prevention Office on Smoking and Health approval on May 1, 2023 for use of 2008 to 2020 GATS data). Data from ITC and GATS countries that became publicly available after these dates were excluded from analyses.

Measures

Knowledge about smoking-caused diseases was assessed by asking participants if they know or believe that smoking causes lung cancer, heart disease/heart attack, and

For peer review only - http://bmjopen.bmj.com/site/about/guidelines.xhtml

stroke. Respondents were also asked if they know or believe that SHS causes lung cancer and heart disease/heart attack in people who do not smoke. Responses were coded as 'yes' vs. 'no/don't know'. Response 'refused' was excluded from the analysis. ITC Survey and GATS questions and response options are presented in Supplementary table 4. Questions for all five disease outcomes were not included at every survey wave across all 26 ITC and 32 GATS countries. We used the most recent data available for each country to calculate point estimates and all-country means of the prevalence of knowledge for each disease outcome, and all longitudinal data available for each country to assess changes in knowledge over time.

Statistical analysis

Point estimates of the prevalence of knowledge for each disease outcome by country were calculated using weighted multinomial survey logistic regression models for pooled repeated cross-sectional GATS data, and generalized estimating equations for pooled longitudinal ITC data. All models incorporated survey design information (strata and primary sampling units for multi-stage sampling surveys), adjusted for sex, age, smoking status (daily, non-daily), and time-in-sample (ITC countries).

Point estimates of all-country means of the prevalence of knowledge for each disease outcome across all ITC countries and all GATS countries, respectively, were calculated using a multi-level model (two levels: individual and country) with random intercepts at the country-level and with roughly equal weights for each country. These point estimates cannot be used to make inferences to the global population. As such, we report the estimated all-country means of knowledge for each health effect without the associated confidence intervals, which would refer to a hypothetical superpopulation

For peer review only - http://bmjopen.bmj.com/site/about/guidelines.xhtml

of countries. The all-country mean estimates in this analysis therefore only serve as the central point of selected countries. GEE and survey logistic regression models were used to test statistical differences in comparing knowledge between the five health effects among respondents in ITC and GATS countries, respectively.

We estimated changes in knowledge for each outcome among adults who smoke in countries with at least two waves of data between 2002 and 2022 (ITC Project Surveys), and between 2008 and 2020 (GATS). Analysis of the longitudinal ITC data used generalized estimating equations to account for within-subject correlations given that a proportion of individuals participated in more than one survey. Analysis of the repeated cross-sectional GATS data used weighted multinomial survey logistic regression models. Two-level models (individual and country) with random intercepts and random slopes of survey years at the country-level were used to test for significant yearly change in knowledge for each health effect within countries. This modelling approach controls for the number of years between the first and last survey waves, and assumed a linear survey year effect within each country in the absence of a compelling theoretical relationship between survey year and country.

Data from ITC and GATS countries were pooled and fitted separately to ensure comparability within ITC countries and within GATS countries. Pearson correlations (r) between ITC and GATS mean prevalence estimates of knowledge for each of the five diseases were used to examine consistency of estimates from different surveys conducted in the 12 overlapping countries.

The predicted marginal standardization method (PREDMARG) was used to generate all marginal estimates of the outcomes. Data analysis were conducted using

For peer review only - http://bmjopen.bmj.com/site/about/guidelines.xhtml

SAS v9.4 and SAS-callable SUDAAN v11.0.3. Statistical significance ($p < .05$ considered statistically significant) and confidence intervals were computed at the 95% confidence level.

RESULTS

Knowledge about diseases caused by smoking and SHS exposure

Knowledge about diseases caused by smoking among adults who smoke was highest for lung cancer (ITC=92.2%, GATS=91.9%), followed by heart disease (ITC=84.7%)/heart attack (GATS=80.0%), and stroke (ITC=75.0%, GATS=66.1%) (Supplementary table 5). Knowledge that SHS causes lung cancer among people who do not smoke (ITC=77.3%, GATS=82.1%) was higher than for SHS causes heart attack (ITC=61.8%)/heart disease (GATS=72.5%) in people who do not smoke. All differences between knowledge about the five diseases caused by smoking and SHS exposure were significant at $p < .001$.

Knowledge that active smoking causes lung cancer, heart disease/heart attack, and stroke

At the most recent survey across 25 ITC countries, 92.2% of adults who smoke overall knew that smoking causes lung cancer, ranging from 98.2% in Bangladesh (2014–15) to 78.1% in Zambia (2014) (Figure 1A). Across all ITC countries, knowledge that smoking causes lung cancer decreased by 0.7 percentage points (PP) per year. Knowledge increased significantly in China (+1.2 PP per year, 71.5% in 2006 to 80.9% in 2013–15, $p<.01$), the Netherlands (+0.9 PP per year, 81.4% in 2011 to 91.8% in 2022, $p<.001$), and Bangladesh (+0.6 PP per year, 94.5% in 2009 to 98.1% in 2014-15, $p<.001$). Knowledge decreased significantly in Japan (-2.0 PP per year, 86.2% in 2018

For peer review only - http://bmjopen.bmj.com/site/about/guidelines.xhtml

to 80.3% in 2021, p<.01), Germany (-0.6 PP per year, 97.8% in 2007 to 91.5% in 2018, p<.001), and France (-0.5 PP per year, 99.1% in 2006-07 to 93.1% in 2019, p<.001) (Figure 1A). There was no significant change in knowledge in all other countries.

At the most recent survey across 32 GATS countries, 91.9% of adults who smoke overall knew that smoking causes lung cancer, ranging from 98.4% in Argentina (2012) to 56.6% in Nigeria (2012) (Figure 1B). Across the 12 GATS countries with at least two years of data, knowledge that smoking causes lung cancer decreased by 0.2 PP per year. Knowledge increased significantly in India (+0.8 PP per year, 86.2% in 2009 to 91.9% in 2016, p<.05), and Bangladesh (+0.5 PP per year, 93.0% in 2008 to 97.1% in 2017, p<.05). Knowledge decreased significantly in Romania (–1.3 PP per year, 96.8% in 2011 to 87.5% in 2018, p<.001), the Russian Federation (–1.2 PP per year, 96.0% in 2009 to 87.8% in 2016, p<.001), and Turkey (–0.4 PP per year, 98.7% in 2008 to 95.3% in 2016, p<.01) (Figure 1B). There was no significant change in knowledge in all other countries. For the 20 GATS countries with only one year of data, the change is presented as zero.

At the most recent survey across 25 ITC countries, 84.7% of adults who smoke knew that smoking causes heart disease, ranging from 94.9% in Bangladesh (2014–15) to 57.7% in China (2013–15) (Figure 2A). Across all ITC countries, knowledge that smoking causes heart disease decreased by 0.3 PP per year. Knowledge increased significantly in China (+2.2 PP per year, 38.0% in 2006 to 57.0% in 2013–15, p<.05), Kenya (+2.2 PP per year, 65.7% in 2012 to 78.9% in 2018, p<.05), India (+1.2 PP per year, 75.5% in 2010-11 to 85.4% in 2018-19, p<.05), Bangladesh (+1.2 PP per year, 88.1% in 2009 to 94.5% in 2014-15, p<.001), and Spain (+1.1 PP per year, 82.8% in

For peer review only - http://bmjopen.bmj.com/site/about/guidelines.xhtml

2016 to 88.4% in 2021, p<.05). Knowledge decreased significantly in Japan (–4.3 PP per year, 77.1% in 2018 to 64.2% in 2021, p<.01), Germany (–1.4 PP per year, 95.6% in 2007 to 80.1% in 2018, p<.001), Uruguay (–0.7 PP per year, 95.3% in 2006 to 89.4% in 2014, p<.05), and France (–0.5 PP per year, 97.1% in 2006–07 to 90.3% in 2019, p<.01) (Figure 2B). There was no significant change in knowledge in all other countries.

At the most recent survey across 32 GATS countries, 80.0% of adults who smoke knew that smoking causes heart attack, ranging from 95.3% in Turkey (2016) to 46.4% in China (2018) (Figure 2B). Across all GATS countries with at least two years of data, knowledge that smoking causes heart attack increased by 0.5 PP per year. Knowledge increased significantly in Viet Nam (+2.2 PP per year, 58.1% in 2010 to 68.6% in 2015, p<.01), India (+1.9 PP per year, 62.3% in 2009 to 75.3% in 2016, p<.001), the Philippines (+1.0 PP per year, 76.6% in 2009 to 82.4% in 2015, p<.05), and Bangladesh (+0.6 PP per year, 88.9% in 2009 to 93.5% in 2017, p<.01. Knowledge decreased significantly in Romania (–2.1 PP per year, 87.0% in 2011 to 72.4% in 2018, p<.001) (Figure 2B). There was no significant change in knowledge in all other countries.

At the most recent survey across 25 ITC countries, 75.0% of adults who smoke knew that smoking causes stroke, ranging from 93.9% in Bangladesh (2014–15) to 37.3% in China (2013–15) (Figure 3A). Across all ITC countries, knowledge that smoking causes stroke increased by 1.6 PP per year. Knowledge increased significantly in Thailand (+5.7 PP per year, 44.0% in 2005 to 83.9% in 2012, p<.001), Greece (+5.5 PP per year, 68.8% in 2016 to 79.8% in 2018, p<.01), Mauritius (+4.6 PP per year, 66.0% in 2009 to 75.3% in 2011, p<.01), Hungary (+4.5 PP per year, 56.0% in 2016 to 64.9% in 2018, p<.05), Kenya (+3.9 PP per year, 44.7% in 2012 to 68.2% in 2018,

For peer review only - http://bmjopen.bmj.com/site/about/guidelines.xhtml

p<.001), Uruguay (+2.8 PP per year, 43.0% in 2006 to 65.1% in 2014, p<.001), China (+2.5 PP per year, 16.2% in 2006 to 36.5% in 2013-15, p<.001), Mexico (+2.2 PP per year, 55.9% in 2006 to 74.8% in 2014-15, p<.01), Republic of Korea (+2.1 PP per year, 37.0% in 2005 to 71.0% in 2021, p<.001), the Netherlands (+1.4 PP per year, 55.6% in 2011 to 70.7% in 2022, p<.001), and India (+1.3 PP per year, 62.9% in 2010-11 to 73.6% in 2018-19, p<.01). Knowledge decreased significantly in Germany (–1.0 PP per year, (88.9% in 2007 to 78.3% in 2018, p<.001) (Figure 3A). There was no significant change in knowledge in all other countries.

At the most recent survey across 32 GATS countries, 66.1% of adults who smoke overall knew that smoking causes stroke, ranging from 94.1% in Bangladesh (2017) to 29.6% in Ethiopia (2016) (Figure 3B). Across all GATS countries with at least two years of data, knowledge that smoking causes stroke increased by 0.5 PP per year. Knowledge increased significantly in India (+2.3 PP per year, 47.4% in 2009 to 63.1% in 2016, p<.001), China (+1.3 PP per year, 26.9% in 2010 to 37.4% in 2018, p<.01, the Russian Federation (+1.2 PP per year, 63.5% in 2009 to 72.0% in 2016, p<.01), and Mexico (+1.2 PP per year, 60.6% in 2009 to 68.1 in 2015, p<.05), and Bangladesh (+1.0 PP per year, 85.9% in 2009 to 94.1% in 2017, p<.001). Knowledge decreased significantly in Romania (–2.0 PP per year, 87.2% in 2011 to 73.2% in 2018, p<.001) (Figure 3B). There was no significant change in knowledge in all other countries.

Knowledge about the risks of SHS exposure causing lung cancer and heart disease/heart attack

At the most recent survey across 26 ITC countries, 77.3% of adults who smoke knew that SHS causes lung cancer in people who do not smoke, ranging from 96.8% in

For peer review only - http://bmjopen.bmj.com/site/about/guidelines.xhtml

Thailand (2012) to 58.4% in Germany (2018) (Figure 4A). Across all ITC countries, knowledge that SHS causes lung cancer in people who do not smoke decreased by 0.1 PP per year. Knowledge increased significantly in Australia (+2.2 PP per year, 60.6% in 2003 to 72.5% in 2008-09, p<.01), China (+2.0 PP per year, 56.9% in 2006 to 73.0% in 2013-15, p<.001), India (+1.7 PP per year, 74.8% in 2010-11 to 88.4% in 2018-19, p<.001), Bangladesh (+1.4% PP per year, 87.1% in 2009 to 94.9% in 2014-15, p<.001), and Thailand (+0.8 PP per year, 91.1% in 2005 to 96.8% in 2012, p<.001). Knowledge decreased significantly in Germany (–2.0 PP per year, 80.6% in 2007 to 58.2% in 2018, p<.001), New Zealand (–1.5 PP per year, 80.8% in 2007-08 to 61.2% in 2020-21, p<.05), France (–1.5 PP per year, 94.0% in 2006-07 to 75.7% in 2019, p<.001), and Uruguay (–1.2 PP per year, 89.3% in 2006 to 79.5% in 2014, p<.05) (Figure 4A). There was no significant change in knowledge in all other countries.

At the most recent survey across 18 GATS countries, 82.1% of adults who smoke knew that SHS causes lung cancer in people who do not smoke, ranging from 96.4% in Thailand (2011) to 41.9% in Nigeria (2012) (Figure 4B). Across all GATS countries with at least two years of data, there was no change in knowledge that SHS causes lung cancer in people who do not smoke. Knowledge increased significantly in Viet Nam (+2.7 PP per year, 71.6% in 2010 to 85.1% in 2015, p<.05) (Figure 4B). There was no significant change in knowledge in all other countries.

In the most recent survey administered across 24 ITC countries, 61.8% of adults who smoke knew that SHS causes heart attack in people who do not smoke, ranging from 88.5% in Bangladesh (2014–15) to 38.4% in Australia (2013) (Figure 5A). Across all ITC countries, knowledge that SHS causes heart attack in people who do not smoke

For peer review only - http://bmjopen.bmj.com/site/about/guidelines.xhtml

increased by 0.2 PP per year. Knowledge increased significantly in Bangladesh (+7.0 PP per year, 56.9% in 2010 to 88.4% in 2014–15, p<.001), India (+2.7 PP per year, 65.0% 2010-11 to 86.9% in 2018-19, p<.001), the Netherlands (+1.6 PP per year, 38.1% in 2012 to 53.8% in 2022, p<.05), Kenya (+2.2 PP per year, 50.9% in 2012 to 63.9% in 2018, p<.05), and China (+2.1 PP per year, 43.3% in 2009 to 53.7% in 2013-15, p<.05). Knowledge decreased significantly in Australia (–3.9 PP per year, 49.1% in 2010–11 to 39.3% in 2013, p<.05), Malaysia (–3.3 PP per year, 82.5% in 2013-14 to 62.6% in 2020, p<.001), Japan (–2.7 PP per year, 64.3% in 2018 to 56.2% in 2021), and New Zealand (–2.5 PP per year, 58.0% in 2016-17 to 47.8% in 2020-21, p<.05) (Figure 5A). There was no significant change in knowledge in all other countries.

At the most recent survey across 18 GATS countries, 72.5% of adults who smoke knew that SHS causes heart disease in people who do not smoke, ranging from 93.5% in Bangladesh (2017) to 21.3% in Viet Nam (2015) (Figure 5B). Across all GATS countries with at least two years of data, knowledge that SHS causes heart disease in people who do not smoke decreased by 0.1 PP per year. Knowledge increased significantly in Viet Nam (+1.4 PP per year, 14.6% in 2010 to 21.3% in 2015, p<.05) (Figure 5B). There was no significant change in knowledge in all other countries.

Comparison of knowledge levels among respondents in ITC and GATS countries

Compared with overall knowledge that smoking causes lung cancer (ITC=92.2%, GATS=91.9%), knowledge that smoking causes heart attack (ITC=84.7%)/heart disease (GATS=80.0%) and stroke (ITC=75.0%, GATS=66.1%) was lower, as was knowledge about SHS harms for people who do not smoke (SHS causes lung cancer: ITC=77.3%,

For peer review only - http://bmjopen.bmj.com/site/about/guidelines.xhtml

GATS=82.1%; SHS causes heart attack (ITC=61.8%)/heart disease (GATS=72.5%) (all p<0.0001) (Supplementary table 5).

Knowledge that smoking causes stroke was the only one of the five harms where there was an increase in both ITC and GATS over time (+1.6 PP in ITC; +0.5 PP in GATS). Knowledge that smoking causes lung cancer was the only health harm where there was a decrease in both ITC and GATS (–0.7 PP in ITC; –0.2 PP in GATS). The average of estimated net change in knowledge across the five health harms was +0.14 PP in both ITC and GATS (Supplementary table 6).

Correlation of ITC and GATS data

In the 12 overlapping ITC and GATS countries, significant positive correlations were found between ITC and GATS estimates of knowledge among adults who smoke that smoking causes lung cancer (r=.80, p=.002), heart disease/heart attack (r=.81, p=.001), and stroke (r=.80, p=.002). For the seven overlapping ITC and GATS countries where knowledge of SHS harms was assessed, moderate but non-significant correlations were found between ITC and GATS estimates of knowledge that SHS causes lung cancer (r=.69, p=.09) and heart disease/heart attack (r=.60, p=.16) in people who do not smoke.

DISCUSSION

Overall there was a strong correlation between ITC and GATS knowledge estimates in countries where both surveys were conducted. There were five key findings from our analyses about knowledge of smoking- and SHS-caused diseases in 46 countries across two decades. First, adults who smoke were more likely to know that smoking elevates their risk for lung cancer compared with heart disease/heart attack

For peer review only - http://bmjopen.bmj.com/site/about/guidelines.xhtml

and stroke. Second, adults who smoke were less likely to know about the risks related to SHS. Third, adults who smoke had higher knowledge that SHS causes lung cancer compared with heart disease/heart attack in people who do not smoke. Fourth, knowledge for smoking- and SHS-caused diseases has improved in several countries over time; however, knowledge has generally remained unchanged in most countries and has regressed in others. Finally, there was a near-zero net change over time in knowledge about smoking- and SHS-caused diseases (+0.14% per year across five disease outcomes in ITC and GATS countries).

Consistent with previous studies [12,14,15], we found that adults who smoke were more likely to know that smoking causes lung cancer than heart disease and stroke. CVD is still the leading preventable cause of mortality worldwide [21,22] and while the relative risk from smoking for CVD is lower than for lung cancer, more people die prematurely from CVD than from lung cancer caused by smoking.[22,23] Gaps in knowledge about smoking and SHS exposure on cardiovascular health among people who smoke needs to be addressed through public health campaigns.

SHS is one of the leading causes of preventable death [24], killing an estimated 1.3 million people who do not smoke in 2021. [25] We found that across 40 countries about 65.0% of adults who smoke knew that SHS exposure causes heart disease/heart attack in people who do not smoke. It is notable that even in several high-income ITC countries, including Australia, Germany, England, the US, Aotearoa/New Zealand, Canada and Hungary, less than half of adults who smoke (38.4–49.9%) knew that SHS causes heart attack in people who do not smoke at the most recent survey, in contrast

For peer review only - http://bmjopen.bmj.com/site/about/guidelines.xhtml

with knowledge that active smoking causes heart disease (70.2–92.3%) and stroke (65.3–85.7%).

Knowledge that smoking causes stroke significantly increased in 11 ITC countries and three GATS countries between 2002 and 2022. Nevertheless, there were no significant changes in knowledge about stroke in 13 ITC countries and seven GATS countries. Likewise, there were no significant changes in knowledge among adults who smoke that smoking causes heart disease/heart attack in people who smoke, and that SHS exposure causes heart disease/heart attack in people who do not smoke in 14 ITC countries and 5 GATS countries, respectively. While significant gains in knowledge about the CVD risks of smoking tobacco and/or SHS exposure were observed in several LMICS such as Bangladesh, India, Thailand, and Viet Nam, knowledge levels have remained low in others such as Nigeria and Ethiopia. Significant declines in knowledge about some of these risks were also observed in several high-income countries, where baseline risk knowledge was relatively high, including Germany and Japan. This suggests that measures to educate people about the diseases caused by smoking may have a greater impact in countries where knowledge is low compared with those where knowledge is already high. It is key for countries to tailor interventions to address knowledge gaps.

Country-level differences observed may reflect differences in public health education strategies and implementation methods about the harms of smoking. For example, health warnings on cigarette packs, size of warnings, and languages used are one way of informing people about diseases caused by smoking, which may encourage those who smoke to reduce consumption or take steps to quit.[26–28]

For peer review only - http://bmjopen.bmj.com/site/about/guidelines.xhtml

Since the WHO FCTC came into force in 2005, countries around the world have made significant progress in educating their populations about the dangers of tobacco use and SHS exposure through the implementation of tobacco advertising, promotion and sponsorship bans,[29] health warnings on tobacco packaging,[30] smoke-free laws,[31] and anti-tobacco campaigns,[30] which have likely contributed to reductions in the tobacco use prevalence from 2000 to 2020.[32] However, we found no overall change (+0.14% per year) in knowledge across five smoking and SHS-caused diseases in both ITC and GATS countries over a two-decade period, even though 43 of the 46 countries included in the present study are WHO FCTC Parties.[33] This may reflect an uneven pace in the implementation of MPOWER measures—including taxation, health warnings on cigarette packs and mass media campaigns that are especially effective for communicating smoking-caused risks.[31] Comprehensive implementation of MPOWER measures, to accelerate reductions in smoking prevalence and prevent any levelling off over time, are needed.

This study has limitations. First, self-reported data may be subject to measurement bias that might result in over- or under-estimation of knowledge or social desirability bias. Second, some ITC survey response rates were low due to the nature of the mode and/or protocols for data collection (Supplementary table 1), which may limit the generalizability of the study findings. Third, we found statistically significant positive correlations for all three smoking-caused diseases in the 12 overlapping ITC and GATS countries, while non-significant correlations were found for the two SHS-caused diseases due to reduced power from the small sample size (N=7 observations). Caution is warranted in the comparison of ITC and GATS estimates due to differences in

For peer review only - http://bmjopen.bmj.com/site/about/guidelines.xhtml

research designs, sampling across ITC Surveys, and between ITC Surveys and GATS, survey year(s) in overlapping ITC and GATS countries, and ITC Survey and GATS questions used to measure knowledge (Supplementary table 1, Supplementary table 2, Supplementary table 4). However, correlation of the ITC and GATS data despite their different research and methodological designs, allows for more confident inferences.[34][35] Fourth, ITC and GATS data correlation analyses were limited to 12 countries, so the results are not generalizable. Finally, our results are limited to knowledge about the harms of smoking among adults who currently smoke tobacco. Our finding for an overall near-zero net change in knowledge over time may or may not reflect changes in overall knowledge in the population. It is possible that while knowledge about diseases caused by smoking and SHS among adults who smoke did not change over time, knowledge may have increased among adults who have quit smoking and those who have never smoked. As such, we examined whether changes in knowledge for each disease outcome differed by smoking status across 12 GATS countries where more than one survey wave was conducted among adults who smoke, adults who have quit smoking, and adults who have never smoked.[d] In general, there were no consistent patterns for changes in knowledge about each disease outcome across the 12 GATS countries (Supplementary table 5). Non-significant increases/decreases in knowledge over time among adults who quit and those who never smoked suggests that this pattern smokers is consistent with that in the overall population.

---

[d] ITC longitudinal cohort surveys collect data from adults who smoke. Adults who quit smoking at follow–up survey waves are weighted as adults who smoke. Given that population representative samples of adults who have never smoked and those who have quit smoking are not available, the analysis of changes in knowledge for each disease outcome by smoking status (adults who smoke, adults who have quit smoking, and adults who have never smoked) was not conducted with ITC data, as it was done with GATS data.

For peer review only - http://bmjopen.bmj.com/site/about/guidelines.xhtml

This study found high knowledge that smoking causes lung cancer among adults who smoke, lower knowledge that smoking causes CVD, and even lower knowledge for SHS harms. There was no overall change in knowledge over two decades. The United Nations 2030 Agenda for Sustainable Development calls for strengthening implementation of the WHO FCTC to achieve the target for a one-third reduction in premature mortality from NCDs by 2030.[36] To achieve this, countries could consider implementation of WHO FCTC measures shown to have synergistic effects on promoting smoking cessation and reducing smoking prevalence.[37,38] An important factor that can facilitate efforts to strengthen policies is to increase the awareness of the harms of using cigarettes and other smoked tobacco products.

For peer review only

For peer review only - http://bmjopen.bmj.com/site/about/guidelines.xhtml

References

1 Murray CJL, Aravkin AY, Zheng P, *et al.* Global burden of 87 risk factors in 204 countries and territories, 1990–2019: a systematic analysis for the Global Burden of Disease Study 2019. *The Lancet*. 2020;396:1223–49. doi: 10.1016/S0140-6736(20)30752-2

2 Yang J, Hammond D, Driezen P, *et al.* Health knowledge and perception of risks among Chinese smokers and non-smokers: findings from the Wave 1 ITC China Survey. *Tobacco Control*. 2010;19:i18–23. doi: 10.1136/TC.2009.029710

3 Demissie HS, Smith T, de Quevedo IG, *et al.* Factors associated with quit attempt and successful quitting among adults who smoke tobacco in Ethiopia: Global Adult Tobacco Survey (GATS) 2016. *Tob Prev Cessat*. 2022;8:12. doi: 10.18332/tpc/146170

4 Hyland A, Li Q, Bauer JE, *et al.* Predictors of cessation in a cohort of current and former smokers followed over 13 years. *Nicotine & Tobacco Research.* 2004;6:S363-9.

5 Evans KA, Sims M, Judge K, *et al.* Assessing the knowledge of the potential harm to others caused by second-hand smoke and its impact on protective behaviours at home. *Journal of Public Health*. 2012;34:183–94. doi: 10.1093/pubmed/fdr104

6 World Health Organization. WHO Framework Convention on Tobacco Control. 2003.

7 World Health Organization. MPOWER. A policy package to reverse the tobacco epidemic. Geneva 2008.

8 Hammond D. Health warning messages on tobacco products: a review. *Tobacco Control*. 2011;20:327–37. doi: 10.1136/tc.2010.037630

9 World Health Organization. Tackling NCDs: ‘best buys’ and other recommended interventions for prevention and control of noncommunicable diseases. Geneva 2017.

10 Brinn M, Carson K, Esterman A, *et al.* Mass media interventions for preventing smoking in young people. *Cochrane Database of Systematic Reviews*. 2010;10:Art. No.: CD001006. doi: 10.1002/14651858.CD001006.pub3

11 Chiosi JJ, Andes L, Asma S, *et al.* Warning about the harms of tobacco use in 22 countries: findings from a cross-sectional household survey. *Tobacco Control*. 2016;25:393–401. doi: 10.1136/tobaccocontrol-2014-052047

12 Siahpush M, McNeill A, Hammond D, *et al.* Socioeconomic and country variations in knowledge of health risks of tobacco smoking and toxic constituents of smoke:

For peer review only - http://bmjopen.bmj.com/site/about/guidelines.xhtml

results from the 2002 International Tobacco Control (ITC) Four Country Survey. *Tobacco Control*. 2006;15:iii65–70. doi: 10.1136/TC.2005.013276

13 Mills SD, Wiesen CA. Beliefs about the health effects of smoking among adults in the United States. *Health Educ Behav*. 2022;49:497–505. doi: 10.1177/10901981211004136

14 Trofor AC, Papadakis S, Lotrean LM, *et al.* Knowledge of the health risks of smoking and impact of cigarette warning labels among tobacco users in six European countries: Findings from the EUREST-PLUS ITC Europe Surveys. *Tobacco Induced Diseases*. 2018;16. doi: 10.18332/tid/99542

15 Ahluwalia I, Smith T, Arrazola R, *et al.* Current tobacco smoking, quit attempts, and knowledge about smoking risks among persons aged ≥15 Years - Global Adult Tobacco Survey, 28 Countries, 2008-2016. *MMWR*. 2018;67:1072–6. doi: 10.15585/MMWR.MM6738A7

16 Lonergan BJ, Meaney S, Perry IJ, *et al.* Smokers still underestimate the risks posed by secondhand smoke: a repeated cross-sectional study. *Nicotine & Tobacco Research*. 2014;16:1121–8. doi: 10.1093/ntr/ntu046

17 World Health Organization. Noncommunicable disease surveillance, monitoring and reporting. Global Adult Tobacco Survey. 2022. https://www.who.int/teams/noncommunicable-diseases/surveillance/systems-tools/global-adult-tobacco-survey

18 Thompson ME, Driezen P, Boudreau C, *et al.* Methods of the International Tobacco Control (ITC) EUREST-PLUS ITC Europe Surveys. *Eur J Public Health*. 2020;30:iii4–9. doi: 10.1093/eurpub/ckz212

19 ITC Project. ITC Project. Methods. Technical reports. International Tobacco Control Policy Evaluation Project. Methods. 2024. https://itcproject.org/methods/technical-reports/

20 Global Tobacco Surveillance System. GTSS Academy. Global Tobacco Surveillance System. Academy. 2024.

21 Roth GA, Mensah GA, Johnson CO, *et al.* Global burden of cardiovascular diseases and risk factors, 1990-2019: update from the GBD 2019 Study. *Journal of the American College of Cardiology*. 2020;76:2982–3021. doi: 10.1016/j.jacc.2020.11.010

22 Global Burden of Disease Collaborative Network. Global Burden of Disease Study 2019 (GBD 2019) results. Institute for Health Metrics and Evaluation. 2020. https://vizhub.healthdata.org/gbd-results/

23 Reitsma MB, Kendrick PJ, Ababneh E, *et al.* Spatial, temporal, and demographic patterns in prevalence of smoking tobacco use and attributable disease burden in

For peer review only - http://bmjopen.bmj.com/site/about/guidelines.xhtml

204 countries and territories, 1990–2019: a systematic analysis from the Global Burden of Disease Study 2019. *The Lancet*. 2021;397:2337–60. doi: 10.1016/S0140-6736(21)01169-7

24 Öberg M, Jaakkola MS, Woodward A, *et al.* Worldwide burden of disease from exposure to second-hand smoke: a retrospective analysis of data from 192 countries. *The Lancet*. 2011;377:139–46. doi: 10.1016/S0140-6736(10)61388-8

25 Brauer M, Roth G, Aravkin A, *et al.* Global burden and strength of evidence for 88 risk factors in 204 countries and 811 subnational locations, 1990-2021: a systematic analysis for the Global Burden of Disease Study 2021. *Lancet*. 2024;403:2162–203. doi: 10.1016/S0140-6736(24)00933-4

26 World Health Organization. Tobacco control (TFI). 2021.

27 Noar S, Francis D, Bridges C, *et al.* The impact of strengthening cigarette pack warnings: systematic review of longitudinal observational studies. *Social Science & Medicine*. 2016;164:118–29. doi: 10.1016/j.socscimed.2016.06.011

28 Ngo A, Cheng KW, Shang C, *et al.* Global evidence on the association between cigarette graphic warning labels and cigarette smoking prevalence and consumption. *International Journal of Environmental Research and Public Health*. 2018;15:421. doi: 10.3390/IJERPH15030421

29 World Health Organization. WHO report on the global tobacco epidemic, 2013: enforcing bans on tobacco advertising, promotion and sponsorship. Geneva: World Health Organization 2013.

30 World Health Organization. WHO report on the global tobacco epidemic, 2011: warning about the dangers of tobacco. Geneva: World Health Organization 2011.

31 World Health Organization. WHO report on the global tobacco epidemic, 2023: protect people from tobacco smoke. Geneva: World Health Organization 2023.

32 World Health Organization. WHO global report on trends in prevalence of tobacco use 2000-2025, fourth edition. Geneva: World Health Organization 2021.

33 World Health Organization. WHO Framework Convention on Tobacco Control. Parties. WHO Framework Convention on Tobacco Control. 2024. https://fctc.who.int/who-fctc/overview/parties

34 Hill AB. The environment and disease: association or causation? *Proc R Soc Med*. 1965;58:295–300.

35 Campbell DT, Fiske DW. Convergent and discriminant validation by the multitrait-multimethod matrix. *Psychological Bulletin*. 1959;56:81–105. doi: 10.1037/h0046016

For peer review only - http://bmjopen.bmj.com/site/about/guidelines.xhtml

36 United Nations. Transforming our world: the 2030 Agenda for Sustainable Development. 2015.

37 Chung-Hall J, Craig L, Gravely S, *et al.* Impact of the WHO FCTC over the first decade: a global evidence review prepared for the Impact Assessment Expert Group. *Tobacco Control*. 2019;28:s119–28. doi: 10.1136/tobaccocontrol-2018-054389

38 Hoffman SJ, Tan C. Overview of systematic reviews on the health-related effects of government tobacco control policies. *BMC Public Health*. 2015;15:744. doi: 10.1186/s12889-015-2041-6

For peer review only

Figure 1A. Percentage of adults who smoke who know or believe that smoking causes lung cancer,† in 25 ITC countries,‡ 2002–2022.

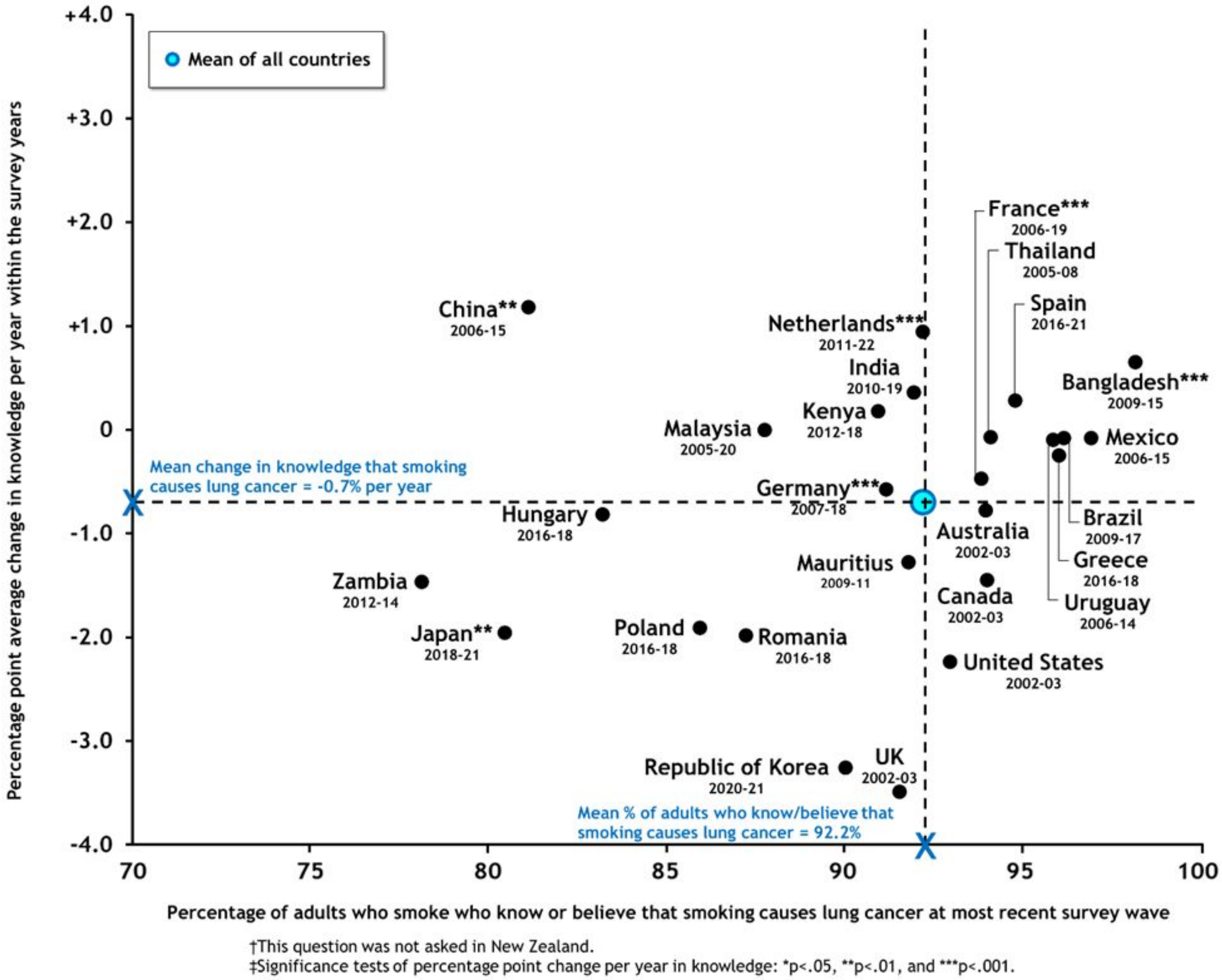

Figure 1B. Percentage of adults who smoke who know or believe that smoking causes lung cancer, in 32 GATS countries,† 2008–2018.

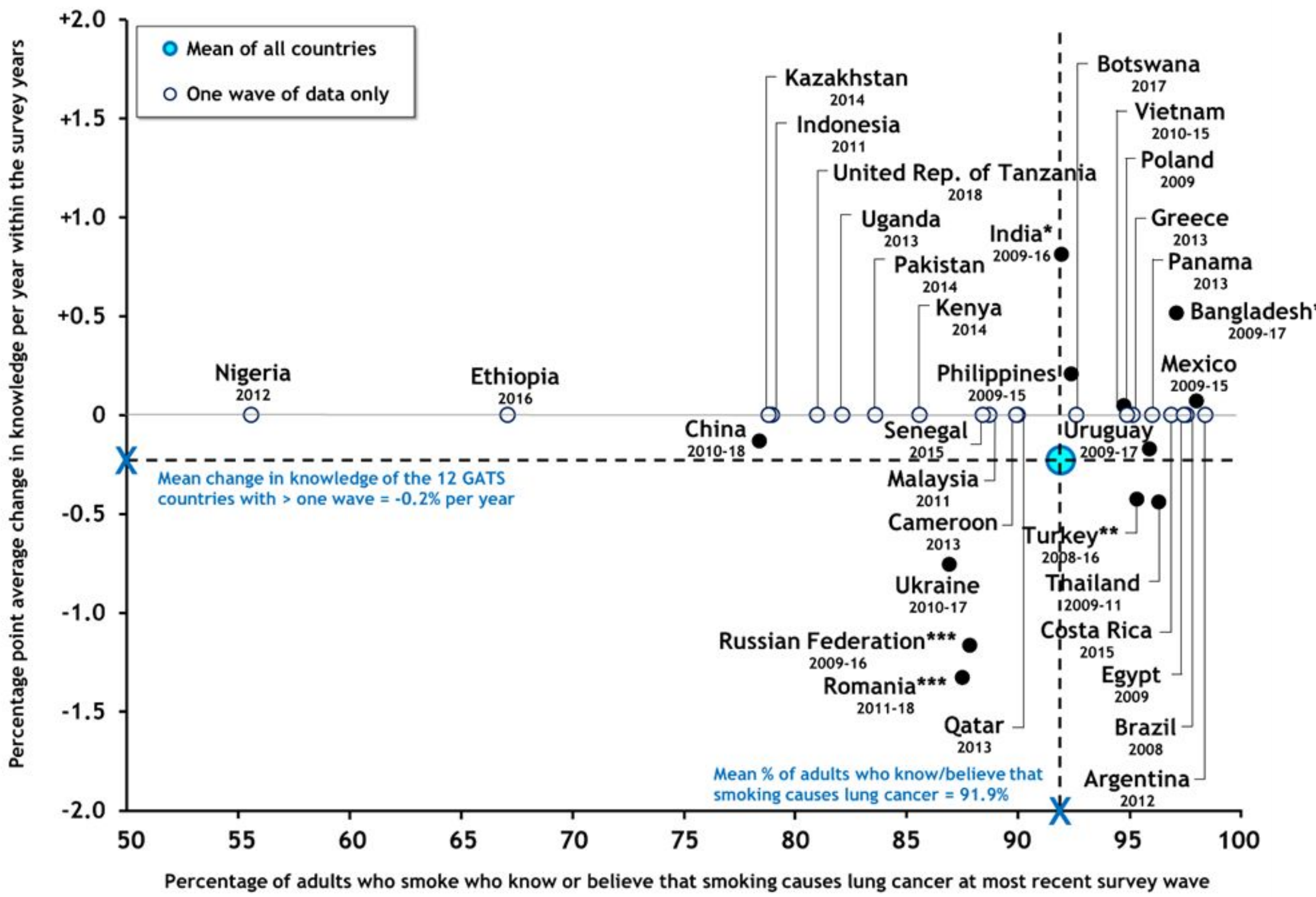

†Significance tests of percentage point change per year in knowledge: *p<.05, **p<.01, and ***p<.001.

1

Figure 2A. Percentage of adults who smoke who know or believe that smoking causes heart disease,† in 25 ITC countries,‡ 2002–2022.

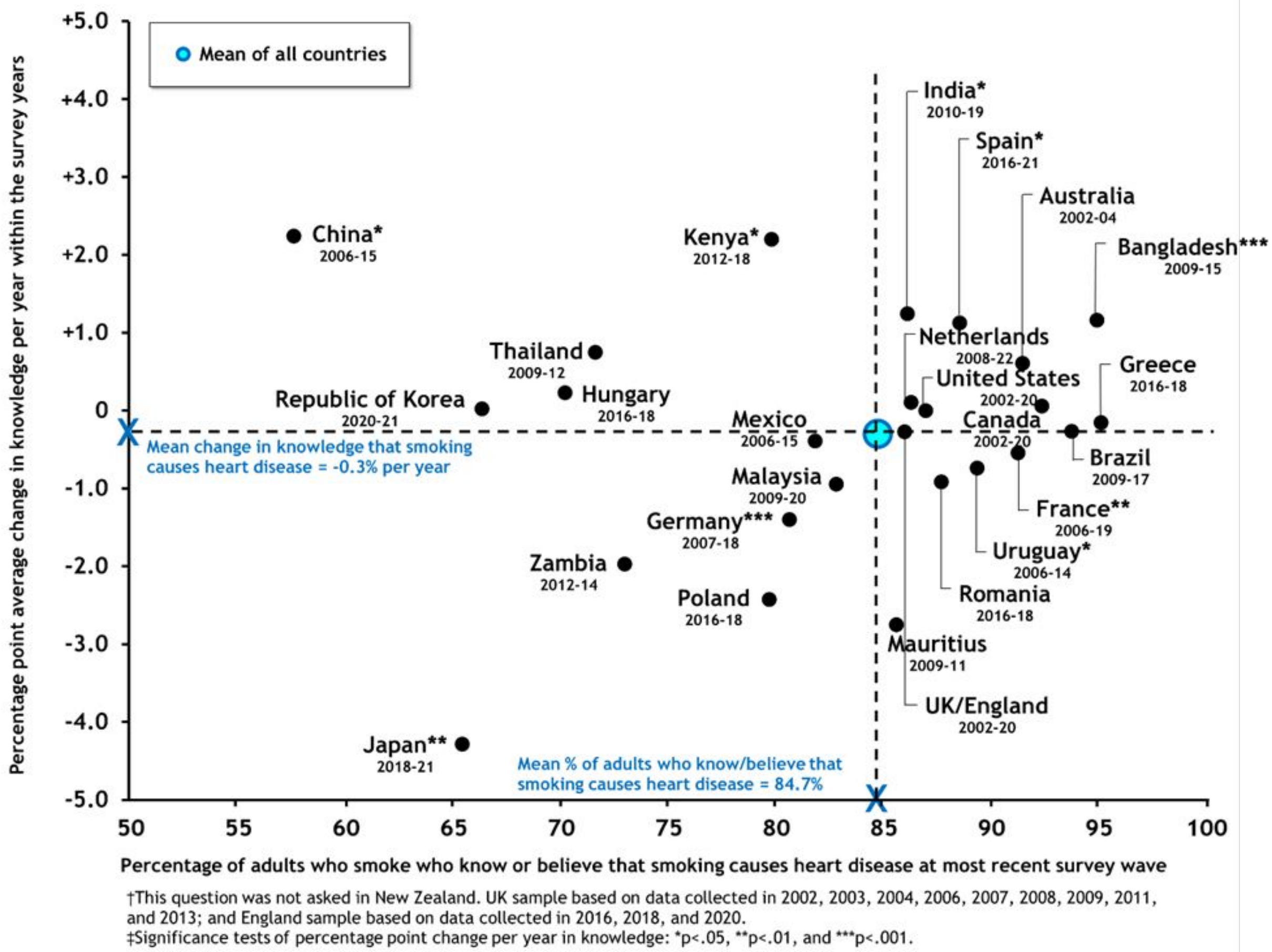

For peer review only - http://bmjopen.bmj.com/site/about/guidelines.xhtml

Figure 2B. Percentage of adults who smoke who know or believe that smoking causes heart attack, in 32 GATS countries,† 2008–2018.

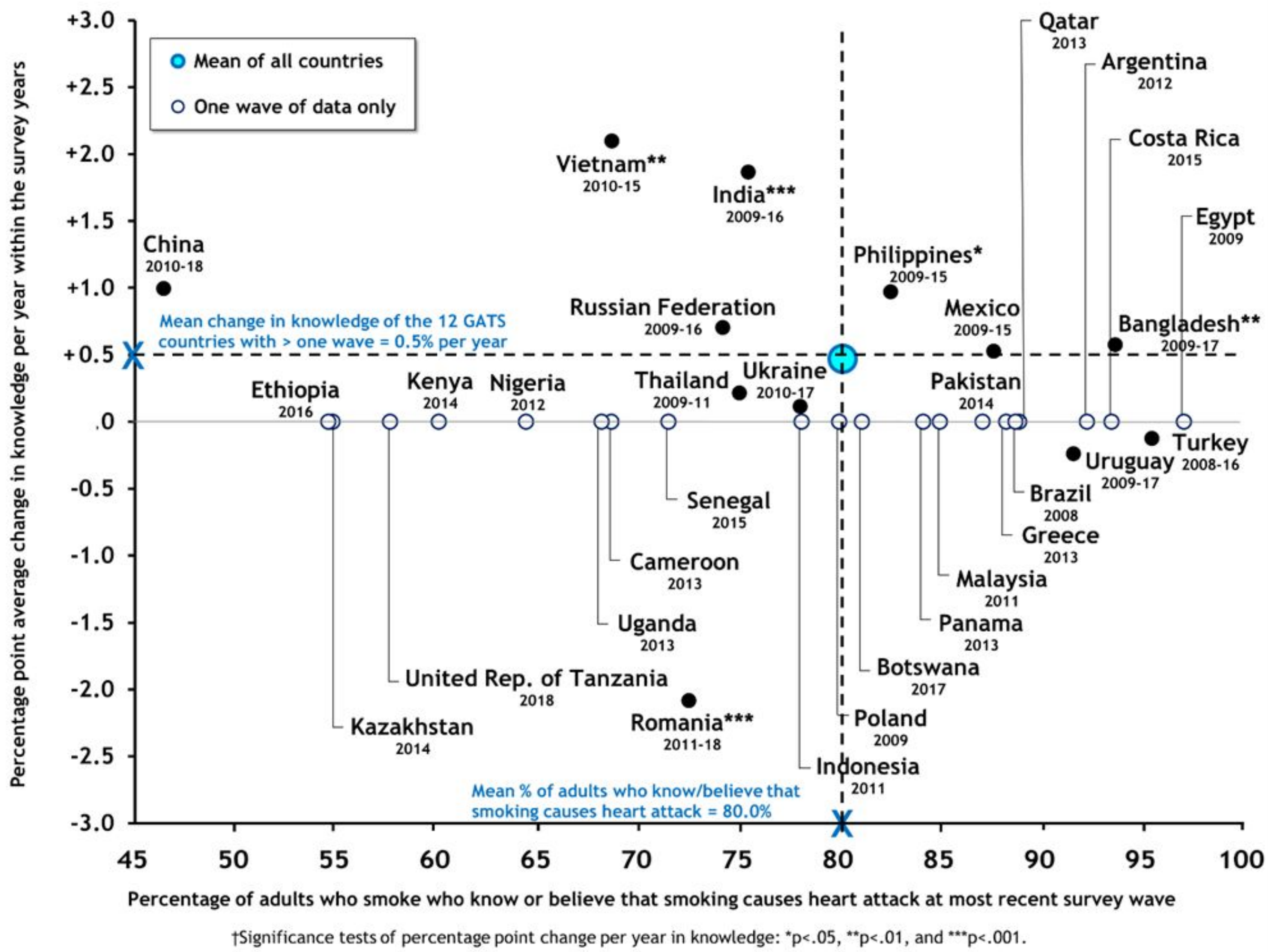

Figure 3A. Percentage of adults who smoke who know or believe that smoking causes stroke,† in 25 ITC countries,‡ 2002–2022.

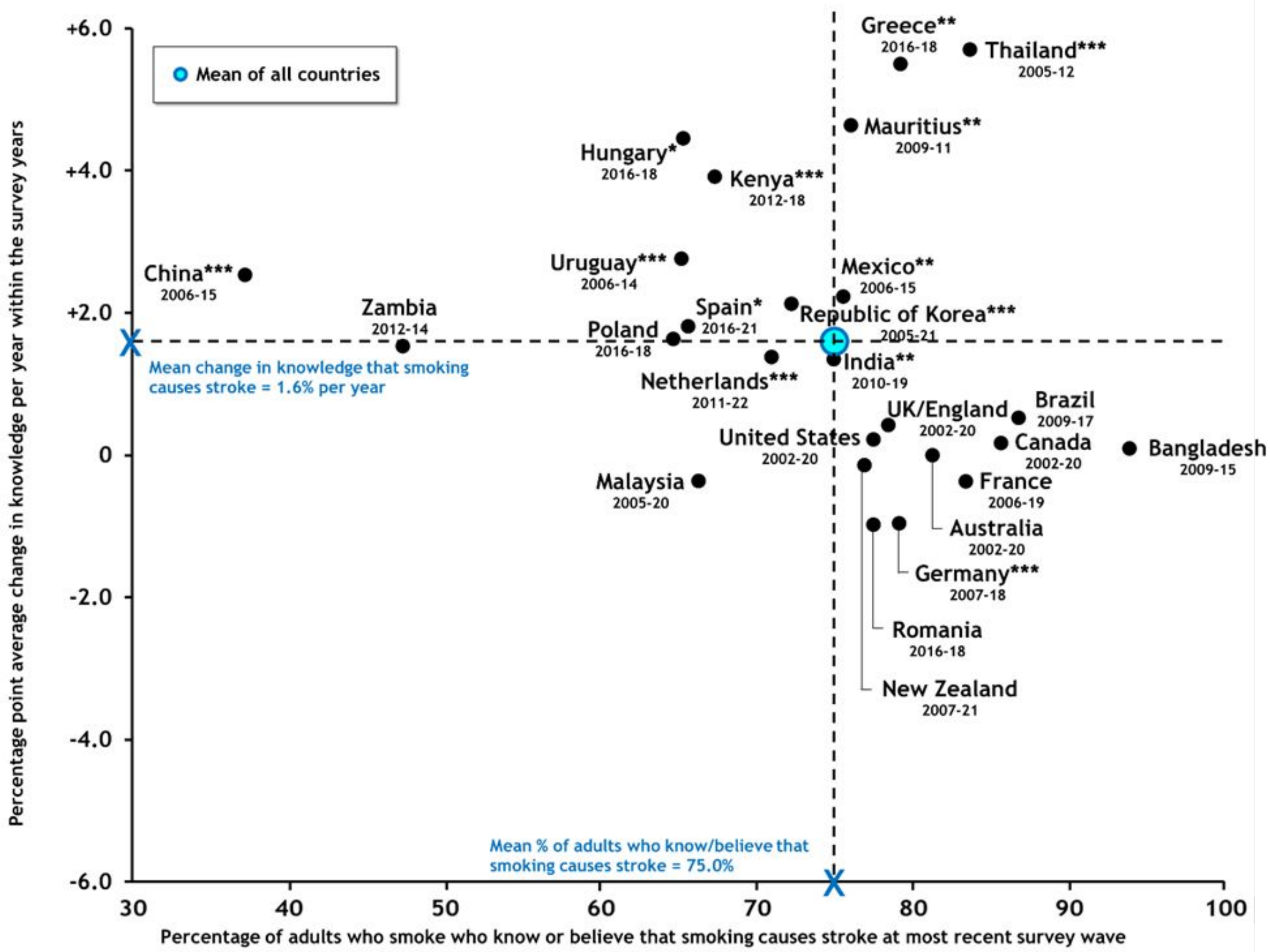

†This question was not asked in Japan. UK sample based on data collected in 2002, 2003, 2004, 2006, 2007, 2008, 2009, 2011, and 2013; and England sample based on data collected in 2016, 2018, and 2020.
‡Significance tests of percentage point change per year in knowledge: *p<.05, **p<.01, and ***p<.001.

For peer review only - http://bmjopen.bmj.com/site/about/guidelines.xhtml

Figure 3B. Percentage of adults who smoke who know or believe that smoking causes stroke, in 32 GATS countries,† 2008–2018.

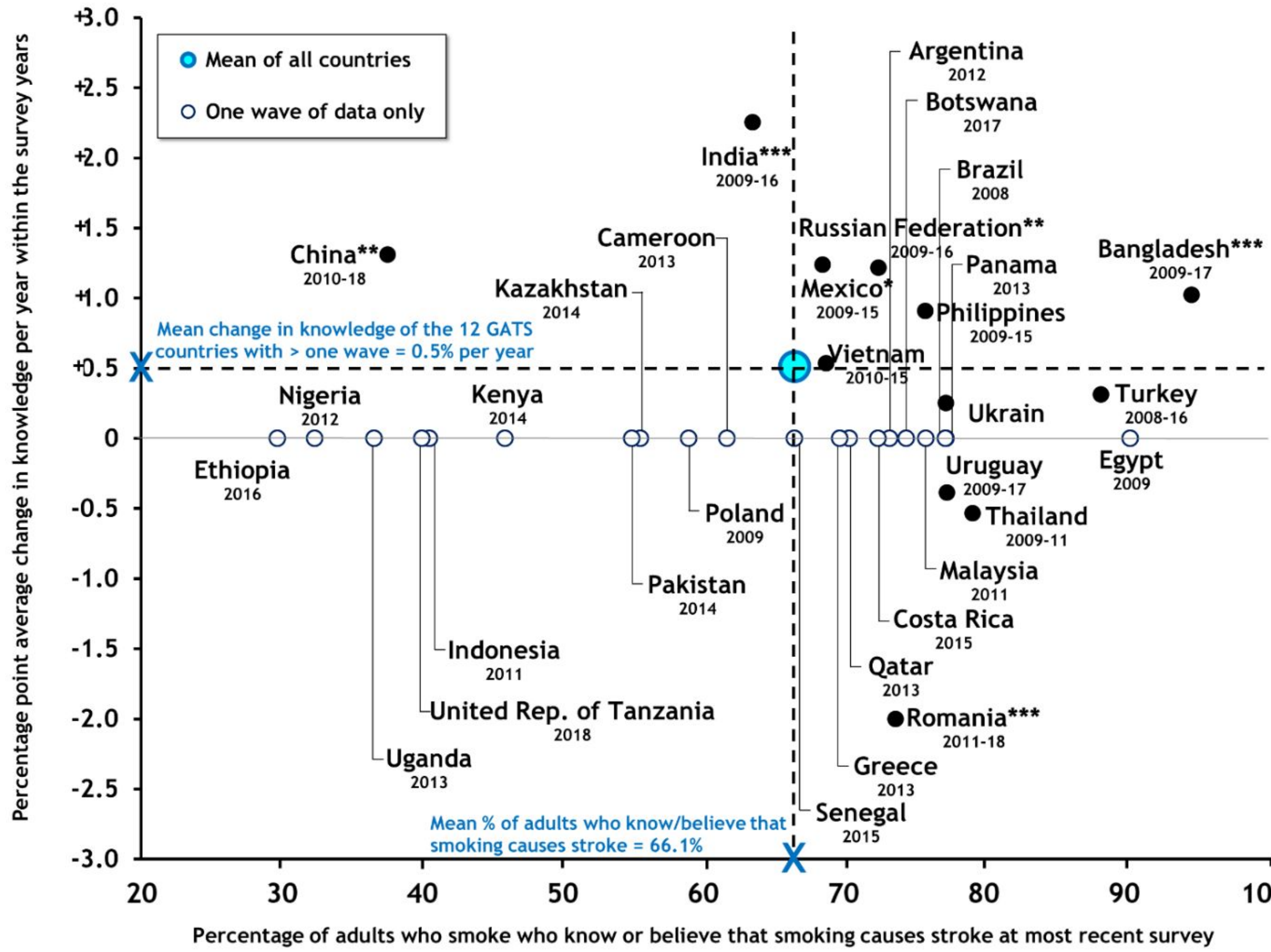

†Significance tests of percentage point change per year in knowledge: *p<.05, **p<.01, and ***p<.001.

For peer review only - http://bmjopen.bmj.com/site/about/guidelines.xhtml

Figure 4A. Percentage of adults who smoke who know or believe that secondhand smoke causes lung cancer, in 26 ITC countries,† 2003–2022.

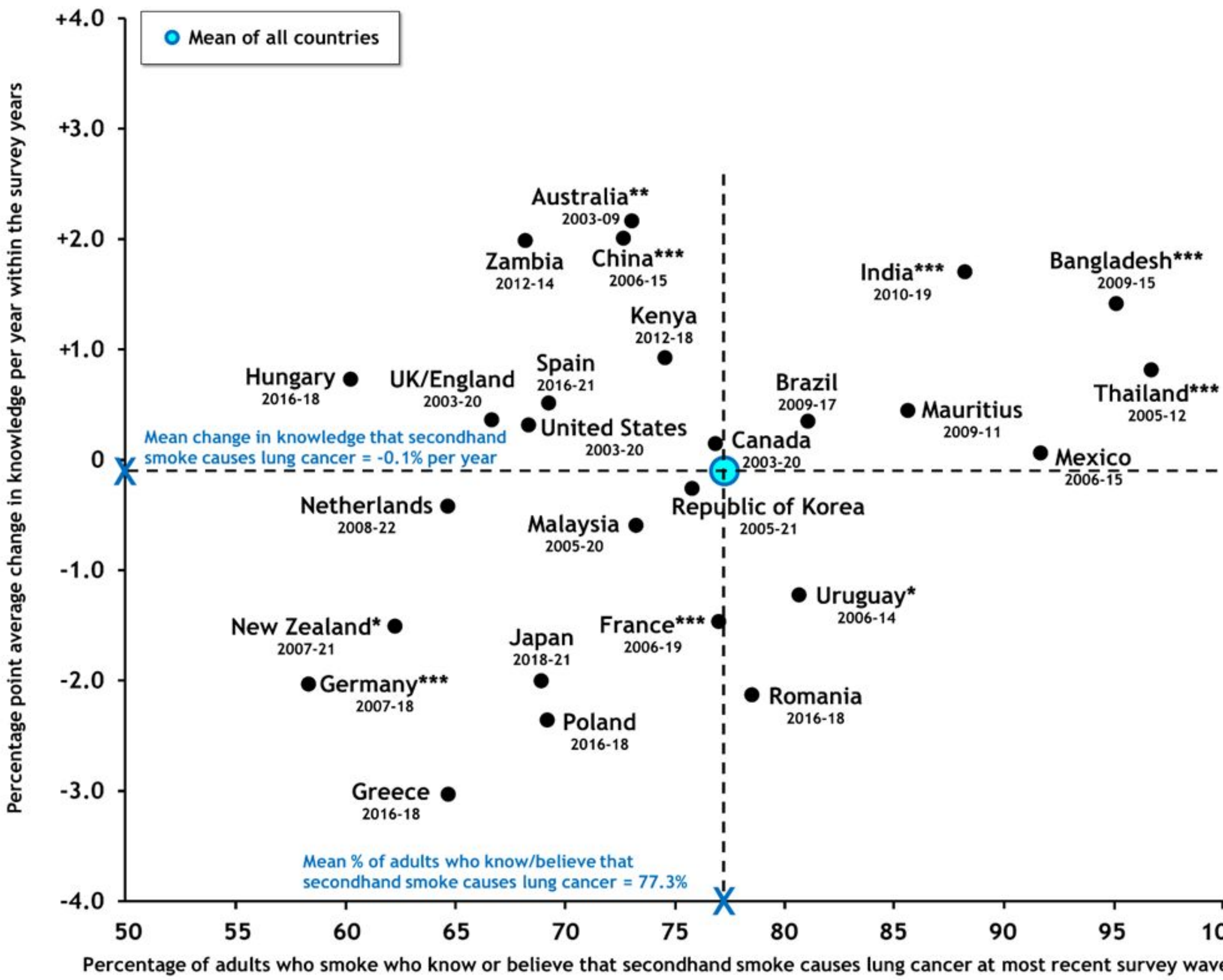

†UK sample based on data collected in 2003, 2004, 2006, 2007, 2008, 2009, 2011, and 2013; and England sample based on data collected in 2016, 2018, and 2020.
‡Significance tests of percentage point change per year in knowledge: *p<.05, **p<.01, and ***p<.001.

Figure 4B. Percentage of adults who smoke who know or believe that secondhand smoke causes lung cancer, in 18 GATS countries,† 2009–2018.

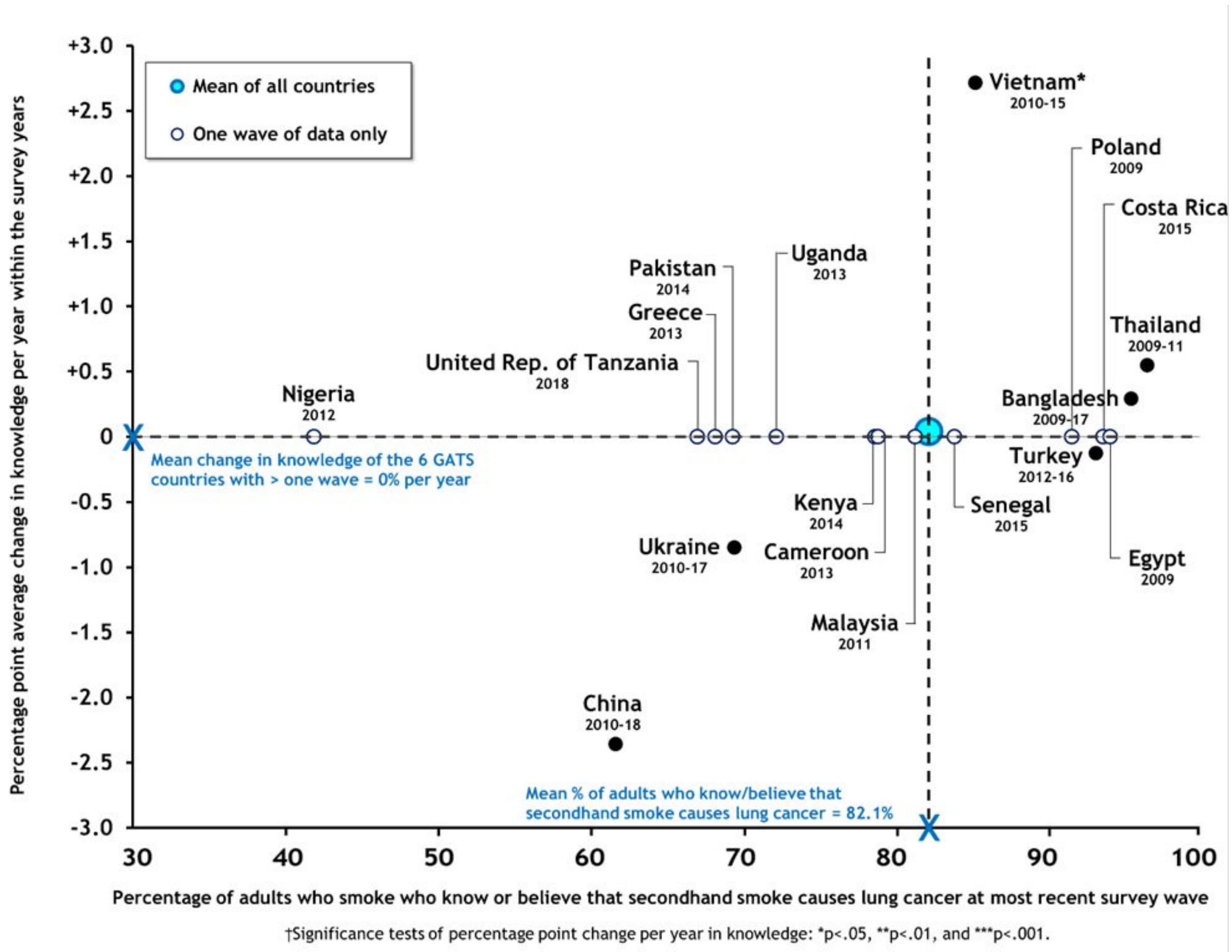

1

Figure 5A. Percentage of adults who smoke who know or believe that secondhand smoke causes heart attack,† in 24 ITC countries,‡ 2008–2022.

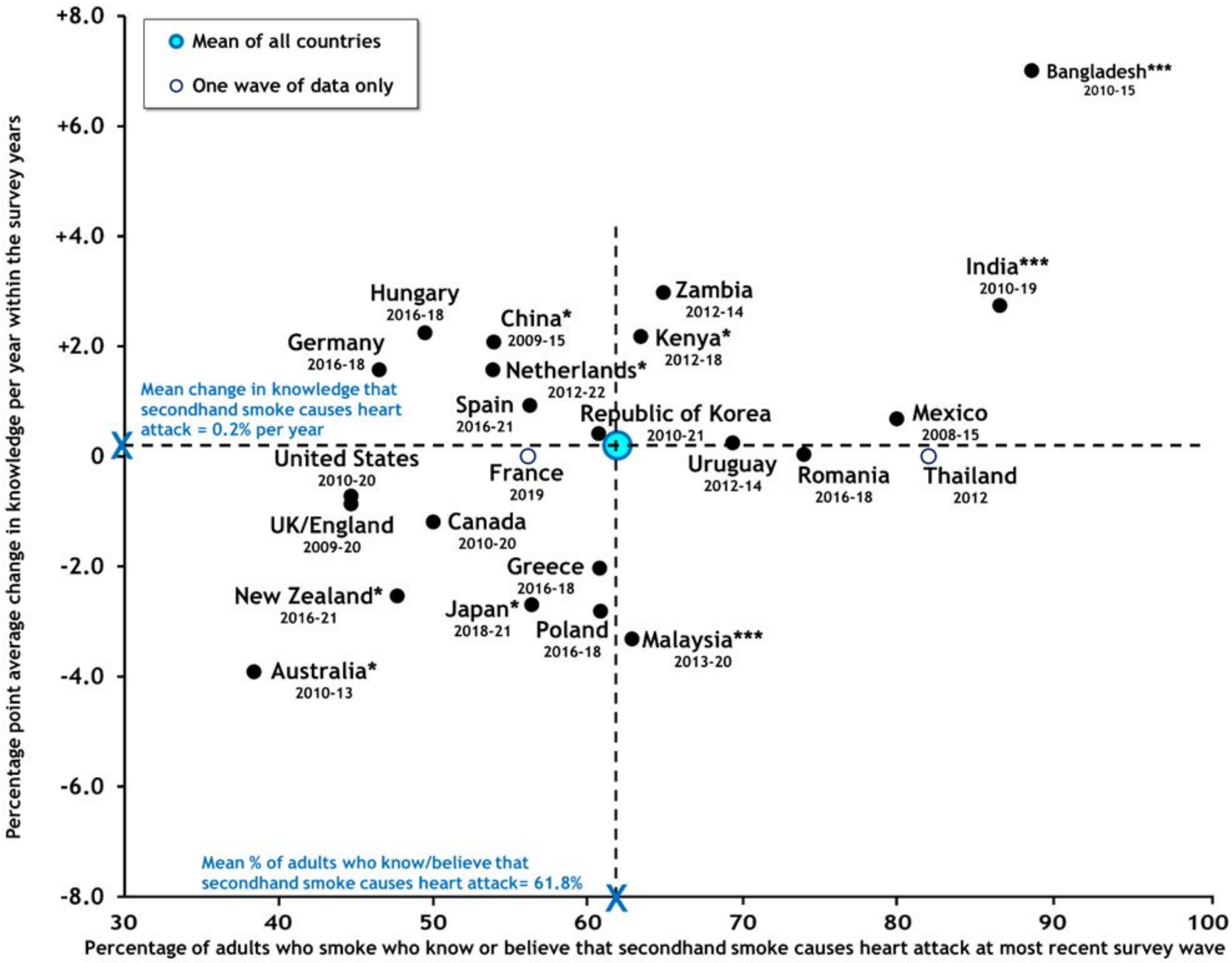

†This question was not asked in Brazil and Mauritius. UK sample based on data collected in 2009, 2011, and 2013; and England sample based on data collected in 2016, 2018, and 2020.
‡Significance tests of percentage point change per year in knowledge: *p<.05, **p<.01, and ***p<.001.

For peer review only - http://bmjopen.bmj.com/site/about/guidelines.xhtml

Figure 5B. Percentage of adults who smoke who know or believe that secondhand smoke causes heart disease, in 18 GATS countries,† 2009–2018.

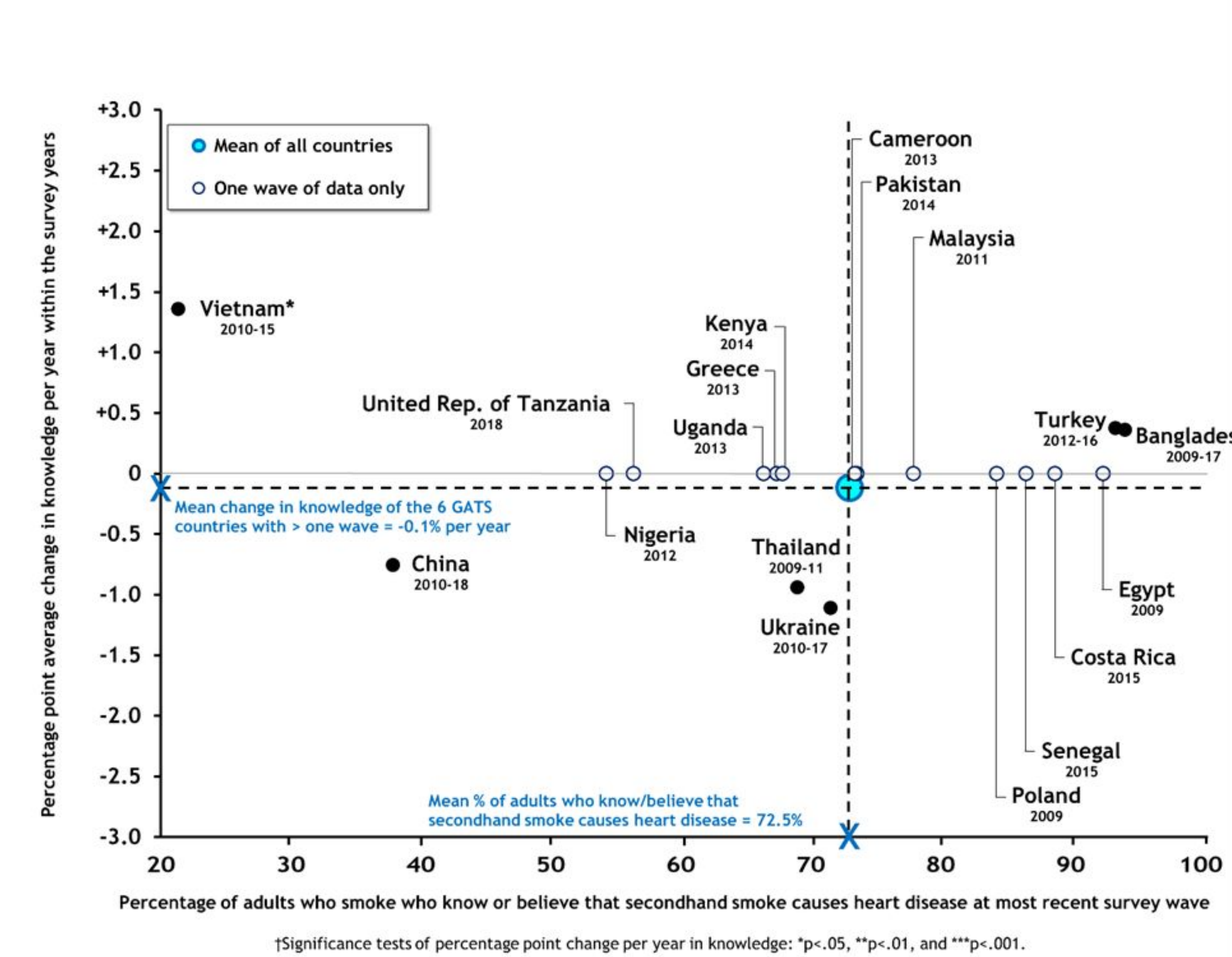

For peer review only - http://bmjopen.bmj.com/site/about/guidelines.xhtml

BMJ Open

Changes in knowledge about major harms of tobacco smoking and secondhand smoke exposure among adults who smoke: findings from 26 countries of the International Tobacco Control (ITC) Project (2002–2022) and 32 countries of the Global Adult Tobacco Survey (GATS) (2008–2020)

Journal:	*BMJ Open*
Manuscript ID	bmjopen-2025-107667.R1
Article Type:	Original research
Date Submitted by the Author:	10-Feb-2026
Complete List of Authors:	Chung-Hall, Janet; University of Waterloo, Psychology<br>Fong, Geoffrey; University of Waterloo, Psychology, School of Public Health Sciences; Ontario Institute for Cancer Research<br>Meng, Gang; University of Waterloo<br>Craig, Lorraine; University of Waterloo, Psychology<br>Indome, Eunice; University of Waterloo<br>Kress, Alissa; National Center for Chronic Disease Prevention and Health Promotion Office on Smoking and Health<br>Shi, Jing; National Center for Chronic Disease Prevention and Health Promotion Office on Smoking and Health; CDC Foundation Inc, Noninfectious Disease Programs<br>Ahluwalia, Indu; National Center for Chronic Disease Prevention and Health Promotion Office on Smoking and Health<br>Ahluwalia, Indu; Office on Smoking and Health, National Center for Chronic Disease Prevention and Health Promotion, Centers for Disease Control and Prevention, Atlanta, Georgia, USA
**Primary Subject Heading**:	Public health
Secondary Subject Heading:	Global health, Health policy, Smoking and tobacco
Keywords:	PUBLIC HEALTH, Tobacco Use, Health

SCHOLARONE™
MANUSCRIPTS

I, the Submitting Author has the right to grant and does grant on behalf of all authors of the Work (as defined in the below author licence), an exclusive licence and/or a non-exclusive licence for contributions from authors who are: i) UK Crown employees; ii) where BMJ has agreed a CC-BY licence shall apply, and/or iii) in accordance with the terms applicable for US Federal Government officers or employees acting as part of their official duties; on a worldwide, perpetual, irrevocable, royalty-free basis to BMJ Publishing Group Ltd ("BMJ") its licensees and where the relevant Journal is co-owned by BMJ to the co-owners of the Journal, to publish the Work in this journal and any other BMJ products and to exploit all rights, as set out in our licence.

The Submitting Author accepts and understands that any supply made under these terms is made by BMJ to the Submitting Author unless you are acting as an employee on behalf of your employer or a postgraduate student of an affiliated institution which is paying any applicable article publishing charge ("APC") for Open Access articles. Where the Submitting Author wishes to make the Work available on an Open Access basis (and intends to pay the relevant APC), the terms of reuse of such Open Access shall be governed by a Creative Commons licence – details of these licences and which Creative Commons licence will apply to this Work are set out in our licence referred to above.

Other than as permitted in any relevant BMJ Author's Self Archiving Policies, I confirm this Work has not been accepted for publication elsewhere, is not being considered for publication elsewhere and does not duplicate material already published. I confirm all authors consent to publication of this Work and authorise the granting of this licence.

For peer review only

Manuscript ID BMJOpen-2025-108667

Changes in knowledge about major harms of tobacco smoking and secondhand smoke exposure among adults who smoke: findings from 26 countries of the International Tobacco Control (ITC) Project (2002–2022) and 32 countries of the Global Adult Tobacco Survey (GATS) (2008–2020)

Janet Chung–Hall,[1] Geoffrey T. Fong, [1,2,3] Gang Meng,[1] Lorraine V. Craig,[1] and Eunice O. Indome[1] for the ITC Project Collaborators, and Alissa C. Kress,[4] Jing Shi,[4,5] and Indu B. Ahluwalia[4] for the Global Adult Tobacco Survey Team

Affiliation(s):
[1] Department of Psychology, University of Waterloo, Waterloo, Ontario, Canada
[2] School of Public Health Sciences, University of Waterloo, Waterloo, Ontario, Canada
[3] Ontario Institute for Cancer Research, Toronto, Ontario, Canada
[4] Office on Smoking and Health, National Center for Chronic Disease Prevention and Health Promotion, Centers for Disease Control and Prevention, Atlanta, Georgia, USA
[5] Noninfectious Disease Programs, CDC Foundation, Atlanta, Georgia, USA assigned to the Office on Smoking and Health, National Center for Chronic Disease Prevention and Health Promotion, Centers for Disease Control and Prevention

Corresponding author:
Dr. Janet Chung-Hall
University of Waterloo
Department of Psychology, ITC Project
200 University Avenue West
Waterloo, Ontario, Canada, N2L 3G1
Email: jchunghall@uwaterloo.ca

Word count: 5117

Disclaimer: The findings and conclusions in this article are those of the authors and do not necessarily represent the official positions of the U.S. Centers for Disease Control and Prevention or the CDC Foundation.

Funding Statement: The ITC Four Country Surveys (Australia, Canada, the United Kingdom, the United States) were supported by grants from the US National Cancer Institute (R01 CA100362, R01 CA090955, P50 CA111236 (Roswell Park Transdisciplinary Tobacco Use Research Center), and P01 CA138389), Robert Wood

For peer review only

Johnson Foundation (045734), the Canadian Institutes of Health Research (FDN-148477, MOP-57897, MOP-79551, MOP-115016), the Canadian Tobacco Control Research Initiative (014578), Australia Commonwealth Department of Health and Ageing, the National Health and Medical Research Council of Australia (265903, 450110, 1005922), and Cancer Research UK (C312/A3726, C312/A6465, C321/A11039, C25586/A19540). The ITC Four Country Smoking and Vaping Surveys (Australia, Canada, England, and the United States) were supported by grants from the US National Cancer Institute (P01 CA200512), the Canadian Institutes of Health Research (FDN-148477), and the National Health and Medical Research Council of Australia (APP1106451). The ITC Bangladesh Surveys were supported by grants from the US National Cancer Institute (P01 CA138389), the International Development Research Centre (IDRC Grant 104831-003), and Canadian Institutes for Health Research (MOP-79551, MOP-115016). The ITC Brazil Surveys were supported by the Instituto Nacional de Câncer (INCA, National Cancer Institute), Secretaria Nacional de Pokiticas sobre Drogas (SENAD, National Secretariat for Drug Policy), Pan-American Health Organization (PAHO), Brazil Fundação do Câncer (BR/LOA/1500048·00), and the Canadian Institutes of Health Research (MOP-115016, FDN-148477). The ITC China Surveys were supported by grants from the US National Cancer Institute at the National Institutes of Health (R01 CA125116), the Roswell Park Transdisciplinary Tobacco Use Research Center (P50 CA111236), Canadian Institutes for Health Research (MOP-79551, MOP-115016), and the Chinese Center for Disease Control and Prevention. The ITC France Surveys (first cohort) were supported by grants from the Observatoire français des drogues et toxicomanies (OFDT), Institut national de prevention et d'education pour la sante (INPES), and the Institut nationale du cancer (INCa). The ITC France Surveys (second cohort) were supported by grants from Santé publique France, the Institut nationale du cancer (INCa), and the Canadian Institutes of Health Research (FDN-148477). The ITC Germany Surveys (first cohort) were supported by grants from the German Federal Ministry of Health, Dieter Mennekes-Umweltstiftung, and Germany Cancer Research Center (DKFZ). The EUREST-PLUS Project (ITC 6 European Country Surveys) in Germany (second cohort), Greece, Hungary, Poland, Romania, and Spain has received funding from the European Union's Horizon2020 research and innovation programme under grant agreement No·681109 (C.I.V.) and the University of Waterloo (G.T.F.), and additional support was provided to the University of Waterloo by the Canadian Institutes of Health Research (FDN-148477). EF is partly supported by Ministry of Universities and Research, Government of Catalonia (2017SGR319) and by the Instituto Carlos III and co-funded by the European Regional Development Fund (FEDER) (INT16/00211 and INT17/00103), Government of Spain and thanks CERCA Programme Generalitat de Catalunya for the institutional support to IDIBELL. The EUREST-PLUS Spain Survey was supported by grants from the Instituto de Salud Carlos III (PI17/01338, Co-funded by European Regional Development Fund ERDF, a way to build Europe) and the Canadian Institute for Health Research (FDN-148477). EF and MF are partly supported by the Ministry of Universities and Research, Government of Catalonia (2021SGR00906). EF and MF are partly supported by the Ministry of Universities and Research, Government of Catalonia (2021SGR00906) and thank CERCA Programme Generalitat de Catalunya for the institutional support to IDIBELL. The ITC India Surveys were supported by grants from the US National Cancer Institute (P50 CA111236, P01 CA138389), the Canadian Institute of Health Research

For peer review only - http://bmjopen.bmj.com/site/about/guidelines.xhtml

(MOP-79551, MOP-115016, FDN-148477), and Healis Sekhsaria Institute for Public Health. The ITC Japan Surveys were supported by the Japan National Cancer Center and Research Development Fund (28-A-24) and the Canadian Institutes of Health Research (FDN-148477). The ITC Kenya Surveys were supported by grants from the Canadian Institutes of Health Research (MOP-115016, FDN-148477). The ITC Thailand Surveys were supported by grants from the US National Cancer Institute (P50 CA111236 (Roswell Park Transdisciplinary Tobacco Use Research Center), P01 CA138389), the Canadian Institutes of Health Research (MOP-79551, MOP-115016), and the ThaiHealth Promotion Foundation. The ITC Malaysia Surveys (first cohort) were supported by grants from the US National Cancer Institute (P50 CA111236 (Roswell Park Transdisciplinary Tobacco Use Research Center) and P01 CA138389), the Canadian Institutes of Health Research (MOP-79551, MOP-115016), and the Malaysian Ministry of Health. The ITC Malaysia Surveys (second cohort) were supported by the Malaysia Ministry of Higher Education-LRGS NanoMITe (RU029-2014), the University of Malaya Research University Grant (RU029C-2014 and RU001A-2021), and the Canadian Institutes of Health Research (FDN-148477). The ITC Mauritius Surveys were supported by grants from the International Development Research Centre, the Canadian Institutes of Health Research (MOP-115016), and was partially supported by the World Lung Foundation. The ITC Mexico Surveys were supported by grants from the Mexican Consejo Nacional de Ciencia y Tecnología (Salud-2007-C01-70032, Salud-2013-01-202671), Bloomberg Global Initiative—International Union Against Tuberculosis and Lung Disease (IUATLD), the US National Cancer Institute (Roswell Park Transdisciplinary Tobacco Use Research Center (P50 CA111236)) and the Canadian Institutes for Health Research (MOP-115016). The ITC Netherlands Surveys (first cohort) were supported by grants from The Netherlands Organisation for Health Research and Development (ZonMw 121010008, ZonMw 200130002), The SILNE Project (funded by the European Commission through FP7 HEALTH-F3-2011-278273) and the Dutch Cancer Foundation (KWF) (UM 2014-7210). The ITC Netherlands Surveys (second cohort) were supported by grants from Longfonds (Lung Foundation Netherlands), Hartstichting (Netherlands Heart Foundation), KWF Kankerbestrijding (Dutch Cancer Society), Trombosestichting Nederland (Thrombosis Foundation), Diabetesfonds (Diabetes Fund) and the Canadian Institutes of Health Research (FDN-148477). The ITC New Zealand Surveys (first cohort) were supported by grants from the New Zealand Health Research Council (06/453), the New Zealand Ministry of Health, University of Otago, the US National Cancer Institute ((P50 CA111236) Roswell Park Transdisciplinary Tobacco Use Research Centre), and the Canadian Institutes of Health Research (MOP-79551). The ITC New Zealand (EASE: Evidence for Achieving Smokefree 2025 Equitably) Surveys (second cohort) were supported by grants from the New Zealand Health Research Council (GA215F 15/072, HRC 19/641), the New Zealand Ministry of Health, and the Canadian Institute for Health Research (FDN-148477). The ITC Korea Surveys (first cohort) were supported by grants from the National Cancer Center of Korea (0731040-1), the Korean Ministry of Health and Welfare, and the US National Cancer Institute (P50 CA111236, R01 CA125116) and the Canadian Institutes of Health Research (MOP-79551). The ITC Korea Survey (second cohort) was supported by a grant from the Korean Ministry of Health and Welfare and the Canadian Institutes of Health Research (MOP-115016). The ITC Korea Surveys (third cohort) were supported by a grant from the Korean Ministry of Health and Welfare and the Canadian

For peer review only - http://bmjopen.bmj.com/site/about/guidelines.xhtml

Institutes of Health Research (FDN-148477). The ITC Uruguay Surveys were supported by grants from the US National Cancer Institute (P50 CA111236 (Roswell Park Transdisciplinary Tobacco Use Research Center), P01 CA138389), and the Canadian Institutes of Health Research (MOP-115016). The ITC Zambia Surveys were supported by a grant from the Canadian Institutes of Health Research (MOP-115016). Additional support to GTF is provided by a Senior Investigator Grant from the Ontario Institute for Cancer Research (2002-present), and a Senior Prevention Scientist Award from the Canadian Cancer Society Research Institute (2002-2009) and O. Harold Warwick Prize (2021) (additional details in Supplementary file 1A and Supplemental Table 1). Financial support for GATS was provided by the CDC Foundation with grants from Bloomberg Philanthropies and the Bill & Melinda Gates Foundation (additional details in Supplementary file 1B and Supplemental Table 2). The funders had no role in the design of the study; in the collection, analyses, or interpretation of data; in the writing of the manuscript, or in the decision to publish the results.

Competing Interests: KMC has in the past and continues to serve as a paid witness in litigation filed against cigarette manufacturers. GTF has been an expert witness or consultant for governments defending their country's policies or regulations in litigation. CG has been a paid expert witness for the Australian Government in litigation concerning nicotine product marketing. KK received a JMWH Bayer Grant from Japan Society for Menopause and Women's Health. ASAN has received an unconditional educational grant from Johnson & Johnson Malaysia Sdn. Bhd. JFT has served as paid expert witness in legal challenges against tobacco and vaping companies. All other authors have no conflicts of interest to declare.

Patient and Public Involvement: Patients or the public were not involved in the design, or conduct, or reporting, or dissemination plans of our research.

For peer review only

For peer review only - http://bmjopen.bmj.com/site/about/guidelines.xhtml

ABSTRACT

Objectives. To assess levels of and trends in knowledge that smoking and secondhand smoke (SHS) causes lung cancer and cardiovascular disease (CVD) in 46 countries over two decades.

Design. Longitudinal cohort and repeat cross-sectional surveys.

Setting. Nationally representative surveys in 26 International Tobacco Control (ITC) countries (2002-2022), and 32 Global Adult Tobacco Survey (GATS) countries (2008-2020).

Participants. Adults (18+ years) who smoke tobacco (26 ITC countries: N=133,410; 32 GATS countries: N=110,537).

Analysis outcomes. Current use of cigarettes at least monthly (all ITC countries) and/or current use of bidis at least monthly (ITC India, Bangladesh, and Zambia). Current use of any combustible tobacco products (all GATS countries). Generalized estimating equations and weighted multinomial regression models assessed country-level prevalence of knowledge for three smoking and two SHS outcomes. Multilevel models estimated all country mean prevalence and tested within country linear trends over time for five disease indicators by survey. Across 12 countries, with data for both surveys, correlations between ITC and GATS assessed consistency across indicators.

Results. Knowledge that smoking causes lung cancer was highest (ITC=92·2%, GATS=91·9%), followed by heart disease (ITC=84·7%)/heart attack (GATS=80·0%), and stroke (ITC=75·0%, GATS=66·1%). Knowledge that SHS causes lung cancer (ITC=77·3%, GATS=82·1%) was highest, followed by heart attack (ITC=61·8%)/heart disease (GATS=72·5%) (all p<·0001). In ITC and GATS countries, average change in

For peer review only - http://bmjopen.bmj.com/site/about/guidelines.xhtml

knowledge across five diseases was +0·14% per year. There was agreement between ITC and GATS data (range rs= ·60–·81 across five indicators).

Conclusions. There is a need to improve and sustain people's knowledge about diseases caused by smoking and SHS, especially CVD risks.

For peer review only

STRENGTHS AND LIMITATIONS OF THIS STUDY

- This study analyzed nationally representative surveys from 46 countries ─ the International Tobacco Control (ITC) Project and the Global Adult Tobacco Survey (GATS) ─ to assess levels of and trends in knowledge about lung cancer and cardiovascular diseases over two decades.
- Use of self-reported data may be subject to recall bias.
- Generalizability of findings is limited by low response rates for some ITC surveys and the restriction of ITC-GATS correlation analyses to 12 countries.

For peer review only

INTRODUCTION

Over 8 million people die from tobacco-caused diseases per year; tobacco smoking is the single most preventable cause of death globally.[1] The WHO Framework Convention on Tobacco Control (FCTC), entered into force in 2005, calls upon Parties (183 to date) to implement evidence-based measures to reduce tobacco use.[2] The WHO MPOWER[a] package includes implementation of six evidence-based measures at country-level.[3] The W strategies include the display of pictorial health warnings on 75% of the surface of cigarette packs [4,5] and mass media campaigns to increase knowledge about tobacco-caused health risks.[6,7]

Knowledge about the harms of smoking is positively associated with quit intentions,[8] attempts,[9] and cessation success.[10] People who smoke and are unaware of the health risks of active smoking may be less likely to take steps to protect themselves from firsthand smoke exposure. Similarly, low awareness of the harms of secondhand smoke (SHS) exposure may reduce efforts to protect others around them from SHS .[7] Despite efforts to inform people about the harms of smoking and SHS, gaps in knowledge remain. Studies have found that adults in the general population,[11] and those who use cigarettes[12–15] and other smoked tobacco products,[15] report higher knowledge that smoking causes lung cancer compared with heart disease and/or stroke.[b] They are also are more likely to know that SHS exposure causes lung cancer

---

[a] The six MPOWER measures are Monitor tobacco use and policies; Protect people from tobacco smoke; Offer to help quit tobacco use; Warn about dangers of tobacco; Enforce bans on tobacco advertising, promotion, and sponsorship; and Raise tobacco taxes.

[b] The 2023 World Health Organization International Classification of Diseases 11th Revision (ICD–11) defines heart disease and heart attack as circulatory system diseases, and stroke as a cerebrovascular disease. In this paper, the term CVD will be used to refer to cardiovascular (coronary heart disease, myocardial infarction) and/or cerebrovascular disease (stroke) to allow for more readable text.

For peer review only - http://bmjopen.bmj.com/site/about/guidelines.xhtml

as compared with heart disease. [14,15]

Limited studies have examined health risk perceptions of SHS. Single-country cross-sectional studies have found variation in knowledge of diseases caused by SHS across countries. Few studies have examined the risk perceptions of SHS. For example, studies from Canada [16] and the United Kingdom (UK) [17] have found high awareness that SHS is a risk factor for cancer among adults in the general population. In China, adults in the general population reported higher knowledge that SHS causes lung cancer compared with heart disease and respiratory disease.[18] Similarly, adult males who smoke in Malaysia had limited knowledge for the risks of heart disease and respiratory diseases from SHS.[19] In contrast, adults who do not smoke in Nigeria had high awareness that SHS causes cancer, cardiovascular disease, and respiratory disease.[20] These country differences may reflect contextual determinants of SHS exposure ─ such as the adoption of smoke-free homes, implementation and enforcement of smoking bans in public places, and SHS mass media campaigns [21–24] ─ which may in turn, influence knowledge of SHS-caused diseases.

Few studies have examined changes in knowledge about smoking- and SHS-caused diseases. A repeated cross-sectional study found significant increases from 1999 to 2006 in risk perceptions of SHS for lung cancer and heart disease among adults of the Irish general adult population.[25] One Global Adult Tobacco Survey (GATS) study in six countries between 2008 and 2016, found significant increases in knowledge that smoking causes lung cancer, heart attack, and stroke among persons aged 15+ years who smoke.[15]

For peer review only - http://bmjopen.bmj.com/site/about/guidelines.xhtml

This study assessed knowledge and changes in knowledge over time about key chronic diseases caused by smoking tobacco and SHS exposure among adults who smoke across 46 countries. Indicators included knowledge that smoking causes lung cancer, heart disease/heart attack, and stroke in people who smoke, and that SHS causes lung cancer and heart disease/heart attack in people who do not smoke.

METHODS

Study design and participants

Data were from the ITC Project and the Global Adult Tobacco Survey System (GTSS) (GATS).

ITC Project longitudinal cohort surveys with replenishment are administered in 31 countries using a standardised set of questions, data collection protocol, and sampling design that allows for cross-country comparisons. For this study, we used ITC Project Survey data from nationally representative samples of adults (aged 18+ years) who smoke tobacco in 26 countries between 2002 and 2022: Australia, Bangladesh, Brazil, Canada, China, England/UK[c], France, Germany, Greece, Hungary, India, Japan, Kenya, Malaysia, Mauritius, Mexico, the Netherlands, New Zealand, Poland, Republic of Korea, Romania, Spain, Thailand, the US, Uruguay, and Zambia (Supplementary table 1). Adults who smoke tobacco were defined as persons aged 18+ years who smoke tobacco, including those who currently smoke manufactured and/or roll-your own cigarettes at least monthly and had smoked at least 100 cigarettes in their lifetime (all

[c] Caution is warranted in the comparison of ITC Survey data from the United Kingdom (which includes England, Wales, Scotland, and Northern Ireland;2002 to 2010–11) and England (2020). However, England accounts for ~85% of the population of the United Kingdom, and smoking prevalence and tobacco control policies are similar across all four countries that comprise the United Kingdom. As such, we combined data from the United Kingdom and England for analysis, as approved by ITC Project Principal Investigators.

For peer review only - http://bmjopen.bmj.com/site/about/guidelines.xhtml

countries), and/or currently smoke bidis at least monthly (India, Bangladesh, and Zambia).

GATS is a nationally representative repeated cross-sectional household survey of persons aged 15+ years that uses a standard core questionnaire, sample design, and data collection methods. GATS data included in this study are from adults who smoke tobacco in 32 countries between 2008 and 2020: Argentina, Bangladesh, Botswana, Brazil, Cameroon, China, Costa Rica, Egypt, Ethiopia, Greece, India, Indonesia, Kazakhstan, Kenya, Malaysia, Mexico, Nigeria, Pakistan, Panama, Philippines, Poland, Qatar, Romania, Russian Federation, Senegal, Tanzania, Thailand, Turkey, Uganda, Ukraine, Uruguay, and Viet Nam (Supplementary table 2). For consistency in analysis across GATS and ITC, an age cut-off of 18+ years was used to define adults who smoke combustible tobacco products (bidis, kreteks, cheroots, manufactured cigarettes, hand-rolled cigarettes, cigars, cigarillos, pipes, waterpipe with tobacco).

Data on knowledge of smoking- and SHS-caused diseases were collected from adults who smoked tobacco and participated in at least one wave of the ITC Surveys (N=133,410) and/or the GATS (N=110,537). We used data from all survey years to test for significant change in knowledge, and data from the first and last survey waves to compute average yearly change in knowledge. Countries included in the ITC Surveys and/or GATS are listed in Supplementary table 3. Sample demographics and methodological details for ITC Surveys are described elsewhere[26] and in online individual country technical reports.[27] Methodological details for the GATS and individual country fact sheets are available online.[28]

For peer review only - http://bmjopen.bmj.com/site/about/guidelines.xhtml

This study includes data from 26 countries of the International Tobacco Control (ITC) Project (2002-2022) that are available upon reasonable request [dataset] [29], and data from 32 countries of the Global Adult Tobacco Survey (GATS) (2008-2020) that are available in a public, open access repository: https://www.gtssacademy.org/. [dataset][28] Approval for use of ITC Survey data was granted by ITC Project Principal Investigators on July 28, 2023 , and approval for use GATS data was granted by theUS Centers for Disease Control and Prevention Office on Smoking and Health approval on May 1, 2023.. Data from ITC and GATS countries that were collected and/or became publicly available after these dates were excluded from analyses. All data relevant to the study are included in the article or uploaded as supplementary information.

Measures

Knowledge about smoking-caused diseases was assessed by asking participants if they know or believe that smoking causes lung cancer, heart disease/heart attack, and stroke. Respondents were also asked if they know or believe that SHS causes lung cancer and heart disease/heart attack in people who do not smoke. Responses were coded as 'yes' vs. 'no/don't know'. Response 'refused' was excluded from the analysis. ITC Survey and GATS questions and response options are presented in Supplementary table 4. Questions for all five disease outcomes were not included at every survey wave across all 26 ITC and 32 GATS countries. We used the most recent data available for each country to calculate point estimates and all-country means of the prevalence of knowledge for each disease outcome, and all longitudinal data available for each country to assess changes in knowledge over time.

Statistical analysis

For peer review only - http://bmjopen.bmj.com/site/about/guidelines.xhtml

Point estimates of the prevalence of knowledge for each disease outcome by country were calculated using weighted multinomial survey logistic regression models for pooled repeated cross-sectional GATS data, and generalized estimating equations for pooled longitudinal ITC data. All models incorporated survey design information (strata and primary sampling units for multi-stage sampling surveys), adjusted for sex, age, smoking status (daily, non-daily), and time-in-sample (ITC countries).

Point estimates of all-country means of the prevalence of knowledge for each disease outcome across all ITC countries and all GATS countries, respectively, were calculated using a multi-level model (two levels: individual and country) with random intercepts at the country-level and with roughly equal weights for each country. These point estimates cannot be used to make inferences to the global population. As such, we report the estimated all-country means of knowledge for each health effect without the associated confidence intervals, which would refer to a hypothetical superpopulation of countries. The all-country mean estimates in this analysis therefore only serve as the central point of selected countries. GEE and survey logistic regression models were used to test statistical differences in comparing knowledge between the five health effects among respondents in ITC and GATS countries, respectively.

We estimated changes in knowledge for each outcome among adults who smoke in countries with at least two waves of data between 2002 and 2022 (ITC Project Surveys), and between 2008 and 2020 (GATS). Analysis of the longitudinal ITC data used generalized estimating equations to account for within-subject correlations given that a proportion of individuals participated in more than one survey. Analysis of the repeated cross-sectional GATS data used weighted multinomial survey logistic

For peer review only - http://bmjopen.bmj.com/site/about/guidelines.xhtml

regression models. Two-level models (individual and country) with random intercepts and random slopes of survey years at the country-level were used to test for significant yearly change in knowledge for each health effect within countries. This modelling approach controls for the number of years between the first and last survey waves, and assumed a linear survey year effect within each country.

Data from ITC and GATS countries were pooled and fitted separately to ensure comparability within ITC countries and within GATS countries. Pearson correlations (r) between ITC and GATS mean prevalence estimates of knowledge for each of the five diseases were used to examine consistency of estimates from different surveys conducted in the 12 overlapping countries.

The predicted marginal standardization method (PREDMARG) was used to generate all marginal estimates of the outcomes. Data analysis were conducted using SAS v9.4 and SAS-callable SUDAAN v11.0.3. Statistical significance ($p < .05$ considered statistically significant) and confidence intervals were computed at the 95% confidence level.

RESULTS

Knowledge about diseases caused by smoking and SHS exposure

Knowledge about diseases caused by smoking among adults who smoke was highest for lung cancer (ITC=92.2%, GATS=91.9%), followed by heart disease (ITC=84.7%)/heart attack (GATS=80.0%), and stroke (ITC=75.0%, GATS=66.1%) (Supplementary table 5). Knowledge that SHS causes lung cancer among people who do not smoke (ITC=77.3%, GATS=82.1%) was higher than for SHS causes heart attack (ITC=61.8%)/heart disease (GATS=72.5%) in people who do not smoke. All differences

For peer review only - http://bmjopen.bmj.com/site/about/guidelines.xhtml

between knowledge about the five diseases caused by smoking and SHS exposure were significant at $p < .001$.

Knowledge that active smoking causes lung cancer, heart disease/heart attack, and stroke

At the most recent survey across 25 ITC countries, 92.2% of adults who smoke overall knew that smoking causes lung cancer, ranging from 98.2% in Bangladesh (2014–15) to 78.1% in Zambia (2014) (Figure 1). Across all ITC countries, knowledge that smoking causes lung cancer decreased by 0.7 percentage points (PP) per year. Knowledge increased significantly in three countries: China (+1.2 PP per year), the Netherlands (+0.9 PP per year), and Bangladesh (+0.6 PP per year). Knowledge decreased significantly in three countries: Japan (-2.0 PP per year), Germany (-0.6 PP per year), and France (-0.5 PP per year) (Figure 1). There was no significant change in knowledge in all other countries. Detailed results for longitudinal trends in knowledge about smoking-caused diseases in ITC countries are provided in Supplementary table 7.

Figure 1. Percentage of adults who smoke who know or believe that smoking causes lung cancer,† in 25 ITC countries,‡ 2002–2022.

At the most recent survey across 32 GATS countries, 91.9% of adults who smoke overall knew that smoking causes lung cancer, ranging from 98.4% in Argentina (2012) to 56.6% in Nigeria (2012) (Figure 2). Across the 12 GATS countries with at least two years of data, knowledge that smoking causes lung cancer decreased by 0.2 PP per year. Knowledge increased significantly in two countries: India (+0.8 PP per year), and Bangladesh (+0.5 PP per year). Knowledge decreased significantly in three countries:

For peer review only - http://bmjopen.bmj.com/site/about/guidelines.xhtml

Romania (–1.3 PP per year), the Russian Federation (–1.2 PP per year), and Turkey (–0.4 PP per year) (Figure 2). There was no significant change in knowledge in all other countries. For the 20 GATS countries with only one year of data, the change is presented as zero. Detailed results for longitudinal trends in knowledge about smoking-caused diseases in GATS countries are provided Supplementary table 8.

Figure 2. Percentage of adults who smoke who know or believe that smoking causes lung cancer, in 32 GATS countries,† 2008–2018.

At the most recent survey across 25 ITC countries, 84.7% of adults who smoke knew that smoking causes heart disease, ranging from 94.9% in Bangladesh (2014–15) to 57.7% in China (2013–15) (Figure 3). Across all ITC countries, knowledge that smoking causes heart disease decreased by 0.3 PP per year. Knowledge increased significantly in five countries: China (+2.2 PP per year), Kenya (+2.2 PP per year), India (+1.2 PP per year), Bangladesh (+1.2 PP per year), and Spain (+1.1 PP per year). Knowledge decreased significantly in four countries: Japan (–4.3 PP per year), Germany (–1.4 PP per year), Uruguay (–0.7 PP per year), and France (–0.5 PP per year) (Figure 3). There was no significant change in knowledge in all other countries.

Figure 3. Percentage of adults who smoke who know or believe that smoking causes heart disease,† in 25 ITC countries,‡ 2002–2022.

At the most recent survey across 32 GATS countries, 80.0% of adults who smoke knew that smoking causes heart attack, ranging from 95.3% in Turkey (2016) to 46.4% in China (2018) (Figure 4). Across all GATS countries with at least two years of data, knowledge that smoking causes heart attack increased by 0.5 PP per year. Knowledge increased significantly in four countries: Viet Nam (+2.2 PP per year), India (+1.9 PP per

For peer review only - http://bmjopen.bmj.com/site/about/guidelines.xhtml

year), the Philippines (+1.0 PP per year), and Bangladesh (+0.6 PP per year). Knowledge decreased significantly in Romania (–2.1 PP per year) (Figure 4). There was no significant change in knowledge in all other countries.

Figure 4. Percentage of adults who smoke who know or believe that smoking causes heart attack, in 32 GATS countries,† 2008–2018.

At the most recent survey across 25 ITC countries, 75.0% of adults who smoke knew that smoking causes stroke, ranging from 93.9% in Bangladesh (2014–15) to 37.3% in China (2013–15) (Figure 5). Across all ITC countries, knowledge that smoking causes stroke increased by 1.6 PP per year. Knowledge increased significantly in 11 countries: Thailand (+5.7 PP per year), Greece (+5.5 PP per year), Mauritius (+4.6 PP per year), Hungary (+4.5 PP per year), Kenya (+3.9 PP per year), Uruguay (+2.8 PP per year), China (+2.5 PP per year), Mexico (+2.2 PP per year), Republic of Korea (+2.1 PP per year), the Netherlands (+1.4 PP per year), and India (+1.3 PP per year). Knowledge decreased significantly in Germany (–1.0 PP per year) (Figure 5). There was no significant change in knowledge in all other countries.

Figure 5. Percentage of adults who smoke who know or believe that smoking causes stroke,† in 25 ITC countries,‡ 2002–2022.

At the most recent survey across 32 GATS countries, 66.1% of adults who smoke overall knew that smoking causes stroke, ranging from 94.1% in Bangladesh (2017) to 29.6% in Ethiopia (2016) (Figure 6). Across all GATS countries with at least two years of data, knowledge that smoking causes stroke increased by 0.5 PP per year. Knowledge increased significantly in five countries: India (+2.3 PP per year), China (+1.3 PP per year), the Russian Federation (+1.2 PP per year), and Mexico (+1.2 PP

For peer review only - http://bmjopen.bmj.com/site/about/guidelines.xhtml

per year), and Bangladesh (+1.0 PP per year). Knowledge decreased significantly in Romania (–2.0 PP per year) (Figure 6). There was no significant change in knowledge in all other countries.

Figure 6. Percentage of adults who smoke who know or believe that smoking causes stroke, in 32 GATS countries,† 2008–2018.

Knowledge about the risks of SHS exposure causing lung cancer and heart disease/heart attack

At the most recent survey across 26 ITC countries, 77.3% of adults who smoke knew that SHS causes lung cancer in people who do not smoke, ranging from 96.8% in Thailand (2012) to 58.4% in Germany (2018) (Figure 7). Across all ITC countries, knowledge that SHS causes lung cancer in people who do not smoke decreased by 0.1 PP per year. Knowledge increased significantly in five countries: Australia (+2.2 PP per year), China (+2.0 PP per year), India (+1.7 PP per year), Bangladesh (+1.4% PP per year), and Thailand (+0.8 PP per year). Knowledge decreased significantly in four countries: Germany (–2.0 PP per year), New Zealand (–1.5 PP per year), France (–1.5 PP per year), and Uruguay (–1.2 PP per year) (Figure 7). There was no significant change in knowledge in all other countries. Detailed results for longitudinal trends in knowledge about SHS-caused diseases in ITC countries are provided in Supplementary table 9.

Figure 7. Percentage of adults who smoke who know or believe that secondhand smoke causes lung cancer, in 26 ITC countries,† 2003–2022.

At the most recent survey across 18 GATS countries, 82.1% of adults who smoke knew that SHS causes lung cancer in people who do not smoke, ranging from 96.4% in

For peer review only - http://bmjopen.bmj.com/site/about/guidelines.xhtml

Thailand (2011) to 41.9% in Nigeria (2012) (Figure 8). Across all GATS countries with at least two years of data, there was no change in knowledge that SHS causes lung cancer in people who do not smoke. Knowledge increased significantly in Viet Nam (+2.7 PP per year) (Figure 8). There was no significant change in knowledge in all other countries. Detailed results for longitudinal trends in knowledge about SHS-caused diseases in GATS countries are provided in Supplementary table 10.

Figure 8. Percentage of adults who smoke who know or believe that secondhand smoke causes lung cancer, in 18 GATS countries,† 2009–2018.

In the most recent survey administered across 24 ITC countries, 61.8% of adults who smoke knew that SHS causes heart attack in people who do not smoke, ranging from 88.5% in Bangladesh (2014–15) to 38.4% in Australia (2013) (Figure 9). Across all ITC countries, knowledge that SHS causes heart attack in people who do not smoke increased by 0.2 PP per year. Knowledge increased significantly in five countries: Bangladesh (+7.0 PP per year), India (+2.7 PP per year), the Netherlands (+1.6 PP per year), Kenya (+2.2 PP per year), and China (+2.1 PP per year). Knowledge decreased significantly in four countries: Australia (–3.9 PP per year), Malaysia (–3.3 PP per year), Japan (–2.7 PP per year), and New Zealand (–2.5 PP per year) (Figure 9). There was no significant change in knowledge in all other countries.

Figure 9. Percentage of adults who smoke who know or believe that secondhand smoke causes heart attack,† in 24 ITC countries,‡ 2008–2022.

At the most recent survey across 18 GATS countries, 72.5% of adults who smoke knew that SHS causes heart disease in people who do not smoke, ranging from 93.5% in Bangladesh (2017) to 21.3% in Viet Nam (2015) (Figure 10). Across all GATS

For peer review only

countries with at least two years of data, knowledge that SHS causes heart disease in people who do not smoke decreased by 0.1 PP per year. Knowledge increased significantly in Viet Nam (+1.4 PP per year) (Figure 10). There was no significant change in knowledge in all other countries.

Figure 10. Percentage of adults who smoke who know or believe that secondhand smoke causes heart disease, in 18 GATS countries,† 2009–2018.

Comparison of knowledge levels among respondents in ITC and GATS countries

Compared with overall knowledge that smoking causes lung cancer (ITC=92.2%, GATS=91.9%), knowledge that smoking causes heart attack (ITC=84.7%)/heart disease (GATS=80.0%) and stroke (ITC=75.0%, GATS=66.1%) was lower, as was knowledge about SHS harms for people who do not smoke (SHS causes lung cancer: ITC=77.3%, GATS=82.1%; SHS causes heart attack (ITC=61.8%)/heart disease (GATS=72.5%) (all $p<0.0001$) (Supplementary table 5).

Knowledge that smoking causes stroke was the only one of the five harms where there was an increase in both ITC and GATS over time (+1.6 PP in ITC; +0.5 PP in GATS). Knowledge that smoking causes lung cancer was the only health harm where there was a decrease in both ITC and GATS (–0.7 PP in ITC; –0.2 PP in GATS). The average of estimated net change in knowledge across the five health harms was +0.14 PP in both ITC and GATS (Supplementary table 6).

Correlation of ITC and GATS data

In the 12 overlapping ITC and GATS countries, significant positive correlations were found between ITC and GATS estimates of knowledge among adults who smoke that smoking causes lung cancer ($r=.80$, $p=.002$), heart disease/heart attack ($r=.81$,

For peer review only - http://bmjopen.bmj.com/site/about/guidelines.xhtml

p=.001), and stroke (r=.80, p=.002). For the seven overlapping ITC and GATS countries where knowledge of SHS harms was assessed, moderate but non-significant correlations were found between ITC and GATS estimates of knowledge that SHS causes lung cancer (r=.69, p=.09) and heart disease/heart attack (r=.60, p=.16) in people who do not smoke.

DISCUSSION

Overall, there was a strong correlation between ITC and GATS knowledge estimates in countries where both surveys were conducted. There were five key findings from our analyses about knowledge of smoking- and SHS-caused diseases in 46 countries across two decades. First, adults who smoke were more likely to know that smoking elevates their risk for lung cancer compared with heart disease/heart attack and stroke. Second, adults who smoke were less likely to know about the risks related to SHS. Third, adults who smoke had higher knowledge that SHS causes lung cancer compared with heart disease/heart attack in people who do not smoke. Fourth, knowledge for smoking- and SHS-caused diseases has improved in several countries over time; however, knowledge has generally remained unchanged in most countries and has regressed in others. Finally, there was a near-zero net change over time in knowledge about smoking- and SHS-caused diseases (+0.14% per year across five disease outcomes in ITC and GATS countries).

There has been a steady upward trend in the global burden of CVD from 1990 to 2019 ─ with the total number of CVD deaths rising from 12.1 million to 18.6 million, and the total number of disability-adjusted life years (DALYs) due to CVD rising from 17.7 million to 34.4 million over the last three decades.[30] The largest number of premature

For peer review only - http://bmjopen.bmj.com/site/about/guidelines.xhtml

non-communicable disease (NCD) deaths globally are caused by CVDs [31,32]. for which smoking is a major modifiable risk factor. In 2019, heart disease and stroke collectively accounted for 2.6 million smoking-attributable deaths worldwide, compared to 1.3 million deaths from lung, tracheal, and bronchus cancer.[33] A 2024 study found that exposure to SHS significantly increased risk for nine health outcomes, with the greatest risk observed for CVD.[34] Although smoking and SHS are known risk factors for CVD, adults in many countries still lack knowledge about their harmful effects on cardiovascular health. Consistent with previous studies [12,14,15], we found that adults who smoke were more likely to know that smoking causes lung cancer than heart disease and stroke. CVD is still the leading preventable cause of mortality worldwide [30,35] and while the relative risk from smoking for CVD is lower than for lung cancer, more people die prematurely from CVD than from lung cancer caused by smoking.[35,36]

SHS is one of the leading causes of preventable death [37], killing an estimated 1.3 million people who do not smoke in 2021. [38] We found that across 40 countries, about 65.0% of adults who smoke knew that SHS exposure causes heart disease/heart attack in people who do not smoke. Notably, even in several high-income ITC countries ─ including Australia, Germany, England, the US, Aotearoa/New Zealand, Canada and Hungary ─ less than half of adults who smoke knew of this risk, in contrast with higher knowledge that active smoking causes heart disease and stroke.

Knowledge that smoking causes stroke significantly increased in 11 ITC countries and three GATS countries between 2002 and 2022. Nevertheless, there were no significant changes in knowledge about stroke in 13 ITC countries and seven GATS

For peer review only - http://bmjopen.bmj.com/site/about/guidelines.xhtml

countries. Likewise, there were no significant changes in knowledge among adults who smoke that smoking causes heart disease/heart attack in people who smoke, and that SHS exposure causes heart disease/heart attack in people who do not smoke in 14 ITC countries and 5 GATS countries, respectively. While significant gains in knowledge about the CVD risks of smoking tobacco and/or SHS exposure were observed in several LMICS such as Bangladesh, India, Thailand, and Viet Nam, knowledge levels have remained low in others such as Nigeria and Ethiopia. Significant declines in knowledge about some of these risks were also observed in several high-income countries, where baseline risk knowledge was relatively high, including Germany and Japan. This suggests that measures to educate people about the diseases caused by smoking may have a greater impact in countries where knowledge is low compared with those where knowledge is already high. It is key for countries to tailor interventions to address knowledge gaps.

Country-level differences observed may reflect differences in public health education strategies and implementation methods about the harms of smoking. For example, health warnings on cigarette packs, size of warnings, and languages used are one way of informing people about diseases caused by smoking, which may encourage those who smoke to reduce consumption or take steps to quit.[39–41] It is possible that people who smoke may have higher levels of knowledge for specific health risks of smoking that were depicted on cigarette pack warnings at the time of ITC and GATS country surveys, compared to health risks that were not addressed by the warnings. However, knowledge alone may be insufficient to change behavior. Social Cognitive Theory provides one theoretical framework for understanding the underlying

For peer review only - http://bmjopen.bmj.com/site/about/guidelines.xhtml

mechanisms of health behaviors and informing the development of interventions, whereby behavior is determined by reciprocal interactions between personal, environmental, and behavioral factors. [42] For example, exposure to health warnings on cigarette packs in combination with knowledge of smoking- and SHS-caused diseases, self-efficacy to reduce or quit smoking, supportive environments such as smoke-free homes, workplaces, and public places, can facilitate smoking cessation and protection against SHS exposure. Future research should examine the associations between knowledge of smoking-caused diseases and health warnings (type (text-only or pictorial), content, size, and location (front and/or back of pack)) as well as other tobacco control policies, which was beyond the scope of the current study.

Since the WHO FCTC came into force in 2005, countries around the world have made significant progress in educating their populations about the dangers of tobacco use and SHS exposure through the implementation of tobacco advertising, promotion and sponsorship bans,[43] health warnings on tobacco packaging,[44] smoke-free laws,[45] and anti-tobacco campaigns,[44] which have likely contributed to reductions in the tobacco use prevalence from 29.3% in 2005 to 20.9% in 2022.[45,46] However, we found no overall change (+0.14% per year) in knowledge across five smoking and SHS-caused diseases in both ITC and GATS countries over a two-decade period, even though 43 of the 46 countries included in the present study are WHO FCTC Parties.[47] This may reflect an uneven pace in the implementation of MPOWER measures—including taxation, health warnings on cigarette packs and mass media campaigns ─ that are especially effective for communicating smoking-caused risks [45] and most impactful when they are integrated as a part of a comprehensive tobacco control

For peer review only - http://bmjopen.bmj.com/site/about/guidelines.xhtml

strategy. [48] For example, the impact of smoke-free laws on knowledge about SHS-caused diseases could potentially be strengthened by complementary mass media campaigns, which have been shown to increase knowledge of SHS risks, reduce smoking in homes, and promote smoking cessation. [23]

Tobacco control policies may be more effective at improving knowledge about smoking and SHS-caused diseases when supported by strong implementation, enforcement, and compliance; however, substantial gaps remain. Studies have found weak implementation, limited accountability, and poor enforcement of smoke-free laws in LMICs after WHO FCTC ratification,[49] and a 2024 systematic review and meta-analysis reported high levels of non-compliance with smoke-free laws, particularly in public places and workplaces.[50] These findings align with an assessment of the WHO FCTC at its 20th anniversary, which concluded that progress has slowed despite earlier gains, due to tobacco industry interference, limited political will and resources, and the rise of new nicotine products, highlighting the need for best-practice implementation, regulation, and intergovernmental cooperation. [51]

It is also important to note that reductions in mortality and morbidity from smoking- and SHS-caused diseases may influence public perceptions of their harms, particularly in countries with declining smoking prevalence. Moreover, advances in healthcare and effective treatments in high-resource countries could reduce the disease burden and improve quality of life even in the absence of smoking prevention policies, potentially contributing to low population knowledge about the harms of smoking and SHS. This underscores the importance of prioritizing resources for tobacco use prevention rather than focusing solely on treatment.

For peer review only - http://bmjopen.bmj.com/site/about/guidelines.xhtml

This study has limitations. First, self-reported data may be subject to recall bias that might result in over- or under-estimation of knowledge or social desirability bias. Second, some ITC survey response rates were low due to the nature of the mode and/or protocols for data collection (Supplementary table 1), which may limit the generalizability of the study findings. Third, we found statistically significant positive correlations for all three smoking-caused diseases in the 12 overlapping ITC and GATS countries, while non-significant correlations were found for the two SHS-caused diseases due to reduced power from the small sample size (N=7 observations). Caution is warranted in the comparison of ITC and GATS estimates due to differences in research designs, sampling across ITC Surveys, and between ITC Surveys and GATS, survey year(s) in overlapping ITC and GATS countries, and ITC Survey and GATS questions used to measure knowledge (Supplementary table 1, Supplementary table 2, Supplementary table 4). However, correlation of the ITC and GATS data despite their different research and methodological designs, allows for more confident inferences.[52][53] Fourth, ITC and GATS data correlation analyses were limited to 12 countries, so the results are not generalizable. Finally, our results are limited to knowledge about the harms of smoking among adults who currently smoke tobacco. Our finding for an overall near-zero net change in knowledge over time may or may not reflect changes in overall knowledge in the population. It is possible that while knowledge about diseases caused by smoking and SHS among adults who smoke did not change over time, knowledge may have increased among adults who have quit smoking and those who have never smoked. As such, we examined whether changes in knowledge for each disease outcome differed by smoking status across 12 GATS countries where more than one

For peer review only - http://bmjopen.bmj.com/site/about/guidelines.xhtml

survey wave was conducted among adults who smoke, adults who have quit smoking, and adults who have never smoked.[d] In general, there were no consistent patterns for changes in knowledge about each disease outcome across the 12 GATS countries (Supplementary table 5). Non-significant increases/decreases in knowledge over time among adults who quit and those who never smoked suggests that this pattern smokers is consistent with that in the overall population.

This study found high knowledge that smoking causes lung cancer among adults who smoke, lower knowledge that smoking causes CVD, and even lower knowledge for SHS harms. There was no overall change in knowledge over two decades. The United Nations 2030 Agenda for Sustainable Development calls for strengthening implementation of the WHO FCTC to achieve the target for a one-third reduction in premature mortality from NCDs by 2030.[54] To achieve this, countries could consider implementation of WHO FCTC measures shown to have synergistic effects on promoting smoking cessation and reducing smoking prevalence.[55,56] An important factor that can facilitate efforts to strengthen policies is to increase the awareness of the harms of using cigarettes and other smoked tobacco products.

CONCLUSION

Across 46 countries over two decades, adults who smoke had high knowledge that smoking causes lung cancer, substantially lower knowledge of cardiovascular harms,

[d]ITC longitudinal cohort surveys primarily recruit adults who smoke at baseline. Respondents who quit smoking at subsequent survey waves may be recontacted as part of the longitudinal cohort; however, these individuals do not constitute a population-representative sample of adults who have quit smoking. Although these respondents retain adults who smoke-based survey weights for longitudinal analyses (to represent the previous-wave population of adults who smoke), they are analytically classified as adults who quit and are not included as adults who currently smoke in our analyses. For this reason, analyses comparing changes in knowledge by smoking status (adults who currently, formerly, and never smoked) were conducted using GATS data only.

For peer review only - http://bmjopen.bmj.com/site/about/guidelines.xhtml

and even lower awareness of SHS-caused diseases. The near-zero net change in knowledge over time underscores the need for comprehensive, well-implemented, and enforced tobacco control strategies ─ particularly health warnings, mass media campaigns, and smoke-free policies ─ to address knowledge gaps and accelerate progress toward global NCD prevention targets.

For peer review only

For peer review only - http://bmjopen.bmj.com/site/about/guidelines.xhtml

Competing interest statement: KMC has in the past and continues to serve as a paid witness in litigation filed against cigarette manufacturers. GTF has been an expert witness or consultant for governments defending their country's policies or regulations in litigation. CG has been a paid expert witness for the Australian Government in litigation concerning nicotine product marketing. KK received a JMWH Bayer Grant from Japan Society for Menopause and Women's Health. ASAN has received an unconditional educational grant from Johnson & Johnson Malaysia Sdn. Bhd. JFT has served as paid expert witness in legal challenges against tobacco and vaping companies. All other authors have no conflicts of interest to declare.

For peer review only

References

1 Murray CJL, Aravkin AY, Zheng P, *et al.* Global burden of 87 risk factors in 204 countries and territories, 1990–2019: a systematic analysis for the Global Burden of Disease Study 2019. *The Lancet*. 2020;396:1223–49. doi: 10.1016/S0140-6736(20)30752-2

2 World Health Organization. WHO Framework Convention on Tobacco Control. 2003.

3 World Health Organization. MPOWER. A policy package to reverse the tobacco epidemic. Geneva 2008.

4 Hammond D. Health warning messages on tobacco products: a review. *Tob Control*. 2011;20:327–37. doi: 10.1136/tc.2010.037630

5 World Health Organization. Tackling NCDs: 'best buys' and other recommended interventions for prevention and control of noncommunicable diseases. Geneva 2017.

6 Brinn M, Carson K, Esterman A, *et al.* Mass media interventions for preventing smoking in young people. *Cochrane Database of Systematic Reviews*. 2010;10:Art. No.: CD001006. doi: 10.1002/14651858.CD001006.pub3

7 Evans KA, Sims M, Judge K, *et al.* Assessing the knowledge of the potential harm to others caused by second-hand smoke and its impact on protective behaviours at home. *J Public Health*. 2012;34:183–94. doi: 10.1093/pubmed/fdr104

8 Yang J, Hammond D, Driezen P, *et al.* Health knowledge and perception of risks among Chinese smokers and non-smokers: findings from the Wave 1 ITC China Survey. *Tob Control*. 2010;19:i18–23. doi: 10.1136/TC.2009.029710

9 Demissie HS, Smith T, de Quevedo IG, *et al.* Factors associated with quit attempt and successful quitting among adults who smoke tobacco in Ethiopia: Global Adult Tobacco Survey (GATS) 2016. *Tob Prev Cessat*. 2022;8:12. doi: 10.18332/tpc/146170

10 Hyland A, Li Q, Bauer JE, *et al.* Predictors of cessation in a cohort of current and former smokers followed over 13 years. *Nicotine Tob Res*. 2004;6:S363-9.

11 Chiosi JJ, Andes L, Asma S, *et al.* Warning about the harms of tobacco use in 22 countries: findings from a cross-sectional household survey. *Tob Control*. 2016;25:393–401. doi: 10.1136/tobaccocontrol-2014-052047

12 Siahpush M, McNeill A, Hammond D, *et al.* Socioeconomic and country variations in knowledge of health risks of tobacco smoking and toxic constituents of smoke: results from the 2002 International Tobacco Control (ITC) Four Country Survey. *Tob Control*. 2006;15:iii65–70. doi: 10.1136/TC.2005.013276

13 Mills SD, Wiesen CA. Beliefs about the health effects of smoking among adults in the United States. *Health Educ Behav*. 2022;49:497–505. doi: 10.1177/10901981211004136

14 Trofor AC, Papadakis S, Lotrean LM, *et al.* Knowledge of the health risks of smoking and impact of cigarette warning labels among tobacco users in six European countries: Findings

For peer review only

from the EUREST-PLUS ITC Europe Surveys. *Tob Induc Dis*. 2018;16. doi: 10.18332/tid/99542

15 Ahluwalia I, Smith T, Arrazola R, *et al.* Current tobacco smoking, quit attempts, and knowledge about smoking risks among persons aged ≥15 Years - Global Adult Tobacco Survey, 28 Countries, 2008-2016. *MMWR*. doi: 10.15585/MMWR.MM6738A7

16 Rydz E, Telfer J, Quinn EK, *et al.* Canadians' knowledge of cancer risk factors and belief in cancer myths. *BMC Public Health*. 2024;24:329. doi: 10.1186/s12889-024-17832-3

17 Shahab L, McGowan JA, Waller J, *et al.* Prevalence of beliefs about actual and mythical causes of cancer and their association with socio-demographic and health-related characteristics: findings from a cross-sectional survey in England. *Eur J Cancer*. 2018;103:308–16. doi: 10.1016/j.ejca.2018.03.029

18 Sun Y, Wang D, Yi Y, *et al.* Outdoor secondhand smoke exposure in public places frequented by minors in the urban area of Hangzhou City, China: a cross-sectional study. *Tob Induc Dis*. 2024;22. doi: 10.18332/tid/192129

19 Abdul Mutalib RNS, Abd Rani NL, Zulkifli A, *et al.* Knowledge, beliefs, and behaviors related to secondhand smoke and smoking in the home: a qualitative study with men in Malaysia. *Nicotine Tob Res*. 2023;25:821–7. doi: 10.1093/ntr/ntac239

20 Ezekiel E, Tomini S, Odukoya O. Secondhand smoke exposure, perceived risks and knowledge of the national tobacco law among non-smoking adults in outdoor motor parks in an urban area, Lagos, Nigeria. *Tob Induc Dis*. ;16:A491. doi: 10.18332/tid/84641

21 Tattan-Birch H, Jarvis MJ. Children's exposure to second-hand smoke 10 years on from smoke-free legislation in England: cotinine data from the Health Survey for England 1998-2018. *Lancet Reg Health Eur*. 2022;15:100315. doi: 10.1016/j.lanepe.2022.100315

22 Nanninga S, Lhachimi SK, Bolte G. Impact of public smoking bans on children's exposure to tobacco smoke at home: a systematic review and meta-analysis. *BMC Public Health*. 2018;18:749. doi: 10.1186/s12889-018-5679-z

23 Lim CCW, Rutherford B, Gartner C, *et al.* A systematic review of second-hand smoking mass media campaigns (2002-2022). *BMC Public Health*. 2024;24:693. doi: 10.1186/s12889-024-18222-5

24 Chido-Amajuoyi OG, Osaghae I, Agaku IT, *et al.* Exposure to school-based tobacco prevention interventions in low-income and middle-income countries and its association with psychosocial predictors of smoking among adolescents: a pooled cross-sectional analysis of Global Youth Tobacco Survey data from 38 countries. *BMJ Open*. 2024;14:e070749. doi: 10.1136/bmjopen-2022-070749

25 Lonergan BJ, Meaney S, Perry IJ, *et al.* Smokers still underestimate the risks posed by secondhand smoke: a repeated cross-sectional study. *Nicotine Tob Res*. 2014;16:1121–8. doi: 10.1093/ntr/ntu046

For peer review only - http://bmjopen.bmj.com/site/about/guidelines.xhtml

26 Thompson ME, Driezen P, Boudreau C, *et al.* Methods of the International Tobacco Control (ITC) EUREST-PLUS ITC Europe Surveys. *Eur J Public Health*. 2020;30:iii4–9. doi: 10.1093/eurpub/ckz212

27 ITC Project. ITC Project. Methods. Technical reports. International Tobacco Control Policy Evaluation Project. Methods. 2024. https://itcproject.org/methods/technical-reports/

[dataset] 28 Global Tobacco Surveillance System. GTSS Academy. Global Tobacco Surveillance System. Academy. 2026. https://www.gtssacademy.org/

[dataset] 29 International Tobacco Control Policy Evaluation Project. Data request forms. 2026. https://itcproject.org/request-data-form/

30 Roth GA, Mensah GA, Johnson CO, *et al.* Global burden of cardiovascular diseases and risk factors, 1990-2019: update from the GBD 2019 Study. *J Am College Cardiology*. 2020;76:2982–3021. doi: 10.1016/j.jacc.2020.11.010

31 National Cancer Institute. Health effects of exposure to environmental tobacco smoke. *Smoking and Tobacco Control Monograph 10*. Bethesda, MD: National Cancer Institute 1999.

32 Barnoya J, Glantz SA. Cardiovascular effects of secondhand smoke: nearly as large as smoking. *Circulation*. 2005;111:2684–98. doi: 10.1161/CIRCULATIONAHA.104.492215

33 Institute for Health Metrics and Evaluation. GBD summaries. Secondhand smoke - Level 3 risk. 2019. https://www.healthdata.org/results/gbd_summaries/2019/secondhand-smoke-level-3-risk

34 Flor LS, Anderson JA, Ahmad N, *et al.* Health effects associated with exposure to secondhand smoke: a Burden of Proof study. *Nat Med*. 2024;30:149–67. doi: 10.1038/s41591-023-02743-4

35 Global Burden of Disease Collaborative Network. Global Burden of Disease Study 2019 (GBD 2019) results. Institute for Health Metrics and Evaluation. 2020. https://vizhub.healthdata.org/gbd-results/

36 Reitsma MB, Kendrick PJ, Ababneh E, *et al.* Spatial, temporal, and demographic patterns in prevalence of smoking tobacco use and attributable disease burden in 204 countries and territories, 1990–2019: a systematic analysis from the Global Burden of Disease Study 2019. *The Lancet*. 2021;397:2337–60. doi: 10.1016/S0140-6736(21)01169-7

37 Öberg M, Jaakkola MS, Woodward A, *et al.* Worldwide burden of disease from exposure to second-hand smoke: a retrospective analysis of data from 192 countries. *The Lancet*. 2011;377:139–46. doi: 10.1016/S0140-6736(10)61388-8

38 Brauer M, Roth G, Aravkin A, *et al.* Global burden and strength of evidence for 88 risk factors in 204 countries and 811 subnational locations, 1990-2021: a systematic analysis for the Global Burden of Disease Study 2021. *The Lancet*. 2024;403:2162–203. doi: 10.1016/S0140-6736(24)00933-4

39 World Health Organization. Tobacco control (TFI). 2021.

For peer review only - http://bmjopen.bmj.com/site/about/guidelines.xhtml

40 Noar S, Francis D, Bridges C, *et al.* The impact of strengthening cigarette pack warnings: systematic review of longitudinal observational studies. *Soc Sci Med*. 2016;164:118–29. doi: 10.1016/j.socscimed.2016.06.011

41 Ngo A, Cheng KW, Shang C, *et al.* Global evidence on the association between cigarette graphic warning labels and cigarette smoking prevalence and consumption. *IJERPH*. 2018;15:421. doi: 10.3390/IJERPH15030421

42 Mantey D, Hunt E, Hoelscher D, *et al. Social Cognitive Theory and health behaviors*. 2024.

43 World Health Organization. WHO report on the global tobacco epidemic, 2013: enforcing bans on tobacco advertising, promotion and sponsorship. Geneva: WHO 2013.

44 World Health Organization. *WHO report on the global tobacco epidemic, 2011: warning about the dangers of tobacco.* World Health Organization 2011.

45 World Health Organization. WHO report on the global tobacco epidemic, 2023: protect people from tobacco smoke. Geneva: World Health Organization 2023.

46 World Health Organization. WHO global report on trends in prevalence of tobacco use 2000–2030. Geneva: World Health Organization 2024.

47 World Health Organization. WHO Framework Convention on Tobacco Control. Parties. WHO Framework Convention on Tobacco Control. 2024. https://fctc.who.int/who-fctc/overview/parties

48 World Health Organization. WHO report on the global tobacco epidemic, 2025. Geneva: World Health Organization 2025.

49 Byron MJ, Cohen JE, Frattaroli S, *et al.* Implementing smoke-free policies in low- and middle-income countries: a brief review and research agenda. *Tob Induc Dis*. 2019;17:60. doi: 10.18332/tid/110007

50 Daba C, Atamo A, Gasheya KA, *et al.* Non-compliance with smoke-free law in public places: a systematic review and meta-analysis of global studies. *Front Public Health*. 2024;12:1354980. doi: 10.3389/fpubh.2024.1354980

51 Gilmore AB, Callard C, Sy D, *et al.* 20th anniversary of the WHO Framework Convention on Tobacco Control coming into force: time for a step change in ambition. *The Lancet*. 2025;405:677–81. doi: 10.1016/S0140-6736(25)00336-8

52 Hill AB. The environment and disease: association or causation? *Proc R Soc Med*. 1965;58:295–300.

53 Campbell DT, Fiske DW. Convergent and discriminant validation by the multitrait-multimethod matrix. *Psychological Bulletin*. 1959;56:81–105. doi: 10.1037/h0046016

54 United Nations. Transforming our world: the 2030 Agenda for Sustainable Development. 2015.

For peer review only - http://bmjopen.bmj.com/site/about/guidelines.xhtml

55 Chung-Hall J, Craig L, Gravely S, *et al.* Impact of the WHO FCTC over the first decade: a global evidence review prepared for the Impact Assessment Expert Group. *Tob Control*. 2019;28:s119–28. doi: 10.1136/tobaccocontrol-2018-054389

56 Hoffman SJ, Tan C. Overview of systematic reviews on the health-related effects of government tobacco control policies. *BMC Public Health*. 2015;15:744. doi: 10.1186/s12889-015-2041-6

For peer review only

For peer review only - http://bmjopen.bmj.com/site/about/guidelines.xhtml

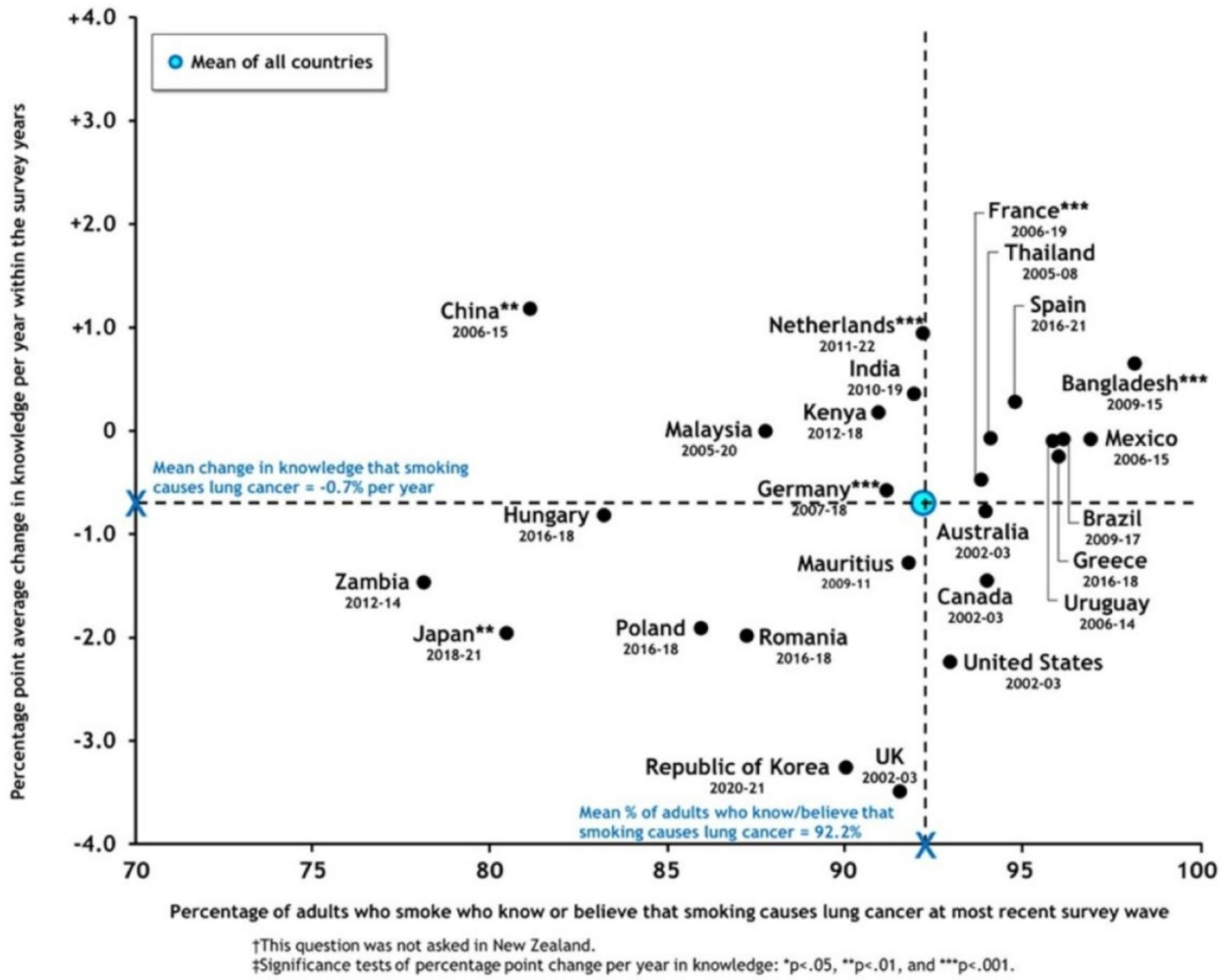

Figure 1. Percentage of adults who smoke who know or believe that smoking causes lung cancer,† in 25 ITC countries,‡ 2002–2022.

148x129mm (300 x 300 DPI)

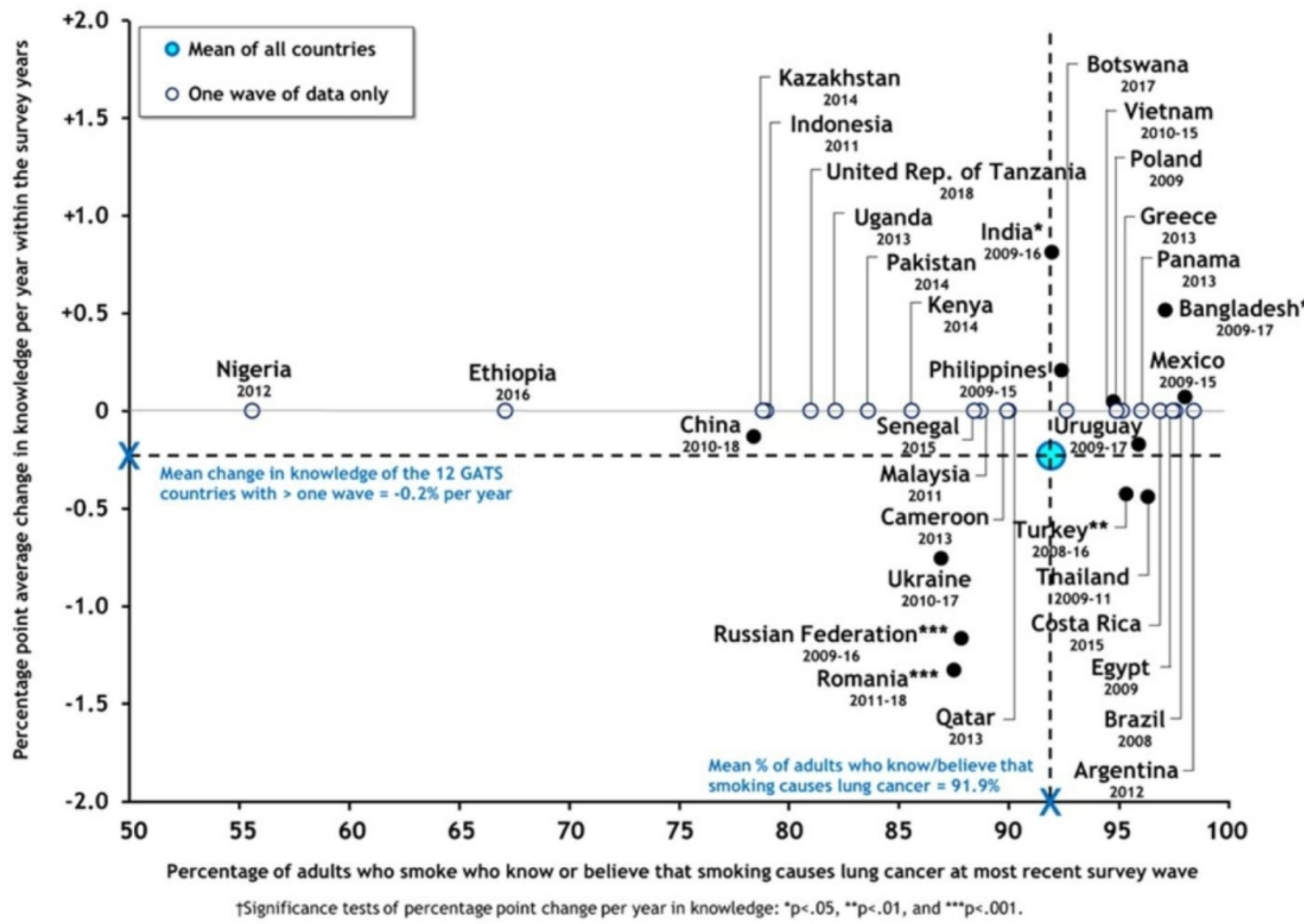

Figure 2. Percentage of adults who smoke who know or believe that smoking causes lung cancer, in 32 GATS countries,† 2008–2018.

123x94mm (300 x 300 DPI)

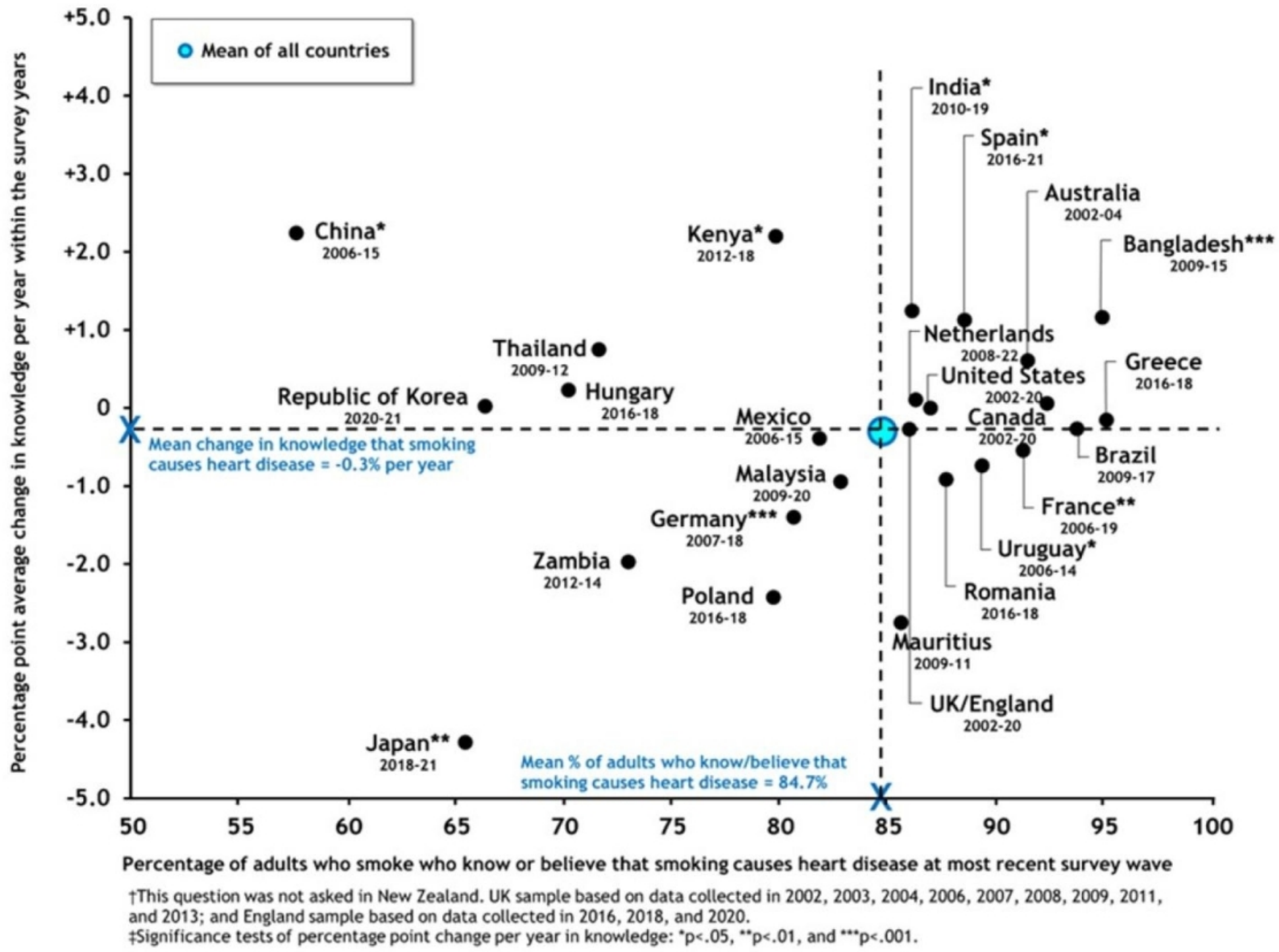

Figure 3. Percentage of adults who smoke who know or believe that smoking causes heart disease,† in 25 ITC countries,‡ 2002–2022.

123x99mm (300 x 300 DPI)

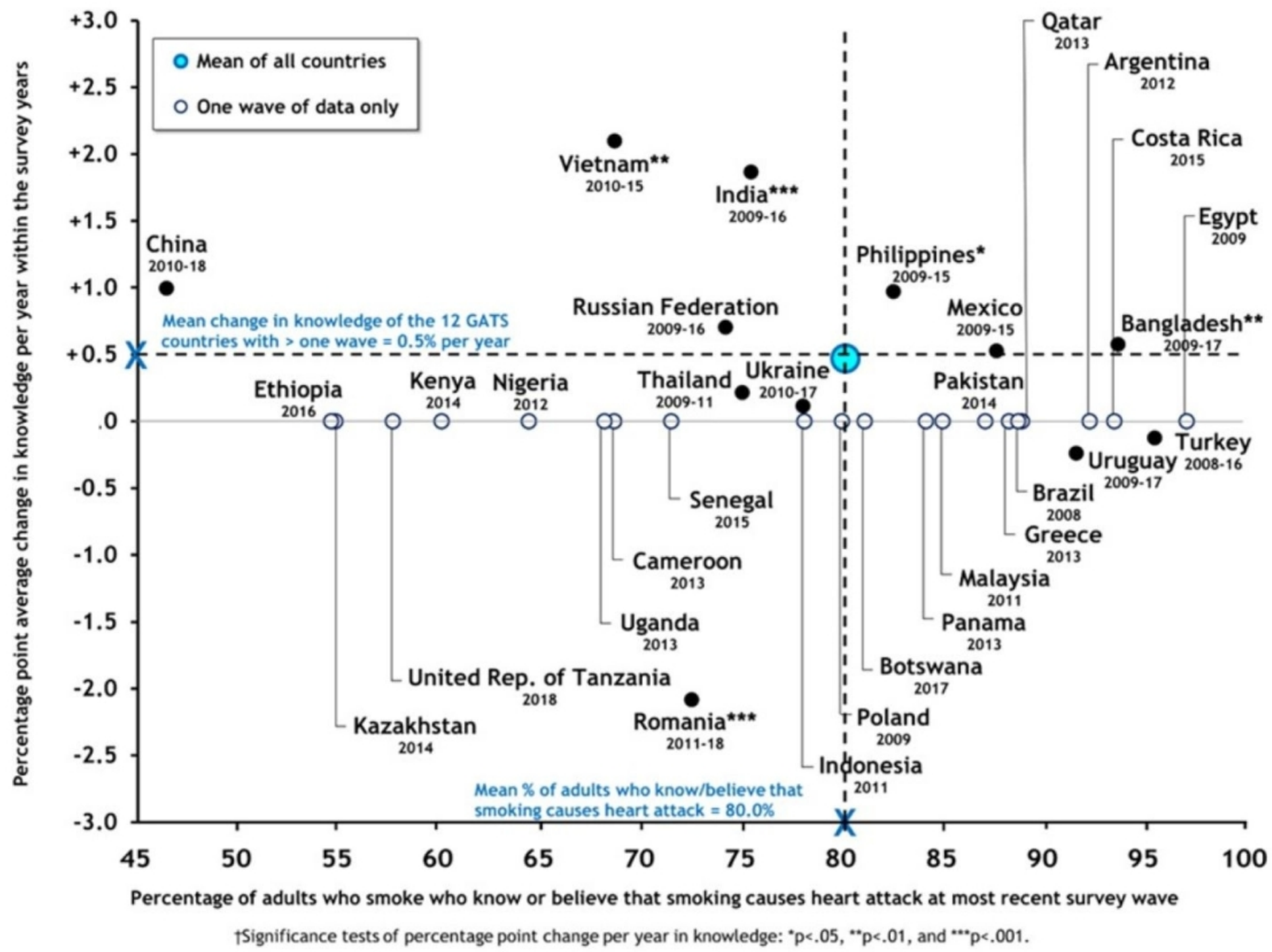

Figure 4. Percentage of adults who smoke who know or believe that smoking causes heart attack, in 32 GATS countries,† 2008–2018.

123x99mm (300 x 300 DPI)

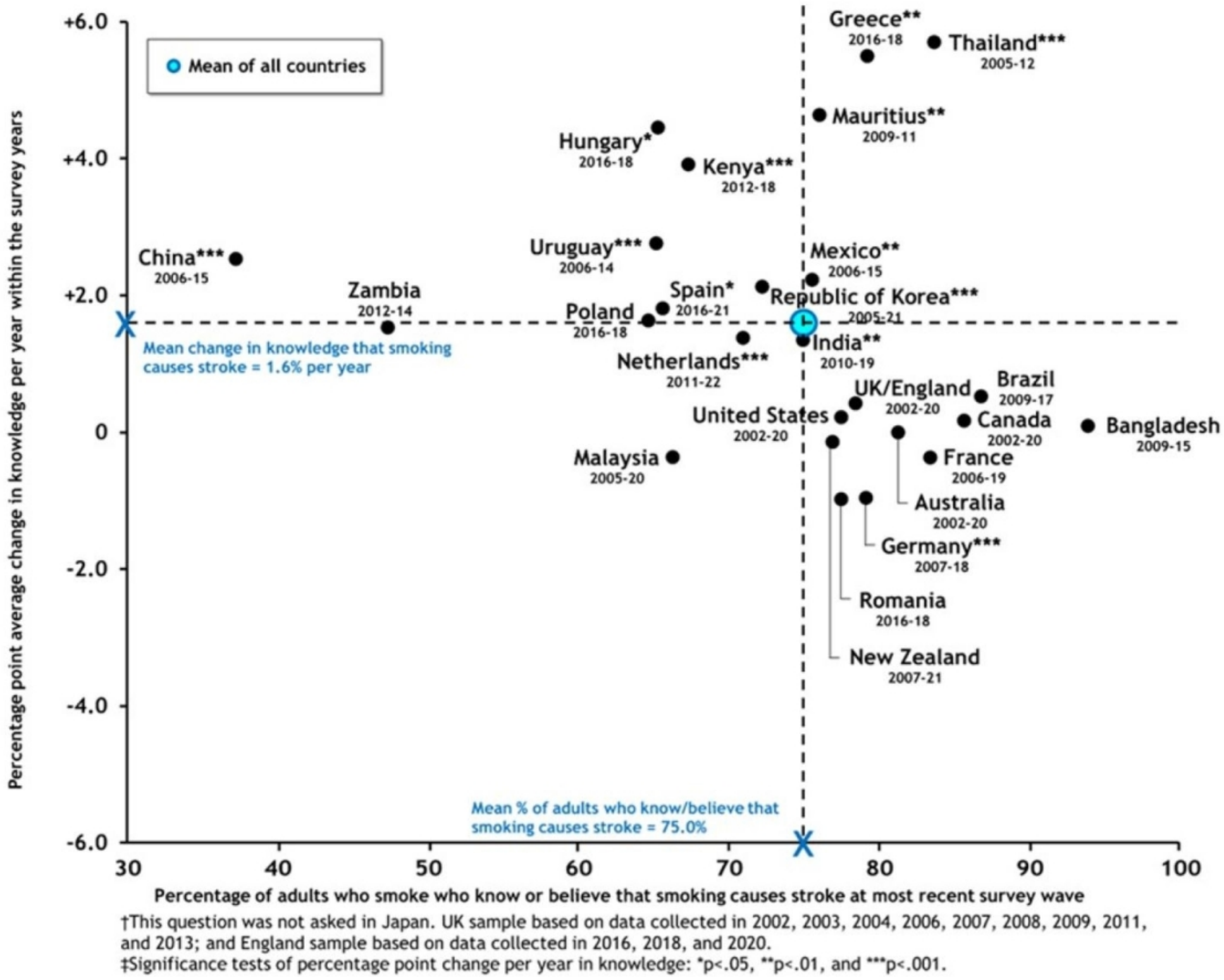

Figure 5. Percentage of adults who smoke who know or believe that smoking causes stroke,† in 25 ITC countries,‡ 2002–2022.

123x106mm (300 x 300 DPI)

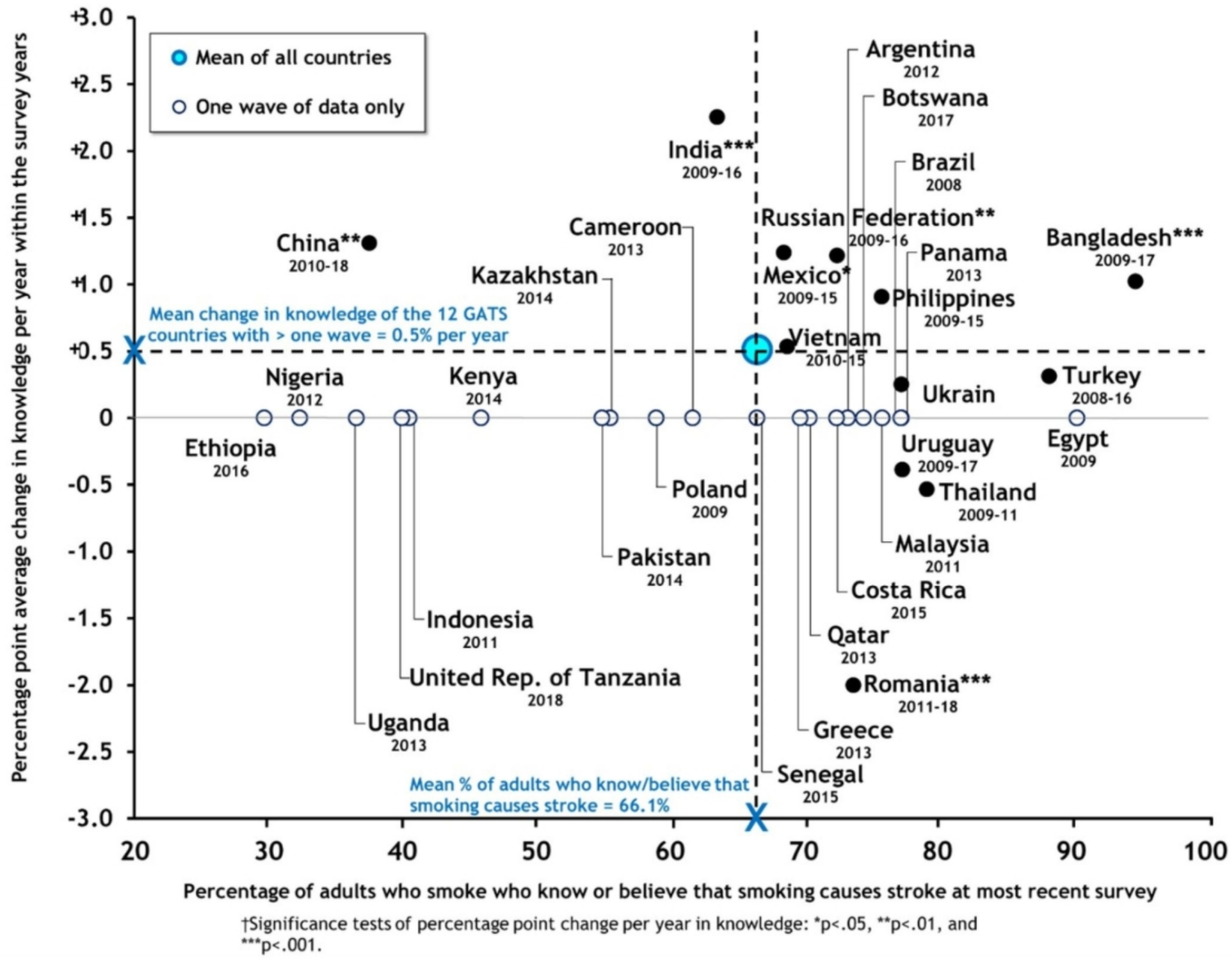

Figure 6. Percentage of adults who smoke who know or believe that smoking causes stroke, in 32 GATS countries,† 2008–2018.

181x152mm (300 x 300 DPI)

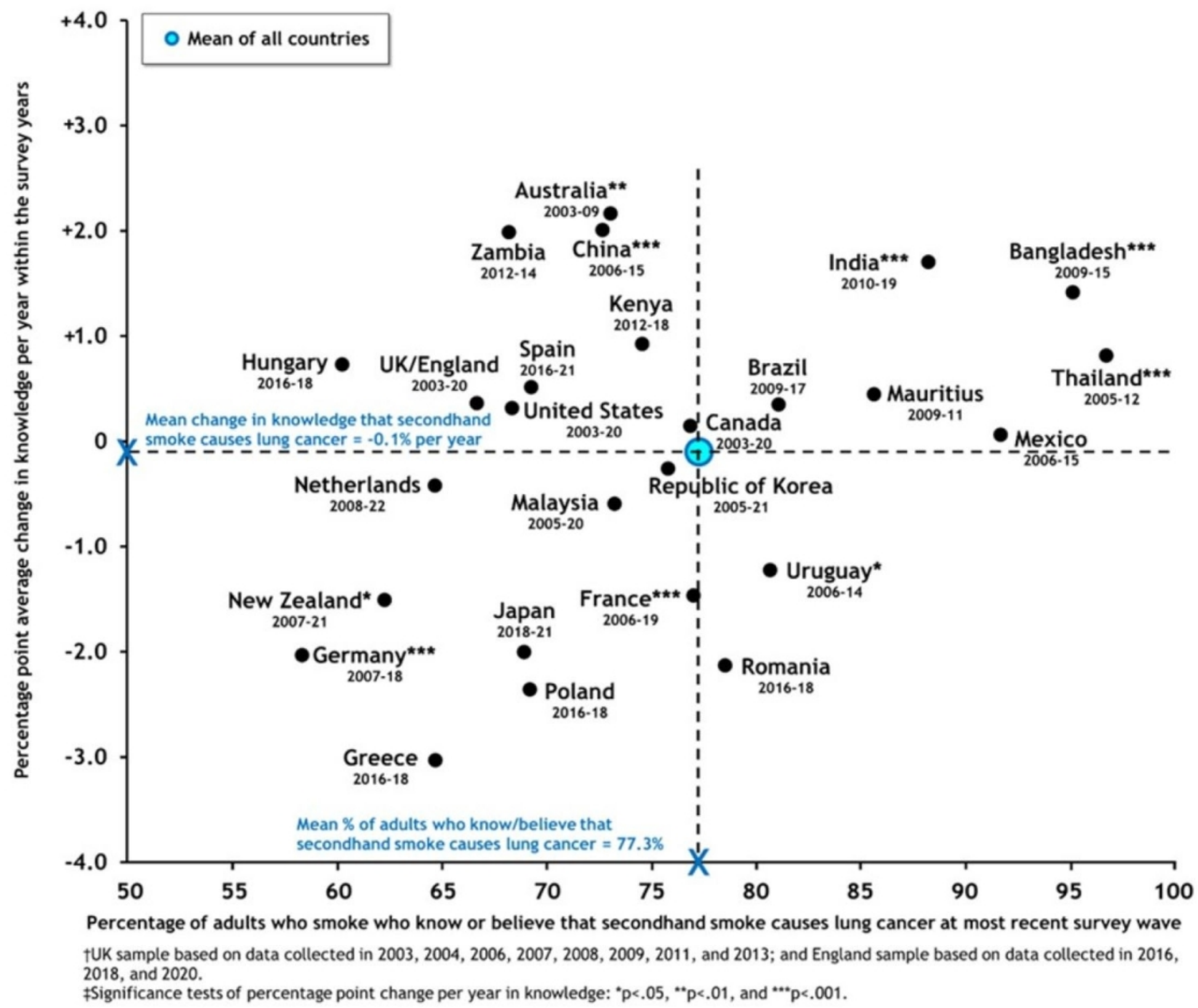

Figure 7. Percentage of adults who smoke who know or believe that secondhand smoke causes lung cancer, in 26 ITC countries,† 2003–2022.

123x112mm (300 x 300 DPI)

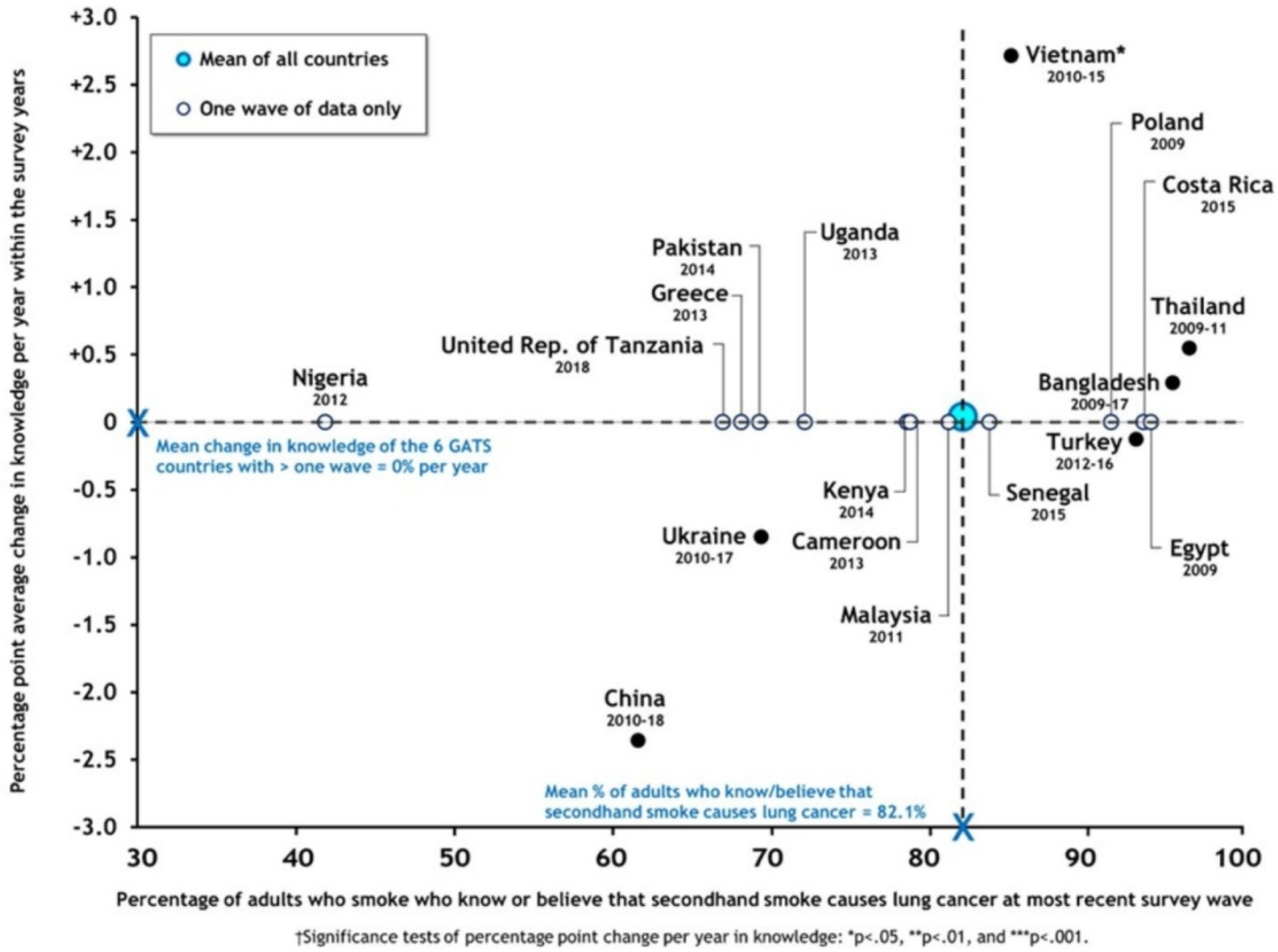

Figure 8. Percentage of adults who smoke who know or believe that secondhand smoke causes lung cancer, in 18 GATS countries,† 2009–2018.

123x93mm (300 x 300 DPI)

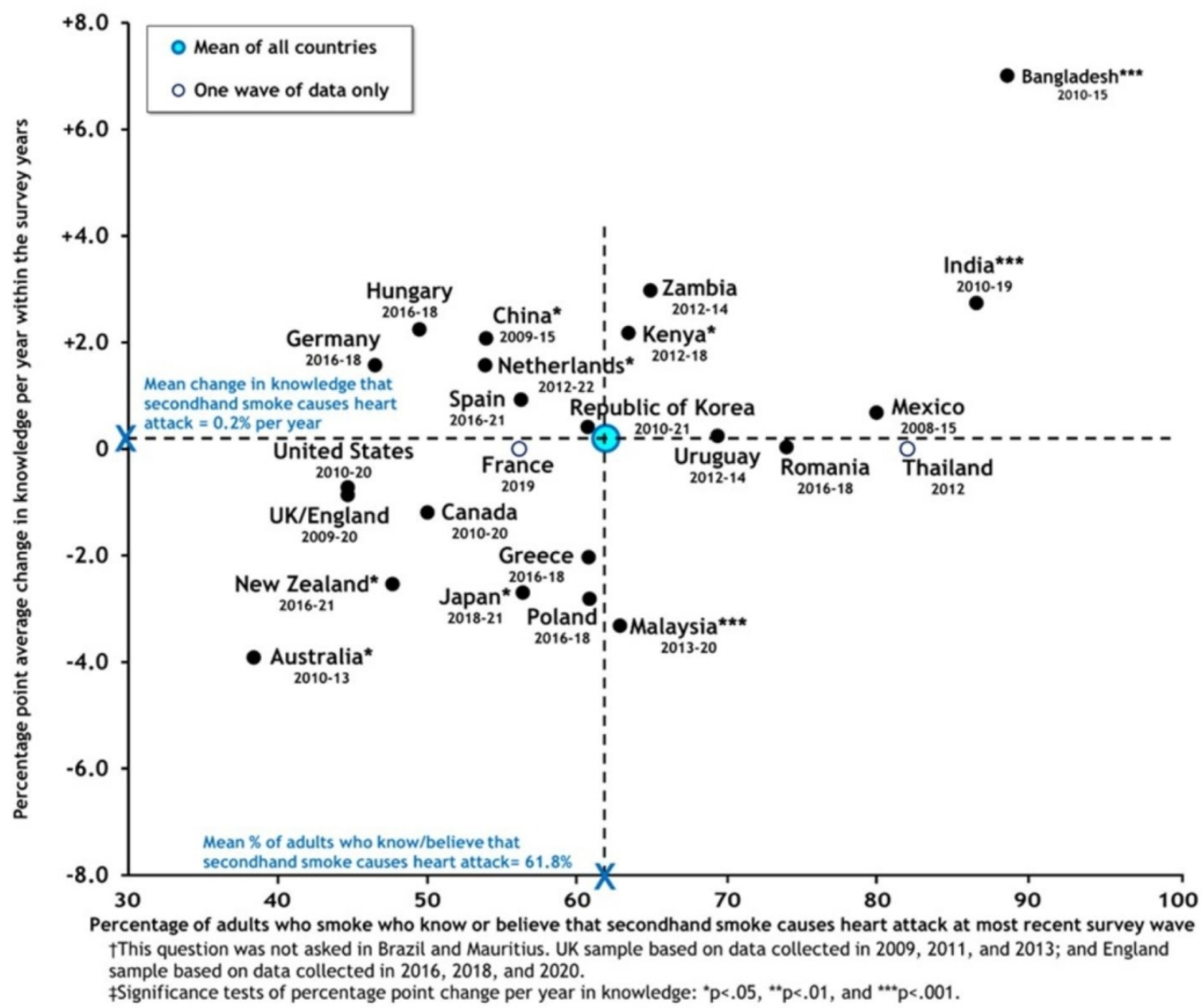

Figure 9. Percentage of adults who smoke who know or believe that secondhand smoke causes heart attack,† in 24 ITC countries,‡ 2008–2022.

123x113mm (300 x 300 DPI)

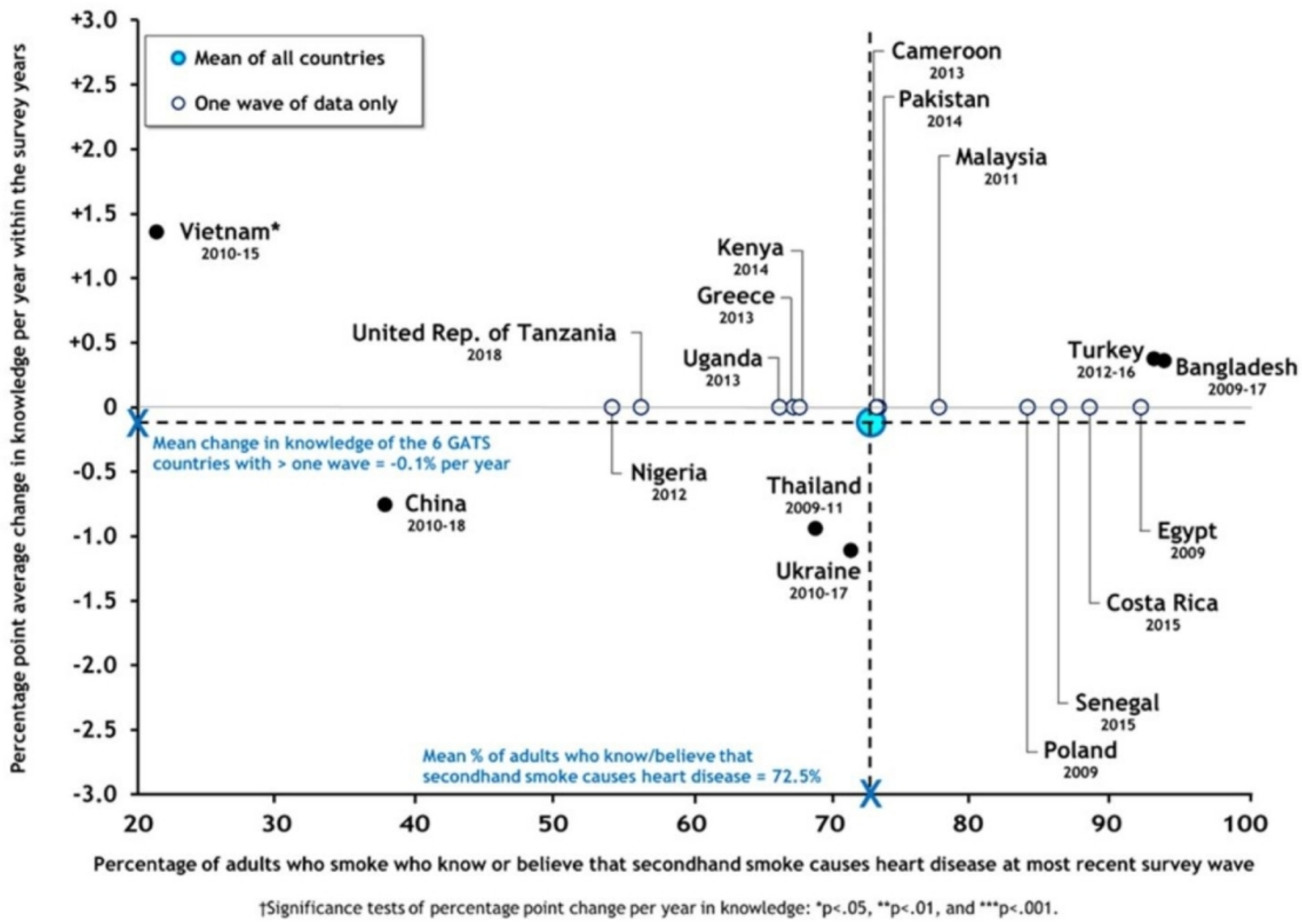

Figure 10. Percentage of adults who smoke who know or believe that secondhand smoke causes heart disease, in 18 GATS countries,† 2009–2018.

123x94mm (300 x 300 DPI)

Changes in knowledge about major harms of tobacco smoking and secondhand smoke exposure among adults who smoke: findings from 26 countries of the International Tobacco Control (ITC) Project (2002–2022) and 32 countries of the Global Adult Tobacco Survey (GATS) (2008–2020)

Journal:	*BMJ Open*
Manuscript ID	bmjopen-2025-107667.R2
Article Type:	Original research
Date Submitted by the Author:	15-Apr-2026
Complete List of Authors:	Chung-Hall, Janet; University of Waterloo, Psychology<br>Fong, Geoffrey; University of Waterloo, Psychology; University of Waterloo, School of Public Health Sciences; Ontario Institute for Cancer Research<br>Meng, Gang; University of Waterloo, Psychology<br>Craig, Lorraine; University of Waterloo, Psychology<br>Indome, Eunice; University of Waterloo, Psychology<br>Collaborators, for the ITC Project; University of Waterloo<br>Kress, Alissa; National Center for Chronic Disease Prevention and Health Promotion Office on Smoking and Health<br>Shi, Jing; National Center for Chronic Disease Prevention and Health Promotion Office on Smoking and Health; CDC Foundation Inc, Noninfectious Disease Programs<br>Ahluwalia, Indu; National Center for Chronic Disease Prevention and Health Promotion Office on Smoking and Health
**Primary Subject Heading**:	Public health
Secondary Subject Heading:	Global health, Health policy, Smoking and tobacco
Keywords:	PUBLIC HEALTH, Tobacco Use, Health

For peer review only - http://bmjopen.bmj.com/site/about/guidelines.xhtml

I, the Submitting Author has the right to grant and does grant on behalf of all authors of the Work (as defined in the below author licence), an exclusive licence and/or a non-exclusive licence for contributions from authors who are: i) UK Crown employees; ii) where BMJ has agreed a CC-BY licence shall apply, and/or iii) in accordance with the terms applicable for US Federal Government officers or employees acting as part of their official duties; on a worldwide, perpetual, irrevocable, royalty-free basis to BMJ Publishing Group Ltd ("BMJ") its licensees and where the relevant Journal is co-owned by BMJ to the co-owners of the Journal, to publish the Work in this journal and any other BMJ products and to exploit all rights, as set out in our licence.

The Submitting Author accepts and understands that any supply made under these terms is made by BMJ to the Submitting Author unless you are acting as an employee on behalf of your employer or a postgraduate student of an affiliated institution which is paying any applicable article publishing charge ("APC") for Open Access articles. Where the Submitting Author wishes to make the Work available on an Open Access basis (and intends to pay the relevant APC), the terms of reuse of such Open Access shall be governed by a Creative Commons licence – details of these licences and which Creative Commons licence will apply to this Work are set out in our licence referred to above.

Other than as permitted in any relevant BMJ Author's Self Archiving Policies, I confirm this Work has not been accepted for publication elsewhere, is not being considered for publication elsewhere and does not duplicate material already published. I confirm all authors consent to publication of this Work and authorise the granting of this licence.

For peer review only

Manuscript ID BMJOpen-2025-108667.R2

Changes in knowledge about major harms of tobacco smoking and secondhand smoke exposure among adults who smoke: findings from 26 countries of the International Tobacco Control (ITC) Project (2002–2022) and 32 countries of the Global Adult Tobacco Survey (GATS) (2008–2020)

Janet Chung–Hall,[1] Geoffrey T. Fong, [1,2,3] Gang Meng,[1] Lorraine V. Craig,[1] and Eunice O. Indome[1] for the ITC Project Collaborators*, and Alissa C. Kress,[4] Jing Shi,[4,5] and Indu B. Ahluwalia[4]

Affiliation(s):
[1] University of Waterloo, Psychology, Waterloo, ON, Canada
[2] University of Waterloo, School of Public Health Sciences, Waterloo, ON, Canada
[3] Ontario Institute for Cancer Research, Toronto, ON, Canada
[4] National Center for Chronic Disease Prevention and Health Promotion Office on Smoking and Health, Atlanta, GA, US
[5] CDC Foundation Inc., Noninfectious Disease Programs, Atlanta, GA, US
* Individual author names, email addresses, and affiliation(s) for ITC Project Collaborators are listed in Supplementary file 1A

Corresponding author:
Dr. Janet Chung-Hall
University of Waterloo, Psychology
200 University Avenue West
Waterloo, ON, Canada, N2L 3G1
Email: jchunghall@uwaterloo.ca

Word count: 5138

Disclaimer: The findings and conclusions in this article are those of the authors and do not necessarily represent the official positions of the U.S. Centers for Disease Control and Prevention or the CDC Foundation.

Funding Statement: The **ITC Four Country Surveys** (Australia, Canada, the United Kingdom, the United States) were supported by grants from the US National Cancer Institute (R01 CA100362, R01 CA090955, P50 CA111236 (Roswell Park Transdisciplinary Tobacco Use Research Center), and P01 CA138389), Robert Wood Johnson Foundation (045734), the Canadian Institutes of Health Research (FDN-148477, MOP-57897, MOP-79551, MOP-115016), the Canadian Tobacco Control Research

For peer review only - http://bmjopen.bmj.com/site/about/guidelines.xhtml

Initiative (014578), Australian Commonwealth Department of Health and Ageing, the National Health and Medical Research Council of Australia (265903, 450110, 1005922), and Cancer Research UK (C312/A3726, C312/A6465, C321/A11039, C25586/A19540). The **ITC Four Country Smoking and Vaping Surveys** (Australia, Canada, England, and the United States) were supported by grants from the US National Cancer Institute (P01 CA200512), the Canadian Institutes of Health Research (FDN-148477), and the National Health and Medical Research Council of Australia (APP1106451). The **ITC Bangladesh Surveys** were supported by grants from the US National Cancer Institute (P01 CA138389), the International Development Research Centre (104831-003), and Canadian Institutes for Health Research (MOP-79551, MOP-115016). The **ITC Brazil Surveys** were supported by the Instituto Nacional de Câncer (INCA, National Cancer Institute), Secretaria Nacional de Pokiticas sobre Drogas (SENAD, National Secretariat for Drug Policy), Pan-American Health Organization (PAHO), Brazil Fundação do Câncer (BR/LOA/1500048·00), and the Canadian Institutes of Health Research (MOP-115016, FDN-148477). The **ITC China Surveys** were supported by grants from the US National Cancer Institute at the National Institutes of Health (R01 CA125116), P50 CA111236 (Roswell Park Transdisciplinary Tobacco Use Research Center), Canadian Institutes for Health Research (MOP-79551, MOP-115016), and the Chinese Center for Disease Control and Prevention. The **ITC France Surveys** (first cohort) were supported by grants from the Observatoire français des drogues et toxicomanies (OFDT), Institut national de prevention et d'education pour la sante (INPES), and the Institut nationale du cancer (INCa). The ITC France Surveys (second cohort) were supported by grants from Santé publique France, the Institut nationale du cancer (INCa), and the Canadian Institutes of Health Research (FDN-148477). The **ITC Germany Surveys** (first cohort) were supported by grants from the German Federal Ministry of Health, Dieter Mennekes-Umweltstiftung, and Germany Cancer Research Center (DKFZ). The EUREST-PLUS Project (ITC 6 European Country Surveys) in Germany (second cohort), Greece, Hungary, Poland, Romania, and Spain has received funding from the European Union's Horizon2020 research and innovation programme 681109, and additional support was provided to the University of Waterloo by the Canadian Institutes of Health Research (FDN-148477). EF is partly supported by Ministry of Universities and Research, Government of Catalonia (2017SGR319) and by the Instituto de Salud Carlos III and co-funded by the European Regional Development Fund (INT16/00211 and INT17/00103), Government of Spain, and CERCA Programme Generalitat de Catalunya for the institutional support to IDIBELL. The EUREST-PLUS Spain Survey was supported by grants from the Instituto de Salud Carlos III (PI17/01338, Co-funded by European Regional Development Fund and the Canadian Institutes for Health Research (FDN-148477). EF and MF are partly supported by the Ministry of Universities and Research, Government of Catalonia (2021SGR00906) ,and CERCA Programme Generalitat de Catalunya for the institutional support to IDIBELL. The **ITC India Surveys** were supported by grants from the US National Cancer Institute (P50 CA111236, P01 CA138389), the Canadian Institutes of Health Research (MOP-79551, MOP-115016, FDN-148477), and Healis Sekhsaria Institute for Public Health. The **ITC Japan Surveys** were supported by the Japan National Cancer Center and Research Development Fund (28-A-24) and the Canadian Institutes of Health Research (FDN-148477). The **ITC Kenya Surveys** were supported by grants from the Canadian Institutes of Health Research (MOP-115016,

For peer review only - http://bmjopen.bmj.com/site/about/guidelines.xhtml

FDN-148477). The **ITC Thailand Surveys** were supported by grants from the US National Cancer Institute (P50 CA111236, P01 CA138389 (Roswell Park Transdisciplinary Tobacco Use Research Center), the Canadian Institutes of Health Research (MOP-79551, MOP-115016), and the ThaiHealth Promotion Foundation. The ITC Malaysia Surveys (first cohort) were supported by grants from the US National Cancer Institute (P50 CA111236 (Roswell Park Transdisciplinary Tobacco Use Research Center) and P01 CA138389), the Canadian Institutes of Health Research (MOP-79551, MOP-115016), and the Malaysian Ministry of Health. The **ITC Malaysia Surveys** (second cohort) were supported by the Malaysia Ministry of Higher Education-LRGS NanoMITe (RU029-2014), the University of Malaya Research University Grant (RU029C-2014 and RU001A-2021), and the Canadian Institutes of Health Research (FDN-148477). The **ITC Mauritius Surveys** were supported by grants from the International Development Research Centre, the Canadian Institutes of Health Research (MOP-115016), and was partially supported by the World Lung Foundation. The **ITC Mexico Surveys** were supported by grants from the Mexican Consejo Nacional de Ciencia y Tecnologia (Salud-2007-C01-70032, Salud-2013-01-202671), Bloomberg Global Initiative—International Union Against Tuberculosis and Lung Disease, the US National Cancer Institute (P50 CA111236 (Roswell Park Transdisciplinary Tobacco Use Research Center) and the Canadian Institutes for Health Research (MOP-115016). The **ITC Netherlands Surveys** (first cohort) were supported by grants from The Netherlands Organisation for Health Research and Development (ZonMw 121010008, ZonMw 200130002), The SILNE Project (funded by the European Commission through FP7 HEALTH-F3-2011-278273) and the Dutch Cancer Foundation (KWF) (UM 2014-7210). The ITC Netherlands Surveys (second cohort) were supported by grants from Longfonds (Lung Foundation Netherlands), Hartstichting (Netherlands Heart Foundation), KWF Kankerbestrijding (Dutch Cancer Society), Trombosestichting Nederland (Thrombosis Foundation), Diabetesfonds (Diabetes Fund) and the Canadian Institutes of Health Research (FDN-148477). The **ITC New Zealand Surveys** (first cohort) were supported by grants from the New Zealand Health Research Council (06/453), the New Zealand Ministry of Health, University of Otago, the US National Cancer Institute (P50 CA111236 (Roswell Park Transdisciplinary Tobacco Use Research Centre)), and the Canadian Institutes of Health Research (MOP-79551). The ITC New Zealand (EASE: Evidence for Achieving Smokefree 2025 Equitably) Surveys (second cohort) were supported by grants from the New Zealand Health Research Council (GA215F 15/072, HRC 19/641), the New Zealand Ministry of Health, and the Canadian Institutes for Health Research (FDN-148477). The **ITC Korea Surveys** (first cohort) were supported by grants from the National Cancer Center of Korea (0731040-1), the Korean Ministry of Health and Welfare, and the US National Cancer Institute (P50 CA111236, R01 CA125116) and the Canadian Institutes of Health Research (MOP-79551). The ITC Korea Survey (second cohort) was supported by a grant from the Korean Ministry of Health and Welfare and the Canadian Institutes of Health Research (MOP-115016). The ITC Korea Surveys (third cohort) were supported by a grant from the Korean Ministry of Health and Welfare and the Canadian Institutes of Health Research (FDN-148477). The **ITC Uruguay Surveys** were supported by grants from the US National Cancer Institute (P50 CA111236 (Roswell Park Transdisciplinary Tobacco Use Research Center), P01 CA138389), and the Canadian Institutes of Health Research (MOP-115016). The ITC Zambia Surveys were supported by a grant from the

For peer review only - http://bmjopen.bmj.com/site/about/guidelines.xhtml

Canadian Institutes of Health Research (MOP-115016). Additional support provided by the Ontario Institute for Cancer Research (Senior Investigator Grant to GTF (2002-present)), and the Canadian Cancer Society Research Institute (Senior Prevention Scientist Award to GTF (2002-09), and O. Harold Warwick Prize to GTF (2021) (Supplementary file 1A). Financial support for **GATS** was provided by the CDC Foundation with grants from Bloomberg Philanthropies and the Bill and Melinda Gates Foundation, and/or in-country donor funding for GATS countries listed in Supplementary file 1B, Supplemental Table 1. The funders had no role in the design of the study; in the collection, analyses, or interpretation of data; in the writing of the manuscript, or in the decision to publish the results.

Ethics statement: The **ITC Four Country** (Australia, Canada, United Kingdom, and United States) survey protocols and all materials, including the survey questionnaires, were cleared for ethics by Research Ethics Board, University of Waterloo, Canada (REB#10556, REB#13978, REB#17469), Human Research Ethics at Cancer Council Victoria, Australia (IRB HREC 0211), Office of Research Subject Protection, Roswell Comprehensive Cancer Center, US (IRB NT 02-20), and Research Ethics Office, King's College London, UK (IRB PNM/13/14-151). The **ITC Four Country Smoking and Vaping** (Australia, Canada, England, and United States) survey protocols and all materials, including the survey questionnaires, were cleared for ethics by Research Ethics Board, University of Waterloo, Canada (REB#20803/30570, REB#20803/30878), Human Research Ethics at Cancer Council Victoria, Australia (IRB HREC 1603), Human Research Ethics Committee, The University of Queensland, Australia (IRB #2016000330), Research Ethics Office, King's College London, UK (IRB RESCM-17/18-2240), and ethics clearance at the Medical University of South Carolina, US was waived due to minimal risk. The **ITC Bangladesh** survey protocols and all materials, including the survey questionnaires, were cleared for ethics by Research Ethics Board, University of Waterloo, Canada (REB#15019, REB#19862/30312), and Bangladesh Medical Research Council (IRB BMRC/ERC/2013-2016/1729, IRB BMRC/ERC/2007-2010/1372). The **ITC Brazil** survey protocols and all materials, including the survey questionnaires, were cleared for ethics by Research Ethics Board, University of Waterloo, Canada (REB#15076, REB#21456/30826), and National Cancer Institute of Brazil (INCA)—IRB 99/08). The **ITC China** survey protocols and all materials, including the survey questionnaires, were cleared for ethics by Research Ethics Board, University of Waterloo, Canada (REB#12539, REB#15305, REB#17014/30105), Cancer Council Victoria, Australia (IRB IER0803), and Chinese Center for Disease Control and Prevention (IRB 201114, IRB 201325). The **ITC France** (first cohort) survey protocols and all materials, including the survey questionnaires, were cleared for ethics by Research Ethics Board, University of Waterloo, Canada (REB#13005, 183/30190). The **ITC France** (second cohort) survey protocols and all materials, including the survey questionnaires, were cleared for ethics by Research Ethics Board, University of Waterloo, Canada (REB#40720). The **ITC Germany** (first cohort) survey protocols and all materials, including the survey questionnaires, were cleared for ethics by Research Ethics Board, University of Waterloo, Canada (REB#13858), and Heidelberg Medical School, Germany (IRB S-138/2007). The **EUREST-PLUS** (**ITC 6 European Country** in Germany (second cohort), Greece, Hungary, Poland, Romania, and Spain) survey protocols and all

For peer review only - http://bmjopen.bmj.com/site/about/guidelines.xhtml

materials, including the survey questionnaires, were cleared for ethics by Research Ethics Board, University of Waterloo, Canada (REB#21262/30709), Comisia de Etica Iuliu Hatieganu University of Medicine and Pharmacy, Romania (IRB 155), Research Ethics Boards of the Bellvitge University Hospital, (IRB PR100/16), Committee and Dean of the Department of Health Care and Life Sciences, State College of Higher Vocational Education, Poland (IRB 1/2016), Research and Ethics Committee, Medical School, University of Athens, Greece (IRB 1516023880), Scientific and Research Committee, Medical Research Council, Hungary (IRB 11012-2-2016/EKU 0198/16, IRB 46344-2/2017/EKU), and Heidelberg Medical School, Germany (IRB 196/2016). The **ITC EUREST-PLUS Spain** (third wave) survey protocols and all materials, including the survey questionnaires, were cleared for ethics by Research Ethics Board, University of Waterloo, Canada (REB#41105), and Research Ethics Boards of the Bellvitge University Hospital, (IRB PR100/16, IRB PR248/17). The **ITC India** survey protocols and all materials, including the survey questionnaires, were cleared for ethics by Research Ethics Board, University of Waterloo, Canada (REB#132,97, REB#15722, REB#22140/31086), and Healis Sekhsaria Institute for Public Health (IRB 00007340). The **ITC Japan** survey protocols and all materials, including the survey questionnaires, were cleared for ethics by Research Ethics Board, University of Waterloo, Canada (REB#22508/31428), National Cancer Center, Japan (IRB 2021-069), and Osaka International Cancer Institute (IRB 21054). The **ITC Kenya** survey protocols and all materials, including the survey questionnaires, were cleared for ethics by Research Ethics Board, University of Waterloo, Canada (REB#18311/30136), and University of Nairobi (IRB KNH-ERC/A/281). The **ITC Thailand** survey protocols and all materials, including the survey questionnaires, were cleared for ethics by Research Ethics Board, University of Waterloo, Canada (REB#11762, REB#15468), Roswell Park Comprehensive Cancer Center, US (NT-02-20), Cancer Council Victoria, Australia (HREC 0420), and Mahidol University, Thailand (IRB 0517·191/1003). The **ITC Malaysia** (first cohort) survey protocols and all materials, including the survey questionnaires, were cleared for ethics by Research Ethics Board, University of Waterloo, Canada (REB#11762, REB#15468), Roswell Park Comprehensive Cancer Center, US (NT-02-20), Cancer Council Victoria, Australia (HREC 0420), and Universiti Sains Malaysia, Malaysia (USM/PPSP/Ethics Com./2004(137·4[3])). The **ITC Malaysia** (second cohort) survey protocols and all materials, including the survey questionnaires, were cleared for ethics by Research Ethics Board, University of Waterloo, Canada (REB#40825) Medical Research Ethics Committee, University Malaya, Malaysia (MREC ID#2019118-8018). The **ITC Mauritius** survey protocols and all materials, including the survey questionnaires, were cleared for ethics by Research Ethics Board, University of Waterloo, Canada (REB#15487), and Mauritius Institute of Health, Mauritius (IRB MHC/CT/NETH/MIH). The **ITC Mexico** survey protocols and all materials, including the survey questionnaires, were cleared for ethics by Research Ethics Board, University of Waterloo, Canada (REB#13195, REB#13472, REB#16353), and Instituto Nacional de Salud Publica, Mexico (IRB 759). The **ITC Netherlands** (first cohort) survey protocols and all materials, including the survey questionnaires, were cleared for ethics by Research Ethics Board, University of Waterloo, Canada (REB#14666, REB#18920/30242). The **ITC Netherlands** (second cohort) survey protocols and all materials, including the survey questionnaires, were cleared for ethics by Research Ethics Board, University of Waterloo, Canada (REB#41704). The **ITC New**

For peer review only - http://bmjopen.bmj.com/site/about/guidelines.xhtml

Zealand (first cohort) survey protocols and all materials, including the survey questionnaires, were cleared for ethics by Research Ethics Board, University of Waterloo, Canada (REB#13547), and Multi-Region Ethics Committee, New Zealand (MEC/06/07/071). The **ITC New Zealand (EASE: Evidence for Achieving Smokefree 2025 Equitably)** (second cohort) survey protocols and all materials, including the survey questionnaires, were cleared for ethics by Research Ethics Board, University of Waterloo, Canada (REB#21211/30726, REB#42549) and Human Ethics Committee, University of Otago (IRB 15/126, IRB 20/020). The **ITC Korea** (first cohort) survey protocols and all materials, including the survey questionnaires, were cleared for ethics by Research Ethics Board, University of Waterloo, Canada (REB#12547, REB#15017), and Internal Review Board, National Cancer Center, Republic of Korea (IRB NCCNCS-05-050, IRB NCCNCS-08-172, IRB NCCNCS-10-373), The **ITC Korea** (second cohort) survey protocols and all materials, including the survey questionnaires, were cleared for ethics by Research Ethics Board, University of Waterloo, Canada (REB#21287/30768) and Internal Review Board, National Cancer Center, Republic of Korea (IRB NCCNCS—2016-0129). The **ITC Korea** (third cohort) survey protocols and all materials, including the survey questionnaires, were cleared for ethics by Research Ethics Board, University of Waterloo, Canada (REB#41512) and Korea Health Promotion Institute (IRB KHPI 120160811107AN01-2004-HR-042-02). The **ITC Uruguay** survey protocols and all materials, including the survey questionnaires, were cleared for ethics by Research Ethics Board, University of Waterloo, Canada (REB#13195, REB#13472, REB#16665/30090) and Universidad de la República, Uruguay (IRB 071140-000649-08). The **ITC Zambia** survey protocols and all materials, including the survey questionnaires, were cleared for ethics by Research Ethics Board, University of Waterloo, Canada (REB#18086/303137), University of Zambia, Zambia (IRB 021-05-12). All secondary data analysis using ITC country data were cleared for ethics by Research Ethics Board, University of Waterloo, Canada (REB#31040). All participants provided consent for participating in the ITC surveys (Supplementary file 1A).

Competing Interests: KMC has in the past and continues to serve as a paid witness in litigation filed against cigarette manufacturers. GTF has been an expert witness or consultant for governments defending their country's policies or regulations in litigation. CG has been a paid expert witness for the Australian Government in litigation concerning nicotine product marketing. KK received a JMWH Bayer Grant from Japan Society for Menopause and Women's Health. ASAN has received an unconditional educational grant from Johnson & Johnson Malaysia Sdn. Bhd. JFT has served as paid expert witness in legal challenges against tobacco and vaping companies. All other authors have no conflicts of interest to declare (Supplementary file 1A).

Patient and Public Involvement: Patients or the public were not involved in the design, or conduct, or reporting, or dissemination plans of our research.

For peer review only - http://bmjopen.bmj.com/site/about/guidelines.xhtml

ABSTRACT

Objectives. To assess levels of and trends in knowledge that smoking and secondhand smoke (SHS) causes lung cancer and cardiovascular disease (CVD) in 46 countries over two decades.

Design. Longitudinal cohort and repeat cross-sectional surveys.

Setting. Nationally representative surveys in 26 International Tobacco Control (ITC) countries (2002-2022), and 32 Global Adult Tobacco Survey (GATS) countries (2008-2020).

Participants. Adults (18+ years) who smoke tobacco (26 ITC countries: N=133,410; 32 GATS countries: N=110,537).

Analysis outcomes. Current use of cigarettes at least monthly (all ITC countries) and/or current use of bidis at least monthly (ITC India, Bangladesh, and Zambia). Current use of any combustible tobacco products (all GATS countries). We conducted individual-level analyses of the ITC and GATS data separately, without combining datasets or analyzing previously published summary statistics. Generalized estimating equations and weighted multinomial regression models assessed country-level prevalence of knowledge for three smoking and two SHS outcomes. Multilevel models estimated all country mean prevalence and tested within country linear trends over time for five disease indicators by survey. Across 12 countries, with data for both surveys, correlations between ITC and GATS assessed consistency across indicators.

Results. Knowledge that smoking causes lung cancer was highest (ITC=92·2%, GATS=91·9%), followed by heart disease (ITC=84·7%)/heart attack (GATS=80·0%), and stroke (ITC=75·0%, GATS=66·1%). Knowledge that SHS causes lung cancer

For peer review only - http://bmjopen.bmj.com/site/about/guidelines.xhtml

(ITC=77·3%, GATS=82·1%) was highest, followed by heart attack (ITC=61·8%)/heart disease (GATS=72·5%) (all p<·0001). In ITC and GATS countries, average change in knowledge across five diseases was +0·14% per year. There was agreement between ITC and GATS data (range rs= ·60–·81 across five indicators).

Conclusions. There is a need to improve and sustain people's knowledge about diseases caused by smoking and SHS, especially CVD risks.

For peer review only

For peer review only - http://bmjopen.bmj.com/site/about/guidelines.xhtml

STRENGTHS AND LIMITATIONS OF THIS STUDY

- This study analyzed nationally representative surveys from 46 countries ─ the International Tobacco Control (ITC) Project and the Global Adult Tobacco Survey (GATS) ─ to assess levels of and trends in knowledge about lung cancer and cardiovascular diseases over two decades.
- Use of self-reported data may be subject to recall bias.
- Generalizability of findings is limited by low response rates for some ITC surveys and the restriction of ITC-GATS correlation analyses to 12 countries.

For peer review only

For peer review only - http://bmjopen.bmj.com/site/about/guidelines.xhtml

INTRODUCTION

Over 8 million people die from tobacco-caused diseases per year; tobacco smoking is the single most preventable cause of death globally.[1] The WHO Framework Convention on Tobacco Control (FCTC), entered into force in 2005, calls upon Parties (183 to date) to implement evidence-based measures to reduce tobacco use.[2] The WHO MPOWER[a] package includes implementation of six evidence-based measures at country-level.[3] The W strategies include the display of pictorial health warnings on 75% of the surface of cigarette packs [4,5] and mass media campaigns to increase knowledge about tobacco-caused health risks.[6,7]

Knowledge about the harms of smoking is positively associated with quit intentions,[8] attempts,[9] and cessation success.[10] People who smoke and are unaware of the health risks of active smoking may be less likely to take steps to protect themselves from firsthand smoke exposure. Similarly, low awareness of the harms of secondhand smoke (SHS) exposure may reduce efforts to protect others around them from SHS .[7] Despite efforts to inform people about the harms of smoking and SHS, gaps in knowledge remain. Studies have found that adults in the general population,[11] and those who use cigarettes[12–15] and other smoked tobacco products,[15] report higher knowledge that smoking causes lung cancer compared with heart disease and/or stroke.[b] They are also are more likely to know that SHS exposure causes lung cancer

[a] The six MPOWER measures are Monitor tobacco use and policies; Protect people from tobacco smoke; Offer to help quit tobacco use; Warn about dangers of tobacco; Enforce bans on tobacco advertising, promotion, and sponsorship; and Raise tobacco taxes.

[b] The 2023 World Health Organization International Classification of Diseases 11th Revision (ICD–11) defines heart disease and heart attack as circulatory system diseases, and stroke as a cerebrovascular disease. In this paper, the term CVD will be used to refer to cardiovascular (coronary heart disease, myocardial infarction) and/or cerebrovascular disease (stroke) to allow for more readable text.

For peer review only - http://bmjopen.bmj.com/site/about/guidelines.xhtml

as compared with heart disease. [14,15]

Limited studies have examined health risk perceptions of SHS. Single-country cross-sectional studies have found variation in knowledge of diseases caused by SHS across countries. Few studies have examined the risk perceptions of SHS. For example, studies from Canada [16] and the United Kingdom (UK) [17] have found high awareness that SHS is a risk factor for cancer among adults in the general population. In China, adults in the general population reported higher knowledge that SHS causes lung cancer compared with heart disease and respiratory disease.[18] Similarly, adult males who smoke in Malaysia had limited knowledge for the risks of heart disease and respiratory diseases from SHS.[19] In contrast, adults who do not smoke in Nigeria had high awareness that SHS causes cancer, cardiovascular disease, and respiratory disease.[20] These country differences may reflect contextual determinants of SHS exposure ─ such as the adoption of smoke-free homes, implementation and enforcement of smoking bans in public places, and SHS mass media campaigns [21–24] ─ which may in turn, influence knowledge of SHS-caused diseases.

Few studies have examined changes in knowledge about smoking- and SHS-caused diseases. A repeated cross-sectional study found significant increases from 1999 to 2006 in risk perceptions of SHS for lung cancer and heart disease among adults of the Irish general adult population.[25] One Global Adult Tobacco Survey (GATS) study in six countries between 2008 and 2016, found significant increases in knowledge that smoking causes lung cancer, heart attack, and stroke among persons aged 15+ years who smoke.[15]

For peer review only - http://bmjopen.bmj.com/site/about/guidelines.xhtml

This study assessed knowledge and changes in knowledge over time about key chronic diseases caused by smoking tobacco and SHS exposure among adults who smoke across 46 countries. Indicators included knowledge that smoking causes lung cancer, heart disease/heart attack, and stroke in people who smoke, and that SHS causes lung cancer and heart disease/heart attack in people who do not smoke.

METHODS

Study design and participants

Data were from the ITC Project and the Global Adult Tobacco Survey System (GTSS) (GATS).

ITC Project longitudinal cohort surveys with replenishment are administered in 31 countries using a standardised set of questions, data collection protocol, and sampling design that allows for cross-country comparisons. For this study, we used ITC Project Survey data from nationally representative samples of adults (aged 18+ years) who smoke tobacco in 26 countries between 2002 and 2022: Australia, Bangladesh, Brazil, Canada, China, England/UK[c], France, Germany, Greece, Hungary, India, Japan, Kenya, Malaysia, Mauritius, Mexico, the Netherlands, New Zealand, Poland, Republic of Korea, Romania, Spain, Thailand, the US, Uruguay, and Zambia (Supplementary table 1). Adults who smoke tobacco were defined as persons aged 18+ years who smoke tobacco, including those who currently smoke manufactured and/or roll-your own cigarettes at least monthly and had smoked at least 100 cigarettes in their lifetime (all

[c] Caution is warranted in the comparison of ITC Survey data from the United Kingdom (which includes England, Wales, Scotland, and Northern Ireland;2002 to 2010–11) and England (2020). However, England accounts for ~85% of the population of the United Kingdom, and smoking prevalence and tobacco control policies are similar across all four countries that comprise the United Kingdom. As such, we combined data from the United Kingdom and England for analysis, as approved by ITC Project Principal Investigators.

For peer review only - http://bmjopen.bmj.com/site/about/guidelines.xhtml

countries), and/or currently smoke bidis at least monthly (India, Bangladesh, and Zambia).

GATS is a nationally representative repeated cross-sectional household survey of persons aged 15+ years that uses a standard core questionnaire, sample design, and data collection methods. GATS data included in this study are from adults who smoke tobacco in 32 countries between 2008 and 2020: Argentina, Bangladesh, Botswana, Brazil, Cameroon, China, Costa Rica, Egypt, Ethiopia, Greece, India, Indonesia, Kazakhstan, Kenya, Malaysia, Mexico, Nigeria, Pakistan, Panama, Philippines, Poland, Qatar, Romania, Russian Federation, Senegal, Tanzania, Thailand, Turkey, Uganda, Ukraine, Uruguay, and Viet Nam (Supplementary table 2). For consistency in analysis across GATS and ITC, an age cut-off of 18+ years was used to define adults who smoke combustible tobacco products (bidis, kreteks, cheroots, manufactured cigarettes, hand-rolled cigarettes, cigars, cigarillos, pipes, waterpipe with tobacco).

Data on knowledge of smoking- and SHS-caused diseases were collected from adults who smoked tobacco and participated in at least one wave of the ITC Surveys (N=133,410) and/or the GATS (N=110,537). We used data from all survey years to test for significant change in knowledge, and data from the first and last survey waves to compute average yearly change in knowledge. Countries included in the ITC Surveys and/or GATS are listed in Supplementary table 3. Sample demographics and methodological details for ITC Surveys are described elsewhere[26] and in online individual country technical reports.[27] Methodological details for the GATS and individual country fact sheets are available online.[28]

For peer review only - http://bmjopen.bmj.com/site/about/guidelines.xhtml

This study includes data from 26 countries of the International Tobacco Control (ITC) Project (2002-2022) that are available upon reasonable request [dataset] [29], and data from 32 countries of the Global Adult Tobacco Survey (GATS) (2008-2020) that are available in a public, open access repository: https://www.gtssacademy.org/. [dataset][28] Approval for use of ITC Survey data was granted by ITC Project Principal Investigators on July 28, 2023 , and approval for use GATS data was granted by theUS Centers for Disease Control and Prevention Office on Smoking and Health approval on May 1, 2023.. Data from ITC and GATS countries that were collected and/or became publicly available after these dates were excluded from analyses. All data relevant to the study are included in the article or uploaded as supplementary information.

Measures

Knowledge about smoking-caused diseases was assessed by asking participants if they know or believe that smoking causes lung cancer, heart disease/heart attack, and stroke. Respondents were also asked if they know or believe that SHS causes lung cancer and heart disease/heart attack in people who do not smoke. Responses were coded as 'yes' vs. 'no/don't know'. Response 'refused' was excluded from the analysis. ITC Survey and GATS questions and response options are presented in Supplementary table 4. Questions for all five disease outcomes were not included at every survey wave across all 26 ITC and 32 GATS countries. We used the most recent data available for each country to calculate point estimates and all-country means of the prevalence of knowledge for each disease outcome, and all longitudinal data available for each country to assess changes in knowledge over time.

Statistical analysis

For peer review only - http://bmjopen.bmj.com/site/about/guidelines.xhtml

We conducted individual-level analyses of the ITC and GATS data separately, without combining datasets or analyzing previously published summary statistics. Point estimates of the prevalence of knowledge for each disease outcome by country were calculated using weighted multinomial survey logistic regression models for pooled repeated cross-sectional GATS data, and generalized estimating equations for pooled longitudinal ITC data. All models incorporated survey design information (strata and primary sampling units for multi-stage sampling surveys), adjusted for sex, age, smoking status (daily, non-daily), and time-in-sample (ITC countries).

Point estimates of all-country means of the prevalence of knowledge for each disease outcome across all ITC countries and all GATS countries, respectively, were calculated using a multi-level model (two levels: individual and country) with random intercepts at the country-level and with roughly equal weights for each country. These point estimates cannot be used to make inferences to the global population. As such, we report the estimated all-country means of knowledge for each health effect without the associated confidence intervals, which would refer to a hypothetical superpopulation of countries. The all-country mean estimates in this analysis therefore only serve as the central point of selected countries. GEE and survey logistic regression models were used to test statistical differences in comparing knowledge between the five health effects among respondents in ITC and GATS countries, respectively.

We estimated changes in knowledge for each outcome among adults who smoke in countries with at least two waves of data between 2002 and 2022 (ITC Project Surveys), and between 2008 and 2020 (GATS). Analysis of the longitudinal ITC data used generalized estimating equations to account for within-subject correlations given

For peer review only - http://bmjopen.bmj.com/site/about/guidelines.xhtml

that a proportion of individuals participated in more than one survey. Analysis of the repeated cross-sectional GATS data used weighted multinomial survey logistic regression models. Two-level models (individual and country) with random intercepts and random slopes of survey years at the country-level were used to test for significant yearly change in knowledge for each health effect within countries. This modelling approach controls for the number of years between the first and last survey waves, and assumed a linear survey year effect within each country.

Data from ITC and GATS countries were pooled and fitted separately to ensure comparability within ITC countries and within GATS countries. Pearson correlations (r) between ITC and GATS mean prevalence estimates of knowledge for each of the five diseases were used to examine consistency of estimates from different surveys conducted in the 12 overlapping countries.

The predicted marginal standardization method (PREDMARG) was used to generate all marginal estimates of the outcomes. Data analysis were conducted using SAS v9.4 and SAS-callable SUDAAN v11.0.3. Statistical significance ($p < .05$ considered statistically significant) and confidence intervals were computed at the 95% confidence level.

RESULTS

Knowledge about diseases caused by smoking and SHS exposure

Knowledge about diseases caused by smoking among adults who smoke was highest for lung cancer (ITC=92.2%, GATS=91.9%), followed by heart disease (ITC=84.7%)/heart attack (GATS=80.0%), and stroke (ITC=75.0%, GATS=66.1%) (Supplementary table 5). Knowledge that SHS causes lung cancer among people who

For peer review only - http://bmjopen.bmj.com/site/about/guidelines.xhtml

do not smoke (ITC=77.3%, GATS=82.1%) was higher than for SHS causes heart attack (ITC=61.8%)/heart disease (GATS=72.5%) in people who do not smoke. All differences between knowledge about the five diseases caused by smoking and SHS exposure were significant at $p < .001$.

Knowledge that active smoking causes lung cancer, heart disease/heart attack, and stroke

At the most recent survey across 25 ITC countries, 92.2% of adults who smoke overall knew that smoking causes lung cancer, ranging from 98.2% in Bangladesh (2014–15) to 78.1% in Zambia (2014) (Figure 1). Across all ITC countries, knowledge that smoking causes lung cancer decreased by 0.7 percentage points (PP) per year. Knowledge increased significantly in three countries: China (+1.2 PP per year), the Netherlands (+0.9 PP per year), and Bangladesh (+0.6 PP per year). Knowledge decreased significantly in three countries: Japan (-2.0 PP per year), Germany (-0.6 PP per year), and France (-0.5 PP per year) (Figure 1). There was no significant change in knowledge in all other countries. Detailed results for longitudinal trends in knowledge about smoking-caused diseases in ITC countries are provided in Supplementary table 7.

Figure 1. Percentage of adults who smoke who know or believe that smoking causes lung cancer,† in 25 ITC countries,‡ 2002–2022.

At the most recent survey across 32 GATS countries, 91.9% of adults who smoke overall knew that smoking causes lung cancer, ranging from 98.4% in Argentina (2012) to 56.6% in Nigeria (2012) (Figure 2). Across the 12 GATS countries with at least two years of data, knowledge that smoking causes lung cancer decreased by 0.2 PP per

For peer review only - http://bmjopen.bmj.com/site/about/guidelines.xhtml

year. Knowledge increased significantly in two countries: India (+0.8 PP per year), and Bangladesh (+0.5 PP per year). Knowledge decreased significantly in three countries: Romania (–1.3 PP per year), the Russian Federation (–1.2 PP per year), and Turkey (–0.4 PP per year) (Figure 2). There was no significant change in knowledge in all other countries. For the 20 GATS countries with only one year of data, the change is presented as zero. Detailed results for longitudinal trends in knowledge about smoking-caused diseases in GATS countries are provided Supplementary table 8.

Figure 2. Percentage of adults who smoke who know or believe that smoking causes lung cancer, in 32 GATS countries,† 2008–2018.

At the most recent survey across 25 ITC countries, 84.7% of adults who smoke knew that smoking causes heart disease, ranging from 94.9% in Bangladesh (2014–15) to 57.7% in China (2013–15) (Figure 3). Across all ITC countries, knowledge that smoking causes heart disease decreased by 0.3 PP per year. Knowledge increased significantly in five countries: China (+2.2 PP per year), Kenya (+2.2 PP per year), India (+1.2 PP per year), Bangladesh (+1.2 PP per year), and Spain (+1.1 PP per year). Knowledge decreased significantly in four countries: Japan (–4.3 PP per year), Germany (–1.4 PP per year), Uruguay (–0.7 PP per year), and France (–0.5 PP per year) (Figure 3). There was no significant change in knowledge in all other countries.

Figure 3. Percentage of adults who smoke who know or believe that smoking causes heart disease,† in 25 ITC countries,‡ 2002–2022.

At the most recent survey across 32 GATS countries, 80.0% of adults who smoke knew that smoking causes heart attack, ranging from 95.3% in Turkey (2016) to 46.4% in China (2018) (Figure 4). Across all GATS countries with at least two years of data,

For peer review only - http://bmjopen.bmj.com/site/about/guidelines.xhtml

knowledge that smoking causes heart attack increased by 0.5 PP per year. Knowledge increased significantly in four countries: Viet Nam (+2.2 PP per year), India (+1.9 PP per year), the Philippines (+1.0 PP per year), and Bangladesh (+0.6 PP per year). Knowledge decreased significantly in Romania (–2.1 PP per year) (Figure 4). There was no significant change in knowledge in all other countries.

Figure 4. Percentage of adults who smoke who know or believe that smoking causes heart attack, in 32 GATS countries,† 2008–2018.

At the most recent survey across 25 ITC countries, 75.0% of adults who smoke knew that smoking causes stroke, ranging from 93.9% in Bangladesh (2014–15) to 37.3% in China (2013–15) (Figure 5). Across all ITC countries, knowledge that smoking causes stroke increased by 1.6 PP per year. Knowledge increased significantly in 11 countries: Thailand (+5.7 PP per year), Greece (+5.5 PP per year), Mauritius (+4.6 PP per year), Hungary (+4.5 PP per year), Kenya (+3.9 PP per year), Uruguay (+2.8 PP per year), China (+2.5 PP per year), Mexico (+2.2 PP per year), Republic of Korea (+2.1 PP per year), the Netherlands (+1.4 PP per year), and India (+1.3 PP per year). Knowledge decreased significantly in Germany (–1.0 PP per year) (Figure 5). There was no significant change in knowledge in all other countries.

Figure 5. Percentage of adults who smoke who know or believe that smoking causes stroke,† in 25 ITC countries,‡ 2002–2022.

At the most recent survey across 32 GATS countries, 66.1% of adults who smoke overall knew that smoking causes stroke, ranging from 94.1% in Bangladesh (2017) to 29.6% in Ethiopia (2016) (Figure 6). Across all GATS countries with at least two years of data, knowledge that smoking causes stroke increased by 0.5 PP per year.

For peer review only - http://bmjopen.bmj.com/site/about/guidelines.xhtml

Knowledge increased significantly in five countries: India (+2.3 PP per year), China (+1.3 PP per year), the Russian Federation (+1.2 PP per year), and Mexico (+1.2 PP per year), and Bangladesh (+1.0 PP per year). Knowledge decreased significantly in Romania (–2.0 PP per year) (Figure 6). There was no significant change in knowledge in all other countries.

Figure 6. Percentage of adults who smoke who know or believe that smoking causes stroke, in 32 GATS countries,† 2008–2018.

Knowledge about the risks of SHS exposure causing lung cancer and heart disease/heart attack

At the most recent survey across 26 ITC countries, 77.3% of adults who smoke knew that SHS causes lung cancer in people who do not smoke, ranging from 96.8% in Thailand (2012) to 58.4% in Germany (2018) (Figure 7). Across all ITC countries, knowledge that SHS causes lung cancer in people who do not smoke decreased by 0.1 PP per year. Knowledge increased significantly in five countries: Australia (+2.2 PP per year), China (+2.0 PP per year), India (+1.7 PP per year), Bangladesh (+1.4% PP per year), and Thailand (+0.8 PP per year). Knowledge decreased significantly in four countries: Germany (–2.0 PP per year), New Zealand (–1.5 PP per year), France (–1.5 PP per year), and Uruguay (–1.2 PP per year) (Figure 7). There was no significant change in knowledge in all other countries. Detailed results for longitudinal trends in knowledge about SHS-caused diseases in ITC countries are provided in Supplementary table 9.

Figure 7. Percentage of adults who smoke who know or believe that secondhand smoke causes lung cancer, in 26 ITC countries,† 2003–2022.

For peer review only - http://bmjopen.bmj.com/site/about/guidelines.xhtml

At the most recent survey across 18 GATS countries, 82.1% of adults who smoke knew that SHS causes lung cancer in people who do not smoke, ranging from 96.4% in Thailand (2011) to 41.9% in Nigeria (2012) (Figure 8). Across all GATS countries with at least two years of data, there was no change in knowledge that SHS causes lung cancer in people who do not smoke. Knowledge increased significantly in Viet Nam (+2.7 PP per year) (Figure 8). There was no significant change in knowledge in all other countries. Detailed results for longitudinal trends in knowledge about SHS-caused diseases in GATS countries are provided in Supplementary table 10.

Figure 8. Percentage of adults who smoke who know or believe that secondhand smoke causes lung cancer, in 18 GATS countries,† 2009–2018.

In the most recent survey administered across 24 ITC countries, 61.8% of adults who smoke knew that SHS causes heart attack in people who do not smoke, ranging from 88.5% in Bangladesh (2014–15) to 38.4% in Australia (2013) (Figure 9). Across all ITC countries, knowledge that SHS causes heart attack in people who do not smoke increased by 0.2 PP per year. Knowledge increased significantly in five countries: Bangladesh (+7.0 PP per year), India (+2.7 PP per year), the Netherlands (+1.6 PP per year), Kenya (+2.2 PP per year), and China (+2.1 PP per year). Knowledge decreased significantly in four countries: Australia (–3.9 PP per year), Malaysia (–3.3 PP per year), Japan (–2.7 PP per year), and New Zealand (–2.5 PP per year) (Figure 9). There was no significant change in knowledge in all other countries.

Figure 9. Percentage of adults who smoke who know or believe that secondhand smoke causes heart attack,† in 24 ITC countries,‡ 2008–2022.

For peer review only - http://bmjopen.bmj.com/site/about/guidelines.xhtml

At the most recent survey across 18 GATS countries, 72.5% of adults who smoke knew that SHS causes heart disease in people who do not smoke, ranging from 93.5% in Bangladesh (2017) to 21.3% in Viet Nam (2015) (Figure 10). Across all GATS countries with at least two years of data, knowledge that SHS causes heart disease in people who do not smoke decreased by 0.1 PP per year. Knowledge increased significantly in Viet Nam (+1.4 PP per year) (Figure 10). There was no significant change in knowledge in all other countries.

Figure 10. Percentage of adults who smoke who know or believe that secondhand smoke causes heart disease, in 18 GATS countries,† 2009–2018.

Comparison of knowledge levels among respondents in ITC and GATS countries

Compared with overall knowledge that smoking causes lung cancer (ITC=92.2%, GATS=91.9%), knowledge that smoking causes heart attack (ITC=84.7%)/heart disease (GATS=80.0%) and stroke (ITC=75.0%, GATS=66.1%) was lower, as was knowledge about SHS harms for people who do not smoke (SHS causes lung cancer: ITC=77.3%, GATS=82.1%; SHS causes heart attack (ITC=61.8%)/heart disease (GATS=72.5%) (all $p<0.0001$) (Supplementary table 5).

Knowledge that smoking causes stroke was the only one of the five harms where there was an increase in both ITC and GATS over time (+1.6 PP in ITC; +0.5 PP in GATS). Knowledge that smoking causes lung cancer was the only health harm where there was a decrease in both ITC and GATS (–0.7 PP in ITC; –0.2 PP in GATS). The average of estimated net change in knowledge across the five health harms was +0.14 PP in both ITC and GATS (Supplementary table 6).

Correlation of ITC and GATS data

For peer review only - http://bmjopen.bmj.com/site/about/guidelines.xhtml

In the 12 overlapping ITC and GATS countries, significant positive correlations were found between ITC and GATS estimates of knowledge among adults who smoke that smoking causes lung cancer (r=.80, p=.002), heart disease/heart attack (r=.81, p=.001), and stroke (r=.80, p=.002). For the seven overlapping ITC and GATS countries where knowledge of SHS harms was assessed, moderate but non-significant correlations were found between ITC and GATS estimates of knowledge that SHS causes lung cancer (r=.69, p=.09) and heart disease/heart attack (r=.60, p=.16) in people who do not smoke.

DISCUSSION

Overall, there was a strong correlation between ITC and GATS knowledge estimates in countries where both surveys were conducted. There were five key findings from our analyses about knowledge of smoking- and SHS-caused diseases in 46 countries across two decades. First, adults who smoke were more likely to know that smoking elevates their risk for lung cancer compared with heart disease/heart attack and stroke. Second, adults who smoke were less likely to know about the risks related to SHS. Third, adults who smoke had higher knowledge that SHS causes lung cancer compared with heart disease/heart attack in people who do not smoke. Fourth, knowledge for smoking- and SHS-caused diseases has improved in several countries over time; however, knowledge has generally remained unchanged in most countries and has regressed in others. Finally, there was a near-zero net change over time in knowledge about smoking- and SHS-caused diseases (+0.14% per year across five disease outcomes in ITC and GATS countries).

For peer review only - http://bmjopen.bmj.com/site/about/guidelines.xhtml

There has been a steady upward trend in the global burden of CVD from 1990 to 2019 ─ with the total number of CVD deaths rising from 12.1 million to 18.6 million, and the total number of disability-adjusted life years (DALYs) due to CVD rising from 17.7 million to 34.4 million over the last three decades.[30] The largest number of premature non-communicable disease (NCD) deaths globally are caused by CVDs [31,32]. for which smoking is a major modifiable risk factor. In 2019, heart disease and stroke collectively accounted for 2.6 million smoking-attributable deaths worldwide, compared to 1.3 million deaths from lung, tracheal, and bronchus cancer.[33] A 2024 study found that exposure to SHS significantly increased risk for nine health outcomes, with the greatest risk observed for CVD.[34] Although smoking and SHS are known risk factors for CVD, adults in many countries still lack knowledge about their harmful effects on cardiovascular health. Consistent with previous studies [12,14,15], we found that adults who smoke were more likely to know that smoking causes lung cancer than heart disease and stroke. CVD is still the leading preventable cause of mortality worldwide [30,35] and while the relative risk from smoking for CVD is lower than for lung cancer, more people die prematurely from CVD than from lung cancer caused by smoking.[35,36]

SHS is one of the leading causes of preventable death [37], killing an estimated 1.3 million people who do not smoke in 2021. [38] We found that across 40 countries, about 65.0% of adults who smoke knew that SHS exposure causes heart disease/heart attack in people who do not smoke. Notably, even in several high-income ITC countries ─ including Australia, Germany, England, the US, Aotearoa/New Zealand, Canada and

For peer review only - http://bmjopen.bmj.com/site/about/guidelines.xhtml

Hungary ─ less than half of adults who smoke knew of this risk, in contrast with higher knowledge that active smoking causes heart disease and stroke.

Knowledge that smoking causes stroke significantly increased in 11 ITC countries and three GATS countries between 2002 and 2022. Nevertheless, there were no significant changes in knowledge about stroke in 13 ITC countries and seven GATS countries. Likewise, there were no significant changes in knowledge among adults who smoke that smoking causes heart disease/heart attack in people who smoke, and that SHS exposure causes heart disease/heart attack in people who do not smoke in 14 ITC countries and 5 GATS countries, respectively. While significant gains in knowledge about the CVD risks of smoking tobacco and/or SHS exposure were observed in several LMICS such as Bangladesh, India, Thailand, and Viet Nam, knowledge levels have remained low in others such as Nigeria and Ethiopia. Significant declines in knowledge about some of these risks were also observed in several high-income countries, where baseline risk knowledge was relatively high, including Germany and Japan. This suggests that measures to educate people about the diseases caused by smoking may have a greater impact in countries where knowledge is low compared with those where knowledge is already high. It is key for countries to tailor interventions to address knowledge gaps.

Country-level differences observed may reflect differences in public health education strategies and implementation methods about the harms of smoking. For example, health warnings on cigarette packs, size of warnings, and languages used are one way of informing people about diseases caused by smoking, which may encourage those who smoke to reduce consumption or take steps to quit.[39–41] It is possible that

For peer review only - http://bmjopen.bmj.com/site/about/guidelines.xhtml

people who smoke may have higher levels of knowledge for specific health risks of smoking that were depicted on cigarette pack warnings at the time of ITC and GATS country surveys, compared to health risks that were not addressed by the warnings. However, knowledge alone may be insufficient to change behavior. Social Cognitive Theory provides one theoretical framework for understanding the underlying mechanisms of health behaviors and informing the development of interventions, whereby behavior is determined by reciprocal interactions between personal, environmental, and behavioral factors. [42] For example, exposure to health warnings on cigarette packs in combination with knowledge of smoking- and SHS-caused diseases, self-efficacy to reduce or quit smoking, supportive environments such as smoke-free homes, workplaces, and public places, can facilitate smoking cessation and protection against SHS exposure. Future research should examine the associations between knowledge of smoking-caused diseases and health warnings (type (text-only or pictorial), content, size, and location (front and/or back of pack)) as well as other tobacco control policies, which was beyond the scope of the current study.

Since the WHO FCTC came into force in 2005, countries around the world have made significant progress in educating their populations about the dangers of tobacco use and SHS exposure through the implementation of tobacco advertising, promotion and sponsorship bans,[43] health warnings on tobacco packaging,[44] smoke-free laws,[45] and anti-tobacco campaigns,[44] which have likely contributed to reductions in the tobacco use prevalence from 29.3% in 2005 to 20.9% in 2022.[45,46] However, we found no overall change (+0.14% per year) in knowledge across five smoking and SHS-caused diseases in both ITC and GATS countries over a two-decade period, even

For peer review only - http://bmjopen.bmj.com/site/about/guidelines.xhtml

though 43 of the 46 countries included in the present study are WHO FCTC Parties.[47] This may reflect an uneven pace in the implementation of MPOWER measures— including taxation, health warnings on cigarette packs and mass media campaigns — that are especially effective for communicating smoking-caused risks [45] and most impactful when they are integrated as a part of a comprehensive tobacco control strategy. [48] For example, the impact of smoke-free laws on knowledge about SHS-caused diseases could potentially be strengthened by complementary mass media campaigns, which have been shown to increase knowledge of SHS risks, reduce smoking in homes, and promote smoking cessation. [23]

Tobacco control policies may be more effective at improving knowledge about smoking and SHS-caused diseases when supported by strong implementation, enforcement, and compliance; however, substantial gaps remain. Studies have found weak implementation, limited accountability, and poor enforcement of smoke-free laws in LMICs after WHO FCTC ratification,[49] and a 2024 systematic review and meta-analysis reported high levels of non-compliance with smoke-free laws, particularly in public places and workplaces.[50] These findings align with an assessment of the WHO FCTC at its 20th anniversary, which concluded that progress has slowed despite earlier gains, due to tobacco industry interference, limited political will and resources, and the rise of new nicotine products, highlighting the need for best-practice implementation, regulation, and intergovernmental cooperation. [51]

It is also important to note that reductions in mortality and morbidity from smoking- and SHS-caused diseases may influence public perceptions of their harms, particularly in countries with declining smoking prevalence. Moreover, advances in

For peer review only - http://bmjopen.bmj.com/site/about/guidelines.xhtml

healthcare and effective treatments in high-resource countries could reduce the disease burden and improve quality of life even in the absence of smoking prevention policies, potentially contributing to low population knowledge about the harms of smoking and SHS. This underscores the importance of prioritizing resources for tobacco use prevention rather than focusing solely on treatment.

This study has limitations. First, self-reported data may be subject to recall bias that might result in over- or under-estimation of knowledge or social desirability bias. Second, some ITC survey response rates were low due to the nature of the mode and/or protocols for data collection (Supplementary table 1), which may limit the generalizability of the study findings. Third, we found statistically significant positive correlations for all three smoking-caused diseases in the 12 overlapping ITC and GATS countries, while non-significant correlations were found for the two SHS-caused diseases due to reduced power from the small sample size (N=7 observations). Caution is warranted in the comparison of ITC and GATS estimates due to differences in research designs, sampling across ITC Surveys, and between ITC Surveys and GATS, survey year(s) in overlapping ITC and GATS countries, and ITC Survey and GATS questions used to measure knowledge (Supplementary table 1, Supplementary table 2, Supplementary table 4). However, correlation of the ITC and GATS data despite their different research and methodological designs, allows for more confident inferences.[52][53] Fourth, ITC and GATS data correlation analyses were limited to 12 countries, so the results are not generalizable. Finally, our results are limited to knowledge about the harms of smoking among adults who currently smoke tobacco. Our finding for an overall near-zero net change in knowledge over time may or may not reflect changes in overall

For peer review only - http://bmjopen.bmj.com/site/about/guidelines.xhtml

knowledge in the population. It is possible that while knowledge about diseases caused by smoking and SHS among adults who smoke did not change over time, knowledge may have increased among adults who have quit smoking and those who have never smoked. As such, we examined whether changes in knowledge for each disease outcome differed by smoking status across 12 GATS countries where more than one survey wave was conducted among adults who smoke, adults who have quit smoking, and adults who have never smoked.[d] In general, there were no consistent patterns for changes in knowledge about each disease outcome across the 12 GATS countries (Supplementary table 5). Non-significant increases/decreases in knowledge over time among adults who quit and those who never smoked suggests that this pattern smokers is consistent with that in the overall population.

This study found high knowledge that smoking causes lung cancer among adults who smoke, lower knowledge that smoking causes CVD, and even lower knowledge for SHS harms. There was no overall change in knowledge over two decades. The United Nations 2030 Agenda for Sustainable Development calls for strengthening implementation of the WHO FCTC to achieve the target for a one-third reduction in premature mortality from NCDs by 2030.[54] To achieve this, countries could consider implementation of WHO FCTC measures shown to have synergistic effects on promoting smoking cessation and reducing smoking prevalence.[55,56] An important

[d]ITC longitudinal cohort surveys primarily recruit adults who smoke at baseline. Respondents who quit smoking at subsequent survey waves may be recontacted as part of the longitudinal cohort; however, these individuals do not constitute a population-representative sample of adults who have quit smoking. Although these respondents retain adults who smoke-based survey weights for longitudinal analyses (to represent the previous-wave population of adults who smoke), they are analytically classified as adults who quit and are not included as adults who currently smoke in our analyses. For this reason, analyses comparing changes in knowledge by smoking status (adults who currently, formerly, and never smoked) were conducted using GATS data only.

For peer review only - http://bmjopen.bmj.com/site/about/guidelines.xhtml

factor that can facilitate efforts to strengthen policies is to increase the awareness of the harms of using cigarettes and other smoked tobacco products.

CONCLUSION

Across 46 countries over two decades, adults who smoke had high knowledge that smoking causes lung cancer, substantially lower knowledge of cardiovascular harms, and even lower awareness of SHS-caused diseases. The near-zero net change in knowledge over time underscores the need for comprehensive, well-implemented, and enforced tobacco control strategies ─ particularly health warnings, mass media campaigns, and smoke-free policies ─ to address knowledge gaps and accelerate progress toward global NCD prevention targets.

For peer review only

For peer review only - http://bmjopen.bmj.com/site/about/guidelines.xhtml

Competing interest statement: KMC has in the past and continues to serve as a paid witness in litigation filed against cigarette manufacturers. GTF has been an expert witness or consultant for governments defending their country's policies or regulations in litigation. CG has been a paid expert witness for the Australian Government in litigation concerning nicotine product marketing. KK received a JMWH Bayer Grant from Japan Society for Menopause and Women's Health. ASAN has received an unconditional educational grant from Johnson & Johnson Malaysia Sdn. Bhd. JFT has served as paid expert witness in legal challenges against tobacco and vaping companies. All other authors have no conflicts of interest to declare.

Contributorship Statement

JCH acted as guarantor. JCH and GTF led the conceptualization of the study. JCH wrote the original draft manuscript and prepared the final version. GM conducted the data analysis. EOI prepared the original figures and JCH prepared the final versions. GTF, GM, LVC, EOI, ACK, JS, IBA and the ITC Project Collaborators contributed to review and editing.

For peer review only

References

1 Murray CJL, Aravkin AY, Zheng P, *et al.* Global burden of 87 risk factors in 204 countries and territories, 1990–2019: a systematic analysis for the Global Burden of Disease Study 2019. *The Lancet*. 2020;396:1223–49. doi: 10.1016/S0140-6736(20)30752-2

2 World Health Organization. WHO Framework Convention on Tobacco Control. 2003.

3 World Health Organization. MPOWER. A policy package to reverse the tobacco epidemic. Geneva 2008.

4 Hammond D. Health warning messages on tobacco products: a review. *Tob Control*. 2011;20:327–37. doi: 10.1136/tc.2010.037630

5 World Health Organization. Tackling NCDs: 'best buys' and other recommended interventions for prevention and control of noncommunicable diseases. Geneva 2017.

6 Brinn M, Carson K, Esterman A, *et al.* Mass media interventions for preventing smoking in young people. *Cochrane Database of Systematic Reviews*. 2010;10:Art. No.: CD001006. doi: 10.1002/14651858.CD001006.pub3

7 Evans KA, Sims M, Judge K, *et al.* Assessing the knowledge of the potential harm to others caused by second-hand smoke and its impact on protective behaviours at home. *J Public Health*. 2012;34:183–94. doi: 10.1093/pubmed/fdr104

8 Yang J, Hammond D, Driezen P, *et al.* Health knowledge and perception of risks among Chinese smokers and non-smokers: findings from the Wave 1 ITC China Survey. *Tob Control*. 2010;19:i18–23. doi: 10.1136/TC.2009.029710

9 Demissie HS, Smith T, de Quevedo IG, *et al.* Factors associated with quit attempt and successful quitting among adults who smoke tobacco in Ethiopia: Global Adult Tobacco Survey (GATS) 2016. *Tob Prev Cessat*. 2022;8:12. doi: 10.18332/tpc/146170

10 Hyland A, Li Q, Bauer JE, *et al.* Predictors of cessation in a cohort of current and former smokers followed over 13 years. *Nicotine Tob Res*. 2004;6:S363-9.

11 Chiosi JJ, Andes L, Asma S, *et al.* Warning about the harms of tobacco use in 22 countries: findings from a cross-sectional household survey. *Tob Control*. 2016;25:393–401. doi: 10.1136/tobaccocontrol-2014-052047

12 Siahpush M, McNeill A, Hammond D, *et al.* Socioeconomic and country variations in knowledge of health risks of tobacco smoking and toxic constituents of smoke: results from the 2002 International Tobacco Control (ITC) Four Country Survey. *Tob Control*. 2006;15:iii65–70. doi: 10.1136/TC.2005.013276

13 Mills SD, Wiesen CA. Beliefs about the health effects of smoking among adults in the United States. *Health Educ Behav*. 2022;49:497–505. doi: 10.1177/10901981211004136

14 Trofor AC, Papadakis S, Lotrean LM, *et al.* Knowledge of the health risks of smoking and impact of cigarette warning labels among tobacco users in six European countries: Findings

For peer review only - http://bmjopen.bmj.com/site/about/guidelines.xhtml

from the EUREST-PLUS ITC Europe Surveys. *Tob Induc Dis*. 2018;16. doi: 10.18332/tid/99542

15 Ahluwalia I, Smith T, Arrazola R, *et al.* Current tobacco smoking, quit attempts, and knowledge about smoking risks among persons aged ≥15 Years - Global Adult Tobacco Survey, 28 Countries, 2008-2016. *MMWR*. doi: 10.15585/MMWR.MM6738A7

16 Rydz E, Telfer J, Quinn EK, *et al.* Canadians' knowledge of cancer risk factors and belief in cancer myths. *BMC Public Health*. 2024;24:329. doi: 10.1186/s12889-024-17832-3

17 Shahab L, McGowan JA, Waller J, *et al.* Prevalence of beliefs about actual and mythical causes of cancer and their association with socio-demographic and health-related characteristics: findings from a cross-sectional survey in England. *Eur J Cancer*. 2018;103:308–16. doi: 10.1016/j.ejca.2018.03.029

18 Sun Y, Wang D, Yi Y, *et al.* Outdoor secondhand smoke exposure in public places frequented by minors in the urban area of Hangzhou City, China: a cross-sectional study. *Tob Induc Dis*. 2024;22. doi: 10.18332/tid/192129

19 Abdul Mutalib RNS, Abd Rani NL, Zulkifli A, *et al.* Knowledge, beliefs, and behaviors related to secondhand smoke and smoking in the home: a qualitative study with men in Malaysia. *Nicotine Tob Res*. 2023;25:821–7. doi: 10.1093/ntr/ntac239

20 Ezekiel E, Tomini S, Odukoya O. Secondhand smoke exposure, perceived risks and knowledge of the national tobacco law among non-smoking adults in outdoor motor parks in an urban area, Lagos, Nigeria. *Tob Induc Dis*. ;16:A491. doi: 10.18332/tid/84641

21 Tattan-Birch H, Jarvis MJ. Children's exposure to second-hand smoke 10 years on from smoke-free legislation in England: cotinine data from the Health Survey for England 1998-2018. *Lancet Reg Health Eur*. 2022;15:100315. doi: 10.1016/j.lanepe.2022.100315

22 Nanninga S, Lhachimi SK, Bolte G. Impact of public smoking bans on children's exposure to tobacco smoke at home: a systematic review and meta-analysis. *BMC Public Health*. 2018;18:749. doi: 10.1186/s12889-018-5679-z

23 Lim CCW, Rutherford B, Gartner C, *et al.* A systematic review of second-hand smoking mass media campaigns (2002-2022). *BMC Public Health*. 2024;24:693. doi: 10.1186/s12889-024-18222-5

24 Chido-Amajuoyi OG, Osaghae I, Agaku IT, *et al.* Exposure to school-based tobacco prevention interventions in low-income and middle-income countries and its association with psychosocial predictors of smoking among adolescents: a pooled cross-sectional analysis of Global Youth Tobacco Survey data from 38 countries. *BMJ Open*. 2024;14:e070749. doi: 10.1136/bmjopen-2022-070749

25 Lonergan BJ, Meaney S, Perry IJ, *et al.* Smokers still underestimate the risks posed by secondhand smoke: a repeated cross-sectional study. *Nicotine Tob Res*. 2014;16:1121–8. doi: 10.1093/ntr/ntu046

For peer review only

26 Thompson ME, Driezen P, Boudreau C, *et al.* Methods of the International Tobacco Control (ITC) EUREST-PLUS ITC Europe Surveys. *Eur J Public Health*. 2020;30:iii4–9. doi: 10.1093/eurpub/ckz212

27 ITC Project. ITC Project. Methods. Technical reports. International Tobacco Control Policy Evaluation Project. Methods. 2024. https://itcproject.org/methods/technical-reports/

[dataset] 28 Global Tobacco Surveillance System. GTSS Academy. Global Tobacco Surveillance System. Academy. 2026. https://www.gtssacademy.org/

[dataset] 29 International Tobacco Control Policy Evaluation Project. Data request forms. 2026. https://itcproject.org/request-data-form/

30 Roth GA, Mensah GA, Johnson CO, *et al.* Global burden of cardiovascular diseases and risk factors, 1990-2019: update from the GBD 2019 Study. *J Am College Cardiology*. 2020;76:2982–3021. doi: 10.1016/j.jacc.2020.11.010

31 National Cancer Institute. Health effects of exposure to environmental tobacco smoke. *Smoking and Tobacco Control Monograph 10*. Bethesda, MD: National Cancer Institute 1999.

32 Barnoya J, Glantz SA. Cardiovascular effects of secondhand smoke: nearly as large as smoking. *Circulation*. 2005;111:2684–98. doi: 10.1161/CIRCULATIONAHA.104.492215

33 Institute for Health Metrics and Evaluation. GBD summaries. Secondhand smoke - Level 3 risk. 2019. https://www.healthdata.org/results/gbd_summaries/2019/secondhand-smoke-level-3-risk

34 Flor LS, Anderson JA, Ahmad N, *et al.* Health effects associated with exposure to secondhand smoke: a Burden of Proof study. *Nat Med*. 2024;30:149–67. doi: 10.1038/s41591-023-02743-4

35 Global Burden of Disease Collaborative Network. Global Burden of Disease Study 2019 (GBD 2019) results. Institute for Health Metrics and Evaluation. 2020. https://vizhub.healthdata.org/gbd-results/

36 Reitsma MB, Kendrick PJ, Ababneh E, *et al.* Spatial, temporal, and demographic patterns in prevalence of smoking tobacco use and attributable disease burden in 204 countries and territories, 1990–2019: a systematic analysis from the Global Burden of Disease Study 2019. *The Lancet*. 2021;397:2337–60. doi: 10.1016/S0140-6736(21)01169-7

37 Öberg M, Jaakkola MS, Woodward A, *et al.* Worldwide burden of disease from exposure to second-hand smoke: a retrospective analysis of data from 192 countries. *The Lancet*. 2011;377:139–46. doi: 10.1016/S0140-6736(10)61388-8

38 Brauer M, Roth G, Aravkin A, *et al.* Global burden and strength of evidence for 88 risk factors in 204 countries and 811 subnational locations, 1990-2021: a systematic analysis for the Global Burden of Disease Study 2021. *The Lancet*. 2024;403:2162–203. doi: 10.1016/S0140-6736(24)00933-4

39 World Health Organization. Tobacco control (TFI). 2021.

For peer review only - http://bmjopen.bmj.com/site/about/guidelines.xhtml

40 Noar S, Francis D, Bridges C, *et al.* The impact of strengthening cigarette pack warnings: systematic review of longitudinal observational studies. *Soc Sci Med*. 2016;164:118–29. doi: 10.1016/j.socscimed.2016.06.011

41 Ngo A, Cheng KW, Shang C, *et al.* Global evidence on the association between cigarette graphic warning labels and cigarette smoking prevalence and consumption. *IJERPH*. 2018;15:421. doi: 10.3390/IJERPH15030421

42 Mantey D, Hunt E, Hoelscher D, *et al. Social Cognitive Theory and health behaviors*. 2024.

43 World Health Organization. WHO report on the global tobacco epidemic, 2013: enforcing bans on tobacco advertising, promotion and sponsorship. Geneva: WHO 2013.

44 World Health Organization. *WHO report on the global tobacco epidemic, 2011: warning about the dangers of tobacco.* World Health Organization 2011.

45 World Health Organization. WHO report on the global tobacco epidemic, 2023: protect people from tobacco smoke. Geneva: World Health Organization 2023.

46 World Health Organization. WHO global report on trends in prevalence of tobacco use 2000–2030. Geneva: World Health Organization 2024.

47 World Health Organization. WHO Framework Convention on Tobacco Control. Parties. WHO Framework Convention on Tobacco Control. 2024. https://fctc.who.int/who-fctc/overview/parties

48 World Health Organization. WHO report on the global tobacco epidemic, 2025. Geneva: World Health Organization 2025.

49 Byron MJ, Cohen JE, Frattaroli S, *et al.* Implementing smoke-free policies in low- and middle-income countries: a brief review and research agenda. *Tob Induc Dis*. 2019;17:60. doi: 10.18332/tid/110007

50 Daba C, Atamo A, Gasheya KA, *et al.* Non-compliance with smoke-free law in public places: a systematic review and meta-analysis of global studies. *Front Public Health*. 2024;12:1354980. doi: 10.3389/fpubh.2024.1354980

51 Gilmore AB, Callard C, Sy D, *et al.* 20th anniversary of the WHO Framework Convention on Tobacco Control coming into force: time for a step change in ambition. *The Lancet*. 2025;405:677–81. doi: 10.1016/S0140-6736(25)00336-8

52 Hill AB. The environment and disease: association or causation? *Proc R Soc Med*. 1965;58:295–300.

53 Campbell DT, Fiske DW. Convergent and discriminant validation by the multitrait-multimethod matrix. *Psychological Bulletin*. 1959;56:81–105. doi: 10.1037/h0046016

54 United Nations. Transforming our world: the 2030 Agenda for Sustainable Development. 2015.

For peer review only

55 Chung-Hall J, Craig L, Gravely S, *et al.* Impact of the WHO FCTC over the first decade: a global evidence review prepared for the Impact Assessment Expert Group. *Tob Control*. 2019;28:s119–28. doi: 10.1136/tobaccocontrol-2018-054389

56 Hoffman SJ, Tan C. Overview of systematic reviews on the health-related effects of government tobacco control policies. *BMC Public Health*. 2015;15:744. doi: 10.1186/s12889-015-2041-6

For peer review only

For peer review only - http://bmjopen.bmj.com/site/about/guidelines.xhtml

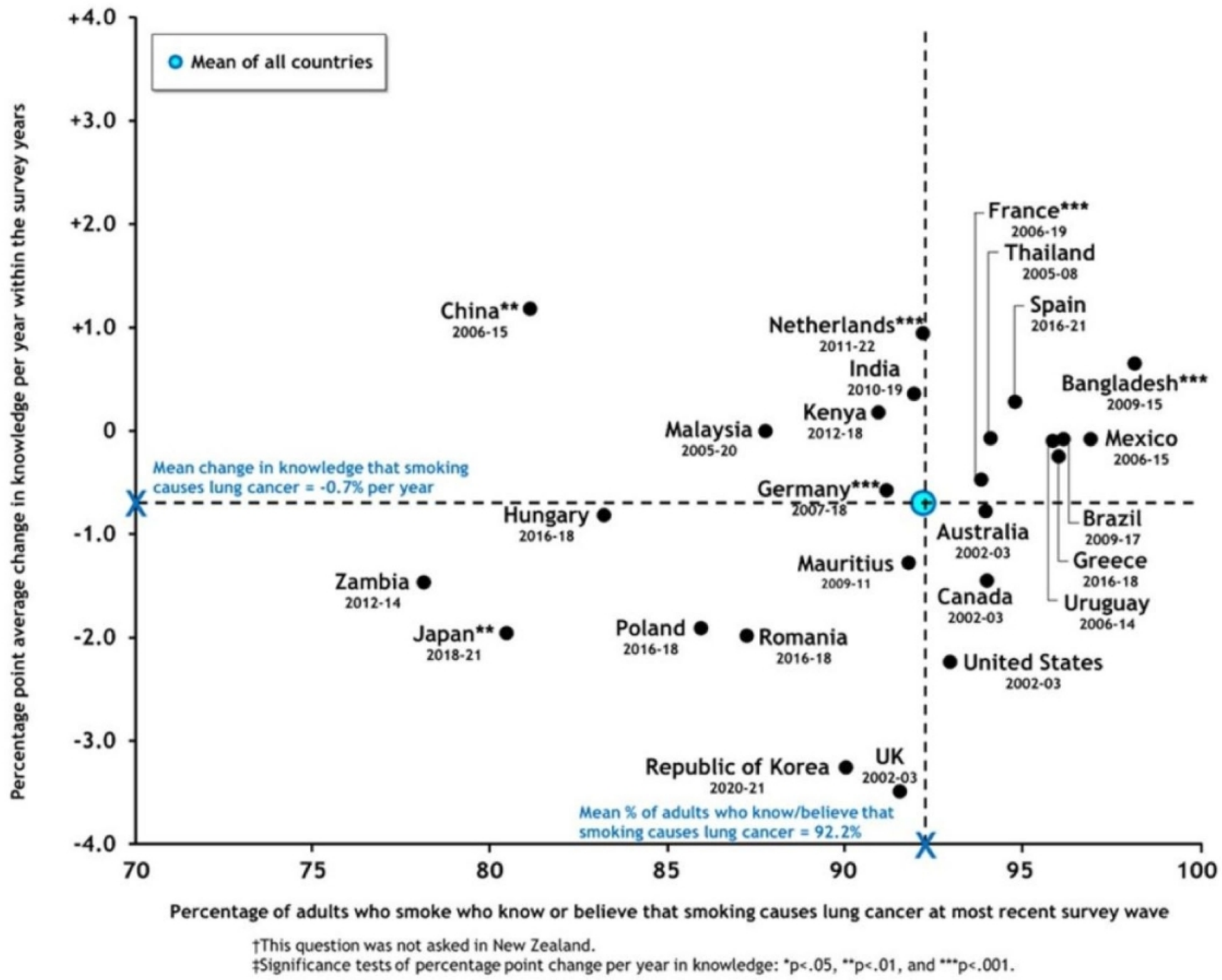

Figure 1. Percentage of adults who smoke who know or believe that smoking causes lung cancer,† in 25 ITC countries,‡ 2002–2022.

148x129mm (300 x 300 DPI)

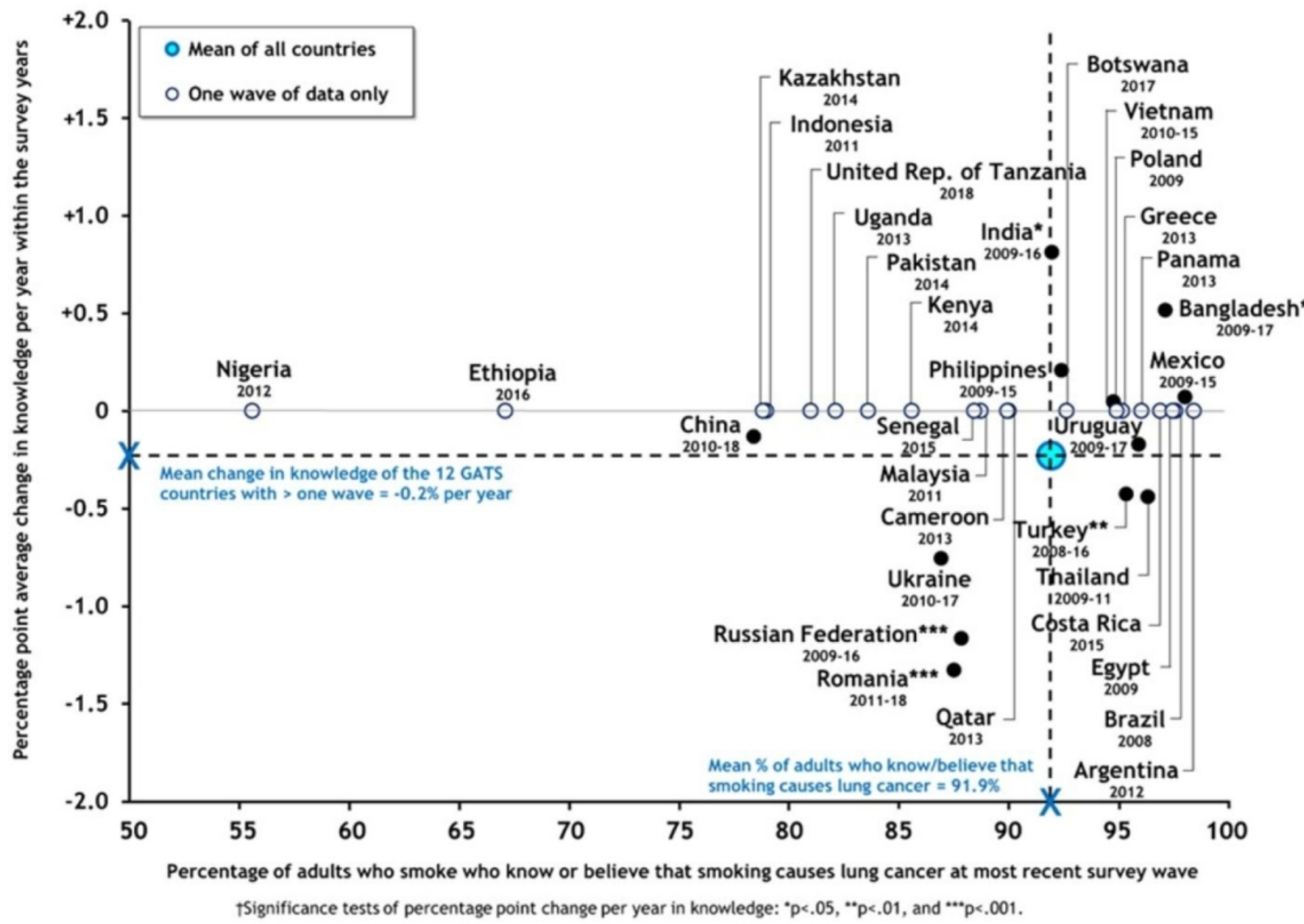

Figure 2. Percentage of adults who smoke who know or believe that smoking causes lung cancer, in 32 GATS countries,† 2008–2018.

123x94mm (300 x 300 DPI)

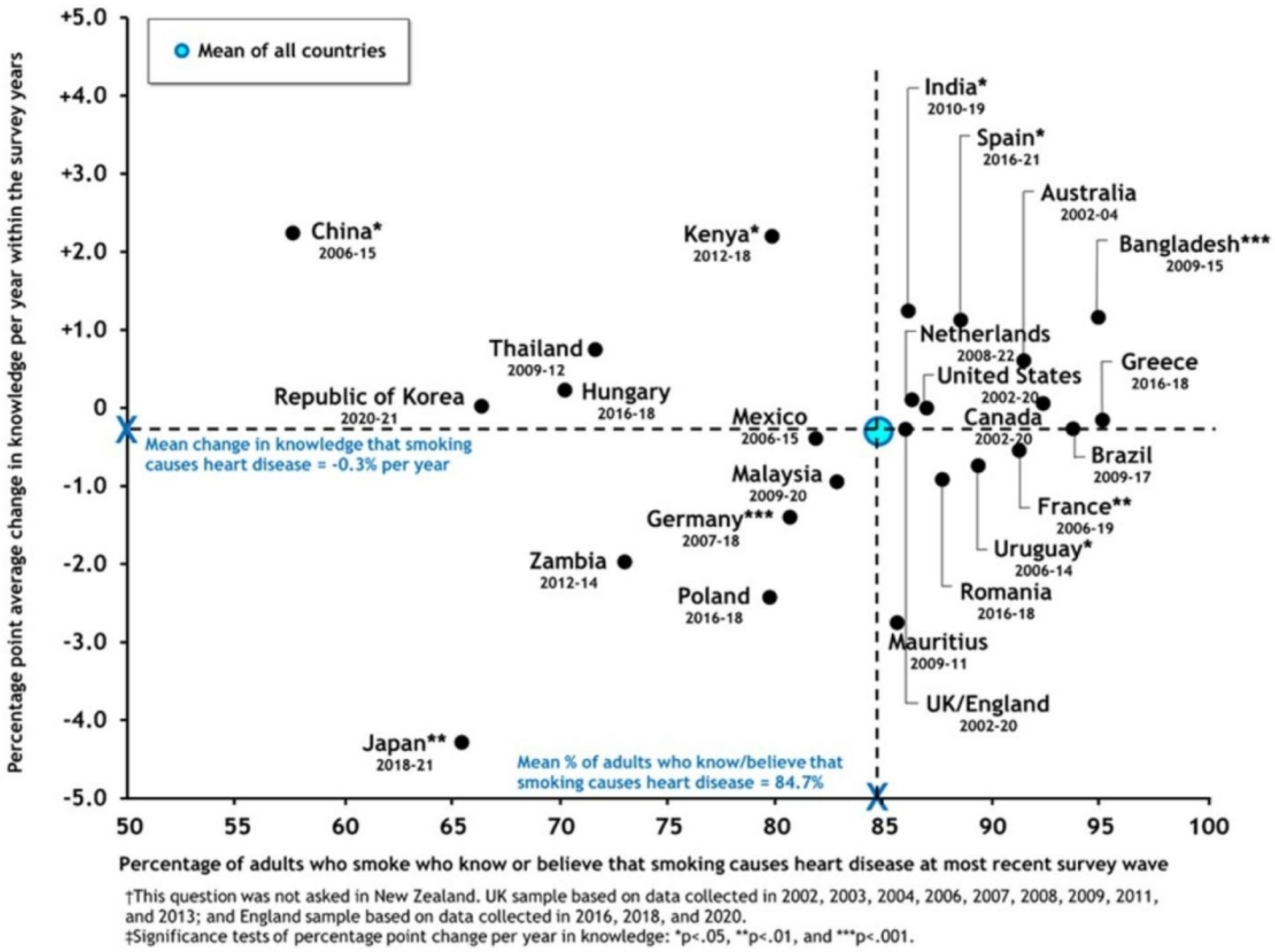

Figure 3. Percentage of adults who smoke who know or believe that smoking causes heart disease,† in 25 ITC countries,‡ 2002–2022.

123x99mm (300 x 300 DPI)

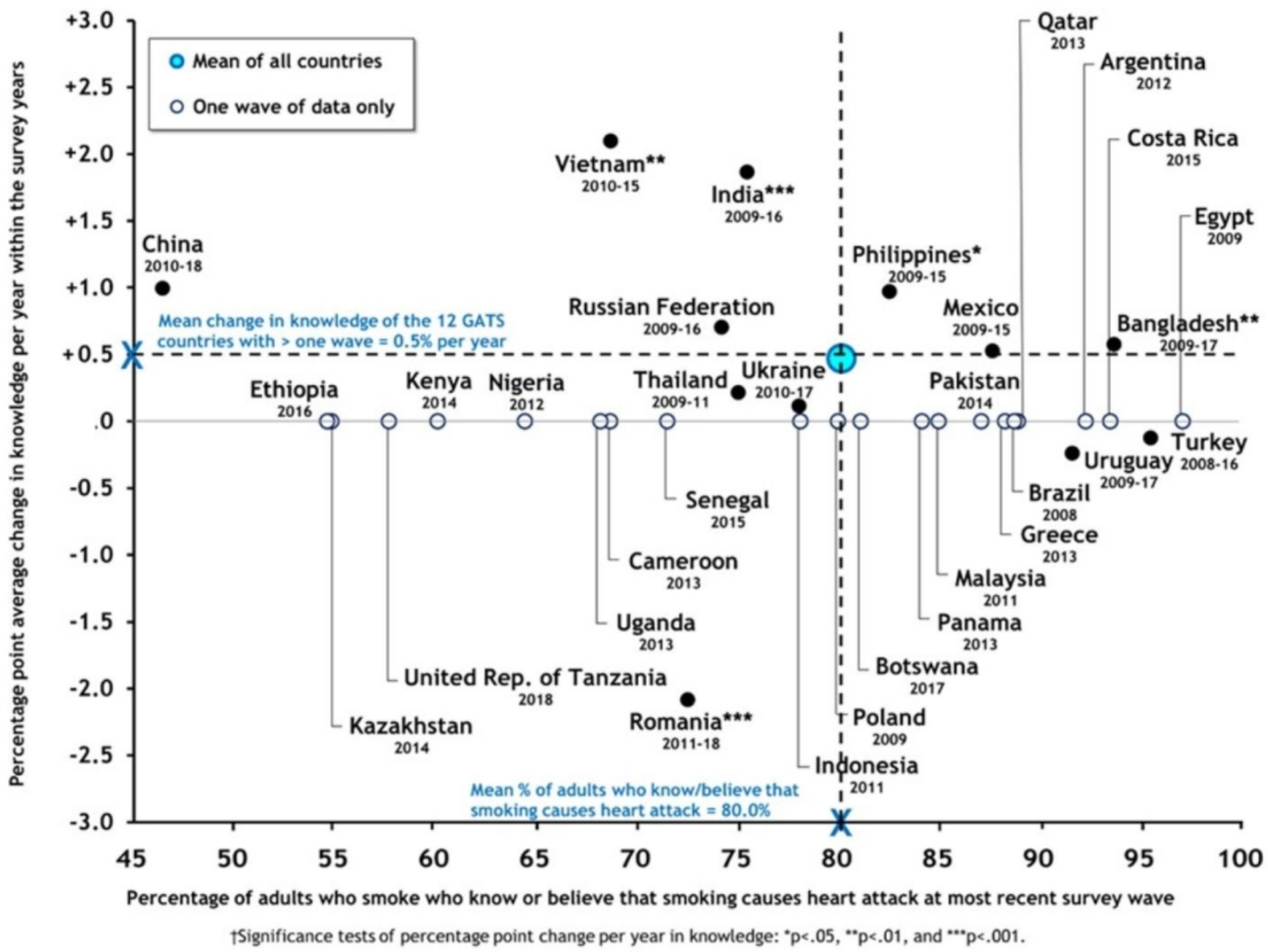

Figure 4. Percentage of adults who smoke who know or believe that smoking causes heart attack, in 32 GATS countries,† 2008–2018.

123x99mm (300 x 300 DPI)

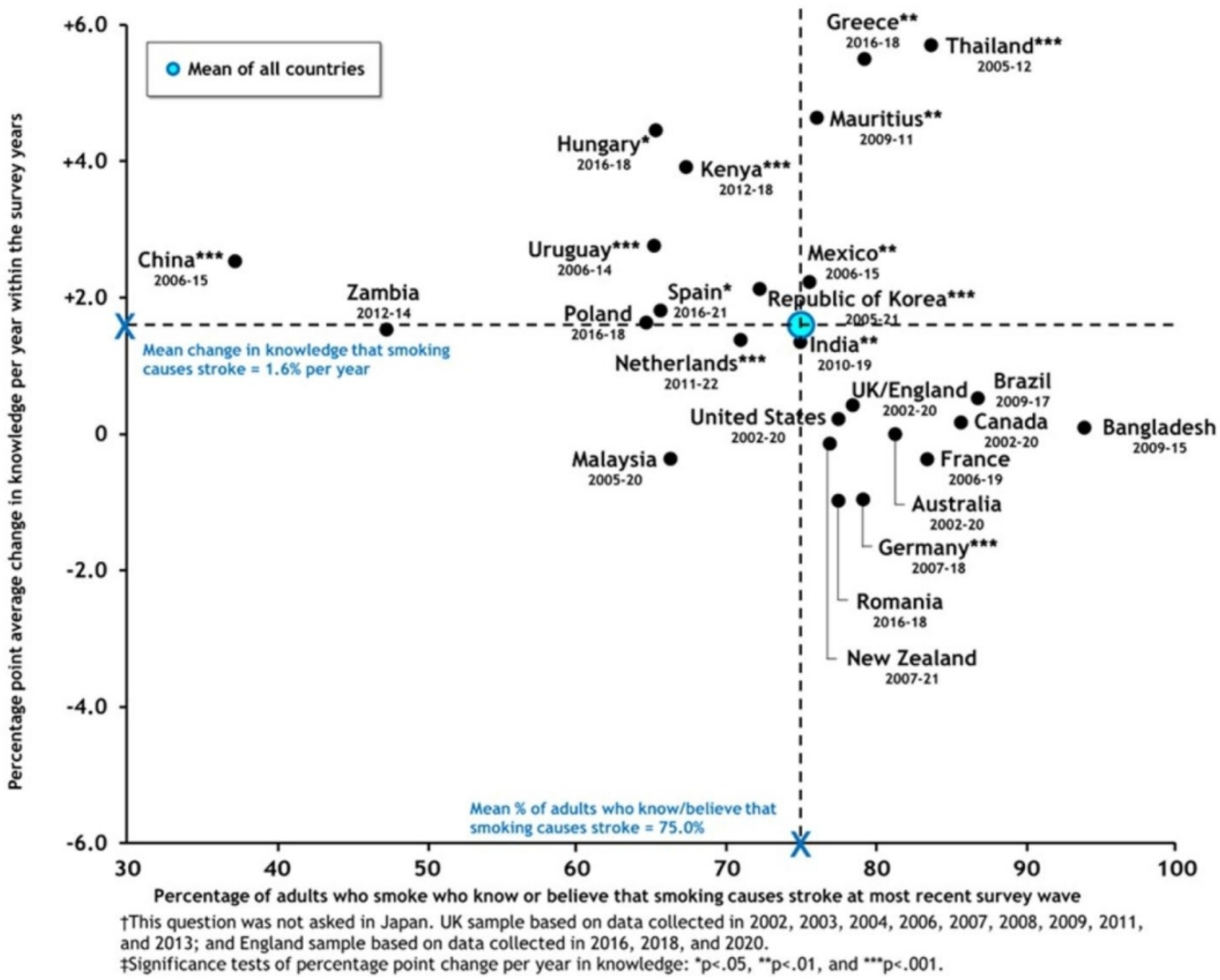

Figure 5. Percentage of adults who smoke who know or believe that smoking causes stroke,† in 25 ITC countries,‡ 2002–2022.

123x106mm (300 x 300 DPI)

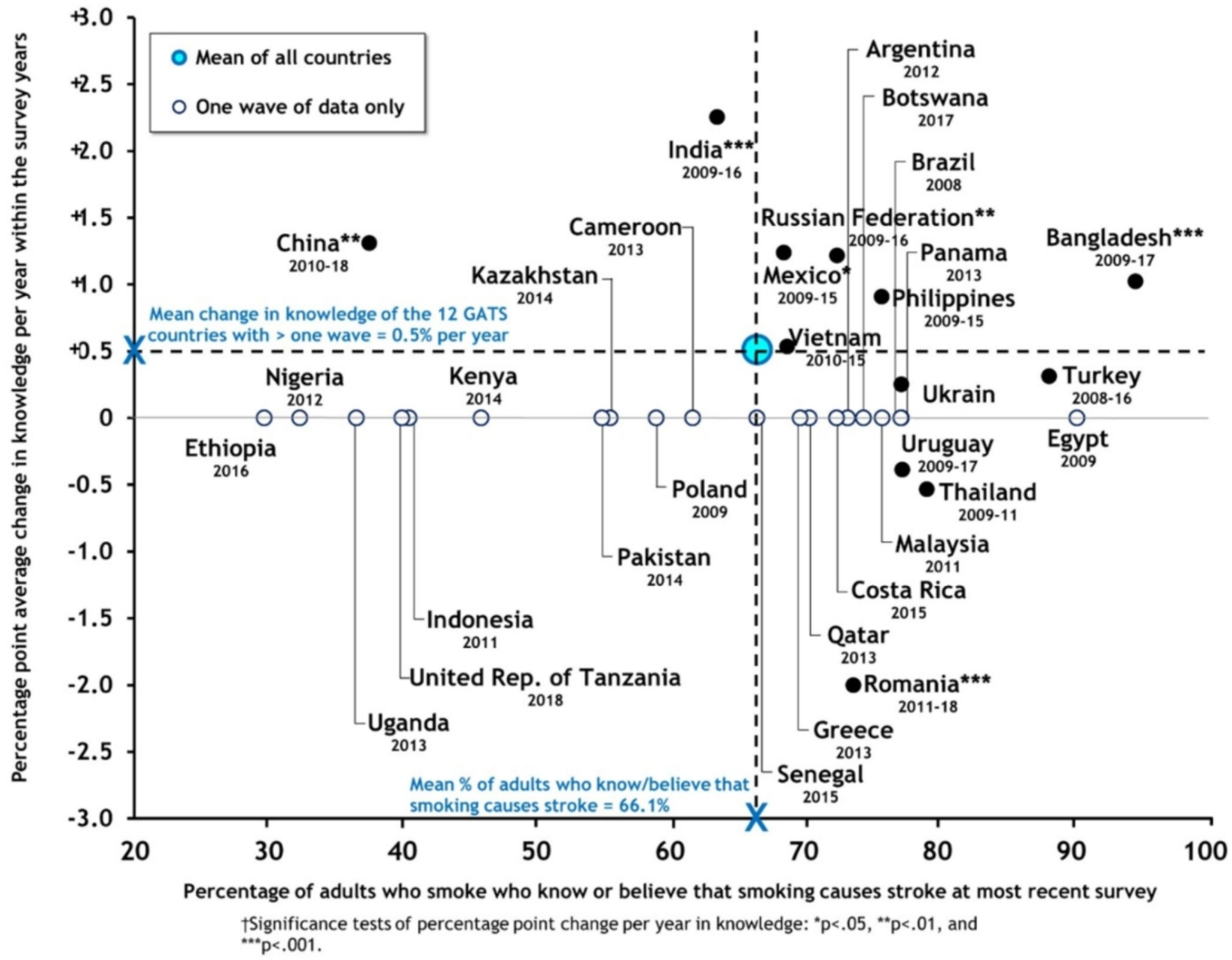

Figure 6. Percentage of adults who smoke who know or believe that smoking causes stroke, in 32 GATS countries,† 2008–2018.

181x152mm (300 x 300 DPI)

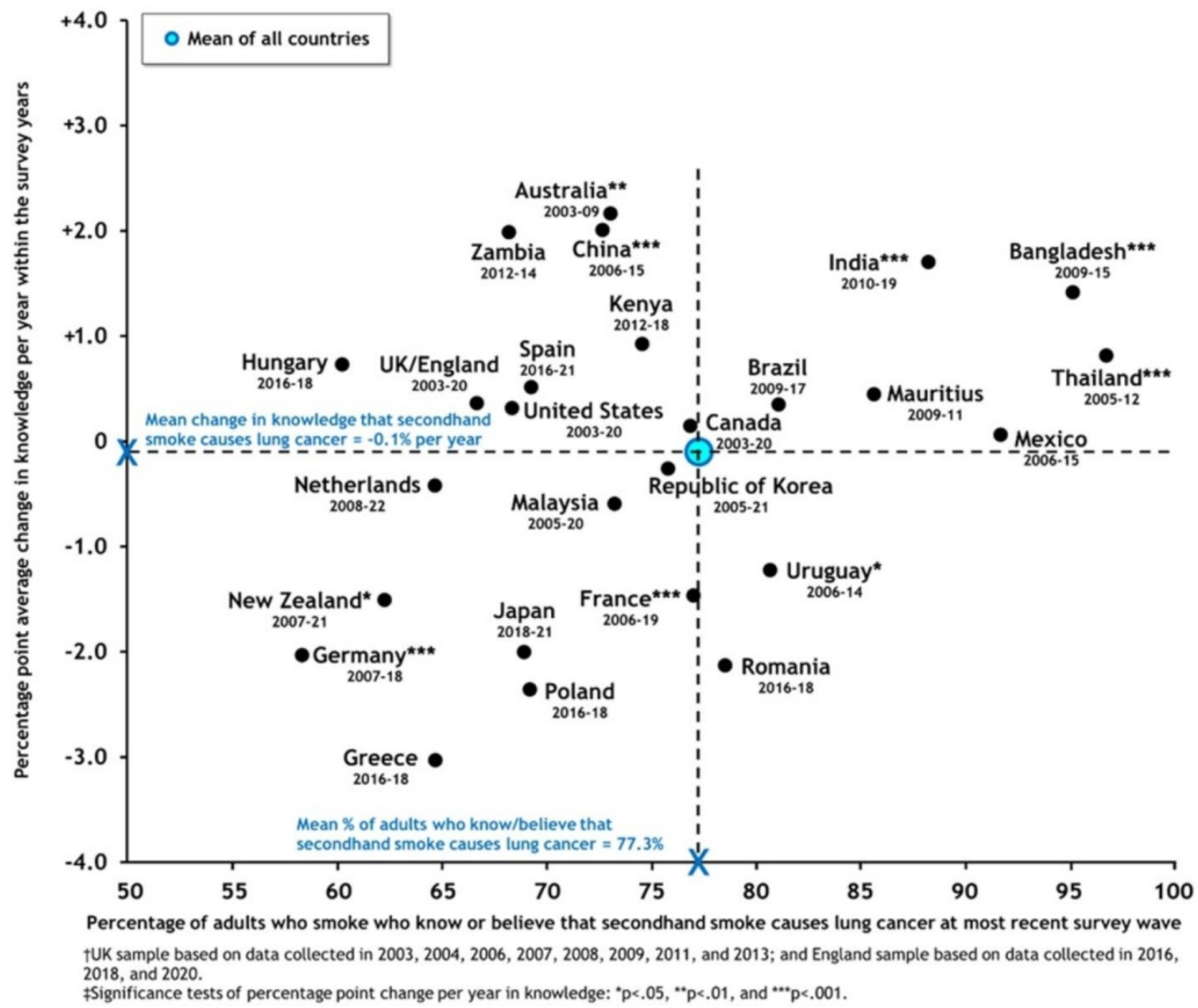

Figure 7. Percentage of adults who smoke who know or believe that secondhand smoke causes lung cancer, in 26 ITC countries,† 2003–2022.

123x112mm (300 x 300 DPI)

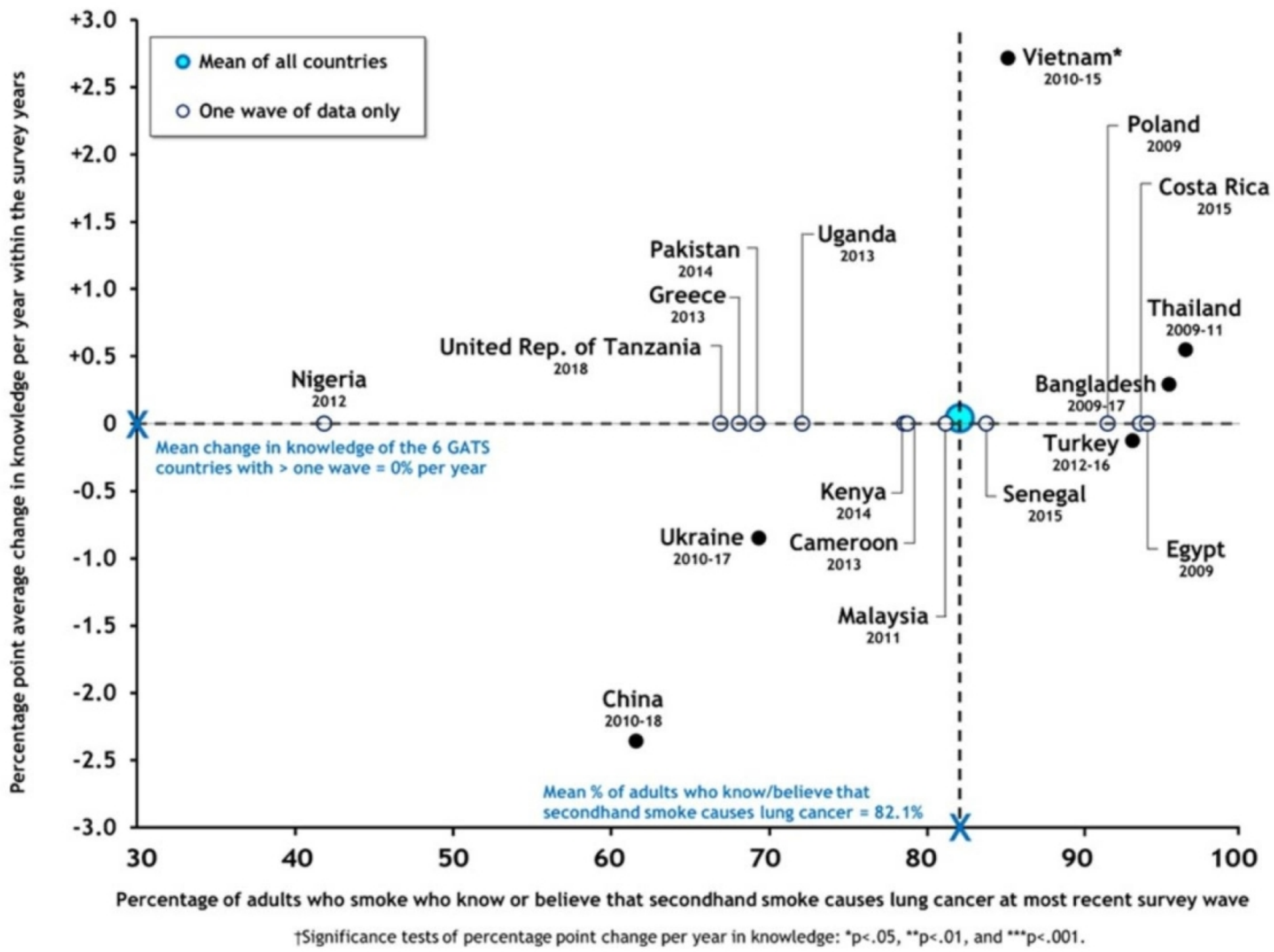

Figure 8. Percentage of adults who smoke who know or believe that secondhand smoke causes lung cancer, in 18 GATS countries,† 2009–2018.

123x93mm (300 x 300 DPI)

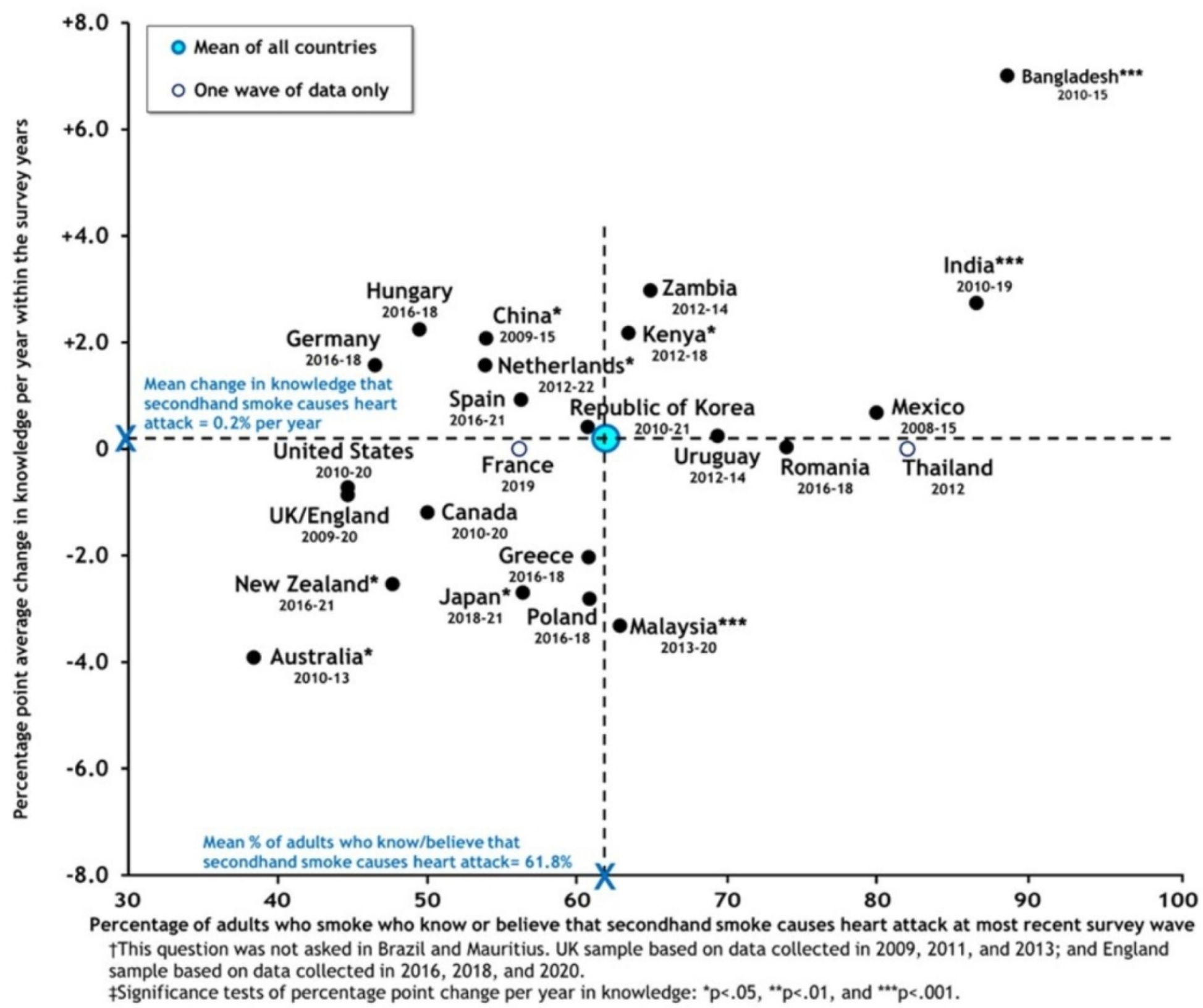

Figure 9. Percentage of adults who smoke who know or believe that secondhand smoke causes heart attack,† in 24 ITC countries,‡ 2008–2022.

123x113mm (300 x 300 DPI)

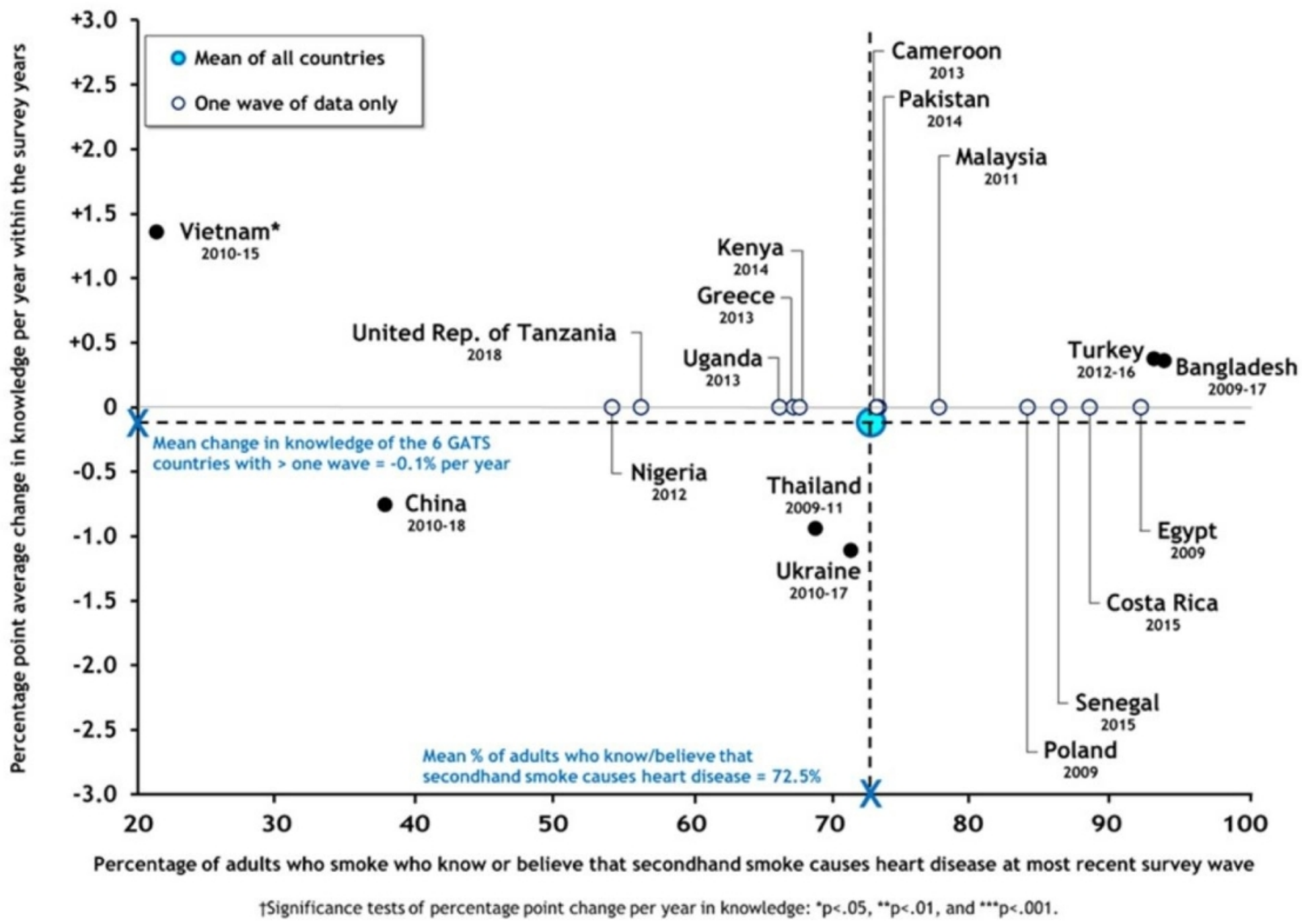

Figure 10. Percentage of adults who smoke who know or believe that secondhand smoke causes heart disease, in 18 GATS countries,† 2009–2018.

123x94mm (300 x 300 DPI)

Supplementary Appendix

Supplementary table 1. International Tobacco Control Policy Evaluation Project (ITC) Survey methods in 26 countries included in analysis, 2002-2022.

Country	Wave	Year	Data Collection Mode	Sample Representativeness	Sampling Design	Adults who Smoke† (N)	Replenishment Response Rate	Replenishment Cooperation Rate
Australia	1	2002	Telephone	National (telephone: random digit dialing)	Stratified random single stage	2194	45.8%	78.8%
	2	2003				1893	36.7%	79.3%
	3	2004				1757	44.2%	79.2%
	4	2005-2006				1602	39.5%	79.9%
	5	2006-2007				1656	45.3%	84.9%
	6	2007-2008				1604	29.1%	79.6%
	7	2008-2009	Telephone, Web	National (telephone; random digit dialing), Probability web panel	Stratified random single stage	1182	30.2%	80.7%
	8	2010-2011				893	28.0%	81.2%
	8.5	2011-2012				848	17.5%	96.6%
	9	2013				792	13.2%	98.3%
	V1	2016	Web	National probability web panel	Stratified single stage	1304	32.7%	93.1%
	V2	2018				1244	22.2%	95.1%
	V3	2020				1219	35.5%	93.4%
Bangladesh	1	2009	Face-to-Face	National	Stratified multi-stage	3041	> 90% household enumeration rate	81.1% individual response rate
	2	2010				2931	No new enumeration	Not computed (a)
	3	2011-2012				2493	> 90% household enumeration rate	Not reported
	4	2014-2015				1880	No new enumeration	Not computed (a)
Brazil	1	2009	Telephone	3 cities: Rio de Janeiro, São Paulo, Porto Alegre	Stratified single stage	1215	10.5%	90.7%
	2	2012-2013				1097	21.6%	98.9%
	3	2016-2017				1216	35.5%	60.4%
Canada	1	2002	Telephone	National (telephone: random digit dialing)	Stratified single stage	2070	49.5%	82.3%
	2	2003				1872	35.3%	86.6%
	3	2004				1735	50.0%	78.8%
	4	2005-2006				1590	29.4%	79.4%
	5	2006-2007				1514	27.3%	75.1%
	6	2007-2008				1413	26.7%	79.9%

For peer review only - http://bmjopen.bmj.com/site/about/guidelines.xhtml

Country	Wave	Year	Data Collection Mode	Sample Representativeness	Sampling Design	Adults who Smoke† (N)	Replenishment Response Rate	Replenishment Cooperation Rate
	7	2008-2009	Telephone, Web	National (telephone: random digit dialing), National probability web panel	Stratified single stage	1185	29.0%	81.1%
	8	2010-2011				904	29.9%	71.4%
	9	2013-2014				720	13.8%	66.5%
	V1	2016	Web	National probability web panel	Stratified single stage	2820	19.1%	91.1%
	V2	2018				2822	25.6%	94.8%
	V3	2020				2843	14.6%	93.2%
China	1	2006	Face-to-Face	6 cities: Beijing, Changsha, Guangzhou, Shanghai, Shenyang, Yinchuan	Stratified multi-stage	4732	Beijing:50.0%<br>Changsha:50.0%<br>Guangzhou:66.0%<br>Shanghai:61.3%<br>Shenyang:50.0%<br>Yinchuan:39.4%	Not computed (c)
	2	2007-2008				4623	Not computed (a)	Not computed (a)
	3	2009		7 cities: Beijing, Changsha, Guangzhou, Shanghai, Shenyang, Yinchuan, Kunming		5210	Beijing:Not computed (c)<br>Changsha:Not computed (c)<br>Guangzhou:85.0%<br>Shanghai:Not computed (c)<br>Shenyang:Not computed (c)<br>Yinchuan:Not computed (c)<br>Kunming:23.0%	Not computed (c)
	4	2010-2011				5077	Beijing:92.2%<br>Changsha:Not computed (c)<br>Guangzhou:85.0%<br>Shanghai:Not computed (c)<br>Shenyang:Not computed (c)<br>Yinchuan:Not computed (c)<br>Kunming:Not computed (c)	Not computed (c)
	5	2013-2015		5 cities: Beijing, Shanghai, Guangzhou, Shenyang, Kunming<br>5 rural areas: Xining, Tongren, Huzhou, Changzhi, Yichun		7387	Not computed (c)	Not computed (c)

For peer review only - http://bmjopen.bmj.com/site/about/guidelines.xhtml

Country	Wave	Year	Data Collection Mode	Sample Representativeness	Sampling Design	Adults who Smoke† (N)	Replenishment Response Rate	Replenishment Cooperation Rate
England	V1	2016	Web	Customized web panel	Stratified single stage	3536	15.2%	92.6%
	V2	2018				4149	44.1%	82.3%
	V3	2020				3233	13.7%	81.2%
France	1	2006-2007	Telephone	National (telephone: random digit dialing)	Stratified single stage	1735	36.3%	75.3%
	2	2008				1540	36.1%	80.5%
	3	2012				1420	21.3%	78.8%
	A1	2019	Web	National: customized web panel	Stratified single stage	1679	9.7%	92.5%
Germany	1	2007	Telephone	National	Stratified single stage	1515	26.1%	94.9%
	2	2009				912	Not computed (a)	Not computed (a)
	3	2011				515	Not computed (a)	Not computed (a)
	E1	2016	Face-to-face		Stratified multi-stage	1003	70.4%	83.4%
	E2	2018				946	68.7%	80.6%
Greece	E1	2016	Face-to-face	National	Stratified multi-stage	1000	89.6%	91.7%
	E2	2018				949	92.6%	93.3%
Hungary	E1	2016	Face-to-face	National	Stratified multi-stage	1000	92.8%	94.0%
	E2	2018				949	90.4%	91.6%
India**	1	2010-2011	Face-to-face	4 states: Bihar, Maharashtra, Madhya Pradesh, West Bengal	Stratified multi-stage	2042	Bihar:97.1%<br>Maharashtra:93.2%<br>Madhya Pradesh:93.0%<br>West Bengal:87.1%	Bihar:98.8%<br>Maharashtra:96.0%<br>Madhya Pradesh: 94.1%<br>West Bengal:93.0%
	2	2012-2013				1784	Bihar:97.7%<br>Maharashtra:82.3%<br>Madhya Pradesh:97.5%<br>West Bengal:82.3%	Bihar:100.0%<br>Maharashtra:90.9%<br>Madhya Pradesh:99.5%<br>West Bengal:90.7%
	3	2018-2019				1472	Bihar:65.1%<br>Maharashtra:81.5%<br>Madhya Pradesh:82.2%<br>West Bengal:75.0%	Bihar:100.0%<br>Maharashtra:93.8%<br>Madhya Pradesh: 99.5%<br>West Bengal: 100.0%
Japan	1	2018	Web	National probability web panel	Stratified single stage	3837	46.7%	96.8%
	2	2019				2689	27.2%	96.9%
	3	2020				2793	23.1%	94.8%
	4	2021				2649	27.4%	94.5%

For peer review only - http://bmjopen.bmj.com/site/about/guidelines.xhtml

Country	Wave	Year	Data Collection Mode	Sample Representativeness	Sampling Design	Adults who Smoke† (N)	Replenishment Response Rate	Replenishment Cooperation Rate
Kenya	1	2012	Face-to-face	National	Stratified multi-stage	1049	82.3%	Not computed (c)
	2	2018				833	Not computed (c)	Not computed (c)
Malaysia	1	2005	Face-to-face	6 states: Kedah, Selangor, Johore, Terengganu, Sabah, Sarawak	Stratified multi-stage	1974	Not computed (c)	Not computed (c)
	2	2006-2007				1478	Not computed (c)	Not computed (c)
	3	2008	Face-to-face, Telephone	7 states: Kedah, Penang, Selangor, Johore, Terengganu, Sabah, Sarawak	Stratified multi-stage	1809	Not computed (c)	Not computed (c)
	4	2009				1872	Not computed (c)	Not computed (c)
	5	2011-2012	Telephone	7 states: Kedah, Penang, Selangor, Johore, Terengganu, Sabah, Sarawak	Stratified multi-stage and single stage	1764	35.6%	55.9%
	6	2013-2014				1694	46.1%	85.5%
	S1	2020	Web	National population having internet access	Stratified single stage	1047	11.3%	Not computed (c)
Mauritius	1	2009	Face-to-face	National	Stratified multi-stage	598	80.7%	89.8%
	2	2010				553	96.9%	100.0%
	3	2011				535	96.0%	100.0%
Mexico	1	2006	Face-to-face	4 cities: Ciudad Juárez, Guadalajara, Ciudad de México, Tijuana	Stratified multi-stage	1079	Not computed (c)	88.7%
	2	2007		4 cities: Ciudad Juárez, Guadalajara, Ciudad de México, Tijuana		941	47.2%	Not computed (c)
	3	2008		7 cities: Ciudad Juárez, Guadalajara, Ciudad de México, Tijuana, Monterrey, Puebla, Mérida		1861	41.9%	76.1%
	4	2010		7 cities: Guadalajara, Ciudad de México, Tijuana, Leon, Monterrey, Puebla, Mérida		1853	41.5%	87.4%
	5	2011		7 cities: Guadalajara, Ciudad de México, Tijuana, Leon, Monterrey, Puebla, Mérida		1764	47.2%	Not computed (c)
	6	2012		7 cities: Guadalajara, Ciudad de México, Tijuana, Leon, Monterrey, Puebla, Mérida		1747	Not computed (c)	93.8%
	7	2014-2015		3 cities: Ciudad de México, Guadalajara, Monterrey		778	Not computed (c)	Not computed (c)

For peer review only - http://bmjopen.bmj.com/site/about/guidelines.xhtml

Country	Wave	Year	Data Collection Mode	Sample Representativeness	Sampling Design	Adults who Smoke† (N)	Replenishment Response Rate	Replenishment Cooperation Rate
Netherlands	1	2008	Telephone, Web	National (telephone: random digit dialing), Probability web panel	Stratified single stage	2072	4.2%	78.1%
	2		Web				83.0%*	Not computed (c)
	3		Telephone, Web				80.0%*	Not computed (c)
	4		Web				70.0%*	Not computed (c)
	5	2011	Web	National probability web panel (TNS NIPObase)	Stratified single stage	1672	65.0% *	Not computed (c)
	6	2012				1551	79.0% *	Not computed (c)
	7	2013				1480	79.0% *	Not computed (c)
	8	2014				1516	70.0% *	Not computed (c)
	9	2015				1217	66.0% *	Not computed (c)
	10	2016				1213	67.0% *	Not computed (c)
	11	2017				1199	69.0% *	Not computed (c)
	D1	2020	Web	National probability web panel (TNS NIPObase)	Stratified single stage	2067	57.6%	96.1%
	D2	2020				1752	58.3%	96.2%
	D3	2021				1721	54.0%	93.9%
	D4	2022				1734	55.4%	95.2%
New Zealand	1	2007-2008	Telephone	National	Sampled from New Zealand Health Survey national sample	1236	32.6%	56.4%
	2	2008-2009				761	Not computed (a)	Not computed (a)
	L1	2016-2017				910	27.6%	53.3%
	L2	2018				726	19.7%	Not computed (c)
	L3	2020-2021	Web	National, customized web panel, targeted social media	Sampled from New Zealand Health Survey national sample, web panel, social media	992	Not computed (b)	62.5%
Poland	1	2016	Face-to-face	National	Stratified multi-stage	1006	79.5%	81.6%
	2	2018				948	86.5%	87.6%
Republic of Korea	1	2005	Telephone	National	Stratified single stage	995	14.5%	71.2%
	2	2008				1737	2.4%	79.4%
	3	2010				1560	8.8%	95.3%
	R1	2016				2000	11.6%	99.8%
	A1	2020	Web	Customized web panel	Stratified single stage	3840	15.2%	97.4%
	A2	2021				3641	16.4%	97.0%

For peer review only

For peer review only - http://bmjopen.bmj.com/site/about/guidelines.xhtml

Country	Wave	Year	Data Collection Mode	Sample Representativeness	Sampling Design	Adults who Smoke† (N)	Replenishment Response Rate	Replenishment Cooperation Rate
Romania	1	2016	Face-to-face	National	Stratified multi-stage	1001	80.0%	81.2%
	2	2018				918	83.4%	84.8%
Spain	1	2016	Face-to-face	National	Stratified multi-stage	1001	81.1%	82.0%
	2	2018				886	83.1%	84.0%
	2.5	2021				864	80.8	96.2
Thailand	1	2005	Face-to-face	4 regions: Northern Region, North-Eastern Region, Central Plain Region, Southern Region	Stratified multi-stage	2000	Not computed (c)	Household cooperation rate 93.6%
	2	2006				1866	Not computed (c)	Not computed (c)
	3	2008				2163	Not computed (c)	Not computed (c)
	4	2009				1907	Not computed (c)	Not computed (c)
	5	2011				1687	Not computed (c)	Not computed (c)
	6	2012				1670	Not computed (c)	Not computed (c)
United States	1	2002	Telephone	National (telephone: random digit dialing)	Stratified single stage	2065	25.6%	83.2%
	2	2003				1837	Not computed (c)	88.6%
	3	2004				1846	34.9%	74.1%
	4	2005-2006				1698	23.4%	74.9%
	5	2006-2007				1668	20.7%	71.9%
	6	2007-2008				1570	22.3%	73.9%
	7	2008-2009	Telephone, Web	National (telephone: random digit dialing), probability web panel	Stratified single stage	1331	29.7%	90.8%
	8	2010-2011				1456	31.6%	90.1%
	9	2013-2015				1456	58.6% (web panel part)	
	V1	2016	Web	National probability web panel	Stratified single stage	2197	60.1%	80.4%
	V2	2018				2066	37.7%	75.2%
	V3	2020				1817	49.3%	86.7%
United Kingdom	1	2002	Telephone	National (telephone: random digit dialing)	Stratified single stage	2306	37.8%	78.7%
	2	2003				1866	25.8%	73.9%

For peer review only

Country	Wave	Year	Data Collection Mode	Sample Representativeness	Sampling Design	Adults who Smoke† (N)	Replenishment Response Rate	Replenishment Cooperation Rate
	3	2004				1748	41.6%	71.6%
	4	2006				1631	21.4%	78.7%
	5	2007				1567	12.9%	87.3%
	6	2008				1492	15.4%	74.6%
	7	2009	Telephone, Web	National (telephone: random digit dialing), National probability web panel	Stratified single stage	1309	32.6%	82.1%
	8	2011				812	Not computed (c)	Not computed (c)
	9	2013				926	Not computed (c)	Not computed (c)
Uruguay	1	2006	Face-to-face	Montevideo City	Stratified multi-stage	887	61.0%	72.6%
	2	2008-2009		5 cities: Montevideo, Salto, Maldonado, Durazno, Rivera		1294	68.7%	78.2%
	3	2010-2011				1224	63.2%	72.4%
	4	2012				1200	Not computed (c)	Not computed (c)
	5	2014				1181	Not computed (c)	Not computed (c)
Zambia	1	2012	Face-to-face	National	Stratified multi-stage	1188	61.1%	93.4%
	2	2014				1045	Not computed (c)	Not computed (c)

† Adults who smoke are defined as individuals (aged 18 years and older) who currently smoke cigarettes at least monthly.

Not computed (a): Little or no replenishment conducted

Not computed (b): Invitation data not available from survey firm

Not computed (c): Incomplete information available from field staff

For peer review only - http://bmjopen.bmj.com/site/about/guidelines.xhtml

Supplementary table 2. Global Adult Tobacco Survey (GATS) methods in 32 countries included in analysis, 2008–2020.

Country	Wave	Year	Data Collection Mode	Sample Representativeness	Sampling Design	Adults who Smoke† (N)	Response Rate
Argentina	1	2012	Face-to-face interview	National	Stratified multi-stage	1610	74.3%
Bangladesh	1	2009	Face-to-face interview	National	Stratified multi-stage	2207	93.6%
	2	2017	Face-to-face interview	National	Stratified multi-stage	2470	90.8%
Botswana	1	2017	Face-to-face interview	National	Stratified multi-stage	628	80.0%
Brazil	1	2008	Face-to-face interview	National	Stratified multi-stage	6911	94.0%
Cameroon	1	2013	Face-to-face interview	National	Stratified multi-stage	399	94.1%
China	1	2010	Face-to-face interview	National	Stratified multi-stage	4002	96.0%
	2	2018	Face-to-face interview	National	Stratified multi-stage	4953	91.5%
Costa Rica	1	2015	Face-to-face interview	National	Stratified multi-stage	650	89.2%
Egypt	1	2009	Face-to-face interview	National	Stratified multi-stage	4124	97.2%
Ethiopia	1	2016	Face-to-face interview	National	Stratified multi-stage	695	93.4%
Greece	1	2013	Face-to-face interview	National	Stratified multi-stage	1662	69.6%
India	1	2009	Face-to-face interview	National	Stratified multi-stage	11538	91.8%
	2	2017	Face-to-face interview	National	Stratified multi-stage	9434	92.9%
Indonesia	1	2011	Face-to-face interview	National	Stratified multi-stage	2783	94.3%
Kazakhstan	1	2014	Face-to-face interview	National	Stratified multi-stage	1020	96.7%
Kenya	1	2014	Face-to-face interview	National	Stratified multi-stage	447	87.1%
Malaysia	1	2011	Face-to-face interview	National	Stratified multi-stage	965	85.3%
Mexico	1	2009	Face-to-face interview	National	Stratified multi-stage	1752	82.5%
	2	2015	Face-to-face interview	National	Stratified multi-stage	1809	82.7%
Nigeria	1	2012	Face-to-face interview	National	Stratified multi-stage	422	89.1%

For peer review only - http://bmjopen.bmj.com/site/about/guidelines.xhtml

Country	Wave	Year	Data Collection Mode	Sample Representativeness	Sampling Design	Adults who Smoke† (N)	Response Rate
Pakistan	1	2014	Face-to-face interview	National	Stratified multi-stage	953	81.0%
Panama	1	2013	Face-to-face interview	National	Stratified multi-stage	973	88.4%
Philippines	1	2009	Face-to-face interview	National	Stratified multi-stage	2698	94.7%
	2	2015	Face-to-face interview	National	Stratified multi-stage	2751	92.1%
Poland	1	2009	Face-to-face interview	National	Stratified multi-stage	2400	65.1%
Qatar	1	2013	Face-to-face interview	National	Stratified multi-stage	1070	98.5%
Romania	1	2011	Face-to-face interview	National	Stratified multi-stage	1044	88.5%
	2	2018	Face-to-face interview	National	Stratified multi-stage	1236	88.0%
Russian Federation	1	2009	Face-to-face interview	National	Stratified multi-stage	4747	97.7%
	2	2016	Face-to-face interview	National	Stratified multi-stage	3312	98.2%
Senegal	1	2015	Face-to-face interview	National	Stratified multi-stage	267	97.0%
Thailand	1	2009	Face-to-face interview	National	Stratified multi-stage	4860	94.2%
	2	2011	Face-to-face interview	National	Stratified multi-stage	4229	96.3%
Turkey	1	2008	Face-to-face interview	National	Stratified multi-stage	2666	90.9%
	2	2012	Face-to-face interview	National	Stratified multi-stage	2376	90.1%
	3	2016	Face-to-face interview	National	Stratified multi-stage	2792	82.2%
Uganda	1	2013	Face-to-face interview	National	Stratified multi-stage	568	86.6%
Ukraine	1	2010	Face-to-face interview	National	Stratified multi-stage	2361	76.1%
	2	2017	Face-to-face interview	National	Stratified multi-stage	1776	64.4%
United Republic of Tanzania	1	2018	Face-to-face interview	National	Stratified multi-stage	375	91.7%
Uruguay	1	2009	Face-to-face interview	National	Stratified multi-stage	1362	95.2%
	2	2017	Face-to-face interview	National	Stratified multi-stage	1043	89.4%

For peer review only - http://bmjopen.bmj.com/site/about/guidelines.xhtml

Country	Wave	Year	Data Collection Mode	Sample Representativeness	Sampling Design	Adults who Smoke[†] (N)	Response Rate
Viet Nam	1	2010	Face-to-face interview	National	Stratified multi-stage	2240	92.7%
	2	2015	Face-to-face interview	National	Stratified multi-stage	1957	95.8%

[†] Defined adults who smoke as persons (aged 18+ years) who currently smoked any combustible tobacco products (bidis, kreteks, cheroots, manufactured cigarettes, hand-rolled cigarettes, cigars, cigarillos, pipes, waterpipe with tobacco).

For peer review only

For peer review only - http://bmjopen.bmj.com/site/about/guidelines.xhtml

Supplementary table 3. International Tobacco Control Policy Evaluation Project (ITC) countries (2002–2022) and Global Adult Tobacco Survey (GATS) countries (2008–2020).

	ITC	GATS
Argentina	NA	X
Australia	X	NA
Bangladesh	X	X
Botswana	NA	X
Brazil	X	X
Cameroon	NA	X
Canada	X	NA
China	X	X
Costa Rica	NA	X
Egypt	NA	X
England	X	NA
Ethiopia	NA	X
France	X	NA
Germany	X	NA
Greece	X	X
Hungary	X	NA
India	X	X
Indonesia	NA	X
Japan	X	NA
Kazakhstan	NA	X
Kenya	X	X
Malaysia	X	X
Mauritius	X	NA
Mexico	X	X
Netherlands	X	NA
New Zealand	X	NA
Nigeria	NA	X
Pakistan	NA	X
Panama	NA	X
Philippines	NA	X
Poland	X	X
Qatar	NA	X
Republic of Korea	X	NA
Romania	X	X
Russian Federation	NA	X
Senegal	NA	X
Spain	X	NA
Thailand	X	X
Turkey	NA	X
Uganda	NA	X
Ukraine	NA	X
United Republic of Tanzania	NA	X
United States	X	NA
Uruguay	X	X
Viet Nam	NA	X
Zambia	X	NA

X=country included in ITC Project Surveys and/or GATS
NA=country not included in ITC Project Surveys and/or GATS

For peer review only

Supplementary table 4. International Tobacco Control Policy Evaluation Project (ITC) Survey and Global Adult Tobacco Survey (GATS) measures of knowledge about the risks of smoking and secondhand smoke exposure for lung cancer, heart disease/heart attack, and stroke.

Health knowledge variable	ITC Project Survey Question and Response Options	GATS Question and Response Options
Smoking causes lung cancer[a]	Based on what you know or believe, does smoking cause lung cancer in smokers? (question asked in 25/26 ITC countries)<br>☐ Yes<br>☐ No<br>☐ Refused<br>☐ Don't know	Based on what you know or believe, does smoking tobacco cause lung cancer? (question asked in 32 GATS countries)<br>☐ Yes<br>☐ No<br>☐ Refused<br>☐ Don't know
Smoking causes heart disease[b,c]	Based on what you know or believe, does smoking cause heart disease in smokers? (question asked in 25/26 ITC countries)<br>☐ Yes<br>☐ No<br>☐ Refused<br>☐ Don't know	Based on what you know or believe, does smoking tobacco cause heart attack? (question asked in 32 GATS countries)<br>☐ Yes<br>☐ No<br>☐ Refused<br>☐ Don't know
Smoking causes stroke	Based on what you know or believe, does smoking cause stroke in smokers? (question asked in 25/26 ITC countries)<br>☐ Yes<br>☐ No<br>☐ Refused<br>☐ Don't know	Based on what you know or believe, does smoking tobacco cause stroke (blood clots in the brain)? (question asked in 32 GATS countries<br>☐ Yes<br>☐ No<br>☐ Refused<br>☐ Don't know
Secondhand smoke causes lung cancer[d]	Based on what you know or believe, does smoking cause lung cancer in non-smokers from secondhand smoke? (question asked in 26 ITC countries)<br>☐ Yes<br>☐ No<br>☐ Refused<br>☐ Don't know	Based on what you know or believe does breathing other people's smoke cause lung cancer? (question asked in 18/32 GATS countries)<br>☐ Yes<br>☐ No<br>☐ Refused<br>☐ Don't know
Secondhand smoke causes heart attack[e]	Based on what you know or believe, does secondhand smoke cause heart attack in non-smokers? (question asked in 22/26 ITC countries)<br>☐ Yes<br>☐ No<br>☐ Refused<br>☐ Don't know	Based on what you know or believe, does breathing in other people's smoke cause heart disease? (question asked in 18/32 GATS countries)<br>☐ Yes<br>☐ No<br>☐ Refused<br>☐ Don't know

[a] ITC Bangladesh Wave 1 (2009), Wave 2 (2010), Wave 3 (2011-12), Wave 4 (2014-15) Survey: Based on what you know or believe, does smoking cigarettes cause lung cancer in smokers?
ITC France Wave 1 (2006-07), Wave 2 (2008), and Wave 3 (2012) Survey: Based on what you know or believe, smoking can cause (Wave 3)/can smoking cause (Wave 1, Wave 2) lung cancer?
ITC Germany Wave 1 (2016) and Wave 2 (2018) Survey: Based on what you know or believe, does smoking cause lung cancer?
ITC India Wave 3 Survey: Based on what you know or believe, does smoking cigarettes cause lung cancer in smokers?
ITC Japan Wave 3 (2020) and Wave 4 (2021) Survey: Based on what you know or believe, does smoking cigarettes cause lung cancer in cigarette smokers?

For peer review only - http://bmjopen.bmj.com/site/about/guidelines.xhtml

ITC Republic of Korea Wave 1 (2005) and Wave 2 (2008) Survey: Based on what you know or believe, does smoking cigarettes cause lung cancer in cigarette smokers?
ITC Republic of Korea Wave 1 (2020) and Wave 2 (2021) Survey: Based on what you know or believe, does smoking cigarettes cause lung cancer in cigarette smokers?
ITC Netherlands Wave 5 (2011), Wave 6 (2012), Wave 7 (2013), Wave 8 (2014), Wave 9 (2015), Wave 10 (2016), Wave 11 (2017) Survey: Based on what you know or believe, does smoking cause lung cancer?
ITC Mauritius Wave 1 (2009) Survey: Based on what you know or believe, does smoking cigarettes cause lung cancer?
[b] ITC Japan Wave 1 (2018), Wave 2 (2019), and Wave 3 (2020) Survey: Based on what you know or believe, does smoking cigarettes cause myocardial infarction in cigarette smokers?
[c] ITC Bangladesh Wave 1 (2009) and Wave 2 (2010) Survey: Based on what you know or believe, does smoking cigarettes cause coronary heart disease/COPD?
[d] ITC Australia Wave 3 (2020) Survey: Based on what you know or believe, does secondhand smoke cause lung disease, including lung cancer, in non-smokers?
ITC Bangladesh Wave 1 (2009) Survey: Based on what you know or believe, does smoking cigarettes cause lung cancer in non-smokers from secondhand smoke?
ITC Canada Wave 3 (2020) Survey: Based on what you know or believe, does secondhand smoke cause lung disease, including lung cancer, in non-smokers?
ITC China Wave 1 (2006), Wave 2 (2007-08), Wave 3 (2009), Wave 4 (2010-11), Wave 5 (2013-15) Survey: Do you think smoking causes lung cancer in non-smokers from secondhand smoke?
ITC England Wave 3 (2020) Survey: Based on what you know or believe, does secondhand smoke cause lung disease, including lung cancer, in non-smokers?
ITC France Wave 1 (2006-07), Wave 2 (2008), Wave 3 (2012) Survey: Based on what you know or believe, can passive smoking cause lung cancer in non-smokers?
ITC India Wave 3 (2018-19) Survey: Based on what you know or believe, does passive cigarette smoking cause lung cancer in non-smokers?
ITC Japan Wave 1 (2018), Wave 2 (2019), Wave 3 (2020) Survey: Based on what you know or believe, does smoking cigarettes cause lung cancer in non-smokers from secondhand smoke?
ITC Japan Wave 4 (2021) Survey: Based on what you know or believe, does secondhand cigarette smoke cause lung cancer in non-smokers?
ITC Republic of Korea Wave 1 (2020) and Wave 2 (2021) Survey: Based on what you know or believe, does secondhand cigarette smoke cause lung cancer in non-smokers?
ITC Spain 2.5 (2021) Survey: Based on what you know or believe, does breathing secondhand smoke cause lung cancer in non-smokers?
ITC United States Wave 3 (2020) Survey: Based on what you know or believe, does secondhand smoke cause lung disease, including lung cancer, in non-smokers?
[e] ITC Bangladesh Wave 2 (2010): Based on what you know or believe, does passive smoking cause coronary heart disease/COPD in non-smokers?

For peer review only

For peer review only - http://bmjopen.bmj.com/site/about/guidelines.xhtml

Supplementary table 5. Proportions of adults who smoke and had knowledge about health risks of smoking at most recent survey for which data was available in ITC countries and GATS countries.[a]

	Smoking causes lung cancer		Smoking causes heart disease (ITC)/heart attack (GATS)		Smoking causes stroke		Secondhand smoke causes lung cancer		Secondhand smoke causes heart attack (ITC)/heart disease (GATS)	
**Country**	**ITC % [95% CI] (year)**	**GATS % [95% CI] (year)**	**ITC % [95% CI] (year)**	**GATS % [95% CI] (year)**	**ITC % [95% CI] (year)**	**GATS % [95% CI] (year)**	**ITC % [95% CI] (year)**	**GATS % [95% CI] (year)**	**ITC % [95% CI] (year)**	**GATS % [95% CI] (year)**
Argentina	NA	98.4 [97.1-99.1] (2012)	NA	92.1 [88.7-94.5] (2012)	NA	72.8 [64.7-79.6] (2012)	NA	NA	NA	NA
Australia	94.0 [92.6-95.1] (2003)	NA	91.4 [89.8-92.8] (2004)	NA	81.3 [77.8-84.4] (2020)	NA	73.1 [70.1-75.9] (2008-09)	NA	38.4 [34.3-42.6] (2013)	NA
Bangladesh	98.2 [96.9-98.9] (2014-15)	97.1 [95.8-98.0] (2017)	94.9 [91.3-97.0] (2014-15)	93.5 [91.9-94.8] (2017)	93.9 [90.3-96.3] (2014-15)	94.1 [92.4-95.5] (2017)	95.2 [93.0-96.7] (2014-15)	95.4 [94.1-96.4] (2017)	88.5 [82.4-92.7] (2014-15)	93.5 [91.9-94.8] (2017)
Botswana	NA	92.6 [89.5-94.8] (2017)	NA	81.0 [76.6-84.7] (2017)	NA	74.0 [68.9-78.4] (2017)	NA	NA	NA	NA
Brazil	96.2 [94.8-97.2] (2016-17)	97.5 [97.1-98.0] (2008)	93.7 [91.8-95.2] (2016-17)	88.6 [87.5-89.5] (2008)	86.8 [84.2-89.1] (2016-2017)	76.7 [75.3-78.1] (2008)	81.1 [78.3-83.7] (2016-17)	NA	NA	NA
Canada	94.0 [92.7-95.1] (2003)	NA	92.3 [91.1-93.4] (2020)	NA	85.7 [84.0-87.2] (2020)	NA	76.9 [74.9-78.8] (2020)	NA	49.9 [47.6-52.2] (2020)	NA
Cameroon	NA	89.9 [85.5-93.1] (2013)	NA	68.6 [61.2-75.1] (2013)	NA	61.3 [54.5-67.7] (2013)	NA	78.8 [73.3-83.4] (2013)	NA	72.9 [65.5-79.2] (2013)
China	81.1 [78.3-83.7] (2013-15)	78.4 [76.1-80.5] (2018)	57.7 [54.6-60.6] (2013-15)	46.4 [43.5-49.3] (2018)	37.3 [34.8-39.7] (2013-15)	37.4 [34.7-40.2] (2018)	72.7 [69.2-76.0] (2013-15)	61.6 [59.0-64.2] (2018)	53.8 [50.7-56.9] (2013-15)	37.7 [35.1-40.4] (2018)
Costa Rica	NA	96.9 [95.0-98.0] (2015)	NA	93.3 [91.0-95.1] (2015)	NA	72.0 [66.9-76.6] (2015)	NA	93.5 [90.8-95.5] (2015)	NA	88.2 [84.8-90.9] (2015)
Egypt	NA	97.4 [96.2-98.2] (2009)	NA	96.9 [96.0-97.6] (2009)	NA	89.8 [88.2-91.2] (2009)	NA	94.0 [92.7-95.1] (2009)	NA	91.8 [90.3-93.1] (2009)
England/ United Kingdom	91.5 [89.9-92.9] (2003)[b]	NA	86.0 [84.0-87.7] (2020)[c]	NA	78.5 [76.1-80.6] (2020)[c]	NA	66.7 [64.1-69.2] (2020)[c]	NA	44.6 [42.0-47.3] (2020)[c]	NA
Ethiopia	NA	67.1 [48.2-81.7] (2016)	NA	54.6 [39.2-69.1] (2016)	NA	29.6 [19.2-42.8] (2016)	NA	NA	NA	NA
France (2019)	93.8 [92.1-95.2] (2019)	NA	91.2 [89.4-92.8] (2019)	NA	83.4 [81.0-85.6] (2019)	NA	77.0 [74.2-79.6] (2019)	NA	56.1 [52.9-59.2] (2019)	NA
Germany	91.2 [85.6-94.7] (2018)	NA	80.6 [73.9-85.9] (2018)	NA	79.2 [72.9-84.3] (2018)	NA	58.4 [51.5-64.9] (2018)	NA	46.4 [39.6-53.4] (2018)	NA
Greece	96.0 [93.1-97.8] (2018)	95.1 [92.7-96.8] (2013)	95.1 [90.9-97.4] (2018)	88.1 [83.5-91.5] (2013)	79.2 [74.2-83.5] (2018)	69.3 [61.8-75.9] (2013)	64.7 [58.7-70.3] (2018)	68.1 [61.1-74.4] (2013)	60.7 [53.8-67.1] (2018)	66.9 [59.8-73.4] (2013)
Hungary	83.2 [78.3-87.2] (2018)	NA	70.2 [64.4-75.5] (2018)	NA	65.3 [59.7-70.6] (2018)	NA	60.3 [54.1-66.2] (2018)	NA	49.4 [43.5-55.3] (2018)	NA
India	91.9 [87.8-94.8] (2018-19)	91.9 [90.9-92.8] (2016)	86.1 [82.0-89.4] (2018-19)	75.3 [73.6-77.0] (2016)	75.0 [68.7-80.3] (2018-19)	63.1 [61.1-65.1] (2016)	88.3 [84.5-91.2] (2018-19)	NA	86.5 [82.1-89.9] (2018-19)	NA
Indonesia	NA	78.8 [75.4-81.9] (2011)	NA	78.0 [74.2-81.3] (2011)	NA	40.4 [35.6-45.3] (2011)	NA	NA	NA	NA
Japan	80.5 [77.9-82.8] (2021)	NA	65.5 [62.7-68.1] (2021)	NA	NA	NA	69.0 [66.7-71.2] (2021)	NA	56.3 [53.5-59.0] (2021)	NA
Kazakhstan	NA	78.9 [75.1-82.3] (2014)	NA	54.8 [50.4-59.0] (2014)	NA	55.2 [50.8-59.5] (2014)	NA	NA	NA	NA
Kenya	90.9 [87.9-93.3] (2018)	85.6 [80.6-89.4] (2014)	79.8 [75.7-83.4] (2018)	60.0 [53.7-66.0] (2014)	67.3 [62.1-72.2] (2018)	45.7 [38.4-53.1] (2014)	74.6 [69.4-79.2] (2018)	78.6 [72.4-83.7] (2014)	63.3 [57.7-68.6] (2018)	67.4 [60.7-73.5] (2014)
Malaysia	87.8 [84.7-90.3] (2020)	88.7 [85.1-91.5] (2011)	82.8 [79.8-85.4] (2020)	84.8 [80.9-88.1] (2011)	66.3 [62.5-70.0] (2020)	75.4 [70.9-79.4] (2011)	73.3 [69.6-76.7] (2020)	81.2 [77.3-84.6] (2011)	62.7 [58.7-66.6] (2020)	77.4 [73.0-81.2] (2011)

For peer review only

For peer review only - http://bmjopen.bmj.com/site/about/guidelines.xhtml

	Smoking causes lung cancer		Smoking causes heart disease (ITC)/heart attack (GATS)		Smoking causes stroke		Secondhand smoke causes lung cancer		Secondhand smoke causes heart attack (ITC)/heart disease (GATS)	
**Country**	**ITC % [95% CI] (year)**	**GATS % [95% CI] (year)**	**ITC % [95% CI] (year)**	**GATS % [95% CI] (year)**	**ITC % [95% CI] (year)**	**GATS % [95% CI] (year)**	**ITC % [95% CI] (year)**	**GATS % [95% CI] (year)**	**ITC % [95% CI] (year)**	**GATS % [95% CI] (year)**
Mauritius	91.8 [87.1-94.9] (2011)	NA	85.6 [80.9-89.3] (2011)	NA	76.1 [70.4-81.0] (2011)	NA	85.7 [80.4-89.8] (2011)	NA	NA	NA
Mexico	96.9 [94.4-98.3] (2014-15)	98.0 [97.1-98.6] (2015)	81.8 [76.7-86.0] (2014-15)	87.5 [85.3-89.4] (2015)	75.6 [70.8-79.8] (2014-15)	68.1 [65.2-70.8] (2015)	91.7 [88.1-94.3] (2014-15)	NA	79.9 [75.0-84.0] (2014-15)	NA
Netherlands	92.2 [90.7-93.5] (2022)	NA	86.3 [84.3-88.0] (2022)	NA	71.0 [68.5-73.3] (2022)	NA	64.7 [62.2-67.2] (2022)	NA	53.8 [51.2-56.4] (2022)	NA
New Zealand	NA	NA	NA	NA	76.9 [73.0-80.4] (2020-21)	NA	62.3 [58.1-66.3] (2020-21)	NA	47.6 [43.5-51.8] (2020-21)	NA
Nigeria	NA	55.6 [48.4-62.6] (2012)	NA	64.3 [57.3-70.8] (2012)	NA	32.2 [25.5-39.8] (2012)	NA	41.9 [35.1-49.0] (2012)	NA	54.0 [46.6-61.2] (2012)
Pakistan	NA	83.6 [79.8-86.8] (2014)	NA	86.9 [83.7-89.6] (2014)	NA	54.6 [49.1-60.1] (2014)	NA	69.3 [64.6-73.5] (2014)	NA	73.1 [68.6-77.1] (2014)
Panama	NA	96.0 [94.1-97.3] (2013)	NA	84.0 [78.2-88.5] (2013)	NA	76.8 [70.0-82.4] (2013)	NA	NA	NA	NA
Philippines	NA	93.6 [92.2-94.8] (2015)	NA	82.4 [79.9-84.6] (2015)	NA	75.3 [72.3-78.2] (2015)	NA	NA	NA	NA
Poland	85.9 [81.1-89.7] (2018)	94.9 [93.6-95.9] (2009)	79.7 [74.2-84.2] (2018)	79.8 [77.5-82.0] (2009)	64.7 [59.4-69.7] (2018)	58.6 [55.7-61.5] (2009)	69.2 [61.4-76.1] (2018)	91.4 [89.7-92.9] (2009)	60.7 [53.1-67.9] (2018)	83.7 [81.4-85.8] (2009)
Qatar	NA	90.0 [86.4-92.7] (2013)	NA	88.7 [85.3-91.4] (2013)	NA	70.0 [64.9-74.6] (2013)	NA	NA	NA	NA
Republic of Korea	90.0 [87.9-91.8] (2021)	NA	66.4 [61.6-70.8] (2021)	NA	72.2 [67.6-76.4] (2021)	NA	75.8 [70.3-80.6] (2021)	NA	60.6 [55.9-65.1] (2021)	NA
Romania	87.2 [83.1-90.5] (2018)	87.5 [84.3-90.1] (2018)	87.7 [83.5-90.9] (2018)	72.4 [68.1-76.3] (2018)	77.5 [72.1-82.1] (2018)	73.2 [68.7-77.3] (2018)	78.6 [73.3-83.0] (2018)	NA	73.9 [68.5-78.6] (2018)	NA
Russian Federation	NA	87.8 [85.8-89.6] (2016)	NA	74.1 [71.2-76.8] (2016)	NA	72.0 [68.9-74.9] (2016)	NA	NA	NA	NA
Senegal	NA	88.4 [83.0-92.3] (2015)	NA	71.4 [64.4-77.5] (2015)	NA	66.1 [58.6-72.8] (2015)	NA	83.8 [77.9-88.4] (2015)	NA	85.9 [80.4-90.1] (2015)
Spain	94.8 [92.2-96.6] (2021)	NA	88.5 [85.1-91.2] (2021)	NA	65.6 [58.6-72.1] (2021)	NA	69.3 [63.7-74.4] (2021)	NA	56.2 [49.6-62.5] (2021)	NA
Thailand	94.1 [92.9-95.1] (2008)	96.3 [95.0-97.2] (2011)	71.6 [67.4-75.5] (2012)	74.9 [72.3-77.3] (2011)	83.7 [81.4-85.7] (2012)	78.7 [76.1-81.0] (2011)	96.8 [95.2-97.9] (2012)	96.4 [95.5-97.1] (2011)	81.9 [77.8-85.4] (2012)	68.5 [65.8-71.2] (2011)
Turkey	NA	95.3 [94.0-96.3] (2016)	NA	95.3 [94.1-96.3] (2016)	NA	87.7 [85.7-89.4] (2016)	NA	93.0 [91.5-94.3] (2016)	NA	92.8 [91.3-94.0] (2016)
Uganda	NA	82.1 [77.9-85.7] (2013)	NA	68.1 [62.7-73.0] (2013)	NA	36.4 [30.5-42.8] (2013)	NA	72.1 [66.6-77.1] (2013)	NA	66.0 [60.1-71.4] (2013)
Ukraine	NA	86.9 [83.7-89.5] (2017)	NA	77.9 [74.6-80.9] (2017)	NA	76.8 [73.5-79.7] (2017)	NA	69.4 [65.9-72.6] (2017)	NA	71.1 [67.8-74.1] (2017)
Uruguay	95.9 [93.9-97.2] (2014)	95.9 [94.0-97.2] (2017)	89.3 [86.2-91.8] (2014)	91.4 [89.2-93.2] (2017)	65.2 [61.6-68.7] (2014)	76.8 [73.9-79.6] (2017)	80.7 [77.4-83.6] (2014)	NA	69.3 [65.4-72.8] (2014)	NA
United Republic of Tanzania	NA	81.0 [76.0-85.1] (2018)	NA	57.6 [51.4-63.6] (2018)	NA	39.8 [33.5-46.4] (2018)	NA	67.0 [61.0-72.4] (2018)	NA	56.0 [50.0-61.9] (2018)
United States	93.0 [91.6-94.1] (2003)	NA	86.9 [84.7-88.9] (2020)	NA	77.5 [74.8-80.0] (2020)	NA	68.4 [65.5-71.2] (2020)	NA	44.6 [41.5-47.8] (2020)	NA
Viet Nam	NA	94.7 [93.1-96.0] (2015)	NA	68.6 [65.6-71.4] (2015)	NA	68.3 [65.5-71.0] (2015)	NA	85.1 [82.7-87.3] (2015)	NA	21.3 [19.0-23.9] (2015)

For peer review only - http://bmjopen.bmj.com/site/about/guidelines.xhtml

	Smoking causes lung cancer		Smoking causes heart disease (ITC)/heart attack (GATS)		Smoking causes stroke		Secondhand smoke causes lung cancer		Secondhand smoke causes heart attack (ITC)/heart disease (GATS)	
**Country**	**ITC % [95% CI] (year)**	**GATS % [95% CI] (year)**	**ITC % [95% CI] (year)**	**GATS % [95% CI] (year)**	**ITC % [95% CI] (year)**	**GATS % [95% CI] (year)**	**ITC % [95% CI] (year)**	**GATS % [95% CI] (year)**	**ITC % [95% CI] (year)**	**GATS % [95% CI] (year)**
Zambia	78.1 [72.6-82.8] (2014)	NA	73.0 [69.8-76.0] (2014)	NA	47.4 [43.4-51.4] (2014)	NA	68.2 [63.3-72.8] (2014)	NA	64.8 [60.2-69.1] (2014)	NA
**Overall [a]**	**92.2**	**91.9**	**84.7**	**80.0**	**75.0**	**66.1**	**77.3**	**82.1**	**61.8**	**72.5**

Abbreviations: CI, confidence interval; NA=no data collected as country was not included in ITC Project Surveys and/or GATS.
[a] Point estimates of the mean knowledge for each health effect across all ITC countries and all GATS countries were calculated using a multi-level model (two levels: individual and country) with random intercepts at the country level, with roughly equal weights for each country. These point estimates cannot be used to make inferences to the global population since the countries included in our analysis were not randomly selected from all countries in the world. As such, we report the estimated all-country means of knowledge for each health effect without the associated confidence intervals, which would refer to a hypothetical superpopulation of countries. The all-country mean estimates in this analysis therefore only serve as the central point of selected countries.
[b] Data collected for United Kingdom sample in 2003.
[c] Data collected for England sample in 2020.

For peer review only - http://bmjopen.bmj.com/site/about/guidelines.xhtml

Supplementary table 6. Overall knowledge at most recent survey and average changes in knowledge per year (percentage points (PP)/year) of five diseases caused by smoking and secondhand smoke, among adults who smoke in ITC and GATS countries.

	**Data Source**			
	**ITC**		**GATS**	
	**Overall knowledge at most recent survey (%)**	**Average change in knowledge per year**	**Overall knowledge at most recent survey (%)**	**Average change in knowledge per year**
Smoking causes lung cancer	92.2	– 0.7 PP	91.9	– 0.2 PP
Smoking causes heart disease (ITC) / heart attack (GATS)	84.7	– 0.3 PP	80.0	+ 0.5 PP
Smoking causes stroke	75.0	+ 1.6 PP	66.1	+ 0.5 PP
Secondhand smoke causes lung cancer	77.3	– 0.1 PP	82.1	0.0 PP
Secondhand smoke causes heart attack (ITC) / heart disease (GATS)	61.8	+ 0.2 PP	72.5	– 0.1 PP
Simple average of estimated net change in knowledge across the five health harms		+0.14 PP		+0.14 PP

Note: This table presents a descriptive summary of changes in knowledge across the five health effects in ITC and GATS countries that are not derived from statistical models. Testing for statistically significant changes in knowledge cannot be assessed.

Abbreviation: PP=percentage points

For peer review only - http://bmjopen.bmj.com/site/about/guidelines.xhtml

Supplementary table 7. Changes in knowledge that smoking causes lung cancer, heart disease, and stroke among adults who smoke in ITC countries.

ITC Country	Smoking causes lung cancer				Smoking causes heart disease				Smoking causes stroke			
	**First survey % [95% CI] (year)**	**Last survey % [95% CI] (year)**	**Average PP change per year**	**p-value**	**First survey % [95% CI] (year)**	**Last survey % [95% CI] (year)**	**Average PP change per year**	**p-value**	**First survey % [95% CI] (year)**	**Last survey % [95% CI] (year)**	**Average PP change per year**	**p-value**
Australia	94.1 [92.9,95.1] (2002)	93.3 [91.8,94.6] (2003)	-0.8	.890	89.2 [87.8,90.6] (2002)	90.5 [88.7,91.9] (2004)	+0.6	.080	80.9 [79.0,82.6] (2002)	80.8 [77.3,83.9] (2020)	0.0	.694
Bangladesh	94.5 [92.3,96.1] (2009)	98.1 [96.6,98.9] (2014-15)	+0.6	.000	88.1 [84.3,91.1] (2009)	94.5 [90.9,96.7] (2014-15)	+1.2	.000	93.1 [90.4,95.1] (2009)	93.6 [89.8,96.1] (2014-15)	+0.1	.100
Brazil	96.6 [95.3,97.6] (2009)	96.0 [94.5,97.1] (2016-17)	-0.1	.700	95.4 [94.0,96.5] (2009)	93.4 [91.5,94.9] (2016-17)	-0.3	.420	82.6 [80.1,84.9] (2009)	86.6 [83.9,88.8] (2016-17)	+0.5	.210
Canada	94.9 [93.8,95.8] (2002)	93.4 [92.1,94.6] (2003)	-1.5	.590	91.2 [89.8,92.4] (2002)	92.1 [90.9,93.2] (2020)	+0.1	.810	82.6 [80.7,84.3] (2002)	85.6 [83.9,87.1] (2020)	+0.2	.870
China	71.5 [69.2,73.7] (2006)	80.9 [77.8,83.7] (2013-15)	+1.2	.007	38.0 [35.5,40.5] (2006)	57.0 [53.8,60.1] (2013-15)	+2.2	.010	16.2 [14.5,18.1] (2006)	36.5 [34.1,39.0] (2013-15)	+2.5	.000
England/UK[a]	94.0 [92.9,95.0] (2002)	90.5 [88.8,92.1] (2003)	-3.5	.090	90.1 [88.8,91.4] (2002)	85.1 [83.1,87.0] (2020)	-0.3	.590	70.5 [68.3,72.5] (2002)	78.1 [75.7,80.3] (2020)	+0.4	.830
France	99.1 [98.4,99.5] (2006-07)	93.1 [91.3,94.6] (2019)	-0.5	.000	97.1 [96.0,97.9] (2006-07)	90.3 [88.4,91.9] (2019)	-0.5	.003	87.6 [85.7,89.3] (2006-07)	83.0 [80.5,85.1] (2019)	-0.4	.310
Germany	97.8 [96.8,98.5] (2007)	91.5 [88.1,94.0] (2018)	-0.6	.000	95.6 [94.3,96.6] (2007)	80.1 [76.1,83.6] (2018)	-1.4	.000	88.9 [86.8,90.7] (2007)	78.3 [74.2,81.9] (2018)	-1.0	.000
Greece	96.4 [94.6,97.7] (2016)	95.9 [92.7,97.8] (2018)	-0.3	.890	95.2 [92.7,96.9] (2016)	94.9 [90.8,97.3] (2018)	-0.2	.430	68.8 [63.4,73.7] (2016)	79.8 [74.7,84.1] (2018)	+5.5	.003
Hungary	84.4 [80.5,87.7] (2016)	82.8 [77.8,86.9] (2018)	-0.8	.320	68.9 [62.7,74.4] (2016)	69.3 [63.5,74.6] (2018)	+0.2	.830	56.0 [50.0,61.7] (2016)	64.9 [59.2,70.1] (2018)	+4.5	.037
India	89.2 [86.5,91.4] (2010-11)	92.1 [87.7,94.9] (2018-19)	+0.4	.130	75.5 [71.2,79.3] (2010-11)	85.4 [80.9,89.0] (2018-19)	+1.2	.010	62.9 [57.6,67.9] (2010-11)	73.6 [67.1,79.3] (2018-19)	+1.3	.006
Japan	86.2 [84.9,87.4] (2018)	80.3 [77.5,82.9] (2021)	-2.0	.007	77.1 [75.5,78.6] (2018)	64.2 [61.3,67.1] (2021)	-4.3	.002	NA	NA	NA	NA
Kenya	90.0 [86.8,92.5] (2012)	91.1 [87.9,93.4] (2018)	+0.2	.700	65.7 [58.8,72.1] (2012)	78.9 [74.6,82.7] (2018)	+2.2	.010	44.7 [37.9,51.8] (2012)	68.2 [63.2,72.9] (2018)	+3.9	.000
Malaysia	87.3 [83.3,90.5] (2005)	87.3 [84.2,89.8] (2020)	0.0	.880	92.0 [90.3,93.4] (2009)	81.5 [78.4,84.2] (2020)	-1.0	.080	70.4 [65.2,75.2] (2005)	65.0 [61.2,68.6] (2020)	-0.4	.610

For peer review only - http://bmjopen.bmj.com/site/about/guidelines.xhtml

ITC Country	Smoking causes lung cancer				Smoking causes heart disease				Smoking causes stroke			
	First survey % [95% CI] (year)	Last survey % [95% CI] (year)	Average PP change per year	p-value	First survey % [95% CI] (year)	Last survey % [95% CI] (year)	Average PP change per year	p-value	First survey % [95% CI] (year)	Last survey % [95% CI] (year)	Average PP change per year	p-value
Mauritius	94.0 [90.7,96.2] (2009)	91.4 [87.1,94.4] (2011)	-1.3	.740	90.3 [85.9,93.4] (2009)	84.8 [80.0,88.6] (2011)	-2.8	.400	66.0 [59.6,71.9] (2009)	75.3 [69.5,80.3] (2011)	+4.6	.008
Mexico	97.2 [95.5,98.2] (2006)	96.5 [93.6,98.1] (2014-15)	-0.1	.980	83.0 [79.8,85.7] (2006)	79.6 [74.1,84.2] (2014-15)	-0.4	.700	55.9 [50.6,61.1] (2006)	74.8 [69.8,79.3] (2014-15)	+2.2	.004
Netherlands	81.4 [77.5,84.7] (2011)	91.8 [90.1,93.1] (2022)	0.9	.000	83.8 [81.9,85.5] (2008)	85.2 [83.2,87.1] (2022)	+0.1	.130	55.6 [52.1,59.0] (2011)	70.7 [68.2,73.1] (2022)	+1.4	.001
New Zealand	NA	NA	NA	NA	NA	NA	NA	NA	78.2 [74.9,81.1] (2007-08)	76.3 [72.5,79.8] (2020-21)	-0.1	.760
Poland	88.5 [84.6,91.6] (2016)	84.7 [79.8,88.6] (2018)	-1.9	.180	83.8 [78.3,88.1] (2016)	78.9 [73.0,83.8] (2018)	-2.4	.180	61.2 [55.4,66.7] (2016)	64.5 [58.7,69.9] (2018)	+1.6	.740
Republic of Korea	91.2 [89.3,92.8] (2020)	87.9 [85.4,90.1] (2021)	-3.3	.770	66.2 [62.6,69.7] (2020)	66.3 [63.1,69.3] (2021)	0.0	.940	37.0 [33.5,40.6] (2005)	71.0 [66.1,75.4] (2021)	+2.1	.000
Romania	90.3 [87.6,92.4] (2016)	86.3 [81.9,89.7] (2018)	-2.0	.180	88.5 [85.1,91.2] (2016)	86.6 [82.4,90.0] (2018)	-0.9	.950	78.5 [74.8,81.8] (2016)	76.5 [71.2,81.2] (2018)	-1.0	.520
Spain	93.2 [88.9,95.9] (2016)	94.6 [91.8,96.4] (2021)	+0.3	.420	82.8 [77.5,87.1] (2016)	88.4 [85.5,90.8] (2021)	+1.1	.040	56.6 [49.9,63.0] (2016)	65.6 [59.8,70.9] (2021)	+1.8	.050
Thailand	94.3 [91.5,96.1] (2005)	94.0 [92.9,95.0] (2008)	-0.1	.840	69.0 [65.8,72.0] (2009)	71.2 [66.8,75.3] (2012)	+0.7	.540	44.0 [40.6,47.4] (2005)	83.9 [81.6,85.9] (2012)	-5.7	.000
United States	94.4 [93.2,95.3] (2002)	92.1 [90.6,93.4] (2003)	-2.2	.250	86.4 [84.7,88.0] (2002)	86.4 [84.0,88.4] (2020)	0.0	.930	73.3 [71.1,75.4] (2002)	77.2 [74.4,79.8] (2020)	+0.2	.800
Uruguay	96.8 [94.3,98.2] (2006)	96.0 [94.1,97.3] (2014)	-0.1	.530	95.3 [93.0,96.9] (2006)	89.4 [86.6,91.7] (2014)	-0.7	.030	43.0 [38.4,47.8] (2006)	65.1 [61.5,68.5] (2014)	+2.8	.000
Zambia	82.0 [79.0,84.6] (2012)	79.0 [73.8,83.5] (2014)	-1.5	.080	76.1 [71.9,79.9] (2012)	72.2 [68.9,75.2] (2014)	-2.0	.100	45.1 [38.7,51.6] (2012)	48.1 [43.1,53.2] (2014)	+1.5	.280

Abbreviations: NA=no data available; PP=percentage points

[a] Data collected in the UK in 2002 and 2003, and England in 2020.

For peer review only - http://bmjopen.bmj.com/site/about/guidelines.xhtml

Supplementary table 8. Changes in knowledge that smoking causes lung cancer, heart attack, and stroke among adults who smoke in GATS countries.

GATS Country	Smoking causes lung cancer				Smoking causes heart attack				Smoking causes stroke			
	First survey % [95% CI] (year)	Last survey % [95% CI] (year)	Average PP change per year	p-value	First survey % [95% CI] (year)	Last survey % [95% CI] (year)	Average PP change per year	p-value	First survey % [95% CI] (year)	Last survey % [95% CI] (year)	Average PP change per year	p-value
Argentina	98.4 [97.1, 99.1] (2012)	NA	NA	NA	92.1 [88.7, 94.5] (2012)	NA	NA	NA	72.8 [64.7, 79.6] (2012)	NA	NA	NA
Bangladesh	93.0 [91.1, 94.4] (2009)	97.1 [95.8, 98.0] (2017)	+0.5	.015	88.9 [86.7, 90.8] (2009)	93.5 [91.9, 94.8] (2017)	+0.6	.009	85.9 [83.3, 88.2] (2009)	94.1 [92.4, 95.5] (2017)	+1.0	.000
Botswana	92.6 [89.5, 94.8] (2017)	NA	NA	NA	81.0 [76.6, 84.7] (2017)	NA	NA	NA	74.0 [68.9, 78.4] (2017)	NA	NA	NA
Brazil	97.5 [97.1, 98.0] (2008)	NA	NA	NA	88.6 [87.5, 89.5] (2008)	NA	NA	NA	76.7 [75.3, 78.1] (2008)	NA	NA	NA
Cameroon	89.9 [85.5, 93.1] (2013)	NA	NA	NA	68.6 [61.2, 75.1] (2013)	NA	NA	NA	61.3 [54.5, 67.7] (2013)	NA	NA	NA
China	79.4 [75.6, 82.8] (2010)	78.4 [76.1, 80.5] (2018)	-0.1	.820	38.5 [34.3, 42.8] (2010)	46.4 [43.5, 49.3] (2018)	+1.0	.110	26.9 [23.0, 31.2] (2010)	37.4 [34.7, 40.2] (2018)	+1.3	0.018
Costa Rica	96.9 [95.0, 98.0] (2015)	NA	NA	NA	93.3 [91.0, 95.1] (2015)	NA	NA	NA	72.0 [66.9, 76.6] (2015)	NA	NA	NA
Egypt	97.4 [96.2, 98.2] (2009)	NA	NA	NA	96.9 [96.0, 97.6] (2009)	NA	NA	NA	89.8 [88.2, 91.2] (2009)	NA	NA	NA
Ethiopia	67.1 [48.2, 81.7] (2016)	NA	NA	NA	54.6 [39.2, 69.1] (2016)	NA	NA	NA	29.6 [19.2, 42.8] (2016)	NA	NA	NA
Greece	95.1 [92.7, 96.8] (2013)	NA	NA	NA	88.1 [83.5, 91.5] (2013)	NA	NA	NA	69.3 [61.8, 75.9] (2013)	NA	NA	NA
India	86.2 [84.7, 87.6] (2009)	91.9 [90.9, 92.8] (2016)	0.8	.042	62.3 [60.1, 64.4] (2009)	75.3 [73.6, 77.0] (2016)	+1.9	.000	47.4 [45.0, 49.7] (2009)	63.1 [61.1, 65.1] (2016)	+2.3	.000
Indonesia	78.8 [75.4, 81.9] (2011)	NA	NA	NA	78.0 [74.2, 81.3] (2011)	NA	NA	NA	40.4 [35.6, 45.3] (2011)	NA	NA	NA
Kazakhstan	78.9 [75.1, 82.3] (2014)	NA	NA	NA	54.8 [50.4, 59.0] (2014)	NA	NA	NA	55.2 [50.8, 59.5] (2014)	NA	NA	NA

For peer review only

For peer review only - http://bmjopen.bmj.com/site/about/guidelines.xhtml

GATS Country	Smoking causes lung cancer				Smoking causes heart attack				Smoking causes stroke			
	First survey % [95% CI] (year)	Last survey % [95% CI] (year)	Average PP change per year	p-value	First survey % [95% CI] (year)	Last survey % [95% CI] (year)	Average PP change per year	p-value	First survey % [95% CI] (year)	Last survey % [95% CI] (year)	Average PP change per year	p-value
Kenya	85.6 [80.6, 89.4] (2014)	NA	NA	NA	60.0 [53.7, 66.0] (2014)	NA	NA	NA	45.7 [38.4, 53.1] (2014)	NA	NA	NA
Malaysia	88.7 [85.1, 91.5] (2011)	NA	NA	NA	84.8 [80.9, 88.1] (2011)	NA	NA	NA	75.4 [70.9, 79.4] (2011)	NA	NA	NA
Mexico	97.5 [96.5, 98.3] (2009)	98.0 [97.1, 98.6] (2015)	+0.1	.590	84.3 [81.9, 86.5] (2009)	87.5 [85.3, 89.4] (2015)	+0.5	.120	60.6 [57.5, 63.6] (2009)	68.1 [65.2, 70.8] (2015)	+1.2	.046
Nigeria	55.6 [48.4, 62.6] (2012)	NA	NA	NA	64.3 [57.3, 70.8] (2012)	NA	NA	NA	32.2 [25.5, 39.8] (2012)	NA	NA	NA
Pakistan	83.6 [79.8, 86.8] (2014)	NA	NA	NA	86.9 [83.7, 89.6] (2014)	NA	NA	NA	54.6 [49.1, 60.1] (2014)	NA	NA	NA
Panama	96.0 [94.1, 97.3] (2013)	NA	NA	NA	84.0 [78.2, 88.5] (2013)	NA	NA	NA	76.8 [70.0, 82.4] (2013)	NA	NA	NA
Philippines	92.4 [90.6, 93.8] (2009)	93.6 [92.2, 94.8] (2015)	+0.2	.520	76.6 [74.1, 78.9] (2009)	82.4 [79.9, 84.6] (2015)	+1.0	.028	69.9 [67.1, 72.5] (2009)	75.3 [72.3, 78.2] (2015)	+0.9	.090
Poland	94.9 [93.6, 95.9] (2009)	NA	NA	NA	79.8 [77.5, 82.0] (2009)	NA	NA	NA	58.6 [55.7, 61.5] (2009)	NA	NA	NA
Qatar	90.0 [86.4, 92.7] (2013)	NA	NA	NA	88.7 [85.3, 91.4] (2013)	NA	NA	NA	70.0 [64.9, 74.6] (2013)	NA	NA	NA
Romania	96.8 [95.4, 97.7] (2011)	87.5 [84.3, 90.1] (2018)	-1.3	.000	87.0 [84.3, 89.2] (2011)	72.4 [68.1, 76.3] (2018)	-2.1	.000	87.2 [84.5, 89.6] (2011)	73.2 [68.7, 77.3] (2018)	-2.0	.000
Russian Federation	96.0 [95.0, 96.8] (2009)	87.8 [85.8, 89.6] (2016)	-1.2	.000	69.2 [66.4, 71.8] (2009)	74.1 [71.2, 76.8] (2016)	+0.7	.170	63.5 [60.5, 66.3] (2009)	72.0 [68.9, 74.9] (2016)	+1.2	.027
Senegal	88.4 [83.0, 92.3] (2015)	NA	NA	NA	71.4 [64.4, 77.5] (2015)	NA	NA	NA	66.1 [58.6, 72.8] (2015)	NA	NA	NA
Thailand	97.2 [96.4, 97.8] (2009)	96.3 [95.0, 97.2] (2011)	-0.4	.080	74.5 [72.2, 76.6] (2009)	74.9 [72.3, 77.3] (2011)	+0.2	.670	79.7 [77.2, 82.1] (2009)	78.7 [76.1, 81.0] (2011)	-0.5	.510
Turkey	98.7 [98.0, 99.2] (2008)	95.3 [94.0, 96.3] (2016)	-0.4	.002	96.3 [95.2, 97.1] (2008)	95.3 [94.1, 96.3] (2016)	-0.1	.460	85.2 [83.2, 87.0] (2008)	87.7 [85.7, 89.4] (2016)	+0.3	.290
Uganda	82.1 [77.9, 85.7] (2013)	NA	NA	NA	68.1 [62.7, 73.0] (2013)	NA	NA	NA	36.4 [30.5, 42.8] (2013)	NA	NA	NA

For peer review only - http://bmjopen.bmj.com/site/about/guidelines.xhtml

GATS Country	Smoking causes lung cancer				Smoking causes heart attack				Smoking causes stroke			
	**First survey % [95% CI] (year)**	**Last survey % [95% CI] (year)**	**Average PP change per year**	**p-value**	**First survey % [95% CI] (year)**	**Last survey % [95% CI] (year)**	**Average PP change per year**	**p-value**	**First survey % [95% CI] (year)**	**Last survey % [95% CI] (year)**	**Average PP change per year**	**p-value**
Ukraine	92.2 [90.5, 93.6] (2010)	86.9 [83.7, 89.5] (2017)	-0.8	.060	77.1 [74.5, 79.5] (2010)	77.9 [74.6, 80.9] (2017)	+0.1	.780	75.0 [72.2, 77.6] (2010)	76.8 [73.5, 79.7] (2017)	+0.3	.550
United Republic of Tanzania	81.0 [76.0, 85.1] (2018)	NA	NA	NA	57.6 [51.4, 63.6] (2018)	NA	NA	NA	39.8 [33.5, 46.4] (2018)	NA	NA	NA
Uruguay	97.2 [95.8, 98.2] (2009)	95.9 [94.0, 97.2] (2017)	-0.2	.300	93.3 [91.0, 95.1] (2009)	91.4 [89.2, 93.2] (2017)	-0.2	.370	79.9 [76.6, 82.9] (2009)	76.8 [73.9, 79.6] (2017)	-0.4	.480
Viet Nam	94.5 [93.1, 95.6] (2010)	94.7 [93.1, 96.0] (2015)	0.0	.920	58.1 [55.2, 61.0] (2010)	68.6 [65.6, 71.4] (2015)	+2.1	.001	65.6 [62.7, 68.4] (2010)	68.3 [65.5, 71.0] (2015)	+0.5	.360

Abbreviations: NA=no data available; PP=percentage points

For peer review only

For peer review only - http://bmjopen.bmj.com/site/about/guidelines.xhtml

Supplementary table 9. Changes in knowledge that SHS causes lung cancer and heart disease among adults who smoke in ITC countries.

	**Secondhand smoke causes lung cancer**				**Secondhand smoke causes heart disease**			
**ITC Country**	**First survey % [95% CI] (year)**	**Last survey % [95% CI] (year)**	**Average PP change per year**	**p-value**	**First survey % [95% CI] (year)**	**Last survey % [95% CI] (year)**	**Average PP change per year**	**p-value**
Australia	60.6 [57.8,63.3] (2003)	72.5 [69.4,75.3] (2008-09)	+2.2	.003	49.1 [45.4,52.8] (2010-11)	39.3 [35.3,43.5] (2013)	-3.9	.024
Bangladesh	87.1 [81.0,91.4] (2009)	94.9 [92.1,96.7] (2014-15)	+1.4	.000	56.9 [51.0,62.7] (2010)	88.4 [82.6,92.4] (2014-15)	+7.0	.000
Brazil	78.0 [75.2,80.6] (2009)	80.7 [77.9,83.2] (2016-17)	+0.3	.380	NA	NA	NA	NA
Canada	74.3 [71.9,76.5] (2003)	76.8 [74.8,78.7 (2020)	+0.1	.690	61.6 [58.1,64.9] (2010-11)	50.2 [47.9,52.5] (2020)	-1.2	.250
China	56.9 [54.6,59.2] (2006)	73.0 [69.4,76.3] (2013-15)	+2.0	.000	43.3 [40.6,46.1] (2009)	53.7 [50.7,56.7] (2013-15)	+2.1	.036
England/UK[a]	59.7 [56.9,62.4] (2003)	65.9 [63.2,68.4] (2020)	+0.4	.890	53.0 [48.8,57.2] (2010-11)	44.8 [42.2,47.5] (2020)	-0.9	.600
France	94.0 [92.6,95.2] (2006-07)	75.7 [72.9,78.3] (2019)	-1.5	.000	55.8 [52.8,58.9] (2019)	NA	NA	NA
Germany	80.6 [78.2,82.8] (2007)	58.2 [53.5,62.8] (2018)	-2.0	.000	41.8 [35.2,48.7] (2016)	44.9 [38.3,51.8] (2018)	+1.6	.310
Greece	70.8 [64.5,76.4] (2016)	64.8 [58.8,70.3] (2018)	-3.0	.110	63.8 [57.6,69.7] (2016)	59.8 [53.1,66.1] (2018)	-2.0	.330
Hungary	58.7 [52.4,64.7] (2016)	60.1 [53.8,66.1] (2018)	+0.7	.770	44.5 [37.8,51.4] (2016)	49.0 [42.9,55.1] (2018)	+2.2	.220
India	74.8 [70.6,78.5] (2010-11)	88.4 [84.6,91.3] (2019-19)	+1.7	.000	65.0 [60.6,69.1] (2010-11)	86.9 [82.7,90.2] (2018-19)	+2.7	.000
Japan	74.9 [73.3,76.4] (2018)	68.9 [66.5,71.1] (2021)	-2.0	.080	64.3 [62.4,66.1] (2018)	56.2 [53.4,59.0] (2021)	-2.7	.046
Kenya	68.8 [63.4,73.7] (2012)	74.3 [69.1,79.0] (2018)	+0.9	.120	50.9 [43.6,58.1] (2012)	63.9 [58.1,69.3] (2018)	+2.2	.041
Malaysia	81.2 [76.3,85.3] (2005)	72.3 [68.6,75.8] (2020)	-0.6	.270	82.5 [80.1,84.7] (2013-14)	62.6 [58.6,66.4] (2020)	-3.3	.000

For peer review only

	Secondhand smoke causes lung cancer				Secondhand smoke causes heart disease			
ITC Country	First survey % [95% CI] (year)	Last survey % [95% CI] (year)	Average PP change per year	p-value	First survey % [95% CI] (year)	Last survey % [95% CI] (year)	Average PP change per year	p-value
Mauritius	84.3 [79.5,88.1] (2009)	85.2 [79.8,89.2] (2011)	+0.4	.210	NA	NA	NA	NA
Mexico	90.7 [87.9,92.9] (2006)	91.2 [87.2,94.1] (2014-15)	+0.1	.330	74.7 [71.2,78.0] (2008)	79.1 [73.7,83.7] (2014-15)	+0.7	.110
Netherlands	69.8 [67.5,72.1] (2008)	63.9 [61.3,66.5] (2022)	-0.4	.490	38.1 [35.1,41.3] (2012)	53.8 [51.2,56.3] (2022)	+1.6	.023
New Zealand	80.8 [77.9,83.4] (2007-08)	61.2 [57.1,65.2] (2020-21)	-1.5	.019	58.0 [53.8,62.0] (2016-17)	47.8 [43.8,51.8] (2020-21)	-2.5	.027
Poland	73.5 [68.0,78.4] (2016)	68.8 [61.4,75.3] (2018)	-2.4	.290	65.7 [59.7,71.2] (2016)	60.1 [52.6,67.1] (2018)	-2.8	.130
Republic of Korea	79.5 [76.7,82.1] (2005)	75.4 [69.6,80.4] (2021)	-0.3	.100	55.8 [52.8,58.7] (2010)	60.3 [55.6,64.8] (2021)	+0.4	.780
Romania	81.9 [78.3,85.1] (2016)	77.7 [72.3,82.3] (2018)	-2.1	.400	73.8 [68.9,78.1] (2016)	73.8 [68.7,78.4] (2018)	0.0	.780
Spain	67.2 [60.3,73.5] (2016)	69.8 [64.9,74.3] (2021)	+0.5	.550	51.8 [44.7,58.9] (2016)	56.5 [50.8,62.0] (2021)	+0.9	.440
Thailand	91.1 [88.2,93.4] (2005)	96.8 [95.4,97.8] (2012)	+0.8	.000	82.2 [78.2,85.6] (2012)	NA	NA	NA
United States	62.7 [60.0,65.2] (2003)	68.1 [65.1,70.9] (2020)	+0.3	.990	51.6 [47.5,55.6] (2010-11)	44.7 [41.4,47.9] (2020)	-0.7	.440
Uruguay	89.3 [86.5,91.6] (2006)	79.5 [76.0,82.7] (2014)	-1.2	.015	69.1 [64.3,73.5] (2012)	69.6 [65.9,73.1] (2014)	+0.2	.900
Zambia	64.3 [57.0,70.9] (2012)	68.2 [62.9,73.1] (2014)	+2.0	.160	59.4 [52.2,66.2] (2012)	65.3 [60.5,69.9] (2014)	+3.0	.080

Abbreviations: NA=no data available; PP=percentage points
[a] Data collected in the UK in 2003 and 2010-11, and England in 2020.

For peer review only

For peer review only - http://bmjopen.bmj.com/site/about/guidelines.xhtml

Supplementary table 10. Changes in knowledge that SHS causes lung cancer and heart disease among adults who smoke in GATS countries.

GATS Country	Secondhand smoke causes lung cancer				Secondhand smoke causes heart disease			
	First survey % [95% CI] (year)	Last survey % [95% CI] (year)	Average PP change per year	p-value	First survey % [95% CI] (year)	Last survey % [95% CI] (year)	Average PP change per year	p-value
Argentina	NA	NA	NA	NA	NA	NA	NA	NA
Bangladesh	93.0 [91.3, 94.4] (2009)	95.4 [94.1, 96.4] (2017)	+0.3	.360	90.6 [88.6, 92.3] (2009)	93.5 [91.9, 94.8] (2017)	+0.4	.160
Botswana	NA	NA	NA	NA	NA	NA	NA	NA
Brazil	NA	NA	NA	NA	NA	NA	NA	NA
Cameroon	78.8 [73.3, 83.4] (2013)	NA	NA	NA	72.9 [65.5, 79.2] (2013)	NA	NA	NA
China	80.5 [75.8, 84.4] (2010)	61.6 [59.0, 64.2] (2018)	-2.4	.050	43.8 [37.7, 50.0] (2010)	37.7 [35.1, 40.4] (2018)	-0.8	.360
Costa Rica	93.5 [90.8, 95.5] (2015)	NA	NA	NA	88.2 [84.8, 90.9] (2015)	NA	NA	NA
Egypt	94.0 [92.7, 95.1] (2009)	NA	NA	NA	91.8 [90.3, 93.1] (2009)	NA	NA	NA
Ethiopia	NA	NA	NA	NA	NA	NA	NA	NA
Greece	68.1 [61.1, 74.4] (2013)	NA	NA	NA	66.9 [59.8, 73.4] (2013)	NA	NA	NA
India	NA	NA	NA	NA	NA	NA	NA	NA
Indonesia	NA	NA	NA	NA	NA	NA	NA	NA
Kazakhstan	NA	NA	NA	NA	NA	NA	NA	NA
Kenya	78.6 [72.4, 83.7] (2014)	NA	NA	NA	67.4 [60.7, 73.5] (2014)	NA	NA	NA
Malaysia	81.2 [77.3, 84.6] (2011)	NA	NA	NA	77.4 [73.0, 81.2] (2011)	NA	NA	NA
Mexico	NA	NA	NA	NA	NA	NA	NA	NA
Nigeria	41.9 [35.1, 49.0] (2012)	NA	NA	NA	54.0 [46.6, 61.2] (2012)	NA	NA	NA
Pakistan	69.3 [64.6, 73.5] (2014)	NA	NA	NA	73.1 [68.6, 77.1] (2014)	NA	NA	NA
Panama	NA	NA	NA	NA	NA	NA	NA	NA
Philippines	NA	NA	NA	NA	NA	NA	NA	NA
Poland	91.4 [89.7, 92.9] (2009)	NA	NA	NA	83.7 [81.4, 85.8] (2009)	NA	NA	NA

For peer review only - http://bmjopen.bmj.com/site/about/guidelines.xhtml

GATS Country	Secondhand smoke causes lung cancer				Secondhand smoke causes heart disease			
	First survey % [95% CI] (year)	Last survey % [95% CI] (year)	Average PP change per year	p-value	First survey % [95% CI] (year)	Last survey % [95% CI] (year)	Average PP change per year	p-value
Qatar	NA	NA	NA	NA	NA	NA	NA	NA
Romania	NA	NA	NA	NA	NA	NA	NA	NA
Russian Federation	NA	NA	NA	NA	NA	NA	NA	NA
Senegal	83.8 [77.9, 88.4] (2015)	NA	NA	NA	85.9 [80.4, 90.1] (2015)	NA	NA	NA
Thailand	95.3 [94.3, 96.1] (2009)	96.4 [95.5, 97.1] (2011)	+0.6	.090	70.4 [67.8, 72.9] (2009)	68.5 [65.8, 71.2] (2011)	-0.9	.340
Turkey	93.5 [92.2, 94.7] (2012)	93.0 [91.5, 94.3] (2016)	-0.1	.830	91.3 [89.3, 92.9] (2012)	92.8 [91.3, 94.0] (2016)	+0.4	.240
Uganda	72.1 [66.6, 77.1] (2013)	NA	NA	NA	66.0 [60.1, 71.4] (2013)	NA	NA	NA
Ukraine	75.3 [72.4, 78.1] (2010)	69.4 [65.9, 72.6] (2017)	-0.9	.490	78.8 [76.3, 81.2] (2010)	71.1 [67.8, 74.1] (2017)	-1.1	.100
United Republic of Tanzania	67.0 [61.0, 72.4] (2018)	NA	NA	NA	56.0 [50.0, 61.9] (2018)	NA	NA	NA
Uruguay	NA	NA	NA	NA	NA	NA	NA	NA
Viet Nam	71.6 [68.8, 74.2] (2010)	85.1 [82.7, 87.3] (2015)	+2.7	.010	14.6 [12.5, 16.9] (2010)	21.3 [19.0, 23.9] (2015)	+1.4	.018

Abbreviations: NA=no data available; PP=percentage points

For peer review only - http://bmjopen.bmj.com/site/about/guidelines.xhtml

Supplementary file 1A. ITC and GATS Project authors, ITC principal investigators, ITC funding statements, ITC ethics statements, and ITC acknowledgements.

Janet Chung-Hall,[1] Geoffrey T. Fong, [1,2,3] Gang Meng,[1] Lorraine V. Craig,[1] Eunice O. Indome[1], for the ITC Project Collaborators, Alissa C. Kress,[4] Jing Shi,[4,5] and Indu B. Ahluwalia[4] for the Global Adult Tobacco Survey Team

Affiliation(s):
1. University of Waterloo, Psychology, Waterloo, ON, Canada
2. University of Waterloo, School of Public Health Sciences, Waterloo, ON, Canada
3. Ontario Institute for Cancer Research, Toronto, ON, Canada
4. National Center for Chronic Disease Prevention and Health Promotion Office on Smoking and Health, Atlanta, GA, US
5. CDC Foundation Inc., Noninfectious Disease Programs, Atlanta, GA, US

ITC Project Collaborators Individual Author Names and Email Addresses:

Raphaël Andler[6] (Raphael.ANDLER@santepubliquefrance.fr), Maansi Bansal-Travers[7] (Maansi.Travers@roswellpark.org), Tonatiuh Barrientos-Gutiérrez[8] (tbarrientos@insp.mx), Marcelo Boado[9] (mrbmsoc2001@yahoo.com.ar), Premduth Burhoo[10] (burhoo@hotmail.com), K. Michael Cummings[11] (cummingk@musc.edu), Cristina de Abreu Perez[12] (cristinadeabreuperez@gmail.com), Tibor Demjén[13] (tibor.demjen@gmail.com), Katherine A. East[14] (K.East@bsms.ac.uk), Richard Edwards[15] (richard.edwards@flinders.edu.au), Esteve Fernández[16-20] (efernandez@iconcologia.net), Marcela Fu[16-19,21] (mfu@iconcologia.net), Coral Gartner[22] (c.gartner@uq.edu.au), Fastone M. Goma[23] (gomafm@yahoo.co.uk), Prakash C. Gupta[24] (pcgupta@healis.org), A.K.M. Ghulam Hussain[25] (think2100@gmail.com), Andrew Hyland[7] (Andrew.Hyland@RoswellPark.org), Lawrence Ikamari[26] (likamari@uonbi.ac.ke), Aree Jampaklay[27] (aree.ude@mahidol.ac.th), Yuan Jiang[28,29] (jiangyuan88@vip.sina.com), Kota Katanoda[30] (kkatanod@ncc.go.jp), Gil-yong Kim[31] (kimgy@khepi.or.kr) , Su Young Kim[32] (suyoung@ncrc.or.kr), Sungkyu Lee[33,34] (wwwvince77@gmail.com), Farizah Mohd Hairi[35,36] (farizah@um.edu.my), Ute Mons[37–39] (ute.mons@uk-koeln.de), Nigar Nargis[40] (nigar.nargis@cancer.org), Amer Siddiq Amer Nordin[35,36] (amersiddiq@um.edu.my), Jane Rahedi Ong'ang'o[41] (jrnabongo@gmail.com), Maizurah Omar[42] (maizurahomar@gmail.com), Mangesh S. Pednekar[24] (pednekarm@healis.org), Krzysztof Przewoźniak[43,44] (krzysztof.przewozniak@wp.pl), Hong Gwan Seo[45] (hgseo@designhospital.co.krmailto:), James F. Thrasher[8,46] (THRASHER@mailbox.sc.edu), Antigona Trofor[47] (atrofor@yahoo.com), Constantine I. Vardavas[48] (vardavas@tobcontrol.eu), Andrew M. Waa[49] (andrew.waa@otago.ac.nz), Marc C. Willemsen[50] (marc.willemsen@maastrichtuniversity.nl), Nick Wilson[49] (nick.wilson@otago.ac.nz), Witold A Zatoński[51-53] (wazatonski@gmail.com)

ITC Project Collaborators Affilation(s):

6. Prevention and Health Promotion Department, Santé publique France, Saint-Maurice, France
7. Department of Health Behavior, Roswell Park Comprehensive Cancer Center, Buffalo, NY, USA
8. Center for Population Health Research, National Institute of Public Health, Cuernavaca, Mexico
9. Department of Sociology, Faculty of Social Sciences, University of the Republic, Montevideo, Uruguay
10. Mauritius Institute of Health, Pamplemousses, Mauritius

For peer review only - http://bmjopen.bmj.com/site/about/guidelines.xhtml

11. Department of Psychiatry & Behavioral Sciences, Medical University of South Carolina, Charleston, South Carolina, USA
12. National School of Public Health Sérgio Arouca, Oswaldo Cruz Foundation, Rio de Janeiro, Brazil
13. Hungarian Focal Point for Tobacco Control, Ministry of Interior, Hungary
14. Department of Primary Care and Public Health, Brighton and Sussex Medical School, Brighton, Sussex, UK
15. Department of Public Health, University of Otago, Wellington, New Zealand
16. Tobacco Control Unit, WHO Collaborating Center, Institut Català d'Oncologia (ICO), l'Hospitalet de Llobregat, Spain
17. Tobacco Control Research Group, Epidemiology, Public Health, Cancer Prevention and Palliative Care Programme-EPIBELL, Institut d'Investigació Biomèdica de Bellvitge (IDIBELL), l'Hospitalet de Llobregat, Spain
18. Centre for Biomedical Research in Respiratory Disease, Institute of Health Carlos III, Madrid, Spain
19. Department of Clinical Sciences, School of Medicine and Health Sciences, University of Barcelona, Barcelona, Catalonia, Spain
20. Secretariat of Public Health, Department of Health, Generalitat de Catalunya, Barcelona, Spain
21. Department of Public Health, Mental health, and Maternal and Child Health Nursing, School of Nursing, University of Barcelona, Barcelona, Catalonia, Spain
22. NHMRC Centre of Research Excellence on Achieving the Tobacco Endgame, Faculty of Health, Medicine, and Behavioural Sciences, The University of Queensland, Brisbane, Queensland, Australia
23. Centre for Primary Care Research, University of Zambia, Lusaka, Zambia
24. Healis Sekhsaria Institute for Public Health, Navi Mumbai, Maharashtra, India
25. Department of Economics, University of Dhaka, Dhaka, Bangladesh
26. Population Studies and Research Institute, University of Nairobi, Nairobi, Kenya
27. Institute for Population and Social Research, Mahidol University, Salaya, Thailand
28. National Tobacco Control Office, Chinese Center for Disease Control and Prevention, Beijing, China
29. ThinkTank Research Center for Health Development, Beijing, China
30. National Cancer Center Institute for Cancer Control, Tokyo, Japan
31. National Tobacco Control Center, Korean Health Promotion Institute, Seoul, Republic of Korea
32. National Center for the Rights of the Child, Seoul, Republic of Korea
33. Korea Center for Tobacco Control Research and Education, Seoul, Republic of Korea
34. Graduate School of Public Health, Institute for Health Promotion, Yonsei University, Seoul, Republic of Korea
35. Nicotine Addiction Research Group, University of Malaya Centre of Addiction Sciences, University of Malaya, Kuala Lumpur, Malaysia
36. Department of Social and Preventive Medicine, Faculty of Medicine, University of Malaya, Kuala Lumpur, Malaysia
37. Division of Primary Cancer Prevention, German Cancer Research Center (DKFZ), Heidelberg, Germany
38. Cancer Prevention Unit & WHO Collaborating Centre, German Cancer Research Center (DKFZ), Heidelberg, Germany
39. Medical Faculty Mannheim, Heidelberg University, Mannheim, Germany
40. Department of Surveillance, Prevention, and Health Services Research, American Cancer Society, Atlanta, GA, USA
41. Centre for Respiratory Disease Research, Kenya Medical Research Institute, Nairobi, Kenya
42. National Poison Centre, Universiti Sains Malaysia, Penang, Malaysia
43. Global Institute of Family Health, University of Kalisz, Kalisz, Poland
44. Global Research, Implementation and Training Lab, Department of Population and Public Health Sciences, Keck School of Medicine, University of Southern California, Los Angeles, CA, USA
45. Department of Disease Screening, Design Hospital, Jeonju, Republic of Korea
46. Department of Health Promotion, Education & Behavior, Arnold School of Public Health, University of South Carolina, Columbia, USA

For peer review only - http://bmjopen.bmj.com/site/about/guidelines.xhtml

47. Grigore T. Popa University of Medicine and Pharmacy, Iasi, Romania
48. Department of Hygiene, Epidemiology and Medical Statistics, School of Medicine, National and Kapodistrian University of Athens, Greece
49. College of Medicine and Public Health, Flinders University, South Australia, Australia
50. Department of Health Promotion, Maastricht University (CAPHRI), Maastricht, The Netherlands
51. Maria Skłodowska-Curie National Research Institute of Oncology, Warsaw, Poland
52. European Observatory of Health Inequalities, President Stanisław Wojciechowski State University of Applied Sciences, Kalisz, Poland
53. Global Institute of Family Health, University of Kalisz, Kalisz, Poland

Ethics statement:

The **ITC Four Country** (Australia, Canada, United Kingdom, and United States) survey protocols and all materials, including the survey questionnaires, were cleared for ethics by Research Ethics Board, University of Waterloo, Canada (REB#10556, REB#13978, REB#17469), Human Research Ethics at Cancer Council Victoria, Australia (IRB HREC 0211), Office of Research Subject Protection, Roswell Comprehensive Cancer Center, US (IRB NT 02-20), and Research Ethics Office, King's College London, UK (IRB PNM/13/14-151). The **ITC Four Country Smoking and Vaping** (Australia, Canada, England, and United States) survey protocols and all materials, including the survey questionnaires, were cleared for ethics by Research Ethics Board, University of Waterloo, Canada (REB#20803/30570, REB#20803/30878), Human Research Ethics at Cancer Council Victoria, Australia (IRB HREC 1603), Human Research Ethics Committee, The University of Queensland, Australia (IRB #2016000330), Research Ethics Office, King's College London, UK (IRB RESCM-17/18-2240), and ethics clearance at the Medical University of South Carolina, US was waived due to minimal risk. The **ITC Bangladesh** survey protocols and all materials, including the survey questionnaires, were cleared for ethics by Research Ethics Board, University of Waterloo, Canada (REB#15019, REB#19862/30312), and Bangladesh Medical Research Council (IRB BMRC/ERC/2013-2016/1729, IRB BMRC/ERC/2007-2010/1372). The **ITC Brazil** survey protocols and all materials, including the survey questionnaires, were cleared for ethics by Research Ethics Board, University of Waterloo, Canada (REB#15076, REB#21456/30826), and National Cancer Institute of Brazil (INCA)—IRB 99/08). The **ITC China** survey protocols and all materials, including the survey questionnaires, were cleared for ethics by Research Ethics Board, University of Waterloo, Canada (REB#12539, REB#15305, REB#17014/30105), Cancer Council Victoria, Australia (IRB IER0803), and Chinese Center for Disease Control and Prevention (IRB 201114, IRB 201325). The **ITC France** (first cohort) survey protocols and all materials, including the survey questionnaires, were cleared for ethics by Research Ethics Board, University of Waterloo, Canada (REB#13005, 183/30190). The **ITC France** (second cohort) survey protocols and all materials, including the survey questionnaires, were cleared for ethics by Research Ethics Board, University of Waterloo, Canada (REB#40720). The **ITC Germany** (first cohort) survey protocols and all materials, including the survey questionnaires, were cleared for ethics by Research Ethics Board, University of Waterloo, Canada (REB#13858), and Heidelberg Medical School, Germany (IRB S-138/2007). The **EUREST-PLUS** (**ITC 6 European Country** in Germany (second cohort), Greece, Hungary, Poland, Romania, and Spain) survey protocols and all materials, including the survey questionnaires, were cleared for ethics by Research Ethics Board, University of Waterloo, Canada (REB#21262/30709), Comisia de Etica Iuliu Hatieganu University of Medicine and Pharmacy, Romania (IRB 155), Research Ethics Boards of the Bellvitge University Hospital, (IRB PR100/16), Committee and Dean of the Department of Health Care and Life Sciences, State College of Higher Vocational Education, Poland (IRB 1/2016), Research and Ethics Committee, Medical School, University of Athens, Greece (IRB 1516023880), Scientific and Research Committee, Medical Research Council, Hungary (IRB 11012-2-2016/EKU 0198/16, IRB 46344-2/2017/EKU), and Heidelberg Medical School, Germany (IRB 196/2016). The **ITC EUREST-PLUS Spain** (third wave) survey protocols and all materials, including the survey questionnaires, were cleared for ethics by Research Ethics Board, University of Waterloo, Canada (REB#41105), and Research Ethics Boards of the Bellvitge University Hospital, (IRB PR100/16, IRB PR248/17). The **ITC India** survey protocols and all materials, including the survey questionnaires, were cleared for ethics by Research Ethics Board, University of Waterloo, Canada (REB#132,97, REB#15722, REB#22140/31086), and Healis Sekhsaria Institute for Public Health (IRB 00007340). The **ITC Japan** survey protocols and all materials, including the survey questionnaires, were cleared for ethics by Research

For peer review only - http://bmjopen.bmj.com/site/about/guidelines.xhtml

Ethics Board, University of Waterloo, Canada (REB#22508/31428), National Cancer Center, Japan (IRB 2021-069), and Osaka International Cancer Institute (IRB 21054). The **ITC Kenya** survey protocols and all materials, including the survey questionnaires, were cleared for ethics by Research Ethics Board, University of Waterloo, Canada (REB#18311/30136), and University of Nairobi (IRB KNH-ERC/A/281). The **ITC Thailand** survey protocols and all materials, including the survey questionnaires, were cleared for ethics by Research Ethics Board, University of Waterloo, Canada (REB#11762, REB#15468), Roswell Park Comprehensive Cancer Center, US (NT-02-20), Cancer Council Victoria, Australia (HREC 0420), and Mahidol University, Thailand (IRB 0517·191/1003). The **ITC Malaysia** (first cohort) survey protocols and all materials, including the survey questionnaires, were cleared for ethics by Research Ethics Board, University of Waterloo, Canada (REB#11762, REB#15468), Roswell Park Comprehensive Cancer Center, US (NT-02-20), Cancer Council Victoria, Australia (HREC 0420), and Universiti Sains Malaysia, Malaysia (USM/PPSP/Ethics Com./2004(137·4[3])). The **ITC Malaysia** (second cohort) survey protocols and all materials, including the survey questionnaires, were cleared for ethics by Research Ethics Board, University of Waterloo, Canada (REB#40825) Medical Research Ethics Committee, University Malaya, Malaysia (MREC ID#2019118-8018). The **ITC Mauritius** survey protocols and all materials, including the survey questionnaires, were cleared for ethics by Research Ethics Board, University of Waterloo, Canada (REB#15487), and Mauritius Institute of Health, Mauritius (IRB MHC/CT/NETH/MIH). The **ITC Mexico** survey protocols and all materials, including the survey questionnaires, were cleared for ethics by Research Ethics Board, University of Waterloo, Canada (REB#13195, REB#13472, REB#16353), and Instituto Nacional de Salud Publica, Mexico (IRB 759). The **ITC Netherlands** (first cohort) survey protocols and all materials, including the survey questionnaires, were cleared for ethics by Research Ethics Board, University of Waterloo, Canada (REB#14666, REB#18920/30242). The **ITC Netherlands** (second cohort) survey protocols and all materials, including the survey questionnaires, were cleared for ethics by Research Ethics Board, University of Waterloo, Canada (REB#41704). The **ITC New Zealand** (first cohort) survey protocols and all materials, including the survey questionnaires, were cleared for ethics by Research Ethics Board, University of Waterloo, Canada (REB#13547), and Multi-Region Ethics Committee, New Zealand (MEC/06/07/071). The **ITC New Zealand (EASE: Evidence for Achieving Smokefree 2025 Equitably)** (second cohort) survey protocols and all materials, including the survey questionnaires, were cleared for ethics by Research Ethics Board, University of Waterloo, Canada (REB#21211/30726, REB#42549) and Human Ethics Committee, University of Otago (IRB 15/126, IRB 20/020). The **ITC Korea** (first cohort) survey protocols and all materials, including the survey questionnaires, were cleared for ethics by Research Ethics Board, University of Waterloo, Canada (REB#12547, REB#15017), and Internal Review Board, National Cancer Center, Republic of Korea (IRB NCCNCS-05-050, IRB NCCNCS-08-172, IRB NCCNCS-10-373), The **ITC Korea** (second cohort) survey protocols and all materials, including the survey questionnaires, were cleared for ethics by Research Ethics Board, University of Waterloo, Canada (REB#21287/30768) and Internal Review Board, National Cancer Center, Republic of Korea (IRB NCCNCS—2016-0129). The **ITC Korea** (third cohort) survey protocols and all materials, including the survey questionnaires, were cleared for ethics by Research Ethics Board, University of Waterloo, Canada (REB#41512) and Korea Health Promotion Institute (IRB KHPI 120160811107AN01-2004-HR-042-02). The **ITC Uruguay** survey protocols and all materials, including the survey questionnaires, were cleared for ethics by Research Ethics Board, University of Waterloo, Canada (REB#13195, REB#13472, REB#16665/30090) and Universidad de la República, Uruguay (IRB 071140-000649-08). The **ITC Zambia** survey protocols and all materials, including the survey questionnaires, were cleared for ethics by Research Ethics Board, University of Waterloo, Canada (REB#18086/303137), University of Zambia, Zambia (IRB 021-05-12). All secondary data analysis using ITC country data were cleared for ethics by Research Ethics Board, University of Waterloo, Canada (REB#31040). All participants provided consent for participating in the ITC surveys.

Funding statement:

The **ITC Four Country Surveys** (Australia, Canada, the United Kingdom, the United States) were supported by grants from the US National Cancer Institute (R01 CA100362, R01 CA090955, P50 CA111236 (Roswell Park Transdisciplinary Tobacco Use Research Center), and P01 CA138389), Robert Wood Johnson Foundation (045734), the Canadian Institutes of Health Research (FDN-148477, MOP-57897, MOP-79551, MOP-115016), the Canadian

For peer review only - http://bmjopen.bmj.com/site/about/guidelines.xhtml

Tobacco Control Research Initiative (014578), Australian Commonwealth Department of Health and Ageing, the National Health and Medical Research Council of Australia (265903, 450110, 1005922), and Cancer Research UK (C312/A3726, C312/A6465, C321/A11039, C25586/A19540). The **ITC Four Country Smoking and Vaping Surveys** (Australia, Canada, England, and the United States) were supported by grants from the US National Cancer Institute (P01 CA200512), the Canadian Institutes of Health Research (FDN-148477), and the National Health and Medical Research Council of Australia (APP1106451). The **ITC Bangladesh Surveys** were supported by grants from the US National Cancer Institute (P01 CA138389), the International Development Research Centre (104831-003), and Canadian Institutes for Health Research (MOP-79551, MOP-115016). The **ITC Brazil Surveys** were supported by the Instituto Nacional de Câncer (INCA, National Cancer Institute), Secretaria Nacional de Pokiticas sobre Drogas (SENAD, National Secretariat for Drug Policy), Pan-American Health Organization (PAHO), Brazil Fundação do Câncer (BR/LOA/1500048·00), and the Canadian Institutes of Health Research (MOP-115016, FDN-148477). The **ITC China Surveys** were supported by grants from the US National Cancer Institute at the National Institutes of Health (R01 CA125116), P50 CA111236 (Roswell Park Transdisciplinary Tobacco Use Research Center), Canadian Institutes for Health Research (MOP-79551, MOP-115016), and the Chinese Center for Disease Control and Prevention. The **ITC France Surveys** (first cohort) were supported by grants from the Observatoire français des drogues et toxicomanies (OFDT), Institut national de prevention et d'education pour la sante (INPES), and the Institut nationale du cancer (INCa). The ITC France Surveys (second cohort) were supported by grants from Santé publique France, the Institut nationale du cancer (INCa), and the Canadian Institutes of Health Research (FDN-148477). The **ITC Germany Surveys** (first cohort) were supported by grants from the German Federal Ministry of Health, Dieter Mennekes-Umweltstiftung, and Germany Cancer Research Center (DKFZ). The EUREST-PLUS Project (ITC 6 European Country Surveys) in Germany (second cohort), Greece, Hungary, Poland, Romania, and Spain has received funding from the European Union's Horizon2020 research and innovation programme 681109, and additional support was provided to the University of Waterloo by the Canadian Institutes of Health Research (FDN-148477). EF is partly supported by Ministry of Universities and Research, Government of Catalonia (2017SGR319) and by the Instituto de Salud Carlos III and co-funded by the European Regional Development Fund (INT16/00211 and INT17/00103), Government of Spain, and CERCA Programme Generalitat de Catalunya for the institutional support to IDIBELL. The EUREST-PLUS Spain Survey was supported by grants from the Instituto de Salud Carlos III (PI17/01338, Co-funded by European Regional Development Fund and the Canadian Institutes for Health Research (FDN-148477). EF and MF are partly supported by the Ministry of Universities and Research, Government of Catalonia (2021SGR00906) ,and CERCA Programme Generalitat de Catalunya for the institutional support to IDIBELL. The **ITC India Surveys** were supported by grants from the US National Cancer Institute (P50 CA111236, P01 CA138389), the Canadian Institutes of Health Research (MOP-79551, MOP-115016, FDN-148477), and Healis Sekhsaria Institute for Public Health. The **ITC Japan Surveys** were supported by the Japan National Cancer Center and Research Development Fund (28-A-24) and the Canadian Institutes of Health Research (FDN-148477). The **ITC Kenya Surveys** were supported by grants from the Canadian Institutes of Health Research (MOP-115016, FDN-148477). The **ITC Thailand Surveys** were supported by grants from the US National Cancer Institute (P50 CA111236, P01 CA138389 (Roswell Park Transdisciplinary Tobacco Use Research Center), the Canadian Institutes of Health Research (MOP-79551, MOP-115016), and the ThaiHealth Promotion Foundation. The ITC Malaysia Surveys (first cohort) were supported by grants from the US National Cancer Institute (P50 CA111236 (Roswell Park Transdisciplinary Tobacco Use Research Center) and P01 CA138389), the Canadian Institutes of Health Research (MOP-79551, MOP-115016), and the Malaysian Ministry of Health. The **ITC Malaysia Surveys** (second cohort) were supported by the Malaysia Ministry of Higher Education-LRGS NanoMITe (RU029-2014), the University of Malaya Research University Grant (RU029C-2014 and RU001A-2021), and the Canadian Institutes of Health Research (FDN-148477). The **ITC Mauritius Surveys** were supported by grants from the International Development Research Centre, the Canadian Institutes of Health Research (MOP-115016), and was partially supported by the World Lung Foundation. The **ITC Mexico Surveys** were supported by grants from the Mexican Consejo Nacional de Ciencia y Tecnologia (Salud-2007-C01-70032, Salud-2013-01-202671), Bloomberg Global Initiative—International Union Against Tuberculosis and Lung Disease, the US National Cancer Institute (P50 CA111236 (Roswell Park Transdisciplinary Tobacco Use Research Center) and the Canadian Institutes for Health Research (MOP-115016). The **ITC Netherlands Surveys** (first cohort) were supported by grants from The Netherlands Organisation for Health Research and Development (ZonMw 121010008, ZonMw 200130002), The SILNE Project (funded by the European Commission through FP7 HEALTH-F3-2011-278273) and the Dutch Cancer Foundation (KWF) (UM 2014-7210). The ITC

For peer review only - http://bmjopen.bmj.com/site/about/guidelines.xhtml

Netherlands Surveys (second cohort) were supported by grants from Longfonds (Lung Foundation Netherlands), Hartstichting (Netherlands Heart Foundation), KWF Kankerbestrijding (Dutch Cancer Society), Trombosestichting Nederland (Thrombosis Foundation), Diabetesfonds (Diabetes Fund) and the Canadian Institutes of Health Research (FDN-148477). The **ITC New Zealand Surveys** (first cohort) were supported by grants from the New Zealand Health Research Council (06/453), the New Zealand Ministry of Health, University of Otago, the US National Cancer Institute (P50 CA111236 (Roswell Park Transdisciplinary Tobacco Use Research Centre)), and the Canadian Institutes of Health Research (MOP-79551). The ITC New Zealand (EASE: Evidence for Achieving Smokefree 2025 Equitably) Surveys (second cohort) were supported by grants from the New Zealand Health Research Council (GA215F 15/072, HRC 19/641), the New Zealand Ministry of Health, and the Canadian Institutes for Health Research (FDN-148477). The **ITC Korea Surveys** (first cohort) were supported by grants from the National Cancer Center of Korea (0731040-1), the Korean Ministry of Health and Welfare, and the US National Cancer Institute (P50 CA111236, R01 CA125116) and the Canadian Institutes of Health Research (MOP-79551). The ITC Korea Survey (second cohort) was supported by a grant from the Korean Ministry of Health and Welfare and the Canadian Institutes of Health Research (MOP-115016). The ITC Korea Surveys (third cohort) were supported by a grant from the Korean Ministry of Health and Welfare and the Canadian Institutes of Health Research (FDN-148477). The **ITC Uruguay Surveys** were supported by grants from the US National Cancer Institute (P50 CA111236 (Roswell Park Transdisciplinary Tobacco Use Research Center), P01 CA138389), and the Canadian Institutes of Health Research (MOP-115016). The ITC Zambia Surveys were supported by a grant from the Canadian Institutes of Health Research (MOP-115016). Additional support provided by the Ontario Institute for Cancer Research (Senior Investigator Grant to GTF (2002-present)), and the Canadian Cancer Society Research Institute (Senior Prevention Scientist Award to GTF (2002-09), and O. Harold Warwick Prize to GTF (2021). Financial support for **GATS** was provided by the CDC Foundation with grants from Bloomberg Philanthropies and the Bill and Melinda Gates Foundation, and/or in-country donor funding for GATS countries listed in Supplemental file 1B. The funders had no role in the design of the study; in the collection, analyses, or interpretation of data; in the writing of the manuscript, or in the decision to publish the results.

Competing interest statement:

KMC has in the past and continues to serve as a paid witness in litigation filed against cigarette manufacturers. GTF has been an expert witness or consultant for governments defending their country's policies or regulations in litigation. CG has been a paid expert witness for the Australian Government in litigation concerning nicotine product marketing. KK received a JMWH Bayer Grant from Japan Society for Menopause and Women's Health. ASAN has received an unconditional educational grant from Johnson & Johnson Malaysia Sdn. Bhd. JFT has served as paid expert witness in legal challenges against tobacco and vaping companies. All other authors have no conflicts of interest to declare.

Acknowledgements: The ITC authors would like to acknowledge and thank all ITC team members at the University of Waterloo, Canada and the ITC country teams for their contributions: **ITC Four Country** and **ITC Four Country Smoking and Vaping** teams at Roswell Park Comprehensive Cancer Center, US, King's College London, UK, and Cancer Council Victoria, University of Melbourne, and The Queensland University, Australia; **ITC Brazil** teams at the Brazilian National Cancer Institute (INCA), Alliance for the Control of Tobacco Use (ACTbr), the Federal University of Rio de Janeiro, the Oswaldo Cruz Foundation, and the International Union against Tuberculosis and Lung Disease (IUATLD); **ITC Bangladesh** teams at the Bureau of Economic Research and the Department of Economics, University of Dhaka; **ITC China** teams at the China CDC and the local CDCs in Beijing, Changsha, Changzhi, Guangzhou, Huzhou, Shanghai, Shenyang, Tongren, Yichun, Yinchuan, Xining, and Yunnan Health Education Institute at Kunming; **ITC France** (first cohort) team at l'Institut national de prevention et d'éducation pour la santé (INPES), l'Observatoire françaisdes drogues et des toxicomanies (OFDT) and l'Institut national du cancer (INCa); **ITC France** (second cohort) team at Santé publique France and l'Institut national du cancer (INCa); **ITC Germany** (first cohort) team at Deutsches Krebsforschungszentrum (DKFZ; German Cancer Research Center), Bundesministerium für Gesundheit (German Federal Ministry of Health), and Dieter Mennekes-Umweltstiftung; **ITC India** teams at Bihar--School of Preventive Oncology, West Bengal--Cancer Foundation of India, Madhya Pradesh--Madhya Pradesh Voluntary Health Association, and Maharashtra—Healis-Sekhsaria Institute for Public Health; **ITC Kenya** team at Population Studies and Research Institute (PSRI), University of Nairobi; **ITC Korea** (first cohort) teams

For peer review only - http://bmjopen.bmj.com/site/about/guidelines.xhtml

at National Cancer Center, Asan Medical Center, Sungjyunjwan University College of Medicine, Ewha Womans University School of Medicine, Dankook University, Korean Cell Line Bank and Cancer Research Center, Hallym University Sacred Heart Hospital; **ITC Korea** (second cohort) teams at National Cancer Center and Seoul National University; **ITC Korea** (third cohort) teams at Korea Health Promotion Institute, National Cancer Center, Korea Center for National Tobacco Control Research & Education, and Seoul National University; **ITC Malaysia** (first cohort) teams at the National Poison Centre, Universiti Sains Malaysia and the Malaysian Ministry of Health; **ITC Malaysia** (second cohort) team at University Malaya; **ITC Mauritius** teams at Mauritius Institute of Health and ViSa; **ITC Mexico** team at the Mexican National Institute of Public Health (INSP); **ITC Netherlands (first cohort)** teams at Maastricht University, University of Amsterdam, and Leiden University Medical Centre); **ITC Netherlands (second cohort)** teams at Maastricht University and University of Amsterdam (ASCoR); **ITC New Zealand** (first and second cohorts) team at University of Otago; **ITC Thailand** team at the Institute for Population and Social Research (IPSR), Mahidol University Salaya; **ITC Uruguay** team at the Departamento de Sociología, the Facultad de Ciencias Sociales of the Universidad de la República de Uruguay and the Sindicato Médico del Uruguay**; ITC Zambia** team at University of Zambia School of Medicine; **ITC EUREST-PLUS Spain** team at Tobacco Control Unit, Catalan Institute of Oncology (ICO) and Bellvitge Biomedical Research Institute (IDIBELL); and **the EUREST-PLUS (ITC 6 European Country)** consortium members: European Network on Smoking and Tobacco Prevention (ENSP), Belgium: Constantine I. Vardavas, Andrea Glahn, Christina N. Kyriakos, Dominick Nguyen, Katerina Nikitara, Cornel Radu-Loghin and Polina Starchenko; University of Crete (UOC), **Greece**: Aristidis Tsatsakis, Charis Girvalaki, Chryssi Igoumenaki, Sophia Papadakis, Aikaterini Papathanasaki, Manolis Tzatzarakis and Alexander I. Vardavas; Kantar Public, Belgium: Nicolas Bécuwe, Lavinia Deaconu, Sophie Goudet, Christopher Hanley and Oscar Rivière; Smoking or Health Hungarian Foundation (SHHF), **Hungary**: Tibor Demjén, Judit Kiss and Anna Piroska Kovacs; Tobacco Control Unit, Catalan Institute of Oncology (ICO) and Bellvitge Biomedical Research Institute (IDIBELL), Catalonia, **Spain**: Esteve Fernández, Yolanda Castellano, Marcela Fu, Sarah O. Nogueira and Olena Tigova; Kings College London (KCL), **United Kingdom**: Ann McNeill, Katherine East and Sara C. Hitchman; Cancer Prevention Unit and WHO Collaborating Centre for Tobacco Control, German Cancer Research Center (DKFZ), **Germany** (second cohort): Ute Mons and Sarah Kahnert; National and Kapodistrian University of Athens (UoA), **Greece**: Yannis Tountas, Panagiotis Behrakis, Filippos T. Filippidis, Christina Gratziou, Paraskevi Katsaounou, Theodosia Peleki, Ioanna Petroulia and Chara Tzavara; Aer Pur Romania, **Romania**: Antigona Carmen Trofor, Marius Eremia, Lucia Lotrean and Florin Mihaltan; European Respiratory Society (ERS), Switzerland: Gernot Rohde, Tamaki Asano, Claudia Cichon, Amy Far, Céline Genton, Melanie Jessner, Linnea Hedman, Christer Janson, Ann Lindberg, Beth Maguire, Sofia Ravara, Valérie Vaccaro and Brian Ward; Maastricht University, the Netherlands: Marc Willemsen, Hein de Vries, Karin Hummel and Gera E. Nagelhout; Health Promotion Foundation (HPF), **Poland**: Witold A. Zatoński, Aleksandra Herbeć, Kinga Janik-Koncewicz, Krzysztof Przewoźniak and Mateusz Zatoński. University of Waterloo (UW), Canada: Geoffrey T. Fong, Thomas K. Agar, Pete Driezen, Shannon Gravely, Anne C. K. Quah and Mary E. Thompson.

ITC Project's Data Availability Statement: In each country participating in the international Tobacco Control Policy Evaluation (ITC) Project, the data are jointly owned by the lead researcher(s) in that country and the ITC Project at the University of Waterloo. Data from the ITC Project are available to approved researchers 2 years after the date of issuance of cleaned data sets by the ITC Data Management Centre. Researchers interested in using ITC data are required to apply for approval by submitting an International Tobacco Control Data Repository (ITCDR) request application and subsequently to sign an ITCDR Data Usage Agreement. The criteria for data usage approval and the contents of the Data Usage Agreement are described online (http://www.itcproject.org).

For peer review only - http://bmjopen.bmj.com/site/about/guidelines.xhtml

Supplementary file 1B. GATS funding statements

Financial support for GATS was provided by the CDC Foundation with grants from Bloomberg Philanthropies and the Bill and Melinda Gates Foundation. Details are provided in Supplemental Table 1.

Supplemental Table 1. Global Adult Tobacco Survey (GATS) donor funding in 32 countries included in analysis, 2008-2020.

Country	Wave	Year	GATS Funding
Argentina	1	2012	Bloomberg Philanthropies
Bangladesh	1	2009	Bloomberg Philanthropies
	2	2017	Bloomberg Philanthropies
Botswana	1	2017	Bill and Melinda Gates Foundation
Brazil	1	2008	56 % Bloomberg Philanthropies<br>44% In-country funding
Cameroon	1	2013	Bill and Melinda Gates Foundation
China	1	2010	Bill and Melinda Gates Foundation
	2	2018	69% Bloomberg Philanthropies<br>31% In-country funding
Costa Rica	1	2015	In-country funding
Egypt	1	2009	Bloomberg Philanthropies
Ethiopia	1	2016	Bill and Melinda Gates Foundation
Greece	1	2013	In-country funding
India	1	2009	36% Bloomberg<br>67% In-country funding
	2	2017	33% Bloomberg Philanthropies<br>67% In-country funding
Indonesia	1	2011	Bloomberg Philanthropies
Kazakhstan	1	2014	Bloomberg Philanthropies
Kenya	1	2014	Bill and Melinda Gates Foundation
Malaysia	1	2011	60% Bloomberg Philanthropies<br>40% In-country funding
Mexico	1	2009	Bloomberg Philanthropies
	2	2015	76% Bloomberg Philanthropies<br>24% In-country funding
Nigeria	1	2012	Bloomberg Philanthropies
Pakistan	1	2014	Bloomberg Philanthropies
Panama	1	2013	In-country funding
Philippines	1	2009	Bloomberg Philanthropies
	2	2015	In- country funding
Poland	1	2009	Bloomberg Philanthropies
Qatar	1	2013	In-country funding
Romania	1	2011	Bloomberg Philanthropies
	2	2018	Bloomberg Philanthropies
Russian Federation	1	2009	Bloomberg Philanthropies
	2	2016	70% Bloomberg Philanthropies<br>30% In-country funding
Senegal	1	2015	Bill and Melinda Gates Foundation

For peer review only - http://bmjopen.bmj.com/site/about/guidelines.xhtml

Country	Wave	Year	GATS Funding
Thailand	1	2009	Bloomberg Philanthropies
	2	2011	73% Bloomberg Philanthropies<br>27% In-country funding
Turkey	1	2008	Bloomberg Philanthropies
	2	2012	Bloomberg Philanthropies
	3	2016	6% Bloomberg Philanthropies<br>94% In-country funding
Uganda	1	2013	Bill and Melinda Gates Foundation
Ukraine	1	2010	Bloomberg Philanthropies
	2	2017	Bloomberg Philanthropies
United Republic of Tanzania	1	2018	Bill and Melinda Gates Foundation
Uruguay	1	2009	Bloomberg Philanthropies
	2	2017	52% Bloomberg Philanthropies<br>48% In-country funding
Viet Nam	1	2010	Bloomberg Philanthropies
	2	2015	92% Bill and Melinda Gates<br>8% In-country funding

For peer review only

For peer review only - http://bmjopen.bmj.com/site/about/guidelines.xhtml
