# Supplementary material for: Changes in knowledge of the major harms of tobacco smoking and secondhand smoke exposure among adults who smoke: findings from 26 countries of the International Tobacco Control (ITC) Project (2002–2022) and 32 countries of the Global Adult Tobacco Survey (GATS) (2008–2020)
Source: BMJ Open. 2026 Aug 4;16(8):e107667. doi: 10.1136/bmjopen-2025-107667 (PMC13479502; doi:10.1136/bmjopen-2025-107667)
Supplement: Supplementary file 3 [file bmjopen-2025-107667.reviewer_comments.pdf]

PEER REVIEW HISTORY

BMJ Open publishes all reviews undertaken for accepted manuscripts. Reviewers are asked to complete a checklist review form and are provided with free text boxes to elaborate on their assessment. These free text comments are reproduced below.

ARTICLE DETAILS

Title (Provisional)

Changes in knowledge about major harms of tobacco smoking and secondhand smoke exposure among adults who smoke: findings from 26 countries of the International Tobacco Control (ITC) Project (2002–2022) and 32 countries of the Global Adult Tobacco Survey (GATS) (2008–2020)

Authors

Chung-Hall, Janet; Fong, Geoffrey T.; Meng, Gang; Craig, Lorraine V; Indome, Eunice O; Collaborators, for the ITC Project; Kress, Alissa C; Shi, Jing; Ahluwalia, Indu B.

VERSION 1 - REVIEW

**Reviewer**	**1**
**Name**	**Rábade-Castedo, Carlos**
**Affiliation**	**Hospital Clínico Universitario de Santiago Servizo de Pneumoloxía**
**Date**	**17-Sep-2025**
**COI**	**None**

This is an interesting study on the knowledge of populations in different countries with different characteristics regarding the causality of smoking and passive smoking exposure and tobacco-related diseases.
It provides valuable information for establishing strategies that give the population a greater awareness of the negative consequences of smoking.
The introduction and the stated objectives are appropriate.
The methodology used to conduct this study is correct.
Results:
Regarding the results presented in this manuscript, I would like to make the following observations:
This study should provide data on the population's awareness of the consequences of smoking not only on cancer and cardiovascular diseases, but also on respiratory diseases such as COPD (one of the leading causes of death in the developed world).
On the other hand, it would be interesting to include the knowledge of the inhabitants of different countries about new tobacco and nicotine products (e-cigarettes, heated tobacco, nicotine pouches, etc.) and the health risks these products pose. The increase in these new

forms of tobacco around the world makes it necessary to know whether the population is informed about the health risks they pose.
Discussion:
Although explained in the discussion, it is striking that smoking control policies in different countries do not seem to influence the population's knowledge about the causality of smoking and passive exposure to tobacco smoke and the harm caused. There should be a correlation between knowledge of the harms of passive exposure to tobacco smoke and populations with more smoke-free environments. Likewise, there should be a correlation between plain packaging or increased advertising about the harmful effects of tobacco and greater awareness of it. I believe this should be better justified in the discussion section. Another interesting reflection to discuss is how the reduction in the number of cases of such harmful diseases as those analyzed (cancer, stroke, ischemic heart disease) could influence those countries with a reduction in smoking prevalence and the perception of the harm caused by these diseases. Likewise, the healthcare systems of countries with more resources, through new treatments for these smoking-related diseases, reduce mortality and improve the quality of life of these patients (new cancer treatments, fibrinolysis for stroke, angioplasty, etc.). This could minimize the harm caused by these tobacco-related diseases in the absence of smoking prevention policies. That is, the use of public resources to treat tobacco-related diseases and not prevent their onset by treating smoking from the moment smoking begins will generate a lower perception of the harm caused by these diseases in the population and a lower awareness of their consequences.

**Reviewer**	**2**
**Name**	**Mantey, Dale S.**
**Affiliation**	**UTHlth Sch Publ Hlth**
**Date**	**10-Oct-2025**
**COI**	**None**

Title: Changes in knowledge about major harms of tobacco smoking and secondhand smoke exposure among adults who smoke findings from 26 countries (ITC) and 32 GATS

General Comments: This is an interesting paper on an important topic. Several strengths including two large datasets which are analyzed together in a valid methodology. The major limitations of this study are in the background and discussion. These sections are largely surface level information. The authors do touch on each of the relevant topics but I would urge them to discuss them further to provide better context of the research question in the introduction and interpretation of the results in the discussion.

Abstract: This section is clear. No comments.

Introduction: This section introduces important moving parts but each of them needs to be discussed in further detail. Specific suggestions are provided below.

1. The first paragraph has a sound foundation but could be clarified. First, please draw a better distinction between firsthand and secondhand smoking in the second and third

sentences. Second, I would merge the third paragraph (“Despite efforts to inform…”) with the first paragraph to give a comprehensive introduction to the knowledge construct.

2. The section on risk perceptions of SHS relies are data that are more than 20 years old. I would strongly urge the authors to cite newer data.

3. I would also suggest the authors discuss context and determinants of SHS. There are two inherent factors in the discussion of SHS. First is the consideration of other people. The most common one there is usually family, particularly children. I would recommend the authors discuss things like household rules around smoking indoors and how factors (e.g., children; child age; etc) may influence that. For example, knowledge of SHS may mean more when someone is thinking about protecting their child.

4. Second, the workplace is a major source of SHS. As smoke-free policies are enacted, building knowledge about the dangers of SHS typically follows in order to build and sustain public support. The authors should discuss any sort of policy or public messaging that may be influencing SHS knowledge. This ties into the second paragraph (“The WHO Framework…”) but the authors should give a broader explanation of policy and SHS knowledge.

5. When discussing the “W” within MPOWER, the authors should also describe a theoretical framework through which raising awareness can change behavior and perceptions. As it is stated, “knowledge alone does not change behavior” which means there are other mechanisms at play. The authors should use a theoretical framework to describe these mechanisms. I would recommend Social Cognitive Theory (SCT) as this is the most used in tobacco prevention and control programs, however, the authors are free to select whichever theory works best for them. Sample citation is provided below

a. Mantey DS, et al. (2024). Social cognitive theory and health behaviors. Health Behavior: Theory, Research, and Practice, 98.

Methods: This section is strong and boosted by the supplemental materials. Minor comments and questions below.

6. The combination of “no” and “don’t know” may cause bias. Did the authors consider sensitivity analyses that excluded “don’t know” similar to the “refused” response? Alternatively, could the authors provide supplemental information on what proportion of the sample was in the “don’t know” category?

7. The measures should be better described. For example, the smoking status variable seems to code non-smokers with smokers who do not smoke daily. This should be made clearer.

Results: This section is dense and could be either shortened or better organized.

8. The authors provide most of their data in this section which is typically a strength. However, the country specific data creates a large number of results to report. I would urge the authors to either limit to the most compelling results to highlight here or, alternatively,

to organize the results into smaller paragraphs in which they report similar outcomes and directions.

Discussion: This section is off to a good start but could be improved with more thorough interpretation of results.

9. The authors touch on important implications and interpretations but do not go far enough. For example, the discussion of country-level differences in education strategies does not speak to the most common form of health education (i.e., school-based programs for kids) which may explain some of the changes seen. A recent study (cited below) found that school-based interventions corresponded with a 69% increase in SHS harm perceptions and 70% increase in support of smoke-free policies.

a. Chido-Amajuoyi OG, et al. (2024). Exposure to school-based tobacco prevention interventions in low-income and middle-income countries and its association with psychosocial predictors of smoking among adolescents: a pooled cross-sectional analysis of Global Youth Tobacco Survey data from 38 countries. BMJ open, 14(2), e070749.

10. The limitations are robust. The authors should be given credit for this.

11. The authors should call back to the WHO MPOWER framework from the introduction. Speak more to the necessity and the procedures through which to achieve the W piece.

**Reviewer**	**3**
**Name**	**Kelson, Zoe**
**Affiliation**	**University of Exeter, Mathematics**
**Date**	**18-Dec-2025**
**COI**	**None**

This study aims to assess levels of and trends in knowledge that smoking and secondhand smoke (SHS) causes lung cancer and cardiovascular disease (CVD) in 46 countries over two decades.

Reviewer comments:

"Nationally representative surveys in 26 International Tobacco Control (ITC) countries (2002-2022), and 32 Global Adult Tobacco Survey (GATS) countries (2008- 2020)...

Adults (18+ years) who smoke tobacco (26 ITC countries: N=133,410; 32 GATS countries: N=110,537)." [Abstract]

and

"ITC Project longitudinal cohort surveys with replenishment are administered in 31 countries using a standardised set of questions, data collection protocol, and sampling design that allows for cross-country comparisons."

and

"GATS is a nationally representative repeated cross-sectional household survey of persons aged 15+ years that uses a standard core questionnaire, sample design, and data collection methods."

Can the survey design please be described in more detail?

Have sample weights been applied to account for survey designs?

Can the authors please comment on the potential impact of COVID-19 on the survey data?

"We used data from all survey waves to test for significant yearly change in knowledge, and data from the first and last survey waves to compute average yearly change in knowledge"

Can the authors please confirm whether survey methods have stayed consistent over time?

"Sample demographics and methodological details for ITC Surveys are described elsewhere[18] and in online individual country technical reports"

Can the sample demographics please be reported here, perhaps in the supplementary material?

Can the authors please confirm if there was any missing data?

"Generalized estimating equations and weighted multinomial regression models assessed country-level prevalence of knowledge for three smoking and two SHS outcomes. Multilevel models estimated all country mean prevalence and tested within country linear trends over time for five disease indicators by survey. Across 12 countries, with data for both surveys, correlations between ITC and GATS assessed consistency across indicators." [Abstract]

and

"Point estimates of the prevalence of knowledge for each disease outcome by country were calculated using weighted multinomial survey logistic regression models for pooled repeated cross-sectional GATS data, and generalized estimating equations for pooled longitudinal ITC data. All models incorporated survey design information (strata and primary sampling units for multi-stage sampling surveys), adjusted for sex, age, smoking status (daily, non-daily), and time-in-sample (ITC countries). Point estimates of all-country means of the prevalence of knowledge for each disease outcome across all ITC countries and all GATS countries, respectively, were calculated using a multi-level model (two levels: individual and country) with random intercepts at the country-level and with roughly equal weights for each country. "

and

"Analysis of the longitudinal ITC data used generalized estimating equations to account for within-subject correlations given that a proportion of individuals participated in more than one survey. Analysis of the repeated cross-sectional GATS data used weighted multinomial survey logistic regression models. Two-level models (individual and country) with random

intercepts and random slopes of survey years at the country-level were used to test for significant yearly change in knowledge for each health effect within countries."

Appropriate modelling methods have been applied, which are summarised concisely in the Abstract.

Was a trend analysis such as joinpoint regression analyses considered by the authors?

"As such, we report the estimated all-country means of knowledge for each health effect without the associated confidence intervals, which would refer to a hypothetical superpopulation of countries."

Can the authors please clarify whether pooled supercountry estimates account for heterogeneity between countries (e.g. weighted by different sample sizes proportional to country population size)?

"Pearson correlations (r) between ITC and GATS mean prevalence estimates of knowledge for each of the five diseases were used to examine consistency of estimates from different surveys conducted in the 12 overlapping countries. "

Were normality and linearity assumptions checked prior to the application of Pearson correlation?

"Knowledge about diseases caused by smoking among adults who smoke was highest for lung cancer (ITC=92.2%, GATS=91.9%), followed by heart disease (ITC=84.7%)/heart attack (GATS=80.0%), and stroke (ITC=75.0%, GATS=66.1%) (Supplementary table 5)...."

Can confidence intervals please be generated for all point estimates, and communicated throughout the Results?

The Figures are clear and informative.

"Use of self-reported data may be subject to measurement bias. • Generalizability of findings is limited by low response rates for some ITC surveys and the restriction of ITC-GATS correlation analyses to 12 countries. " [Strengths and Limitations of this Study]

and

"This study has limitations..."

A thorough discussion on the study limitations is provided by the authors.

Thanks for providing a copy of the STROBE checklist.

VERSION 1 - AUTHOR RESPONSE

Reviewer 1's comments:

1. **This is an interesting study on the knowledge of populations in different countries with different characteristics regarding the causality of smoking and passive smoking exposure and tobacco-related diseases. It provides**

valuable information for establishing strategies that give the population a greater awareness of the negative consequences of smoking. The introduction and the stated objectives are appropriate. The methodology used to conduct this study is correct.

Authors' response: Thank you for the positive feedback.

2. **Results: Regarding the results presented in this manuscript, I would like to make the following observations: This study should provide data on the population's awareness of the consequences of smoking not only on cancer and cardiovascular diseases, but also on respiratory diseases such as COPD (one of the leading causes of death in the developed world).**

   Authors' response: We thank the reviewer for this suggestion. We agree that respiratory diseases, such as COPD, represent an important health burden. However, our study focuses on knowledge that smoking and SHS causes lung cancer and CVD because these are outcomes for which comparable longitudinal data are available in both the ITC Surveys and GATS at all waves and all countries; there are other health outcomes/diseases that have been asked about in the ITC surveys, but not uniformly across countries and waves, and these do not overlap with the GATS.

   We thank the reviewer for reaffirming our objective of calling attention to the lower awareness that smoking causes cancer and CVDs. We have added text to the Discussion to emphasize that CVD is the leading cause of smoking-attributable deaths, and that our study extends prior research showing that adults who smoke have higher knowledge that smoking causes lung cancer than CVD.

   If we had data on the population's awareness of COPD, we certainly would have included the findings for that major cause of death from smoking.

3. **On the other hand, it would be interesting to include the knowledge of the inhabitants of different countries about new tobacco and nicotine products (e-cigarettes, heated tobacco, nicotine pouches, etc.) and the health risks these products pose. The increase in these new forms of tobacco around the world makes it necessary to know whether the population is informed about the health risks they pose.**

   Authors' response: We agree that examining knowledge of the health risks of new tobacco and nicotine products is important, but this is beyond the scope of the current study, which focuses on smoked tobacco products (especially given the severe length constraints of articles). That has been and will be the focus of papers from the ITC Project (we are less clear about the availability of appropriate data on perceptions about the harmfulness of e-cigarettes, heated tobacco products, nicotine pouches, etc.).

   We have, however, added a brief summary of key expert recommendations for strengthening FCTC implementation after the last two decades, which

includes the application of FCTC regulations to new tobacco products (citation: Gilmore AB, Callard C, Sy D, *et al.* 20th anniversary of the WHO Framework Convention on Tobacco Control coming into force: time for a step change in ambition. *The Lancet*. 2025;405:677–81. doi: 10.1016/S0140-6736(25)00336-8).

4. **Discussion: Although explained in the discussion, it is striking that smoking control policies in different countries do not seem to influence the population's knowledge about the causality of smoking and passive exposure to tobacco smoke and the harm caused. There should be a correlation between knowledge of the harms of passive exposure to tobacco smoke and populations with more smoke-free environments. Likewise, there should be a correlation between plain packaging or increased advertising about the harmful effects of tobacco and greater awareness of it. I believe this should be better justified in the discussion section.**

   Authors' response: We agree that, in principle, strong policies should be associated with greater population knowledge of harms caused by smoking and SHS. However, the association between smoke-free laws and population knowledge could depend on effective policy implementation and enforcement. That is, poor enforcement of smoke-free laws may undermine policy impact, which may then limit their impact on population-level knowledge. There is also evidence suggesting that smoke-free laws are more likely to be effective for increasing population-level knowledge when implemented alongside other interventions, such as mass media campaigns that are especially effective for improving knowledge and reducing exposure to SHS. We have added text in the Discussion to clarify these points. We also agree that stronger "W" policies, namely health warnings and mass media campaigns, are positively associated with awareness of tobacco-related harms. We have expanded the Discussion to clarify that our observed country-level differences may reflect variation in the content and strength (text vs pictorial) of health warnings and mass media campaigns in place at the time of the surveys.

5. **Another interesting reflection to discuss is how the reduction in the number of cases of such harmful diseases as those analyzed (cancer, stroke, ischemic heart disease) could influence those countries with a reduction in smoking prevalence and the perception of the harm caused by these diseases. Likewise, the healthcare systems of countries with more resources, through new treatments for these smoking-related diseases, reduce mortality and improve the quality of life of these patients (new cancer treatments, fibrinolysis for stroke, angioplasty, etc.). This could minimize the harm caused by these tobacco-related diseases in the absence of smoking prevention policies. That is, the use of public resources to treat tobacco-related diseases and not prevent their onset by treating smoking from the moment smoking begins will generate a lower perception of the harm caused by these diseases in the population and a lower awareness of their consequences.**

Authors' response: We thank the reviewer for the suggestion and we have added related text to the Discussion section.

Reviewer 2's comments:

1. **General Comments: This is an interesting paper on an important topic. Several strengths including two large datasets which are analyzed together in a valid methodology. The major limitations of this study are in the background and discussion. These sections are largely surface level information. The authors do touch on each of the relevant topics but I would urge them to discuss them further to provide better context of the research question in the introduction and interpretation of the results in the discussion**.

   Authors' response: Thank you for the acknowledgement of the strengths of our study. We have revised the Introduction and Discussion sections – please refer to our detailed responses below. We would like to note that the revisions made in response to the Reviewer's suggestions has added considerable length to the manuscript.

2. **Abstract: This section is clear. No comments.**

3. **Introduction: This section introduces important moving parts but each of them needs to be discussed in further detail. Specific suggestions are provided below. 1. The first paragraph has a sound foundation but could be clarified. First, please draw a better distinction between firsthand and secondhand smoking in the second and third sentences. Second, I would merge the third paragraph ("Despite efforts to inform…") with the first paragraph to give a comprehensive introduction to the knowledge construct.**

   Authors' response: We have made the suggested revisions to the Introduction.

4. **The section on risk perceptions of SHS relies are data that are more than 20 years old. I would strongly urge the authors to cite newer data.**

   Authors' response: A key contribution, we believe, that our paper makes is to examine trends in knowledge about diseases caused by smoking and SHS over 20 years. Thus, a focus in our literature review was on other studies that had examined **changes in knowledge over time.**

   To our knowledge, studies of changes in knowledge for smoking- and SHS-caused diseases are limited to the two studies that are cited in the Introduction. Our study thus fills a critical gap in the literature by providing the most comprehensive multi-country assessment to date of trends in knowledge about diseases caused by smoking and SHS. Specifically, our study examines changes in knowledge in 46 countries across two decades using data from two major global surveillance systems ─the International Tobacco Control (ITC) Policy Evaluation Project (2002-2022) and the Global Adult Tobacco

Survey (GATS) (2008-2020). We assessed knowledge of key diseases caused by smoking (lung cancer, heart disease/heart attack, and stroke) in people who smoke, and diseases caused by exposure to SHS (lung cancer and heart disease/heart attack) in people who do not smoke. We also found strong agreement between the ITC and GATS measures in those countries where the two surveys had been conducted, strengthening the findings of our study.

We agree with the reviewer regarding the importance of citing more recent studies on SHS risk perceptions. Accordingly, we have revised the Introduction to include a summary of recent single-country cross-sectional studies on risk perceptions of SHS. This contextualizes our study within the current literature while emphasizing the lack of longitudinal evidence.

5. **I would also suggest the authors discuss context and determinants of SHS. There are two inherent factors in the discussion of SHS. First is the consideration of other people. The most common one there is usually family, particularly children. I would recommend the authors discuss things like household rules around smoking indoors and how factors (e.g., children; child age; etc) may influence that. For example, knowledge of SHS may mean more when someone is thinking about protecting their child.**

   Authors' response: We appreciate the reviewer's suggestion to discuss factors related to SHS exposure, such as household smoking rules and the presence and/or age of children. We agree that these factors are important for understanding SHS exposure. We have added text and relevant citations to the Introduction to describe how cross-country differences in knowledge of SHS-caused diseases may reflect broader contextual determinants of SHS exposure, such as the adoption of smoke-free homes, implementation and enforcement of public smoking bans, and SHS mass media campaigns.

6. **Second, the workplace is a major source of SHS. As smoke-free policies are enacted, building knowledge about the dangers of SHS typically follows in order to build and sustain public support. The authors should discuss any sort of policy or public messaging that may be influencing SHS knowledge. This ties into the second paragraph ("The WHO Framework…") but the authors should give a broader explanation of policy and SHS knowledge.**

   Authors' response: We agree on the importance of adding a broad explanation of policy and SHS knowledge. Please see our response to Comment 3 above. We have also expanded the Discussion to summarize how policy environments (i.e., health warnings, mass media campaigns) may influence SHS knowledge.

7. **When discussing the "W" within MPOWER, the authors should also describe a theoretical framework through which raising awareness can change behavior and perceptions. As it is stated, "knowledge alone does not change behavior" which means there are other mechanisms at play. The authors should use a theoretical framework to describe these mechanisms. I would recommend Social Cognitive Theory (SCT) as this is**

the most used in tobacco prevention and control programs, however, the authors are free to select whichever theory works best for them. Sample citation is provided belowa. Mantey DS, et al. (2024). Social cognitive theory and health behaviors. Health Behavior: Theory, Research, and Practice, 98.

Authors' response: We thank the reviewer for this suggestion. We have expanded our discussion of health warnings on cigarette packs as a strategy to communicate specific smoking- and SHS-related health risks. We have used SCT as a theoretical framework to explain the potential mechanisms that may influence risk perceptions and behavior. That is, we emphasize that health warnings may influence behavior (e.g., smoking cessation, actions to protect self and others against SHS exposure) through interactions with personal, environmental, and other behavioral factors (e.g., increased knowledge of smoking- and SHS-caused diseases, self-efficacy to cut down on or to quit smoking, smoke-free environments).

8. **Methods: This section is strong and boosted by the supplemental materials. Minor comments and questions below.**

The combination of "no" and "don't know" may cause bias. Did the authors consider sensitivity analyses that excluded "don't know" similar to the "refused" response? Alternatively, could the authors provide supplemental information on what proportion of the sample was in the "don't know"category?

Authors' response: For subjective questions assessing respondents' knowledge, "don't know" is a meaningful and valid response, as it indicates uncertainty or lack of knowledge regarding the item being assessed. In contrast, a "refused" response does not provide information about the respondent's knowledge and may reflect reasons unrelated to knowledge (e.g., unwillingness to answer). Therefore, when estimating the prevalence of correct knowledge, "don't know" responses were retained in the denominator, whereas "refused" responses were excluded. We acknowledge the reviewer's concern regarding potential bias; however, excluding "don't know" responses would systematically inflate estimates of correct knowledge by restricting the denominator to respondents who expressed an opinion. This concern is particularly relevant for items assessing less commonly known information, where uncertainty itself reflects limited knowledge. Consistent with established survey methodology and prior ITC analyses, "don't know" responses were therefore retained in the denominator to provide a more conservative and substantively valid estimate of population knowledge.

The measures should be better described. For example, the smoking status variable seems to code non-smokers with smokers who do not smoke daily. This should be made clearer.

Authors' response: For the main analyses, smokers were defined as adults (18+) who currently smoke combustible tobacco products: at least monthly in ITC surveys and daily or non-daily in GATS surveys. Non-smokers were not

combined with smokers in any primary analyses. The only analyses involving non-smokers were sensitivity analyses examining changes in knowledge by smoking status, which were conducted using GATS data only. ITC data were not used for these analyses because ITC longitudinal surveys primarily recruit adults who smoke, and respondents who quit between waves do not constitute a population-representative sample of quitters.

We recognize that the sentence in footnote (f), *"Adults who quit smoking at follow–up survey waves are weighted as adults who smoke",* may be unclear. We have revised footnote (f) for clarity as follows: *"ITC longitudinal cohort surveys primarily recruit adults who smoke at baseline. Respondents who quit smoking at subsequent survey waves may be recontacted as part of the longitudinal cohort; however, these individuals do not constitute a population-representative sample of adults who have quit smoking. Although these respondents retain adults who smoke-based survey weights for longitudinal analyses (to represent the previous-wave population of adults who smoke), they are analytically classified as adults who quit and are not included as adults who currently smoke in our analyses. For this reason, analyses comparing changes in knowledge by smoking status (adults who currently, formerly, and never smoked) were conducted using GATS data only."*

Results: This section is dense and could be either shortened or better organized. The authors provide most of their data in this section which is typically a strength. However, the country specific data creates a large number of results to report. I would urge the authors to either limit to the most compelling results to highlight here or, alternatively, to organize the results into smaller paragraphs in which they report similar outcomes and directions.

Authors' response: We agree that reporting country-specific results generates a large volume of findings, which is expected given that we examine both current levels of knowledge and changes in knowledge across over 20 years in 25 ITC countries and 32 GATS countries. We have already organized the Results section by structuring the findings for each of the five disease outcomes to highlight patterns and directions of effects across countries, rather than presenting results for each country. Specifically, for each disease outcome we report: (1) overall levels of knowledge at the most recent survey wave, with the range from the highest- to lowest-knowledge countries, and (2) overall changes in knowledge over time, and results for countries with statistically significant increases or decreases (we have revised to list countries and their associated percentage point (PP) change values, with detailed results provided in Supplementary tables 7 to 10). This approach allows us to highlight the most compelling findings while improving readability and reducing detailed country-specific reporting.

9. **Discussion: This section is off to a good start but could be improved with more thorough interpretation of results. 9. The authors touch on important implications and interpretations but do not go far enough. For example, the**

discussion of country-level differences in education strategies does not speak to the most common form of health education (i.e., school-based programs for kids) which may explain some of the changes seen. A recent study (cited below) found that school-based interventions corresponded with a 69% increase in SHS harm perceptions and 70% increase in support of smoke-free policies. a. Chido-Amajuoyi OG, et al. (2024). Exposure to school-based tobacco prevention interventions in low-income and middle-income countries and its association with psychosocial predictors of smoking among adolescents: a pooled cross-sectional analysis of Global Youth Tobacco Survey data from 38 countries. BMJ open, 14(2), e070749.10. The limitations are robust. The authors should be given credit for this.11. The authors should call back to the WHO MPOWER framework from the introduction. Speak more to the necessity and the procedures through which to achieve the W piece.

Authors' response: We have added text on the importance of mass media campaigns for strengthening the impact of smoke-free laws on knowledge of SHS harm perceptions and we have cited the Chido-Amajuoyi et al. 2024 study in the Discussion. We have also expanded the Discussion to summarize potential mechanisms for how health warnings may influence behavioral outcomes.

Reviewer 3's comments:

1. **Abstract: and "ITC Project longitudinal cohort surveys with replenishment are administered in 31 countries using a standardised set of questions, data collection protocol, and sampling design that allows for cross-country comparisons." And "GATS is a nationally representative repeated cross-sectional household survey of persons aged 15+years that uses a standard core questionnaire, sample design, and data collection methods." Can the survey design please be described in more detail? Have sample weights been applied to account for survey designs? Can the authors please comment on the potential impact of COVID-19 on the survey data?**

   Authors' response: The ITC Project surveys use stratified, multistage probability sampling to recruit nationally representative cohorts of adults who smoke, with longitudinal follow-up and replenishment samples added at subsequent waves to maintain representativeness. This design enables both within-country longitudinal analyses and cross-country comparisons using a standardized questionnaire and protocol.

   The Global Adult Tobacco Survey (GATS) is a nationally representative repeated cross-sectional household survey that uses a multistage cluster sampling design with stratification, based on a standardized core questionnaire and uniform data collection procedures across countries.

   All analyses incorporated survey design features, including sampling weights, strata, and primary sampling units, to account for unequal probabilities of

selection and complex survey designs. Weighted survey logistic regression models were used for pooled GATS data, and weighted generalized estimating equations were used for pooled ITC data. Marginal estimates were generated using the predicted marginal standardization (PREDMARG) method based on these weighted models.

Regarding COVID-19, some survey waves were conducted during or shortly after the pandemic period, which may have affected field operations, response rates, or data collection modes in certain countries. However, the primary outcomes examined—knowledge of smoking- and SHS-caused diseases—are relatively stable cognitive constructs and are unlikely to be strongly influenced by short-term disruptions. Moreover, analyses focused on within-country trends over time and correlations between ITC and GATS estimates, which reduces the likelihood that COVID-19-related disruptions would systematically bias the findings.

2. **"We used data from all survey waves to test for significant yearly change in knowledge, and data from the first and last survey waves to compute average yearly change in knowledge" Can the authors please confirm whether survey methods have stayed consistent over time?" Sample demographics and methodological details for ITC Surveys are described elsewhere [18] and in online individual country technical reports" Can the sample demographics please be reported here, perhaps in the supplementary material? Can the authors please confirm if there was any missing data?**

   Authors' response: The survey methods are consistent across ITC countries, with changes to survey mode (e.g., telephone to web-based survey) in some countries. Details are provided in Supplementary table 1. Survey methods are consistent across GATS countries.

   Our study reports findings from 25 ITC countries and 32 GATS countries, with data collected across multiple survey waves over a 20-year period. To ensure transparency while maintaining feasibility, we referred readers to the individual ITC and GATS country reports, which are publicly available online and provide complete sample demographics for all countries and survey waves.

   Missing data for the knowledge variables was minimal across both ITC surveys and GATS. On average, item-level missingness was less than 1% in most survey waves. there are no missing data for the control variables, including age, sex, and smoking status. Given the very low proportion of missing data, complete-case analyses were conducted, and the impact of missing data on the study findings is expected to be negligible.
3. **"Generalized estimating equations and weighted multinomial regression models assessed country-level prevalence of knowledge for three smoking and two SHS outcomes. Multilevel models estimated all country mean prevalence and tested within country linear trends over time for five disease indicators by survey. Across 12 countries, with data for both surveys, correlations between ITC and GATS assessed consistency across**

indicators." [Abstract] and "Point estimates of the prevalence of knowledge for each disease outcome by country were calculated using weighted multinomial survey logistic regression models for pooled repeated cross-sectional GATS data, and generalized estimating equations for pooled longitudinal ITC data. All models incorporated survey design information (strata and primary sampling units for multi-stage sampling surveys), adjusted for sex, age, smoking status (daily, non-daily), and time-in-sample (ITC countries).Point estimates of all-country means of the prevalence of knowledge for each disease outcome across all ITC countries and all GATS countries, respectively, were calculated using a multi-level model (two levels: individual and country) with random intercepts at the country-level and with roughly equal weights for each country. " and "Analysis of the longitudinal ITC data used generalized estimating equations to account for within-subject correlations given that a proportion of individuals participated in more than one survey. Analysis of the repeated cross-sectional GATS data used weighted multinomial survey logistic regression models. Two-level models (individual and country) with random intercepts and random slopes of survey years at the country-level were used to test for significant yearly change in knowledge for each health effect within countries." Appropriate modelling methods have been applied, which are summarised concisely in the Abstract. Was a trend analysis such as join point regression analyses considered by the authors?

Authors' response: The timing of surveys was irregular across countries, and the primary outcomes were model-based prevalence estimates derived from complex survey data rather than simple annual rates. Instead, our analytic objectives focused on estimating country-specific prevalence and testing overall linear changes in knowledge over time while appropriately accounting for complex survey design, weighting, within-subject correlation (ITC), and between-country heterogeneity. These objectives are more appropriately addressed using generalized estimating equations, weighted multinomial survey regression, and multilevel models with random intercepts and slopes.

Joinpoint regression cannot readily accommodate individual-level covariate adjustment, survey weights and design features, or the hierarchical structure of individuals nested within countries that are central to the ITC and GATS study designs. Moreover, given the limited number of time points, joinpoint analyses would be underpowered and potentially unstable, increasing the risk of identifying spurious change points.

For these reasons, linear trend modeling within the established survey regression and multilevel framework provides a more robust and interpretable assessment of temporal change in knowledge in this context.

4. **"As such, we report the estimated all-country means of knowledge for each health effect without the associated confidence intervals, which would refer to a hypothetical superpopulation of countries." Can the authors please**

clarify whether pooled supercountry estimates account for heterogeneity between countries (e.g. weighted by different sample sizes proportional to country population size)?

Authors' response: The all-country mean estimates were obtained from multilevel models with random intercepts at the country level, which explicitly account for between-country heterogeneity in baseline levels of knowledge. In these models, individuals are nested within countries, and country-level random effects allow prevalence to vary across countries beyond what is explained by individual-level covariates. Individual-level sampling weights were incorporated in the multilevel models; however, the all-country means were calculated by giving approximately equal weight to each country at the country level, rather than weighting by country sample size or national population size.

We employed this approach to avoid having countries with larger samples disproportionately influence the pooled estimates and to reflect an average across participating countries rather than a population-weighted global estimate. Consequently, the pooled estimates should not be interpreted as representing a superpopulation of countries or the global population, particularly given that the included countries were not randomly selected. For this reason, we report point estimates of the all-country means without confidence intervals, as the associated uncertainty would refer to a hypothetical superpopulation of countries rather than to the finite set of countries included in the study.

5. **"Pearson correlations (r) between ITC and GATS mean prevalence estimates of knowledge for each of the five diseases were used to examine consistency of estimates from different surveys conducted in the 12 overlapping countries. " Were normality and linearity assumptions checked prior to the application of Pearson correlation?**

   Authors' response: Yes, linearity was checked visually through scatter plots. The trends are approximately linear and there is no curvature trend. Normality was checked using multiple tests: Shapiro–Wilk, Kolmogorov–Smirnov, Cramér–von Mises, and Anderson–Darling. Most of these did not reject normality, except for primary lung cancer where the Shapiro–Wilk and Kolmogorov–Smirnov tests yielded p-levels of 0.05 and 0.03, barely exceeding the α=0.05 criterion for statistical significance. Pearson correlations are known to be robust to the mild non-normality that was present here (and we are aware that Pearson correlations are not robust for extreme departures from normality).

   We also assessed the convergence of the ITC and GATS findings through the more robust Spearman correlation, and that yielded the same strong associations between the two data sets.

6. **"Knowledge about diseases caused by smoking among adults who smoke was highest for lung cancer(ITC=92.2%, GATS=91.9%), followed by heart**

disease (ITC=84.7%)/heart attack (GATS=80.0%), and stroke (ITC=75.0%, GATS=66.1%) (Supplementary table 5)...."Can confidence intervals please be generated for all point estimates, and communicated throughout the Results?

Authors' response: We have already stated in the Methods section that "Point estimates of all-country means of the prevalence of knowledge for each disease outcome across all ITC countries and all GATS countries, respectively, were calculated using a multi-level model (two levels: individual and country) with random intercepts at the country-level and with roughly equal weights for each country. These point estimates cannot be used to make inferences to the global population. As such, we report the estimated all-country means of knowledge for each health effect without the associated confidence intervals, which would refer to a hypothetical superpopulation of countries. The all-country mean estimates in this analysis therefore only serve as the central point of selected countries."

7. **The Figures are clear and informative. A thorough discussion on the study limitations is provided by the authors. Thanks for providing a copy of the STROBE checklist.**

Authors' response: Thank you for the positive feedback.

VERSION 2 - REVIEW

**Reviewer**	**2**
**Name**	**Mantey, Dale S.**
**Affiliation**	**UTHlth Sch Publ Hlth**
**Date**	**23-Feb-2026**
**COI**	

The authors have made substantial improvements. The manuscript is much clearer and will make for a sound contribution to the literature.

VERSION 2 - AUTHOR RESPONSE

Dr. Dale Mantey's comment:

1. **The authors have made substantial improvements. The manuscript is much clearer and will make for a sound contribution to the literature.**

Authors' response: Thank you very much for the acknowledgement of our efforts to improve the manuscript. We appreciate the positive feedback and

are pleased to hear that our revisions have enhanced the clarity and overall contribution of the study.
